# Cang-ai volatile oil alleviates nasal inflammation via Th1/Th2 cell imbalance regulation in a rat model of ovalbumin-induced allergic rhinitis

**DOI:** 10.3389/fphar.2024.1332036

**Published:** 2024-05-21

**Authors:** Yang Zhou, Bojun Chen, Yi Fu, Chunping Wan, Huayan Li, Lin Wang, Xiaoyi Huang, Zhao Wu, Gang Li, Lei Xiong, Dongdong Qin

**Affiliations:** ^1^ The First School of Clinical Medicine, Yunnan University of Chinese Medicine, Kunming, China; ^2^ Key Laboratory of Traditional Chinese Medicine for Prevention and Treatment of Neuropsychiatric Diseases, Yunnan University of Chinese Medicine, Kunming, China; ^3^ Yunnan Provincial University Key Laboratory of Aromatic Chinese Herb Research, Kunming, China; ^4^ Yunnan Innovation Team of Application Research on Traditional Chinese Medicine Theory of Disease Prevention at Yunnan University of TCM, Kunming, China; ^5^ The Third Affiliated Hospital, Yunnan University of Chinese Medicine, Kunming, China; ^6^ School of Pharmacy, Yunnan University of Chinese Medicine, Kunming, China

**Keywords:** Chinese aromatic botanical drugs, allergic rhinitis, immune balance, Th1/Th2, cytokines, transcription factors, Cang-ai volatile oil

## Abstract

We previously revealed that Cang-ai volatile oil (CAVO) regulates T-cell activity, enhancing the immune response in people with chronic respiratory diseases. However, the effects of CAVO on allergic rhinitis (AR) have not been investigated. Herein, we established an ovalbumin (OVA)-induced AR rat model to determine these effects. Sprague–Dawley (SD) rats were exposed to OVA for 3 weeks. CAVO or loratadine (positive control) was given orally once daily for 2 weeks to OVA-exposed rats. Behavior modeling nasal allergies was observed. Nasal mucosa, serum, and spleen samples of AR rats were analyzed. CAVO treatment significantly reduced the number of nose rubs and sneezes, and ameliorated several hallmarks of nasal mucosa tissue remodeling: inflammation, eosinophilic infiltration, goblet cell metaplasia, and mast cell hyperplasia. CAVO administration markedly upregulated expressions of interferon-γ, interleukin (IL)-2, and IL-12, and downregulated expressions of serum tumor necrosis factor-α, IL-4, IL-5, IL-6, IL-13, immunoglobulin-E, and histamine. CAVO therapy also increased production of IFN-γ and T-helper type 1 (Th1)-specific T-box transcription factor (T-bet) of the cluster of differentiation-4+ T-cells in splenic lymphocytes, and protein and mRNA expressions of T-bet in nasal mucosa. In contrast, levels of the Th2 cytokine IL-4 and Th2-specific transcription factor GATA binding protein-3 were suppressed by CAVO. These cumulative findings demonstrate that CAVO therapy can alleviate AR by regulating the balance between Th1 and Th2 cells.

## Graphical Abstract

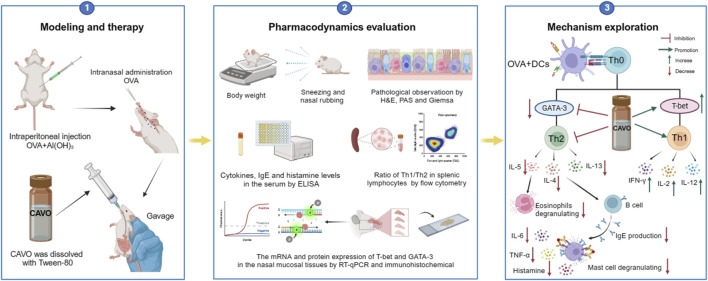

## 1 Introduction

The main cause of allergic rhinitis (AR) is the activation of immunoglobulin (Ig) E after allergen exposure. This condition impacts the nasal mucous membrane and is a common, long-lasting, non-infectious inflammatory disorder. Its primary symptoms include persistent sneezing, nasal congestion, itching in the nasal area, and clear, watery nasal discharge. AR is frequently associated with both asthma and conjunctivitis (Bousquet et al., 2020). Epidemiologically, AR affects 40% of the world’s population and its incidence has progressively increased in developed countries over recent decades. Likewise, the prevalence of AR has increased in developing countries (including China) over the past 20–30 years (Cheng et al., 2018). Though AR is not life-threatening, persistent or recurrent episodes can seriously damage quality of life, productivity, and academic performance due to the lack of curative treatment. AR can also cause fatigue, reduced energy, and poor perception in adult patients; in pediatric patients it mainly causes reduced learning ability, memory loss, and anxiety, and can increase autism symptoms. The prevalence of depression, anxiety, and sleep disorders is higher in patients with AR than in the general population, and allergies can even be a risk factor for suicide (Bousquet et al., 2001).

Though the pathophysiology of AR is not entirely clear, it is widely acknowledged that an imbalance between T-helper type 1 (Th1) cells and Th2 cells during differentiation and cytokine release, which results in a Th2>Th1 immunological response, is the primary etiology (Okano et al., 2011; Meng et al., 2019a). Allergen-specific immunotherapy (ASIT) is the sole potentially curative and specific AR treatment method. Disadvantages of the ASIT approach include long treatment cycles, poor treatment adherence, and wide variation in treatment outcomes (Bousquet et al., 2008). Consequently, there is increasing need for new or alternative methods for relieving AR symptoms.

Complementary and alternative medicine (CAM) has become an increasingly attractive part of standard healthcare. After decades of research, natural products have become a core for novel drug development (Rastelli et al., 2020). Several Chinese aromatic botanical drugs are used in allergic disease treatment, with fewer side effects compared with synthetic drugs (Ahmed, 2018) and stable curative effects (Park et al., 2021). Botanical drugs and aromatherapy are common CAM rhinologic treatments (Roehm et al., 2012), showing anti-inflammatory, immune-modulating, and antibacterial effects (Grazul et al., 2023). Volatile oils, which are secondary metabolites of aromatic plants, can be administered by inhalation, orally, or directly on the skin (Thangaleela et al., 2022).

Cang-ai volatile oil (CAVO) is a complicated volatile oil preparation, derived from a clinically effective prescription for the treatment of respiratory disorders and prepared from a variety of Chinese aromatic botanical drugs. The composition of CAVO has been determined by gas chromatography-mass spectrometry (GC-MS) (Chen et al., 2019), its 10 most abundant volatile metabolites are listed in Table 1. A 2015 patent was obtained for this formula under the State Intellectual Property Office of China (authorization number: CN201310388088.4). CAVO has been demonstrated to regulate T-cell activity, which improves immunological response in chronic respiratory disorders (Xie Y et al., 2015). In asthmatic mice, intragastric CAVO treatment significantly improves airway remodeling and inflammation, and suppresses the immune responses dominated by alveolar macrophages (Yu, 2023). Functional near-infrared spectroscopy was used to show that inhaling CAVO alleviates depression mood and cortical excitability (Wei et al., 2023).

**TABLE 1:** The top 10 species of volatile compounds in CAVO.

Top no.	Identification	Metabolites	Molecular formula	Structure	%
1	Phenols	Eugenol	C_10_H_12_O_2_	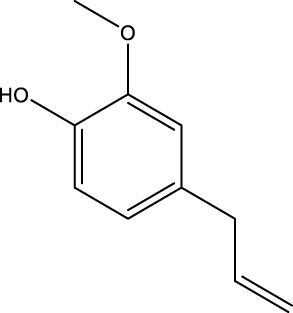	42.21
2	Terpenoids	1,8-Cineole	C_10_H_18_O	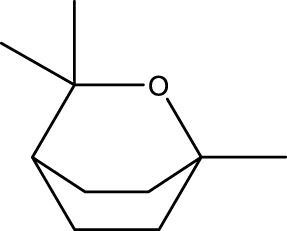	11.91
3	Terpenoids	Patchouli alcohol	C_15_H_26_O	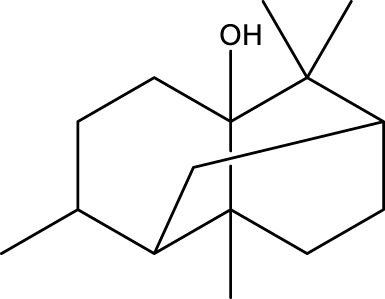	9.03
4	Phenols	Acetyl eugenol	C_12_H_14_O_3_	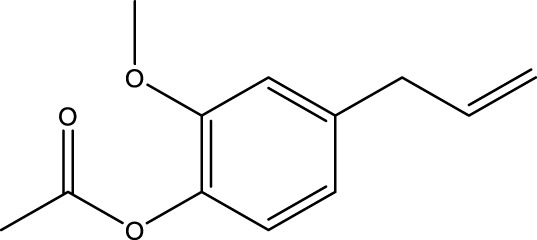	8.17
5	Terpenoids	Linalool	C_10_H_18_O	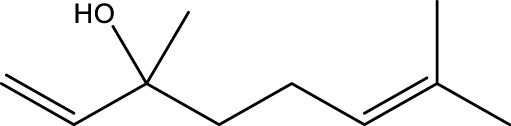	3.66
6	Terpenoids	Linalyl acetate	C_12_H_20_O_2_	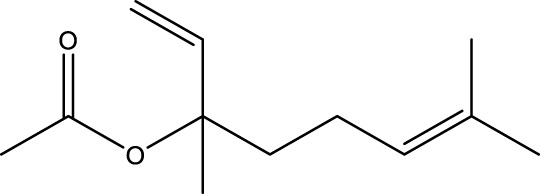	2.74
7	Terpenoids	β-Caryophyllene	C_15_H_24_	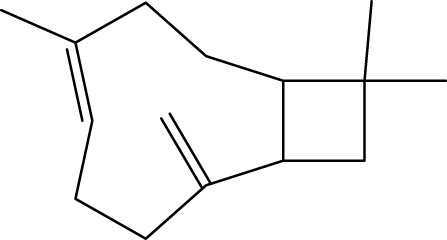	1.95
8	Terpenoids	Terpinen-4-ol	C_10_H_18_O	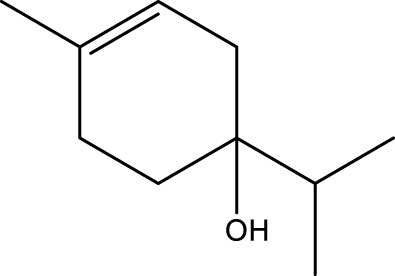	1.95
9	Terpenoids	Cinene	C_10_H_16_	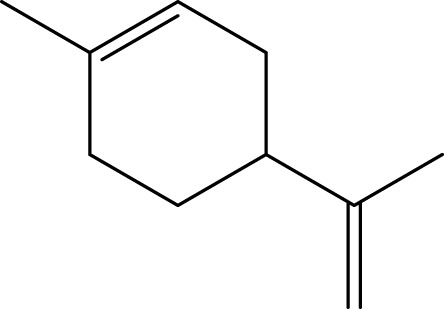	1.66
10	Terpenoids	α-Terpineol	C_10_H_18_O	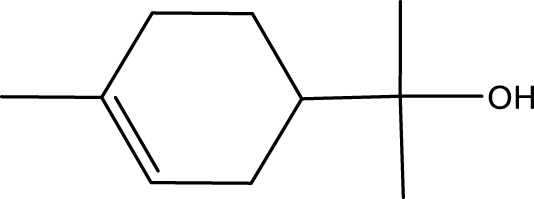	1.08

The top 10 species of volatile substances in CAVO are also unique phytochemical metabolites found in botanical drugs and spices, which have a wide range of biological activities. Among them, eugenol possesses immunomodulatory properties, making it a common agent in inhalation and aerosol therapies for upper respiratory mucous membrane inflammation and cold prevention (Pytko-Polończyk and Muszyńska, 2016). In nasal secretion cultures induced by lipopolysaccharide (LPS), 1,8-cineole greatly reduced the number of mucus-filled nasal cells and downregulated mucin genes (Nakamura et al., 2020). This suggests that 1,8-cineole may be used as a mucolytic/secretory agent, a Th1/Th2 cytokine immunomodulator, and an adjuvant in the treatment of chronic obstructive pulmonary disease, asthma, and sinusitis (Juergens, 2014). Patchouli alcohol significantly reduces LPS-induced levels in TNF-α, IL-1β, and IL-6 mRNA in RAW264.7 cells (Xian et al., 2011). In immune system modulation experiments in Kunming mice, patchouli alcohol boosts production of antibodies by suppressing immune cell activity, stimulating monocyte-macrophage system functions (Liao et al., 2013). Acetyl eugenol, a eugenol derivative, displays a wide range of biological activities, including antioxidant, antibacterial, and anti-inflammatory (Abdou et al., 2021). Linalool ameliorates airway inflammation and mucus hypersecretion in OVA-exposed allergic asthmatic mice by down-regulating inflammatory mediators and the MAPKs/NF-κB signaling pathway (Kim et al., 2019). Linalyl acetate inhibits thymic stromal lymphopoietin production and mRNA expression by blocking the caspase-1/NF-κB pathway and reducing intracellular calcium levels, thereby treating atopic and inflammatory diseases (Moon et al., 2018). Β-caryophyllene dose-dependently inhibits mast cell degranulation and histamine release through activation of the AdipoR1/AdipoR2 and Nrf2/HO-1 signaling pathways, and effectively alleviates systemic and cutaneous anaphylactic shock model induced by metabolites 48/80 in BALB/c mice (Pathak et al., 2021). Terpinen-4-ol is highly contact toxic to house dust mites and can be used as a natural acaricide (Yang et al., 2013). Cinene has significant anti-inflammatory and antioxidant activity, and can prevent and repair respiratory damage (Santana et al., 2020). Intraperitoneal injection of α-Terpineol to mice 60 min before ovalbumin-induced asthma significantly decreases leukocyte migration and lowers pleural cavity TNF-α levels (Pina et al., 2019).

The beneficial potential of CAVO in AR has not been evaluated. Hence, we hypothesized that CAVO might possess therapeutic advantages in the AR context. Herein, we investigated the impacts of CAVO on an ovalbumin (OVA)-induced AR rat model. The goal was to explore whether amelioration of AR symptoms elicited by CAVO treatment was associated with regulation of the Th1/Th2-associated immune response.

## 2 Materials and methods

### 2.1 Study approval

Experimental animals were cared for according to the Guide for the Care and Use of Laboratory Animals (National Institutes of Health, Bethesda, MD, United States) and the study was approved by the Animal Ethics Committee of Yunnan University of Chinese Medicine (R-062022161).

### 2.2 Animals

Male and female Sprague–Dawley rats (190 ± 10 g; *n* = 60; 6–7 weeks) were purchased from SPF Biotechnology (Beijing, China), which were randomly divided into six groups. Each group had five males and five females. Each group had five males and five females. The rats were housed in the specific pathogen-free (SPF) unit of the Experimental Animal Center at Yunnan University of Chinese Medicine under controlled conditions: 20°C ± 2°C, 12-h light/dark cycle, and 65% humidity. Ad lib regular nourishment and hydration were accessible. Animal health was regularly monitored as part of routine care. Rats were allowed to acclimatize to the housing environment for at least 7 days prior to the experiment. Up to five rats were housed in each plastic cage with autoclaved corncob bedding material (SPF Biotechnology).

### 2.3 Welfare-related assessments and interventions

Prior to the experiment, laboratory animal technicians were properly trained, a reasonable animal experiment implementation plan was developed, and it was ensured that the housing environment was clean, comfortable, and safe for experimental animals. The team also ensured that each animal could achieve natural behaviors (i.e., turning around, standing, stretching, lying down, licking, and grooming). During the experiment, cages were cleaned and disinfected regularly, including replacing sterilized bedding. Appropriate gentleness was used to catch animals, to avoid causing anxiety, panic, pain, or injury. Animals were observed as part of daily management; abnormal animal behavior was investigated and ameliorative measures were taken. After the experiment, euthanasia was carried out in accordance with humanitarian principles (e.g., the experiment endpoint considered animal suffering duration, disposal occurred after confirmed death).

### 2.4 Preparation and identification of the primary CAVO metabolites

CAVO is composed of 10 traditional Chinese medicinal botanical drugs: dried rhizome of *Atractylodes lancea* (Thunb.) DC. (100 g, deposition voucher: 20221103-01); dried leaves of *Artemisia argyi* H. Lév. and Vaniot. (100 g, deposition voucher: 20221103-02); dried and ripe fruit peel of *Zanthoxylum bungeanum* Maxim. (50 g, deposition voucher: 20221103-03); dried aboveground parts of *Pogostemon cablin* (Blanco) Benth. (50 g, deposition voucher: 20221103-04); dried stamen of *Eugenia caryophyllata* (Thunb.) (50 g, deposition voucher: 20221103-05); dried aboveground parts of *Mosla chinensis* Maxim. (50 g, deposition voucher: 20221103-06); dried rhizomes of *Kaempferia galanga* L. (50 g, deposition voucher: 20221103-07); dried aboveground parts of *Eupatorium fortunei* Turcz. (50 g, deposition voucher: 20221103-08); dried rhizome of *Acori tatarinowii rhizome* (50 g, deposition voucher: 20221103-09); and dried and ripe fruits of *Amomi fructus rotundus*. (50 g, deposition voucher: 20221103-10).

All medicinal materials were purchased from the Yunnan Hehe Traditional Chinese Medicine Pieces Co., Ltd., identified by Professor Jie Zhang of Yunnan University of Chinese Medicine, and stored in the First Clinical Medical College of Yunnan University of Chinese Medicine. Extraction was conducted using hydrodistillation. Medicinal materials were weighed in proportion, ground into powder, mixed, and soaked in 8 times the amount of water for 4 h before extraction for 6 h. The extraction process followed that described in the chapter *2204 Volatile Oil Determination Method A* in the 2020 version of the *Chinese Pharmacopoeia* (Pharmacopoeia Commission China, 2020). The total volatile oil yield was 3.6% (v/w), and the extract was dried over anhydrous sodium sulfate and stored in brown glass at 4°C (Gao et al., 2022). Preparation considered recent best practice guidelines for pharmacological and toxicological research studies of natural chemical characterization of extracts (Heinrich et al., 2022). The high-performance liquid chromatography analysis was used for quality control with the eugenol as the marker compound (Zhang et al., 2020).

### 2.5 Experimental drugs

CAVO was dissolved with Tween-80 in double-distilled water to prepare a solution, which was kept at 4°C until needed. GC-MS was used to analyze CAVO, as previously described (Chen et al., 2019), with an aim to identify and measure its metabolites. OVA was purchased from Millipore Sigma (catalog number: A5503-5G; Burlington, MA, United States) and used according to the manufacturer’s instructions (i.e., at room temperature, away from light and humidity). The positive control pharmaceutical, loratadine capsules (batch number: 210402), was obtained from Hainan Huluwa Pharmaceutical Group (Hainan, China).

### 2.6 CAVO acute toxicity test

Based on preliminary results, it was determined that the minimum and maximum lethal doses of CAVO via intragastric administration were 1 mL/kg and 5.8 mL/kg, respectively. A total of 70 SPF Kunming mice (weighing 20 ± 2 g) were randomly allocated to 7 groups, each consisting of 10 mice with an equal distribution of males and females. Prior to the experiment, the mice underwent a 12-h fasting period and were administered varying doses of CAVO intragastrically, with a dose ratio of 1:0.75 and an administration volume of 0.4 mL/10 g. Three hours after administration, the mice were allowed *ad libitum* access to food and water. Over a 14-day observation period, some animals exhibited symptoms such as irritability, salivation, ataxia, urinary incontinence, reduced activity, curling up, and apnea; some animals succumbed to these effects within 1–3 days. Surviving mice showed an increase in body weight after the 14-day feeding period. Surviving animals were euthanized by cervical dislocation. Post-mortem examination revealed lung congestion and thinning of the stomach and intestinal walls in a few mice, with no abnormalities observed in other major organs. The CAVO median lethal dose (LD_50_), calculated using the modified Couch method, was 1.647 g/kg (95% confidence limit: 1.361–1.993 g/kg) (Li, 2014).

### 2.7 AR rat model establishment

**FIGURE 1:**
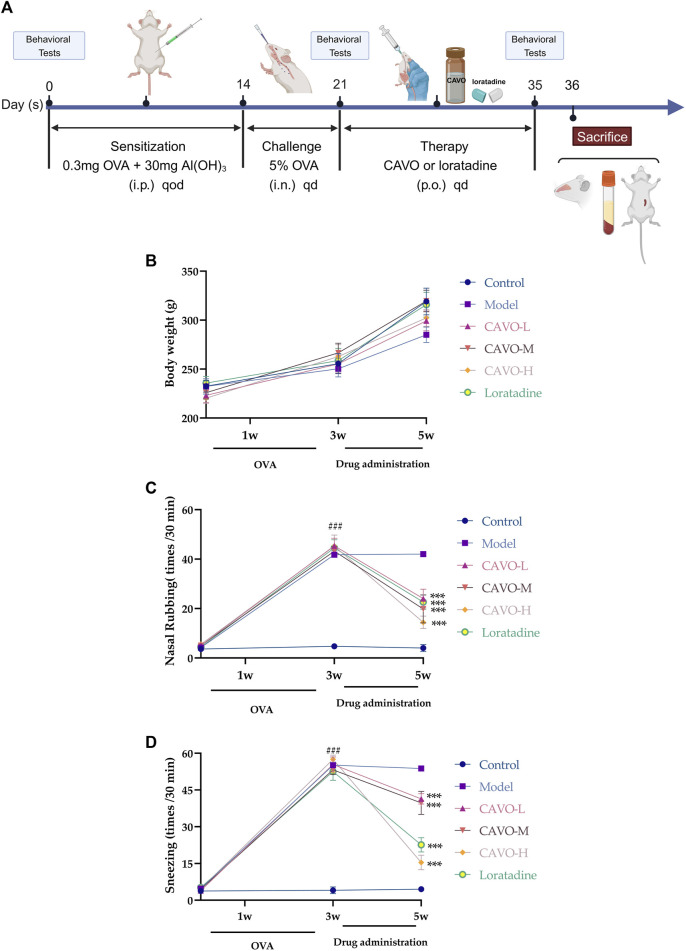
Effect of CAVO on the body weight and nasal symptoms of rats with OVA-induced AR. **(A)** Flowchart. **(B)** Effects of CAVO on bodyweight. **(C)** Nasal rubbing. **(D)** Sneezing. Oral administration of CAVO (20.59, 41.18, 82.36 mg/kg) and loratadine (1 mg/kg) decreased the frequency of nasal rubbing and sneezing in rats with AR significantly. Data are presented as the mean ± SEM (n = 10 per group). Repeated-measures ANOVA was performed to analyze the metrics in the CAVO and loratadine groups and compare them with the control group. Significant differences at ^###^ *p* < 0.001 compared with the control group and ^***^ *p* < 0.001 compared with the model group. Abbreviations: i.p., intraperitoneal; i.n., intranasal, p.o., *per ora*.

The OVA-induced AR rat model was developed based on the literature and previous explorations of successful modeling methods (Ren et al., 2017). The remaining rats (aside from the control group) were used as AR model animals. On experimental days 1, 3, 5, 7, 9, 11, and 13, rats were intraperitoneally (i.p.) injected with physiologic (0.9%) saline (1 mL) and OVA (0.3 mg) for antigen sensitization, with aluminum hydroxide (30 mg) as an adjuvant. Subsequently, rats were challenged through nasal drops applied bilaterally (∼50 μL of 5% OVA solution on each side once daily on days 14–21) (Figure 1A). The control group was injected (i.p.) with 0.9% saline and nasal drops were applied (using the same method described above) with an identical volume of 0.9% saline.

### 2.8 Grouping and treatment protocol

Fifty AR rats were randomly divided into five equal groups: (1) AR model (10 mL of physiologic saline/kg body weight); (2) AR model plus CAVO low dosage (CAVO at 1/80 of median lethal dosage [LD_50_] = 20.59 mg/kg); (3) AR model plus CAVO middle dosage (CAVO at 1/40 of LD_50_ = 41.18 mg/kg); (4) AR model plus CAVO high dosage (CAVO at 1/20 of LD_50_ = 82.36 mg/kg); and (5) AR model plus loratadine (loratadine at 1 mg/kg). Administration of agents was via the oral route for 2 weeks. After 2 weeks, the rats were killed. Nasal tissues, serum, and spleen were collected for analysis.

### 2.9 Bodyweight and behavioral tests

All rats were weighed and behaviorally tested before and after model establishment and after drug treatment. The number of sneezes and nose rubs were assessed for 30 min by investigators blinded to the experimental protocol.

### 2.10 Nasal mucosa histopathology

Nasal mucous membrane samples were collected, immediately preserved in 10% neutral formalin for 2 days at room temperature, treated with EDTA buffer (0.1 M) for 2 weeks to remove calcium deposits, and subsequently enclosed in paraffin. The samples were sliced into 4 um sections to undergo staining (hematoxylin and eosin, PAS, Wright-Giemsa) and count eosinophils, goblet cells, and mast cells.

### 2.11 Cytokines, IgE, and histamine

Serum levels of IFN-γ, IL-2, IL-12, TNF-α, IL-4, IL-5, IL-6, IL-13, IgE, and histamine were detected by enzyme-linked immunoassay kits (Elabscience, Wuhan, China), and well absorbance was measured at 450 nm using a BioTek Epoch 2 (Agilent Technologies, Santa Clara, CA, United States) microplate reader.

### 2.12 Subset analyses of spleen Th1/Th2 cells

A rat spleen was placed in a culture dish. Phosphate-buffered saline (PBS) and erythrocyte lysate were added dropwise, followed by grinding with a rod until no large particles were suspended. A single-cell suspension was obtained by passage through a 200-mesh filter. A single-cell suspension (2×10^7^ cells/mL) was screened. The counted cell suspension was added to a 48-well plate containing RPMI-1640 medium. GolgiPlug (550583; BD Pharmingen, San Diego, CA, United States) was added to each reaction well and then left to stand for 4–6 h in a 37°C incubator. We wished to determine the different subpopulations of regulatory T-cells. Single-cell suspensions were collected into flow tubes. Fixable Viability Stain (564406; BD Pharmingen) was added, followed by vortex-mixing, and incubation was undertaken in the dark. The suspension was centrifuged (1,500 rpm for 5 min, at room temperature) to remove the supernatant, and stained with a cluster of differentiation CD3 and CD4 surface antibodies. Finally, samples were examined using a flow cytometer (BD FACSCanto™ II; Beckman Coulter, Fullerton, CA, United States) and results were analyzed using FlowJo (www.flowjo.com/).

The antibodies used for CD3^+^CD4^+^ IFN-γ^+^ IL-4^+^ were those against fluorescein isothiocyanate (FITC)-anti-rat CD3 (559975; BD Pharmingen), phycoerythrin (PE)-Cy7-anti-rat CD4 (201516; Biolegend, San Diego, CA, United States), Alexa Fluor-anti-rat IFN-γ (507810; Biolegend), and PE anti-rat IL-4 (511906; Biolegend).

The antibodies used for CD3^+^CD4^+^ T-bet^+^ GATA-3^+^ were those against FITC-anti-rat CD3 (559975; BD Pharmingen), PE-CY7-anti-rat CD4 (201516; Biolegend), PE-T-bet/T-box transcription factor 21 (TBX21; 4B10; sc-21749 PE; Santa Cruz Biotechnology, Santa Cruz, CA, United States), and Alexa Fluor-mouse anti-GATA3 (560068; Biolegend).

### 2.13 mRNA expression levels of T-bet and GATA-3

Using TRIzol^®^ Reagent, total RNA was isolated from nasal mucosa samples. We used an ultraviolet spectrophotometer to assess RNA purity. Reverse transcription of RNA to complementary DNA was completed with Prime Script RT Reagent Kit containing gDNA Eraser (Takara Biotech, Shiga, Japan). Using the TB Green^®^ Premix Ex TaqTM II kit (Tli RnaseH Plus; Takara Biotechnology), real-time polymerase chain reaction was performed on complementary DNA samples. We employed the endogenous reference enzyme glyceraldehyde 3-phosphate dehydrogenase (GAPDH). Relative mRNA expression of T-bet and GATA-3 was calculated using the 2^−ΔΔCT^ method. The primer sequences we used (sense and antisense, respectively) were: 5′-TTA​TAC​GTC​CAC​CCA​GAC​TCC​C-3′ and 5′-CTC​ACC​GTC​ATT​CAC​CTC​CAC-3′ (138 bp) for T-bet; 5′-GCCAGGCAAGATGAGAAAGAG-3′and 5′-CAT​AGG​GCG​GAT​AGG​TGG​TAA​T-3′ (183 bp) for GATA-3; 5′-CTG​GAG​AAA​CCT​GCC​AAG​TAT​G-3′ and 5′-GGT​GGA​AGA​ATG​GGA​GTT​GCT-3′ (151 bp) for GAPDH.

### 2.14 Immunohistochemistry of T-bet and GATA-3 protein expression levels

Prepared paraffin sections of nasal mucosal tissue were dewaxed and placed in a citrate antigen repair buffer (pH 9.0) repair cassette for antigen repair in a microwave oven. Sections were incubated with 3% hydrogen peroxide in the dark and then washed in PBS (pH 7.4) to block endogenous peroxidase activity. Sections were covered uniformly with 3% bovine serum albumin to block non-specific staining for 35 min at room temperature. Nasal mucosa sections were respectively incubated overnight at 4°C with diluted T-bet polyclonal antibody (PA5104407; Thermo Fisher Scientific, Waltham, MA, United States) and GATA-3 antibody (ab282110; Abcam, Cambridge, UK). Sections were cleaned three times in PBS before being incubated for 50 min at room temperature in the dark with a secondary goat anti-rabbit or anti-mouse IgG antibody that had been biotin-conjugated. Proteins were visualized as brown pigments using a standard protocol for diaminobenzidine. Afterward, sections were re-stained with hematoxylin, dehydrated sequentially in a graded series of alcohol solutions, and sealed with neutral gum. Morphology images were acquired by a tissue-section digital scanning image-analysis system (Pannoramic MIDI; 3DHISTECH, Budapest, Hungary). An identical brown color was selected by digital pathology image-analysis software (Aipathwell; Servicebio, Beijing, China) as a uniform criterion for judging the positivity of all cells. The percentage of positive cells per section was calculated using the equation:

Percentage of positive cells per section = number of positive cells/total number of cells × 100.

Any three high-magnification fields (×200) of each section within each group were analyzed, and an average value was calculated.

### 2.15 Outcomes

Primary outcomes were behavioral tests, changes in nasal mucosa histopathology, levels of cytokines, IgE, and histamine, subsets of spleen Th1/Th2 cells, and mRNA and protein expression levels of T-bet and GATA-3. Body weight was a secondary outcome.

### 2.16 Statistical analyses

GraphPad Prism 9.4.0 (San Diego, CA, United States) was used for graph plotting and quantitative analyses. Data normality was tested using the Kolmogorov–Smirnov test, and all data were normally distributed. Repeated-measurement data were analyzed with repeated-measures analysis of variance (ANOVA). After *post hoc* analyses with one-way ANOVA, Tukey’s multiple-comparison test was conducted. All data are presented as mean ± standard error of the mean (SEM). A significance level of less than 0.05 is considered significant.

## 3 Results

### 3.1 Effect of CAVO on the body weight and nasal symptoms of rats with OVA-induced AR

The body weight of rats per group showed an upward trend (Figure 1B). The increase in average body weight in the model group was slower compared to the control group. The CAVO and loratadine groups experienced a faster increase in body weight compared to the model group. Despite this, there were no notable variances in body mass (*p* > 0.05).

During the experiment, the number of nose rubs (F = 159.19, *p* = 7.54 × 10^−60^) (Figure 1C) and the number of sneezes (F = 326, *p* = 7.97 × 10^−76^) (Figure 1D) changed significantly over time. After 3 weeks of modeling, in comparison to the control group, the OVA-induced group exhibited substantially more nose rubs and sneezes (*p* < 0.05).

In comparison with the model group, the number of sneezes and nose rubs after 2 weeks of medication administration. The number of nose rubs (*p* = 8.61 × 10^−8^ of the low-CAVO-dosage, *p* = 2.03 × 10^−6^ of the middle-CAVO-dosage, *p* < 0.001 of the high-CAVO-dosage, and *p* = 2.85 × 10^−10^ of the loratadine) and the number of sneezes (*p* = 3.17 × 10^−7^ of the low-CAVO-dosage, *p* = 9.99 × 10^−6^ of the middle-CAVO-dosage, *p* = 4.56 × 10^−14^ of the high-CAVO-dosage, and *p* = 2.46 × 10^−13^ of the loratadine) for CAVO groups and the loratadine group were significantly lower. The results stated above indicated that CAVO administration could alleviate OVA-induced nasal allergic symptoms significantly.

### 3.2 Effect of CAVO on the pathologic changes of nasal mucosa in rats with OVA-induced AR

The nasal lining is the primary controller of airflow in the respiratory system and serves as the initial barrier against airborne infectious pathogens. It is crucial to maintain and repair the integrity of epithelial cells and stimulate the immune system to carry out these tasks (Greiner et al., 2011). To study the effects of CAVO on OVA-induced inflammation, nasal mucus secretion, and degranulation of epithelial cells in the nasal mucosal tissue of AR rats, we undertook staining (H&E) of nasal mucosal tissue and eosinophils in each group, goblet cell (PAS staining), and mast cells (Giemsa staining).

**FIGURE 2:**
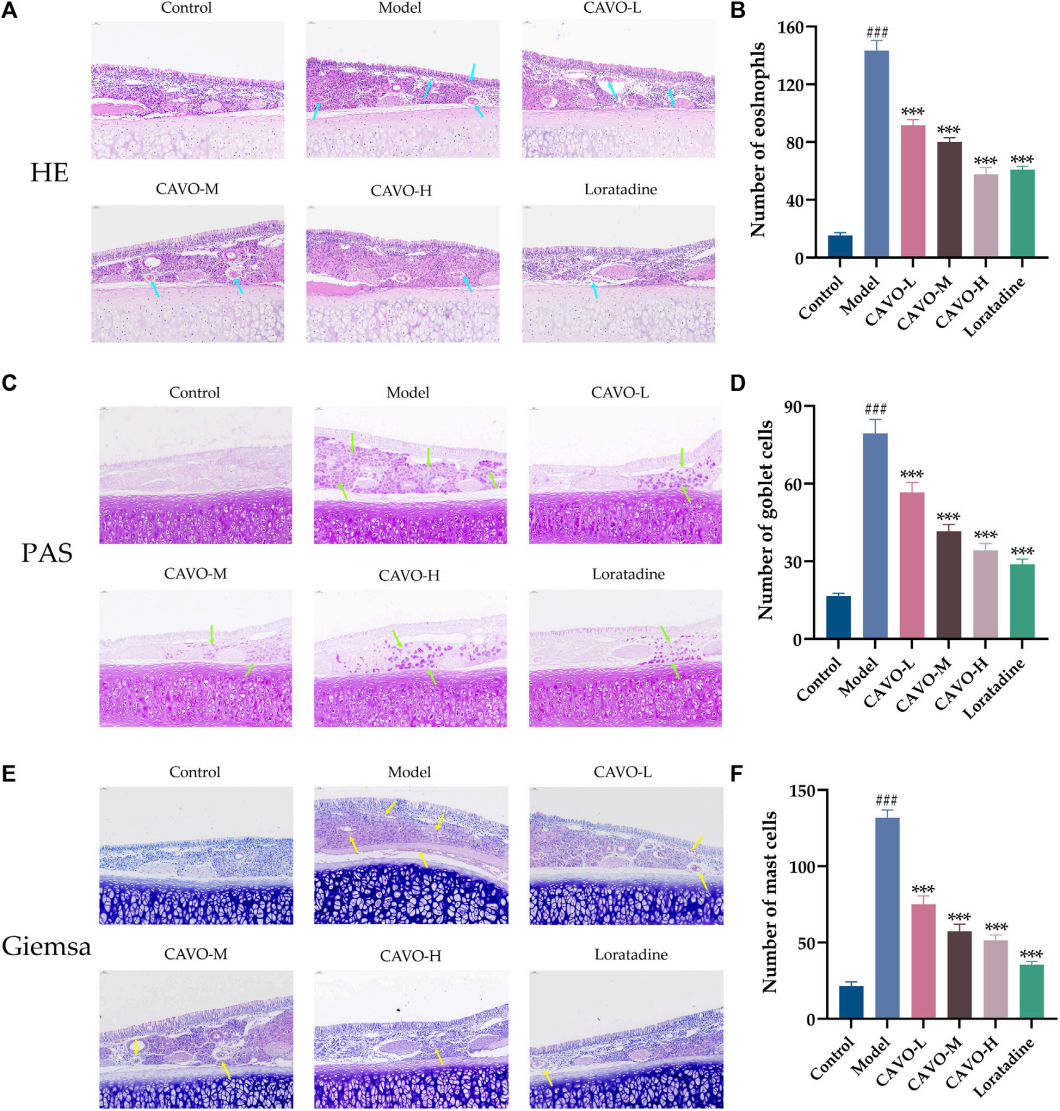
Effect of CAVO on the histopathological changes in the nasal mucosa of rats with OVA-induced AR. **(A)** H&E staining showing histopathological changes and eosinophils in nasal tissues. **(C)** PAS staining showing goblet cells. **(E)** Giemsa staining showing mast cells. Photographs of representative nasal mucosal in each group (×200 magnification; scale bars = 50 μm; arrows indicate positively stained cells). Bar graphs represent the counts of eosinophils **(B)**, goblet cells **(D)**, and mast cells **(F)**. Data are presented as the mean ± SEM (*n* = 5 per group). Significant differences at ^###^ *p* < 0.001 compared with the control group and ^***^ *p* < 0.001 compared with the model group. Comparison among multiple groups was conducted by one-way ANOVA, followed by Tukey’s *post hoc* test. Abbreviations: H&E, hematoxylin, and eosin. PAS, periodic acid-Schiff.

Light microscopy showed that comparatively to control rats, the nasal mucosa of rats in the model group was inverted and interrupted, epithelial cells were detached, mucosa lamina propria capillaries were dilated (or even congested and edematous), many eosinophil infiltrates (*p* = 1.78 × 10^−14^) (blue arrows, Figures 2A, B), proliferative expansion of goblet cells (*p* = 1.15 × 10^−11^) (green arrows, Figures 2C, D), and many proliferative infiltrates of mast cells (*p* = 2.82 × 10^−14^) (yellow arrows, Figures 2E, F). A dosage-dependent reduction of these histopathological changes was observed with both CAVO and loratadine administered orally. CAVO treatment improved epithelial disruption and mesenchymal edema and inhibited eosinophil infiltration (*p* = 1.02 × 10^−7^ of the low-CAVO-dosage, *p* = 2.10 × 10^−9^ of the middle-CAVO-dosage, *p* = 3.78 × 10^−12^ of the high-CAVO-dosage, and *p* = 8.57 × 10^−12^ of the loratadine) and the proliferation of goblet cells (*p* = 5.84 × 10^−4^ of the low-CAVO-dosage, *p* = 2.68 × 10^−7^ of the middle-CAVO-dosage, *p* = 9.57 × 10^−9^ of the high-CAVO-dosage, and *p* = 1.03 × 10^−9^ of the loratadine) and mast cells (*p* = 1.32 × 10^−8^ of the low-CAVO-dosage, *p* = 5.74 × 10^−11^ of the middle-CAVO-dosage, *p* = 1.10 × 10^−11^ of the high-CAVO-dosage, and *p* = 2.41 × 10^−13^ of the loratadine). In the group with high CAVO dosages, improvements were more apparent. Thus, CAVO treatment improved tissue remodeling in the nasal mucosa of rats suffering from AR by reducing infiltration by inflammatory cells.

### 3.3 Effect of CAVO on serum levels of Th1-cell and Th2-cell cytokines, IgE, and histamine of rats with OVA-induced AR

**FIGURE 3:**
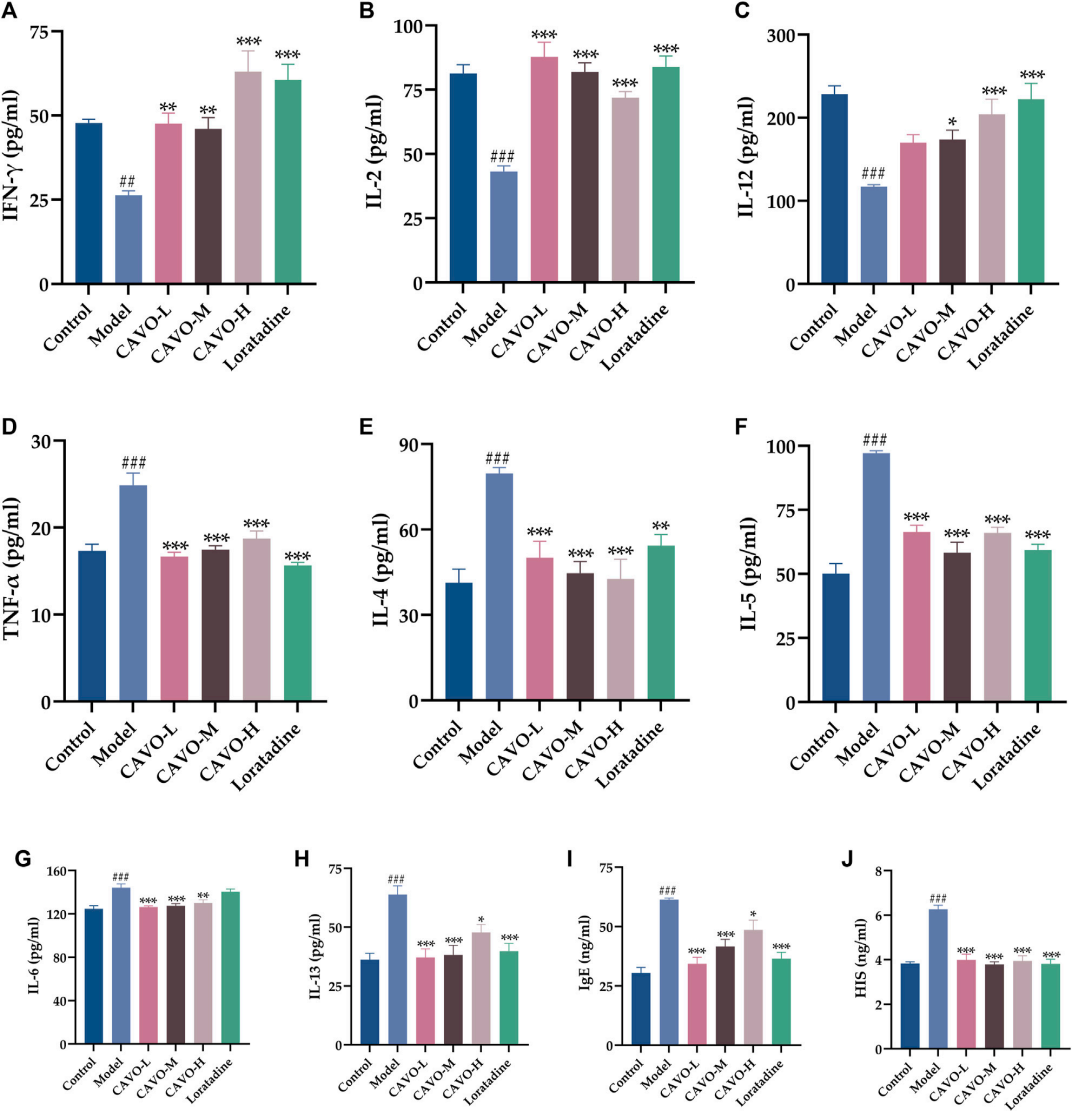
Effect of CAVO on serum levels of Th1 and Th2 cytokines, IgE, and histamine of rats with OVA-induced AR. Levels of **(A)** IFN-γ, **(B)** IL-2, **(C)** IL-12, **(D)** TNF-α, **(E)** IL-4, **(F)** IL-5, **(G)** IL-6, **(H)** IL-13, **(I)** IgE, and **(J)** HIS were evaluated using ELISA. Data are presented as the mean ± SEM (*n* = 10 per group). Significant differences at ^##^ *p* < 0.01 and ^###^ *p* < 0.001 compared with the control group, ^*^ *p* < 0.05, ^**^ *p* < 0.01, and ^***^ *p* < 0.001 compared with the model group. Comparison among multiple groups was conducted by one-way ANOVA, followed by Tukey’s *post hoc* test.

OVA administration significantly decreased expression of INF-γ (*p* = 1.98 × 10^−3^) (Figure 3A), IL-2 (*p* = 3.42 × 10^−8^) (Figure 3B), IL-12 (*p* = 2.33 × 10^−6^) (Figure 3C), but significantly increased expression of TNF-α (*p* = 2.81 × 10^−7^) (Figure 3D), IL-4 (*p* = 1.01 × 10^−5^) (Figure 3E), IL-5, (*p* = 5.26 × 10^−6^) (Figure 3F), IL-6 (*p* = 1.80 × 10^−5^) (Figure 3G), IL-13, (*p* = 8.69 × 10^−6^) (Figure 3H), IgE (*p* = 2.31 × 10^−9^) (Figure 3I), and histamine (*p* = 5.38 × 10^−7^) (Figure 3J).

Two weeks after administration, contrary to the expression of INF-γ, IL-2, and IL-12 in the model group, the expression of INF-γ (*p* = 2.25 × 10^−3^ of the low-CAVO-dosage, *p* = 5.77 × 10^−3^ of the middle-CAVO-dosage, *p* = 6.70 × 10^−8^ of the high-CAVO-dosage, and *p* = 3.74 × 10^−7^ of the loratadine), IL-2 (*p* = 3.52 × 10^−10^ of the low-CAVO-dosage, *p* = 2.28 × 10^−8^ of the middle-CAVO-dosage, *p* = 2.28 × 10^−5^ of the high-CAVO-dosage, and *p* = 5.67 × 10^−9^ of the loratadine), and IL-12 (*p* = 6.31 × 10^−2^ of the low-CAVO-dosage, *p* = 3.77 × 10^−2^ of the middle-CAVO-dosage, *p* = 2.53 × 10^−4^ of the high-CAVO-dosage, and *p* = 7.82 × 10^−6^ of the loratadine) of drug-treated groups was significantly higher. In comparison with expression of TNF-α, IL-4, IL-5, IL-6, IL-13, IgE, and histamine of the model group, expression of TNF-α (*p* = 3.09 × 10^−8^ of the low-CAVO-dosage, *p* = 4.05 × 10^−7^ of the middle-CAVO-dosage, *p* = 2.37 × 10^−5^ of the high-CAVO-dosage, and *p* = 1.12 × 10^−9^ of the loratadine), IL-4 (*p* = 9.07 × 10^−4^ of the low-CAVO-dosage, *p* = 5.87 × 10^−5^ of the middle-CAVO-dosage, *p* = 2.08 × 10^−5^ of the high-CAVO-dosage, and *p* = 6.21 × 10^−3^ of the loratadine), IL-5 (*p* = 3.22 × 10^−6^ of the low-CAVO-dosage, *p* = 4.31 × 10^−5^ of the middle-CAVO-dosage, *p* = 3.09 × 10^−7^ of the high-CAVO-dosage, and *p* = 3.11 × 10^−8^ of the loratadine), IL-6 (*p* = 9.05 × 10^−5^ of the low-CAVO-dosage, *p* = 3.04 × 10^−4^ of the middle-CAVO-dosage, *p* = 3.32 × 10^−3^ of the high-CAVO-dosage, and *p* = 9.02 × 10^−1^ of the loratadine), IL-13 (*p* = 1.76 × 10^−5^ of the low-CAVO-dosage, *p* = 3.78 × 10^−5^ of the middle-CAVO-dosage, *p* = 2.02 × 10^−2^ of the high-CAVO-dosage, and *p* = 1.20 × 10^−4^ of the loratadine), IgE (*p* = 9.54 × 10^−8^ of the low-CAVO-dosage, *p* = 8.39 × 10^−5^ of the middle-CAVO-dosage, *p* = 2.29 × 10^−2^ of the high-CAVO-dosage, and *p* = 6.79 × 10^−7^ of the loratadine), and histamine (*p* = 2.63 × 10^−5^ of the low-CAVO-dosage, *p* = 1.21 × 10^−7^ of the middle-CAVO-dosage, *p* = 6.92 × 10^−6^ of the high-CAVO-dosage, and *p* = 7.63 × 10^−7^ of the loratadine) of drug-treated groups was significantly lower.

### 3.4 Effect of CAVO on the expression of cytokines and specific transcription factors in the CD4^+^ T-cell subpopulation in rat splenic lymphocytes

**FIGURE 4:**
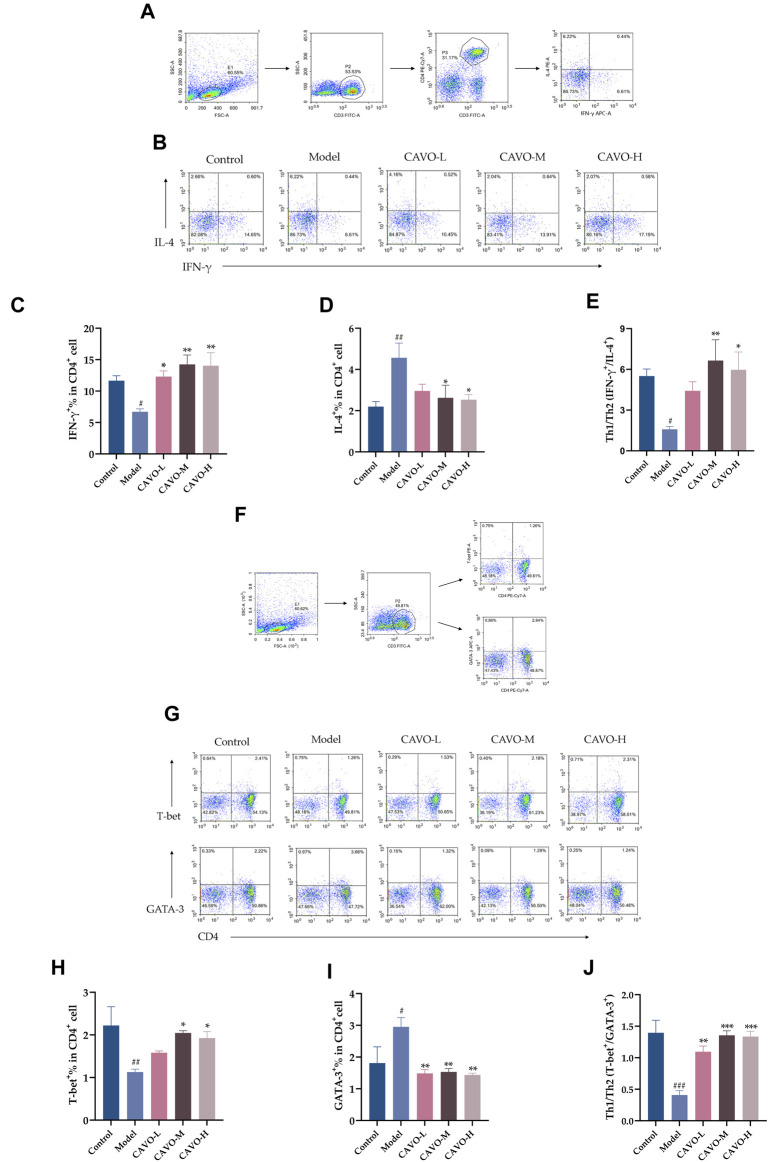
Effect of CAVO on expression of IFN-γ, IL-4, T-bet, GATA-3, and Th1 cells/Th2 cells ratios in a CD4^+^ T-cell subpopulation in rat splenic lymphocytes. Flow cytometry gating strategies: **(A)** Gating strategy for determining the frequencies of IFN-γ and IL-4 expressing CD4^+^T cells per spleen. **(F)** Gating strategy for identifying the frequencies T-bet and GATA-3 producing CD4^+^T cells per spleen. Total lymphocytes were gated on CD3^+^CD4^+^T cells for analysis of expression of IFN-γ, IL-4, T-bet, and GATA3. Representative flow-cytometric analysis of expression of **(B)** IFN-γ, IL-4, **(G)** T-bet, and GATA-3 in CD4^+^ T cells. Percentages of **(C)** IL-4^+^ cells, **(D)** IFN-γ^+^ cells, **(H)** T-bet^+^ cells, and **(I)** GATA-3^+^ cells among CD4^+^ T cells. **(E, J)** Ratios between IFN-γ^+^, IL-4^+,^ and T-bet^+^, GATA-3^+^ are presented to assess the balance between Th1 cells/Th2 cells. Data are presented as the mean ± SEM (n = 5 per group). Significant differences at #*p* < 0.05, ##*p* < 0.01, and ###*p* < 0.001 compared with the control group, *p < 0.05, **p < 0.01, and ***p < 0.001 compared with the model group. Comparison among multiple groups was conducted by one-way ANOVA, followed by Tukey’s *post hoc* test.

CD4^+^IFN-γ^+^ and CD4^+^T-bet^+^ are markers of Th1 cells. CD4^+^IL-4^+^ and CD4^+^GATA-3^+^ are markers of Th2 cells. A flow cytometry analysis of rat spleens was conducted to analyze CD4^+^ T cell levels of cytokines and transcription factors (Figure 4).

As compared with the control group, the model group had reduced expression of IFN-γ^+^ (*p* = 4.28 × 10^−2^) (Figure 4C) and T-bet^+^ (*p* = 5.8 × 10^−3^) (Figure 4H) in the CD4^+^ T cells of rat splenic lymphocytes and increased expression of IL-4^+^ (*p* = 6.83 × 10^−3^) (Figure 4D) and GATA-3^+^ (*p* = 2.77 × 10^−2^) (Figure 4I).

However, after CAVO treatment, expression of IFN-γ^+^ (*p* = 2.02 × 10^−2^ of the low-CAVO-dosage, *p* = 1.80 × 10^−3^ of the middle-CAVO-dosage, and *p* = 2.30 × 10^−3^ of the high-CAVO-dosage), and a higher level of T-bet^+^ (*p* = 3.84 × 10^−1^ of the low-CAVO-dosage, *p* = 2.13 × 10^−2^ of the middle-CAVO-dosage, *p* = 4.97 × 10^−2^ of the high-CAVO-dosage) in CD4^+^ T cells was observed in the CAVO group, but the level of IL-4^+^ (*p* = 7.97 × 10^−2^ of the low-CAVO-dosage, *p* = 2.81 × 10^−2^ of the middle-CAVO-dosage, and *p* = 2.11 × 10^−2^ of the high-CAVO-dosage), and GATA-3^+^ (*p* = 4.32 × 10^−3^ of the low-CAVO-dosage, *p* = 5.65 × 10^−3^ of the middle-CAVO-dosage, and *p* = 3.34 × 10^−3^ of the high-CAVO-dosage) was suppressed. OVA administration resulted in a lower ratio of Th1 cells/Th2 cells: Th1/Th2 (IFN-γ^+^/IL-4^+^) (*p* = 3.54 × 10^−2^) (Figure 4E), and Th1/Th2 (T-bet^+^/GATA-3^+^) (*p* < 1 × 10^−4^) (Figure 4J), but this ratio was reversed upon CAVO treatment: Th1/Th2 (IFN-γ^+^/IL-4^+^) (*p* = 1.65 × 10^−1^ of the low-CAVO-dosage, *p* = 5.9 × 10^−3^ of the middle-CAVO-dosage, *p* = 1.75 × 10^−2^ of the high-CAVO-dosage), and Th1/Th2 (T-bet^+^/GATA-3^+^) (*p* = 1.3 × 10^−3^ of the low-CAVO-dosage, *p* < 1 × 10^−4^ of the middle-CAVO-dosage, *p* < 1 × 10^−4^ of the high-CAVO-dosage). The findings indicated that CAVO has the potential to restore the Th1 cells/Th2 cells imbalance by regulating cytokine secretion and transcription factor expression.

### 3.5 Effect of CAVO on expression of transcription factors in the nasal mucosal tissues of rats with OVA-induced AR

Inhibiting Th2 differentiation while regulating Th1 development, T-bet is a Th1-specific transcription factor. As opposed to that, GATA-3 directs the differentiation of Th2 cells from naive Th cells (Butcher and Zhu, 2021). T-bet and GATA-3 mRNA and protein levels were measured by RT-qPCR and immunohistochemistry in rat nasal mucosa.

**FIGURE 5:**
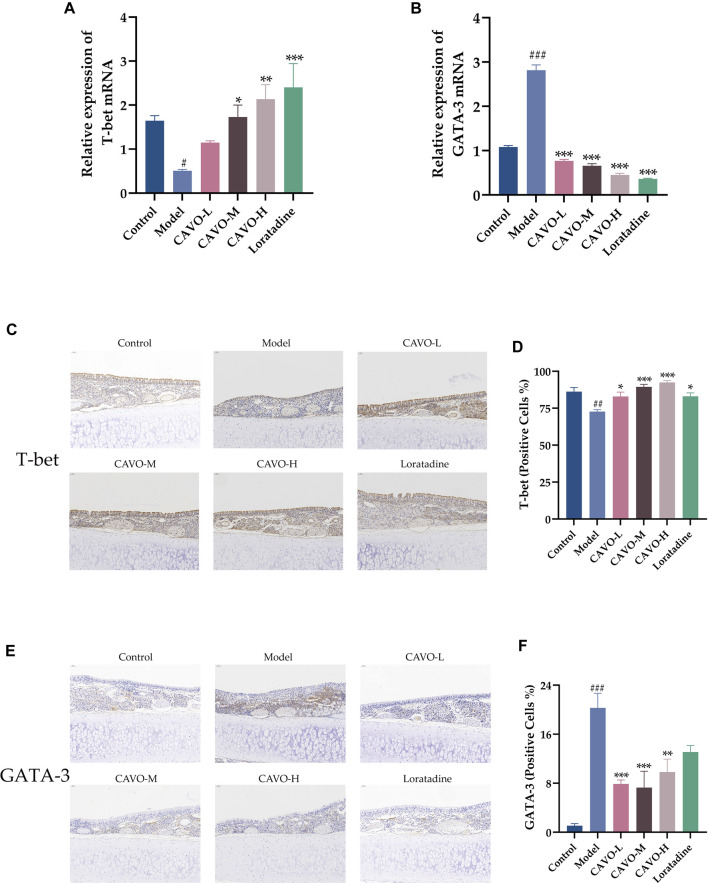
Effect of CAVO on the expression of mRNA and protein of T-bet and GATA-3 in nasal the mucosa of rats with OVA-induced AR. mRNA expression of **(A)** T-bet and **(B)** GATA-3. Representative images of protein expression of **(C)** T-bet and **(E)** GATA-3 in nasal mucosa according to immunohistochemical staining. (×200 magnification; scale bars = 50 µm). **(D)** Quantification of relative T-bet expression. **(F)** Quantification of relative GATA-3 expression. Data are presented as the mean ± SEM (n = 5 per group). Significant differences at #*p* < 0.05, ##*p* < 0.01 and ###*p* < 0.001 compared with the control group, *p < 0.05, **p < 0.01, and ***p < 0.001 compared with the model group. Comparison among multiple groups was conducted by one-way ANOVA, followed by Tukey’s *post hoc* test.

Model group mRNA expression of T-bet (*p* = 3.92 × 10^−2^) (Figure 5A) was significantly reduced compared to the control group, but GATA-3 (*p* = 1.33 × 10^−4^) (Figure 5B) was markedly upregulated. Microscopic observation of the brownish-yellow areas between the epithelial tissues of the nasal mucosa in the model group appeared to show a weaker positive signal for T-bet than that in the control group (Figure 5C). That is, lower protein expression (*p* = 2.45 × 10^−3^) (Figure 5D) but an enhanced positive signal for GATA-3 (Figure 5E), i.e., high protein expression (*p* = 8.94 × 10^−7^) (Figure 5F). In nasal mucosal tissues, treatment with CAVO and loratadine reversed this low T-bet expression significantly (mRNA expression: *p* = 3.91 × 10^−1^ of the low-CAVO-dosage, *p* = 2.41 × 10^−2^ of the middle-CAVO-dosage, *p* = 2.20 × 10^−3^ of the high-CAVO-dosage, *p* = 4.00 × 10^−4^ of the loratadine (Figure 5A); protein expression: *p* = 2.92 × 10^−2^ of the low-CAVO-dosage, *p* = 1.77 × 10^−4^ of the middle-CAVO-dosage, *p* = 1.62 × 10^−5^ of the high-CAVO-dosage, and *p* = 2.74 × 10^−2^ of the loratadine) (Figure 5D). In nasal mucosal tissues, GATA-3 expression (mRNA expression: *p* = 5.92 × 10^−5^ of the low-CAVO-dosage, *p* = 5.29 × 10^−5^ of the middle-CAVO-dosage, *p* = 2.99 × 10^−5^ of the high-CAVO-dosage, and *p* = 1.23 × 10^−4^ of the loratadine (Figure 5B); protein expression: *p* = 5.92 × 10^−4^ of the low-CAVO-dosage, *p* = 3.27 × 10^−4^ of the middle-CAVO-dosage, *p* = 4.01 × 10^−3^ of the high-CAVO-dosage, and *p* = 7.86 × 10^−2^ of the loratadine) (Figure 5F) was inhibited significantly. CAVO intervention reversed the upregulation of GATA-3 expression while rescuing the reduction of T-bet expression, in the nasal mucosal tissues of rats with AR.

## 4 Discussion

The cumulative results demonstrate that CAVO can be used to treat AR because it can regulate Th1/Th2 cell imbalances. In a rat AR model, CAVO administration: alleviated OVA-induced nasal allergy symptoms; ameliorated several hallmarks of nasal mucosa tissue remodeling (inflammation, eosinophilic infiltration, goblet cell metaplasia, and mast cell hyperplasia); suppressed upregulation of serum Th2-related cytokines, IgE, and histamine; restored reduced Th1-related cytokines; and increased OVA-induced T-bet expression in nasal mucosal tissue; and decreased GATA-3 activity in the spleen.

Successful animal models are the vehicle and foundation for human disease and drug research (Meng et al., 2019b). The range of available species for AR experimental animals is currently wide, including primarily guinea pigs, mice, rats, and New Zealand rabbits (Al Hamwi et al., 2022). Rodents have commonly been used as a model because they are cost-effective and relatively easy to work with. The guinea pig was the initial animal selected for AR modelling and has since emerged as the preferred animal for researching allergic diseases due to its heightened susceptibility to nasal or tracheal allergic reactions (Tanaka et al., 1988). However, it is important to note that after sensitization, the complement system of guinea pigs can be easily activated to release anaphylatoxins, which may impact modelling success. Mice are commonly used in AR experiments due to their ease of breeding, reproduction, and genetic purity. Nevertheless, mice have a smaller nasal mucosa area compared with rats, posing challenges to material collection and subsequent nasal mucosa research (Pabst, 2003). In contrast, rats combine the features of guinea pigs and mice, demonstrating a propensity to develop specific IgE antibodies against inhaled allergens and readily manifesting AR behaviors. Consequently, AR researchers globally prefer rats. After weighing the pros and cons of various experimental animals, this study ultimately chose SD rats.

OVA is an antigenic protein with carrier activity that offers the benefits of being non-toxic, minimally irritating, highly immunogenic, and capable of inducing persistent antibody production. The OVA-induced AR model accurately replicates both the clinical symptoms of human AR and the associated pathophysiological changes (Kilic et al., 2019). Indicators play an important part in the clinical diagnosis of AR and are central to the success of the preparation of the AR model. Herein, after intraperitoneal injection and administration of OVA nasal drops, the number of nose rubs and sneezes in model rats was altered, showing that OVA challenge-induced AR-like features in rats are consistent with those previously described (Kamimura et al., 2021). We also determined that these rats’ abnormalities were ameliorated by treatments that provide therapeutic benefit in patients with AR. These results suggest that the rat model of OVA-induced AR herein satisfies the criteria for validation of an animal model (i.e., face, construct, and predictive validities) (Ronca et al., 2017).

AR is an allergic airway illness mediated by Th2 cells and a type 2 immune response (Li et al., 2009). The primary trigger for the emergence of allergy illness is the activation of the type 2 (Th2) phenotype by allergen-specific helper T-cells during the first allergen exposure (i.e., the ‘sensitization phase’). T follicular helper T-cells release cytokines like IL-4 and IL-13 into B-cell follicles, which encourages B cells to produce IgE and IgG1, which activate mast cells and basophils (Yang et al., 2020). In response to allergen stimulation, Th2 effector cells release IL-4, IL-13, and IL-5 (Gowthaman et al., 2019), which cause inflammation and tissue remodeling by infiltrating and activating eosinophils (León and Ballesteros-Tato, 2021) and mucus overproduction (Leόn, 2017). These are the key components of the type-I metamorphic response and initiation of the immune response. In contrast, Th1 cells exert antagonistic effects by inhibiting eosinophil recruitment, reducing immunoglobulin synthesis in B cells (Kuperman et al., 2002), and activating phagocytosis, thus enhancing the body’s anti-infective ability. The dynamic balance between Th1 and Th2 cells is important for maintaining immune system health (Li et al., 2011), and its dysregulation can trigger allergic reactions (Pawankar et al., 2011). Cell imbalance is thus a key cause of AR development (Meng et al., 2019a).

Eosinophil infiltration is a main feature, and the best marker, of mucosal inflammation in AR (Wang et al., 2013). Another sign of nasal mucosal histopathology is the expansion and proliferation of goblet cells. In a healthy state, goblet cells are distributed sparsely in the mucosal columnar epithelium. If stimulated by allergens or inflammation, the number of goblet cells increases rapidly, and they synthesize and secrete mucin to form a mucosal barrier to protect the nasal mucosal epithelium (Zhang et al., 2022). The volume of mast cell infiltration in nasal mucosal tissue is also positively correlated with AR symptoms (Xu et al., 2018). Herein, CAVO treatment dose-dependently improved the pathologic features of AR in the OVA-induced AR model, ameliorating the injury caused by epithelial cell detachment, dampening nasal mucosa interstitial congestion and edema, inhibiting eosinophil infiltration, and inhibiting the proliferation of goblet cells and mast cells.

In AR, histamine plays an important part in itching, sneezing, and nasal congestion (Yokota et al., 2015). After allergen excitation in the nose, the histamine level is increased in the nasal lavage of allergic individuals compared with that of non-allergic individuals (Li et al., 2023). CAVO lowered the serum histamine level in rats with OVA-induced AR, alike that seen with the positive control (loratadine). CAVO thus demonstrated potential as an anti-histamine drug derived from natural botanical products. These findings demonstrate, for the first time, the outstanding therapeutic effect of CAVO in rats with OVA-induced AR. Additionally, rats treated with CAVO did not exhibit mortality or any other severe negative consequences. Hence, CAVO is safe for rats at doses capable of exerting anti-allergic effects.

IFN-γ and IL-4 are key factors regulating serum IgE concentration, and an imbalance in their levels is closely associated with AR (Steelant et al., 2019). IFN-γ is a characteristic anti-inflammatory factor secreted by Th1 cells that inhibits the proliferation of Th2 cells while promoting the differentiation of primitive CD4^+^ T-cells into Th1 cells. IFN-γ promotes the activation and proliferation of macrophages, mediates cellular production of antibodies, and enhances effective immune responses to pathogenic bacteria and exogenous infection factors (Gu et al., 2017). IL-4 is a characteristic pro-inflammatory factor secreted by Th2 cells, and the reduction of IL-4 is effective in suppressing AR symptoms (Steelant et al., 2018). Thus, IL-4 is crucial in causing the nasal epithelium to become more dysfunctional and in promoting the infiltration of immune cells, including lymphocytes, neutrophils, and eosinophils, into the nasal mucosa *in situ* (Steelant et al., 2019). A recent study confirmed that reducing IL-4 levels contributes to nasal epithelial resistance to environmental allergens and pathogens (Li et al., 2021). Herein, in AR model rats, CAVO treatment restored the substantial reduction in the serum IFN-γ level induced by OVA, increased the proportion of IFN-γ in the CD4^+^ subpopulation of splenic lymphocytes, and resulted in IL-4 levels being significantly reduced.

IL-2 is secreted by Th1 cells (Steelant et al., 2018) and has anti-inflammatory and proinflammatory roles. By encouraging the production of IL-4R and IL-4, strong and persistent IL-2 signaling strengthens the aggregation of Th2 cells, enabling a positive IL-4 feedback loop to start and maintain the Th2-cell phenotype (Kasumagić-Halilovic et al., 2018). Dendritic cell-derived IL-12 triggers the differentiation of Th1 cells by increasing IFN-γ production and T-bet expression, which is a transcription factor unique to Th1 cells (Zhu et al., 2012). IL-6 is an important immune and inflammatory mediator regulating various cellular functions (Liao et al., 2008). Increased propensity of Th2 cells and allergy symptoms are brought on by loss-of-function mutations in IL-6 signaling, especially the IL-6 receptor (Sobota et al., 2008). A recent study showed that IL-6 inhibited IL-2 signaling during early activation of T-cells and also had a critical and T-bet non-dependent role in suppressing Th2 cell differentiation (Bachus et al., 2023).

TNF-α enhances the expression of inflammatory cells, including eosinophils, in respiratory vascular endothelial cells and infiltration into nasal mucosal tissues, thereby leading to AR (Hinds et al., 2013). Inhibition of TNF-α expression can reduce the risk of allergic reactions (Erkalp et al., 2014). IL-13 responds to allergens and parasites similarly to IL-4 and synergistically stimulates mast cells (León, 2022). IgE production is considered an important step in the induction of the type-I allergic reaction (Gandhi et al., 2017). Cytokine recruitment, namely, IL-4, IL-5, IL-6, and TNF-α, encourages IgE synthesis in the first phases of an allergic reaction (Ayoub et al., 2003). Consequently, inhibition of Th2 cytokine levels could reduce the severity of various rhinitis types (Kappen et al., 2017). Herein, CAVO administration significantly decreased the levels of TNF-α, IL-5, IL-6, IL-13, and IgE, and increased levels of IL-2 and IL-12 in the serum of rats with OVA-induced AR, which in turn corrected the imbalance between Th1 cells and Th2 cells by regulating the levels of cytokines and IgE.

T-bet is a key transcription factor that induces the differentiation of primary CD4^+^ T-cells into Th1 cells (Kim et al., 2012). Recently, the first instance of full autosomal recessive T-bet deficiency was documented. The growth of IFN-γ producing cells was interfered with by this human T-bet defect (Kiwamoto et al., 2006). A Th2-skewing of T-bet-deficient CD4^+^ cells may also cause inflammation of the upper respiratory tract, peripheral eosinophilia, and an increase in Th2-cytokines IL-4, IL-5, and IL-13 (Dutta et al., 2013). Interestingly, T-bet proteins are also important regulators of T-cell downregulation in the asthmatic airway (Szabo et al., 2000). Hence, T-bet plays a significant role in the prevention of allergic airway diseases that are initiated by Th2 cells and triggered by Th2 cells.

GATA-3 has a central role in the Th2 immune response (Yang et al., 2021). GATA-3 can not only stimulate the expression of Th2-related cytokines (Naito et al., 2011) and regulate IgE levels (Zhang et al., 1999), it can inhibit Th1-cell differentiation (Yang et al., 2021). One clinical study found that GATA-3 expression is higher in patients suffering from AR (Kim et al., 2018). Control of GATA-3 expression is crucial for initiating Th2 and Th1 responses (Maes et al., 2011), so GATA-3 is recognized as an excellent candidate for the management of allergic diseases (Malmhäll et al., 2007). T-bet inhibits the differentiation program of Th2 cells by downregulating GATA-3 expression directly through epigenetic repression of the GATA-3 locus and inhibiting GATA-3 function through protein interactions (Zhu et al., 2012). T-bet and GATA-3 are thus important indicators for the evaluation of Th1/Th2 imbalance in AR (Eifan et al., 2012).

We demonstrated herein that OVA-induced AR increases in GATA-3 expression are largely reversed by CAVO treatment, and that the previously downregulated expression of T-bet induced by OVA is upregulated by CAVO. Restoration of Th1/Th2 homeostasis was associated with CAVO regulating the expression of the transcription factors T-bet and GATA-3.

AR and its corresponding condition belong to the category of ‘Bi-Qiu’ in traditional Chinese medicine (TCM). According to ancient Chinese physicians, the main pathophysiology of AR was external pathogenic factors such as wind, heat, cold and phlegm dampness, as well as the dysfunction of viscera, including the lungs, spleen, and kidneys. TCM has been used to treat AR for thousands of years, with many traditional botanical formulations in wide clinical practice use (Cheng et al., 2018). The famous classic formula Yu-ping-feng powder (YPFP), which originated from the book *Danxi’s Experiential Therapy*, has the effect of consolidating resistance to ward off external pathogens; it is commonly used to prevent and treat immunodeficiency and allergic diseases. Clinical studies have reported that YPFP appears to have an ameliorative effect on symptoms such as nasal itching and sneezing (Cheong et al., 2022), and reduces the levels of interleukins like IgE and IL-4 in patients with AR (Fang et al., 2014). Xiao-qing-long Tang (XQLT) (from the book *Shang-Han-Lun* by *Zhongjing Zhang*, who is regarded a medical genius by later generations), has the effect of warming the lung to dissipate cold, which can effectively treat respiratory diseases (Wang et al., 2020). A systematic review and meta-analysis of randomized controlled trials demonstrated that oral XQLT can relieve AR symptoms and is effective and safe for clinical use (Yan et al., 2022). XQLT has been suggested to have immunopharmacological properties, which were recently evaluated in a study using OVA as an antigen. XQLT ameliorates type 2 immune response in OVA-induced AR mice by regulating ILC2s through inhibition of IL-33/ST2 and JAK/STAT pathways (Zhang et al., 2023).

Chinese aromatic medicines are pungent and diffuse, with the effects of opening the orifices and promoting qi circulation. They can also enhance lung function and defensive qi and guard against pathogenic factors, similar to the immune functions of the mucous membranes. The results herein show that IgE increased significantly in AR rats after CAVO treatment, which may be related to the fact that CAVO stimulates the body’s immune response, enhancing nasal mucosa-based resistance. Aromatherapy has been found to reduce the total nasal symptom score, especially in nasal obstruction, and improved quality of life among patients with chronic AR (Choi and Park, 2016). CAVO is not only a representative aromatic volatile oil preparation in aromatherapy (Cui et al., 2022), it embodies the core TCM principle of ‘treating disease from the root’. The effect of aromatics to dredge/open the orifices can reduce the disturbance of pathological substances to patients with AR. Eliminating dampness and turbidity can sterilize and disinfect, eliminate allergens, and purify the living environment. The effect of strengthening healthy qi and eliminating pathogens can fundamentally enhance patient immunity.

Nevertheless, this study was inconclusive in identifying the material basis and key targets of the CAVO anti-AR effect, which can be further explored and validated by conducting single-metabolite controlled studies and using relevant target inhibitors or target knockout models. Second, CAVO was orally administered herein, so it will be necessary to determine its efficacy via intranasal administration. Finally, clinical studies will be essential to validate the immunomodulatory effects of CAVO on AR.

## 5 Conclusion

These cumulative results suggest that CAVO treatment significantly attenuates both AR-like symptoms in OVA-induced rats (including number of nose rubs and sneezes) and nasal mucosa tissue remodeling. Further evaluation revealed that CAVO therapy regulated the AR-caused imbalance between Th1 and Th2 cells, simultaneously attenuating the Th2 immune response and boosting the Th1 immune response. These findings support the use of CAVO as a supplementary strategy for the prevention and treatment of AR by illuminating its immunomodulatory potential.

## Data Availability

The original contributions presented in the study are included in the article/Supplementary Material, further inquiries can be directed to the corresponding authors.
