# Supplementary figures and images for: Cang-ai volatile oil alleviates nasal inflammation via Th1/Th2 cell imbalance regulation in a rat model of ovalbumin-induced allergic rhinitis (part 1 of 2)
Source: Front Pharmacol. 2024 May 21;15:1332036. doi: 10.3389/fphar.2024.1332036 (PMC11148258; doi:10.3389/fphar.2024.1332036)

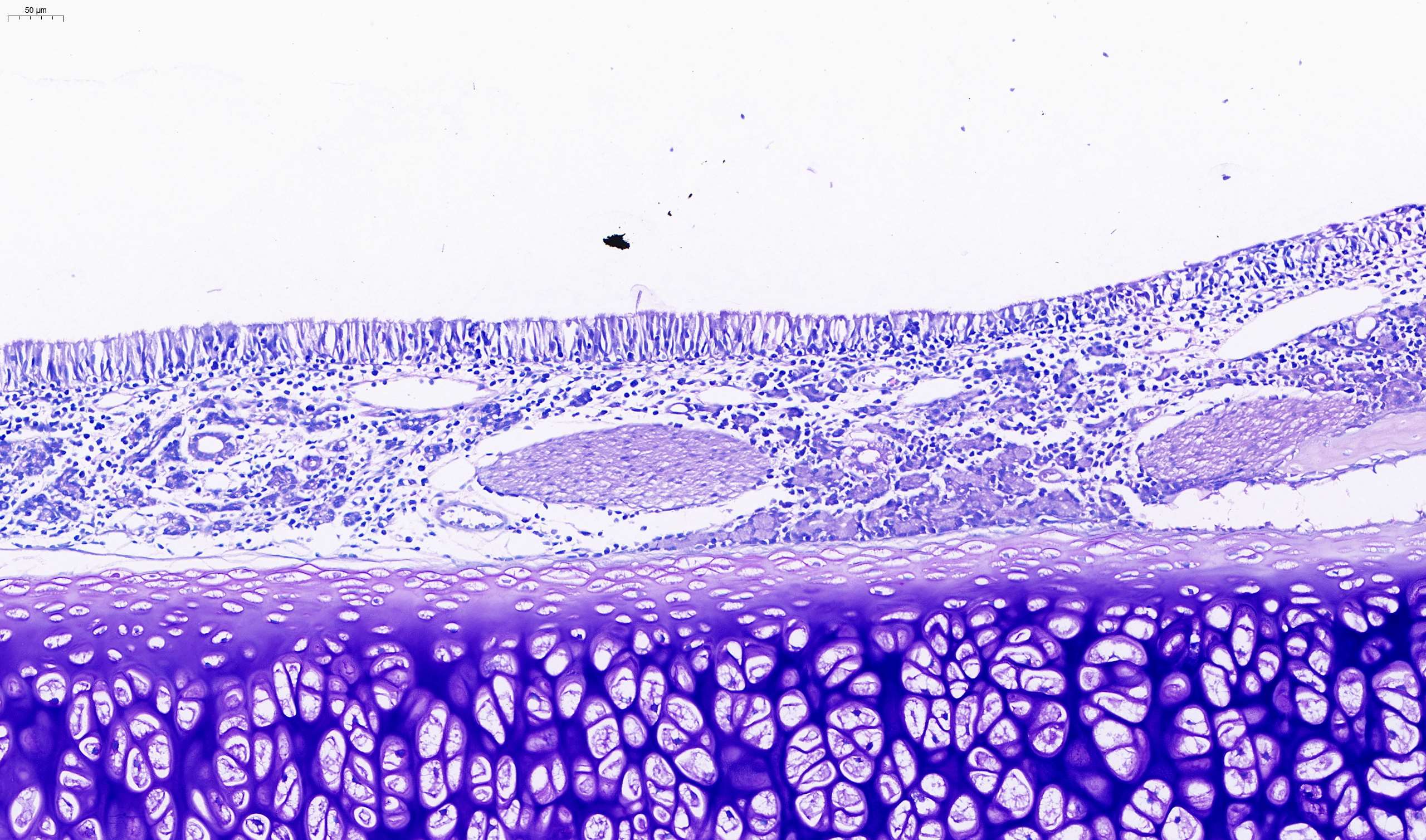

Supplement: Supplementary file 1 [file DataSheet3.ZIP › Microscopy images-Giemsa_200x_50um/CAVO-H/CAVO-H 1 Giemsa_200x_50um_1.jpeg]

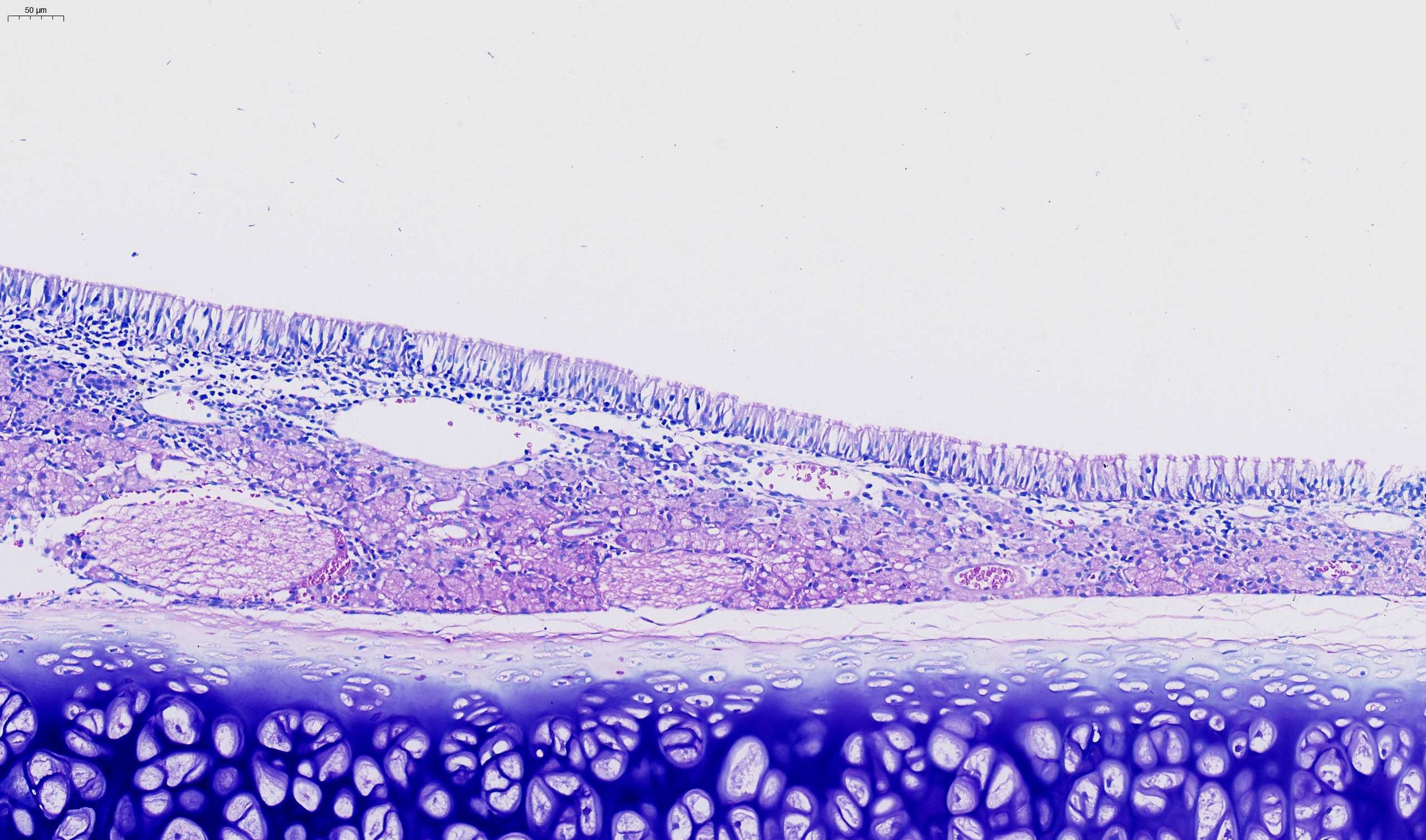

Supplement: Supplementary file 1 [file DataSheet3.ZIP › Microscopy images-Giemsa_200x_50um/CAVO-H/CAVO-H 2 Giemsa_200x_50um_1.jpeg]

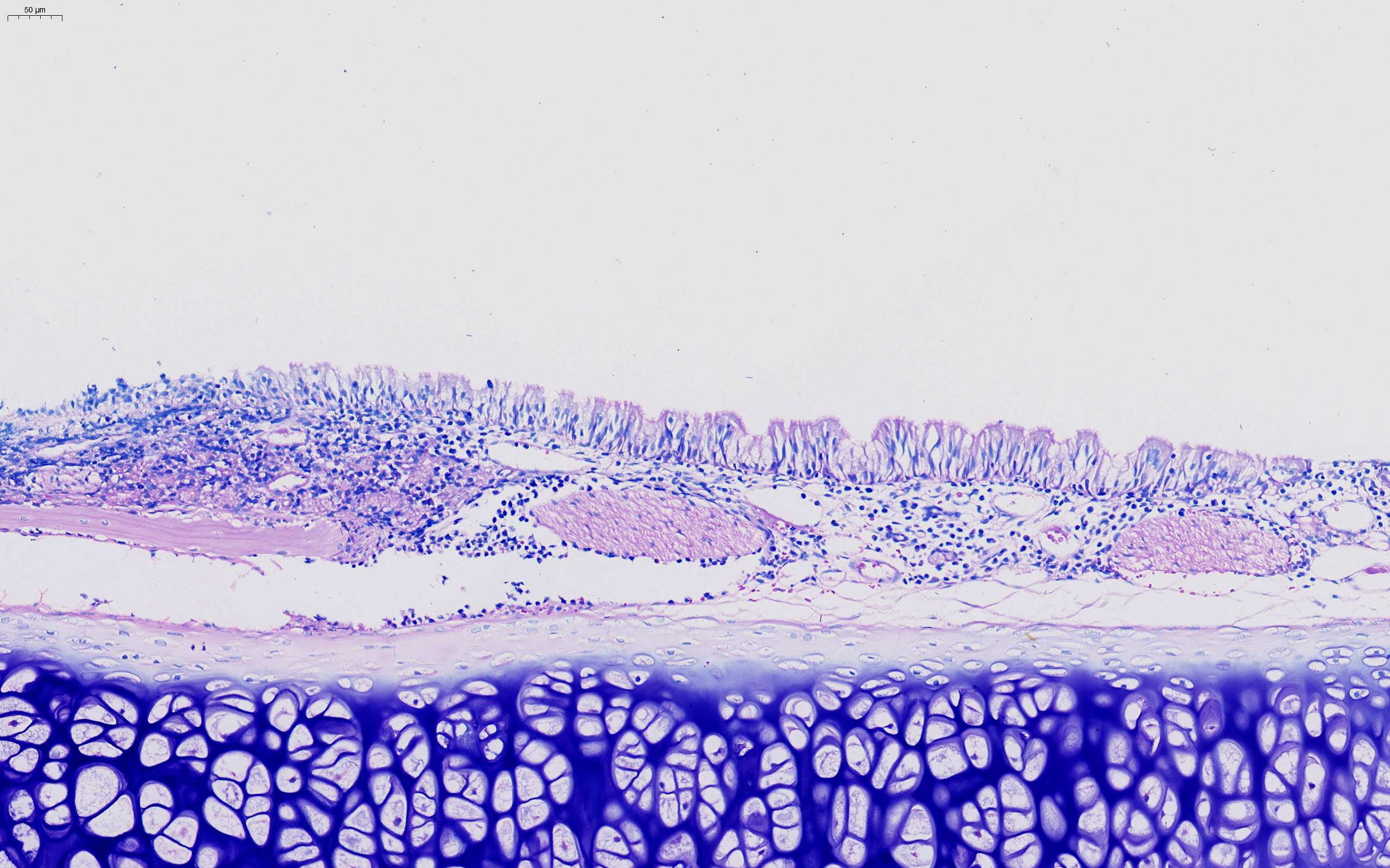

Supplement: Supplementary file 1 [file DataSheet3.ZIP › Microscopy images-Giemsa_200x_50um/CAVO-H/CAVO-H 3 Giemsa_200x_50um_1.jpeg]

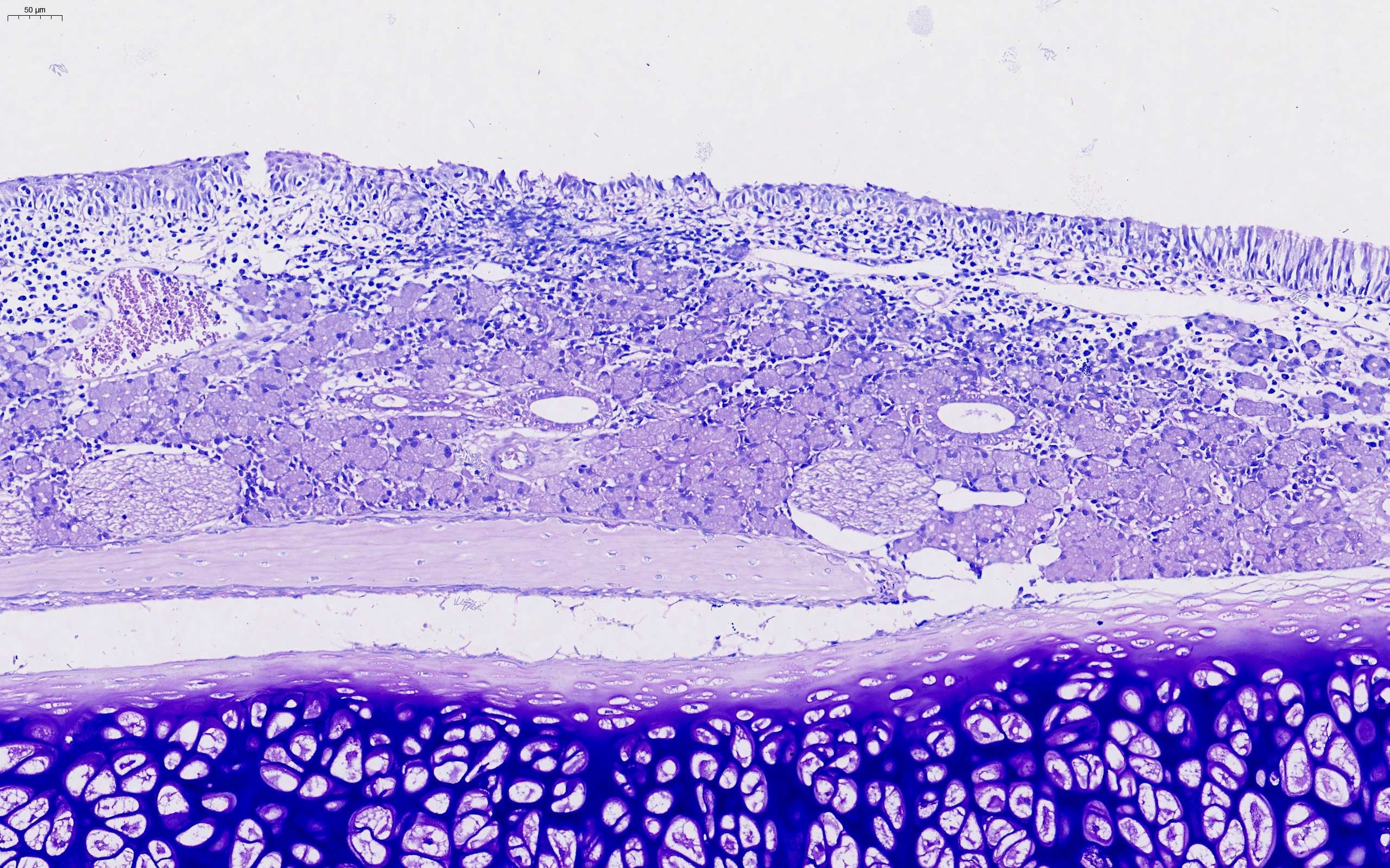

Supplement: Supplementary file 1 [file DataSheet3.ZIP › Microscopy images-Giemsa_200x_50um/CAVO-H/CAVO-H 4 Giemsa_200x_50um_1.jpeg]

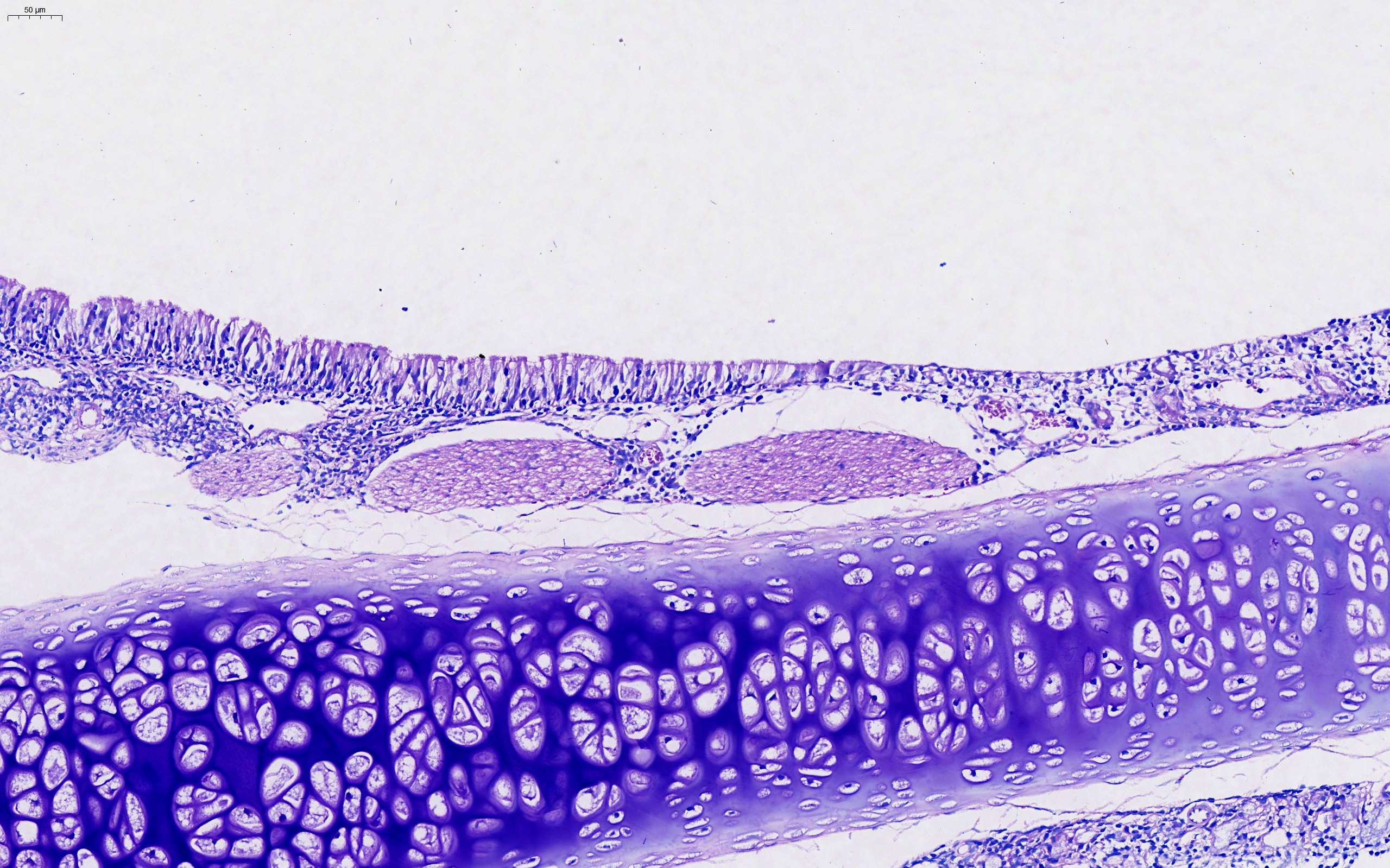

Supplement: Supplementary file 1 [file DataSheet3.ZIP › Microscopy images-Giemsa_200x_50um/CAVO-H/CAVO-H 5 Giemsa_200x_50um_1.jpeg]

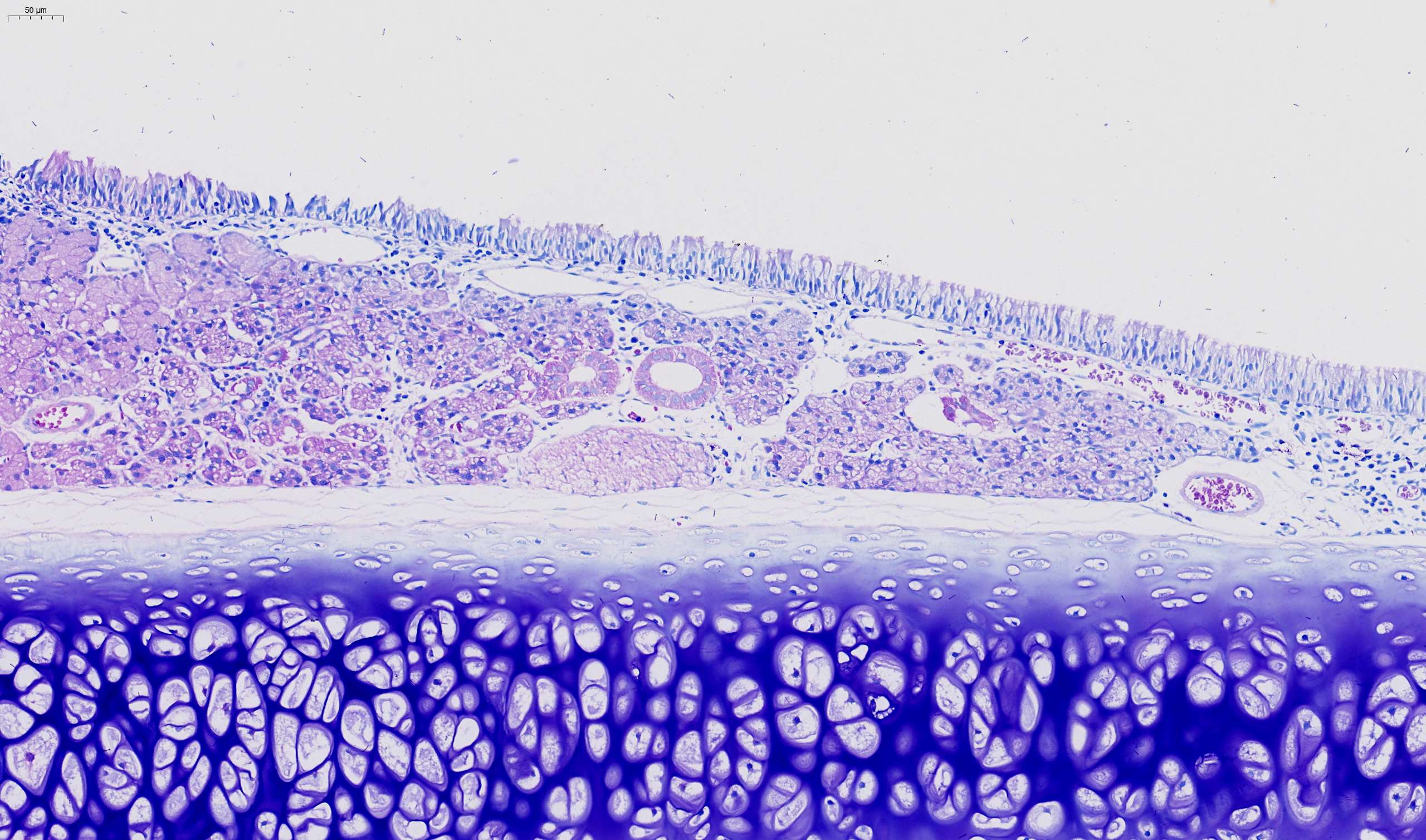

Supplement: Supplementary file 1 [file DataSheet3.ZIP › Microscopy images-Giemsa_200x_50um/CAVO-L/CAVO-L 1 Giemsa_200x_50um_1.jpeg]

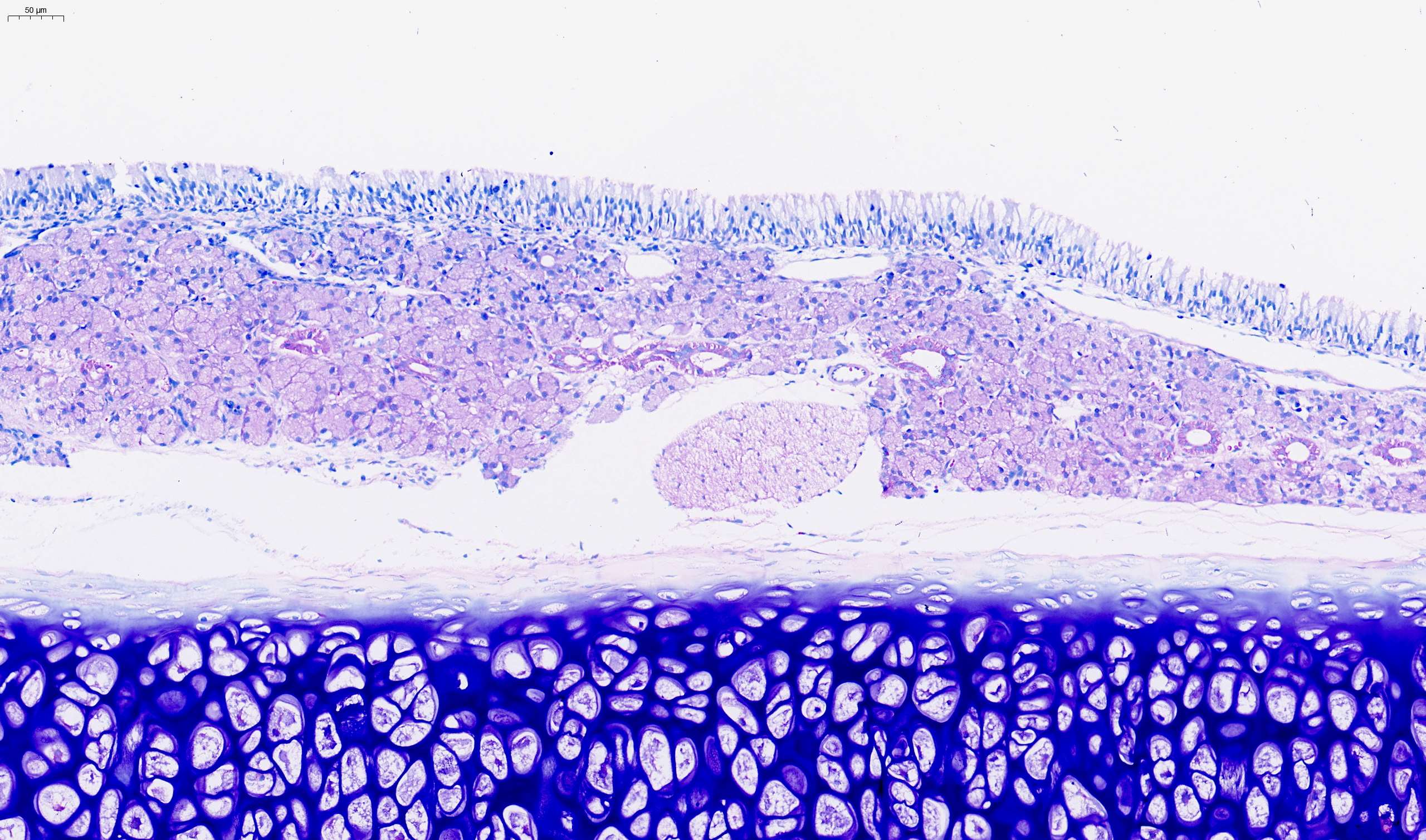

Supplement: Supplementary file 1 [file DataSheet3.ZIP › Microscopy images-Giemsa_200x_50um/CAVO-L/CAVO-L 2 Giemsa_200x_50um_1.jpeg]

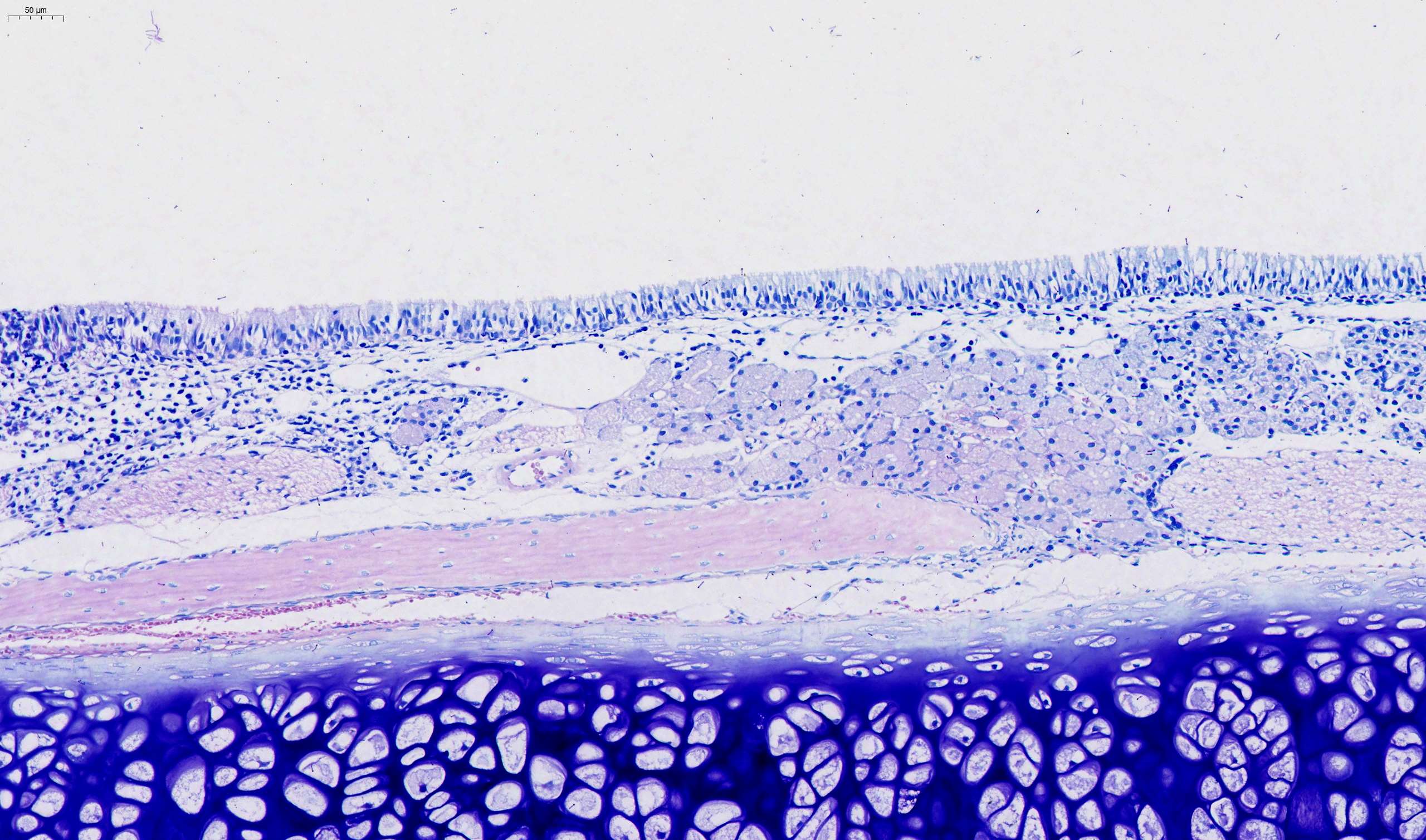

Supplement: Supplementary file 1 [file DataSheet3.ZIP › Microscopy images-Giemsa_200x_50um/CAVO-L/CAVO-L 3 Giemsa_200x_50um_1.jpeg]

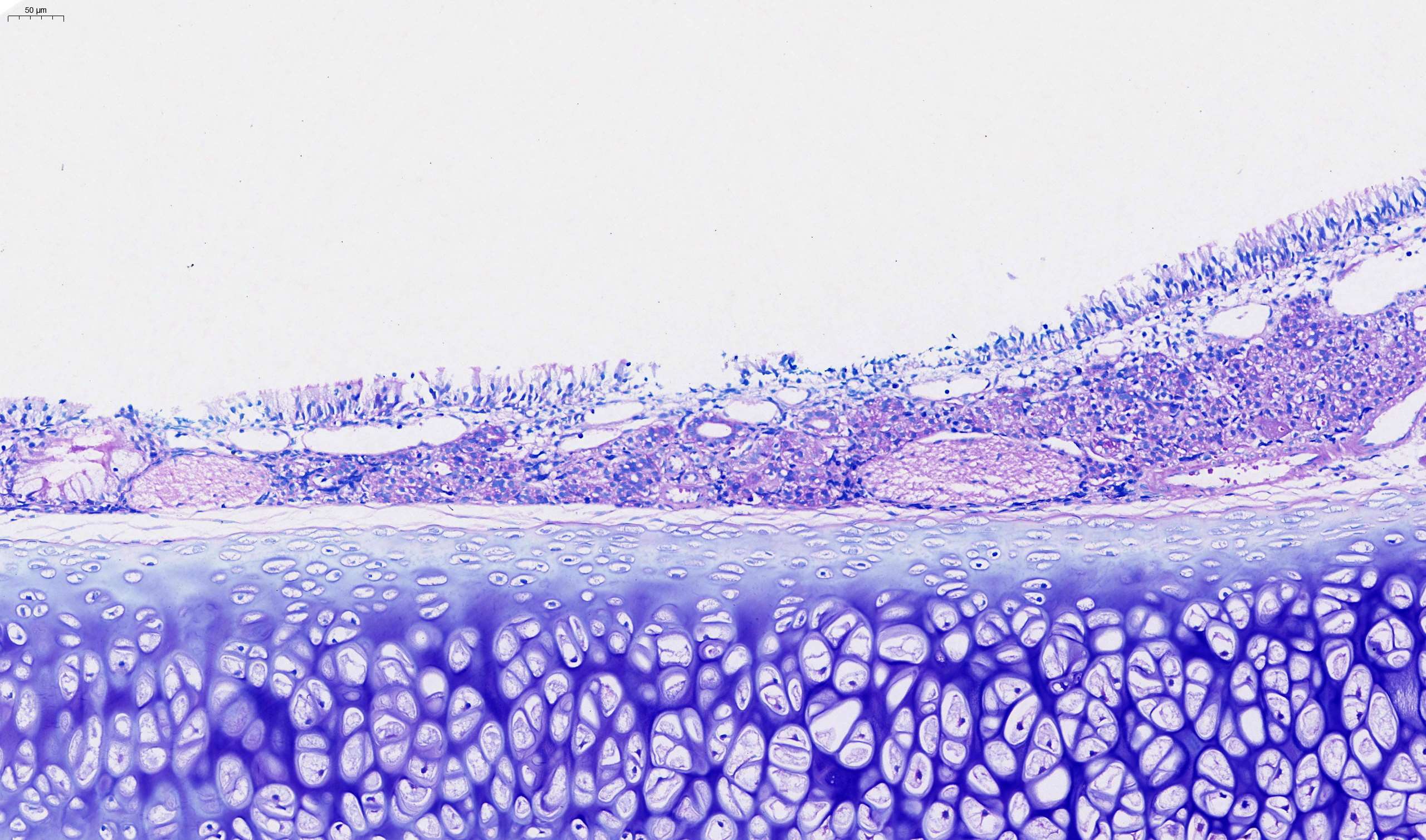

Supplement: Supplementary file 1 [file DataSheet3.ZIP › Microscopy images-Giemsa_200x_50um/CAVO-L/CAVO-L 4 Giemsa_200x_50um_1.jpeg]

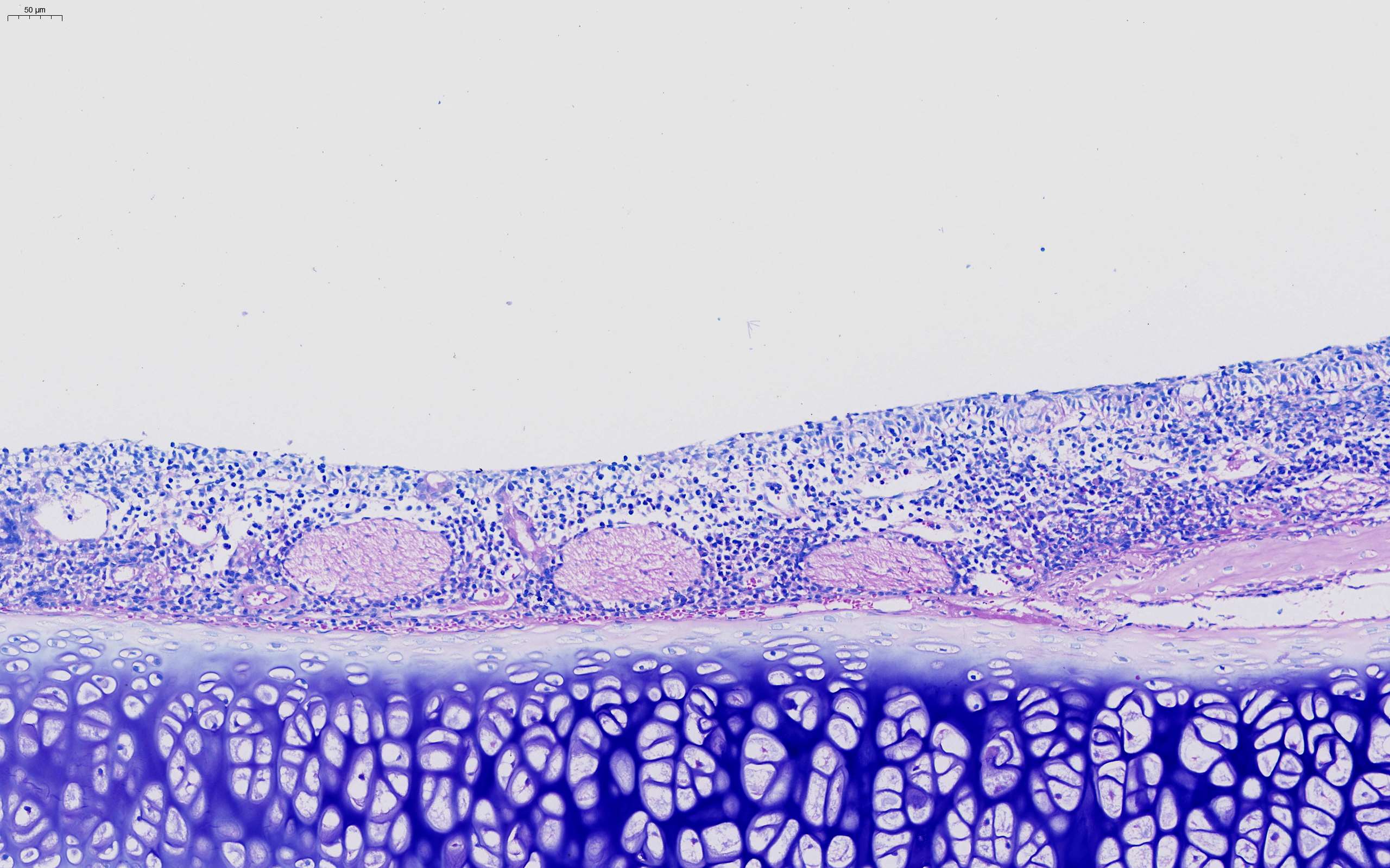

Supplement: Supplementary file 1 [file DataSheet3.ZIP › Microscopy images-Giemsa_200x_50um/CAVO-L/CAVO-L 5 Giemsa_200x_50um_1.jpeg]

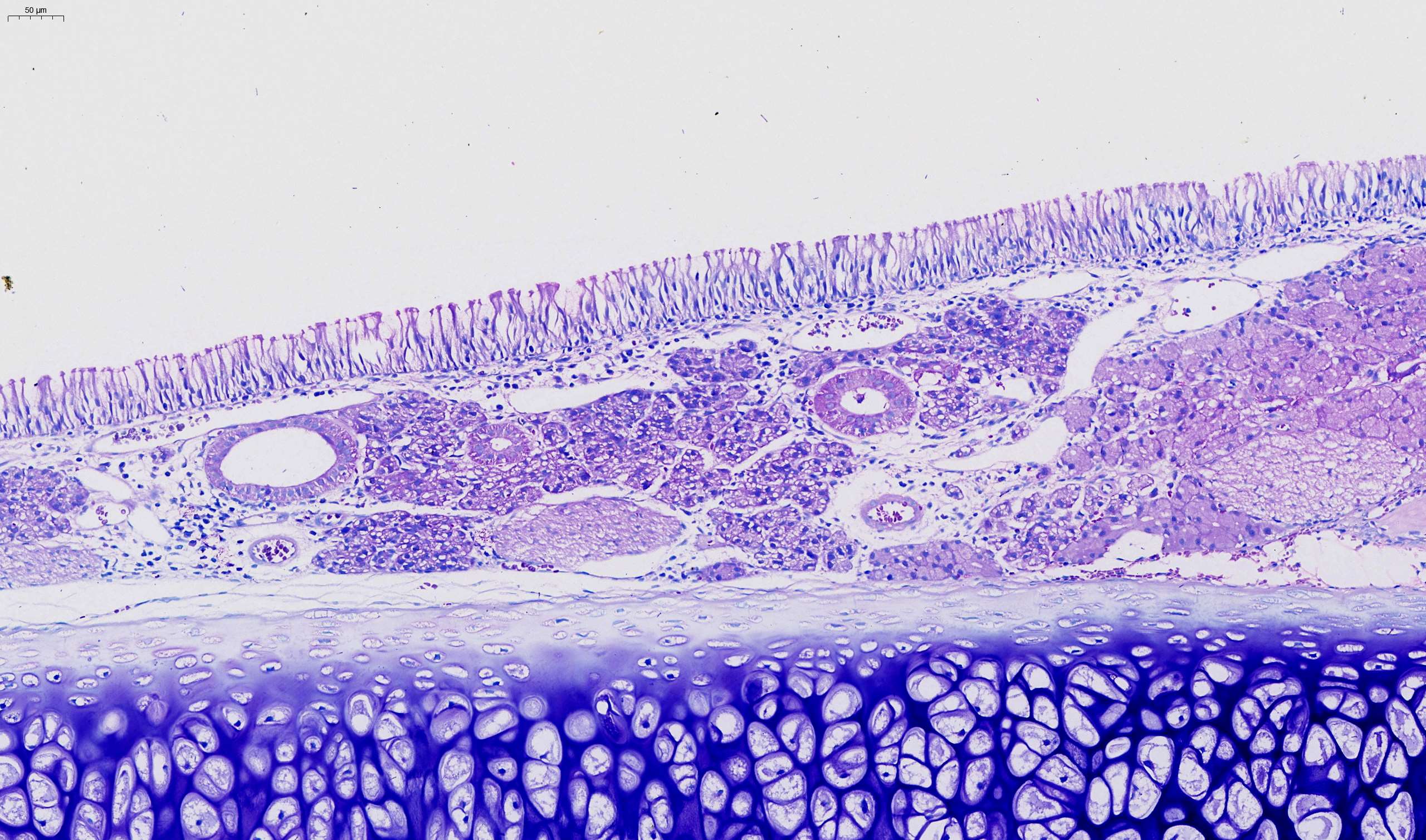

Supplement: Supplementary file 1 [file DataSheet3.ZIP › Microscopy images-Giemsa_200x_50um/CAVO-M/CAVO-M 1 Giemsa_200x_50um_1.jpeg]

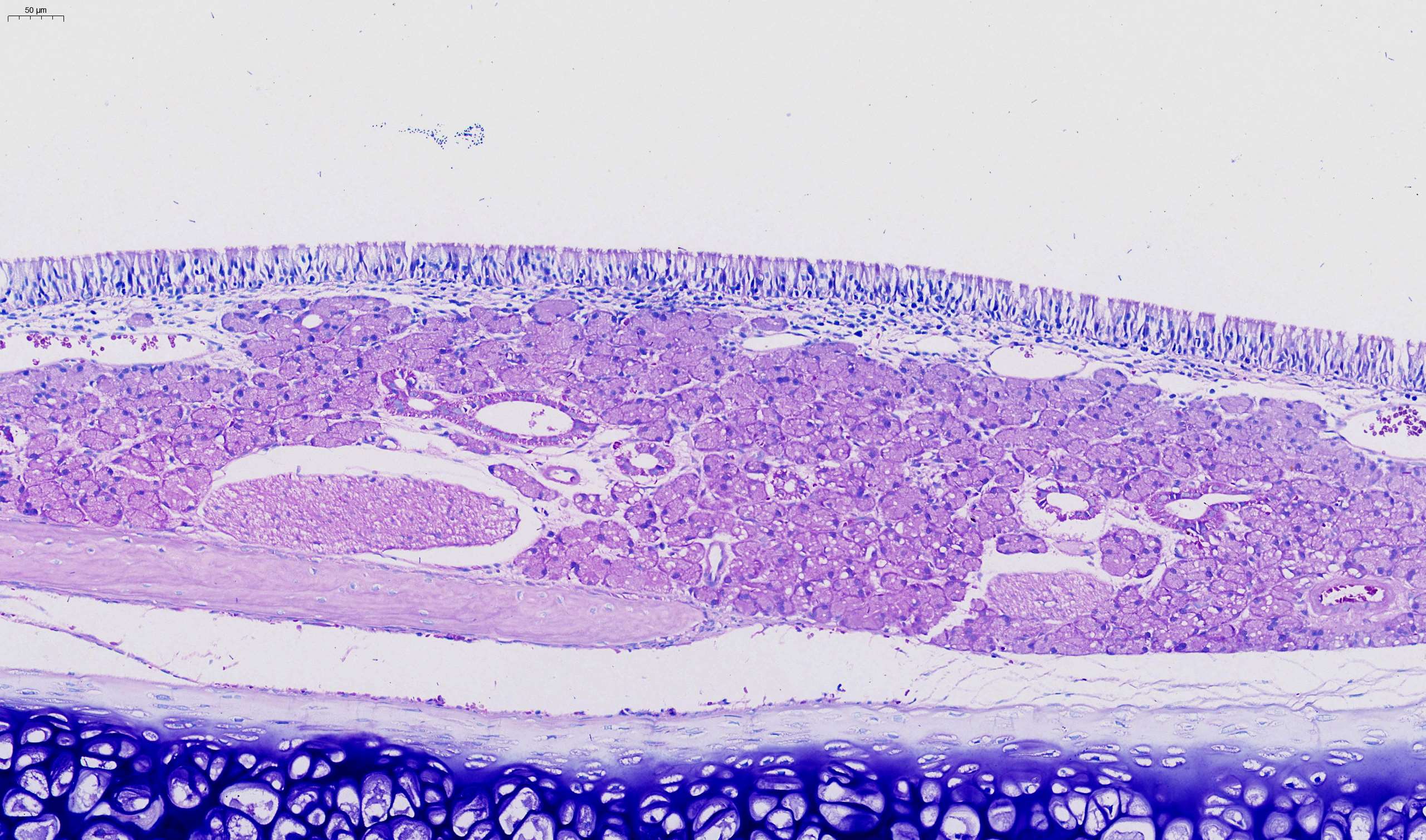

Supplement: Supplementary file 1 [file DataSheet3.ZIP › Microscopy images-Giemsa_200x_50um/CAVO-M/CAVO-M 2 Giemsa_200x_50um_1.jpeg]

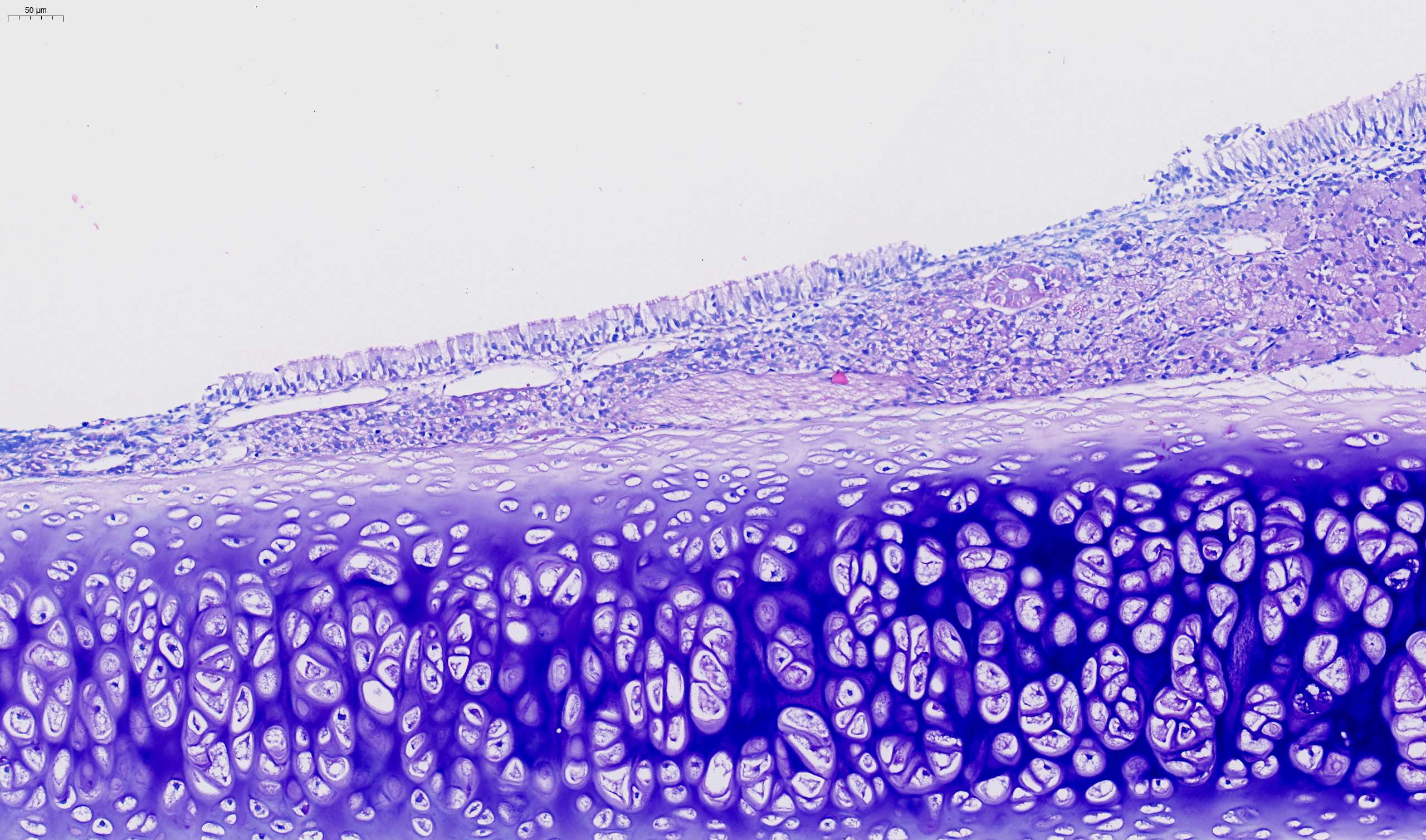

Supplement: Supplementary file 1 [file DataSheet3.ZIP › Microscopy images-Giemsa_200x_50um/CAVO-M/CAVO-M 3 Giemsa_200x_50um_1.jpeg]

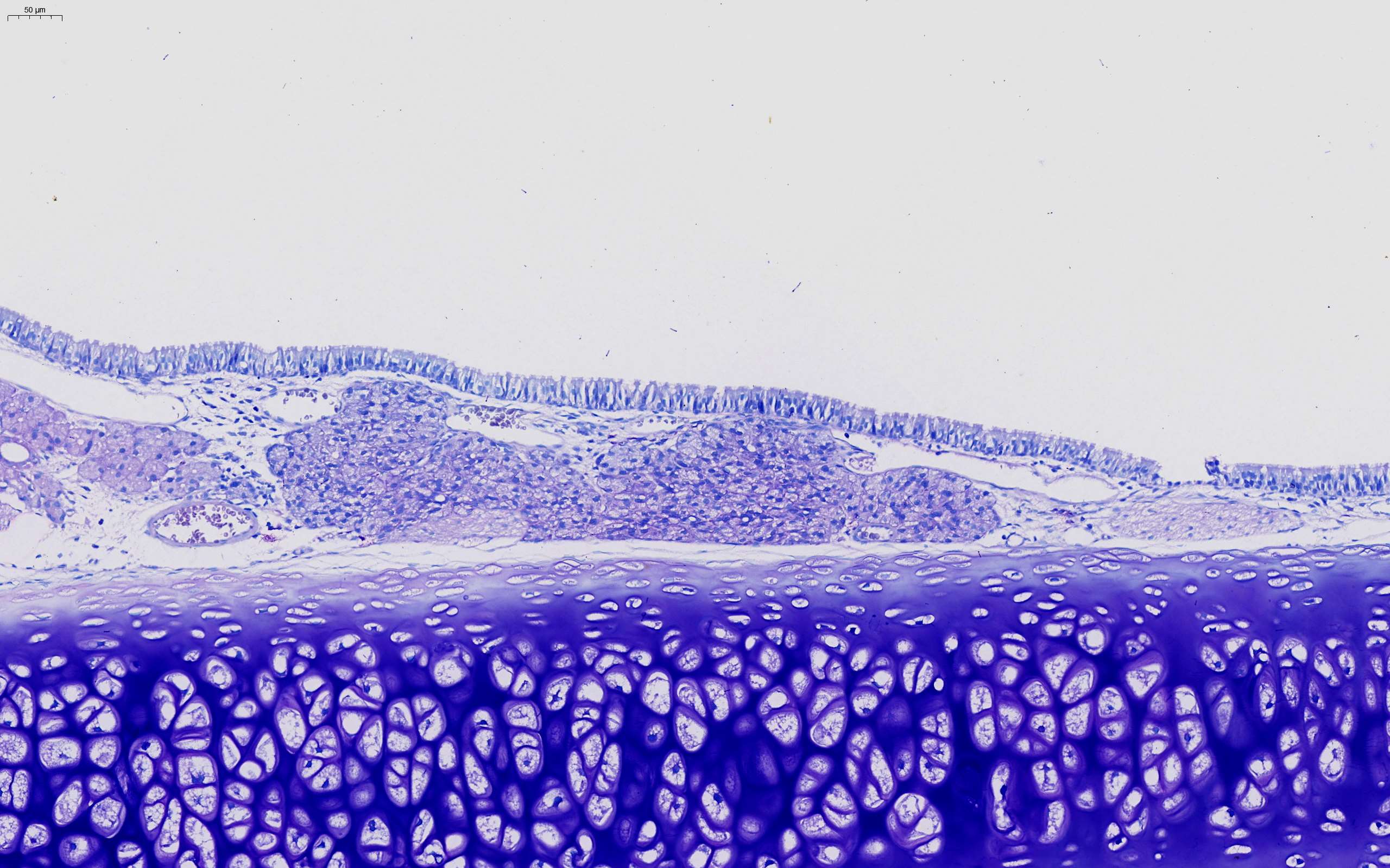

Supplement: Supplementary file 1 [file DataSheet3.ZIP › Microscopy images-Giemsa_200x_50um/CAVO-M/CAVO-M 4 Giemsa_200x_50um_1.jpeg]

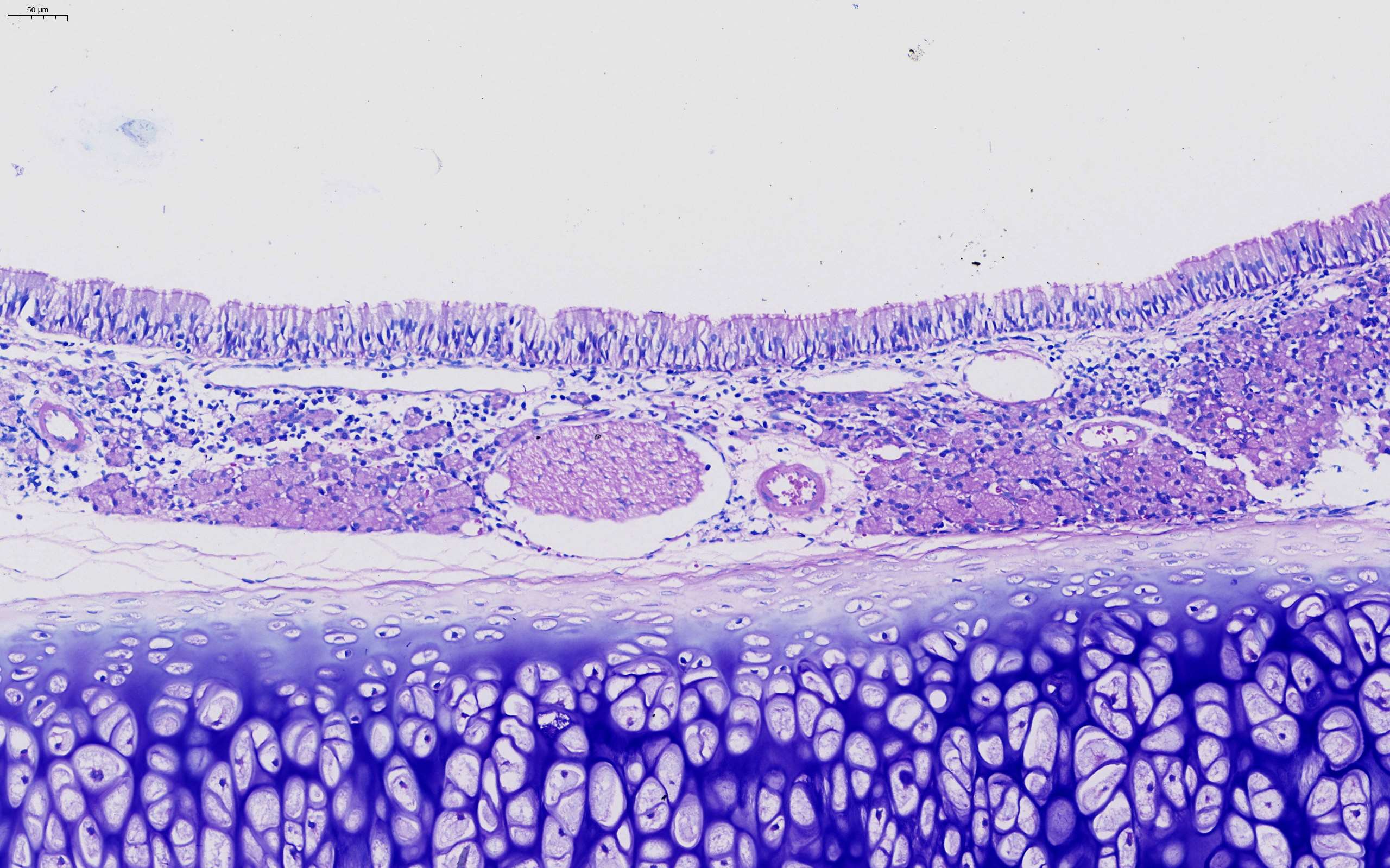

Supplement: Supplementary file 1 [file DataSheet3.ZIP › Microscopy images-Giemsa_200x_50um/CAVO-M/CAVO-M 5 Giemsa_200x_50um_1.jpeg]

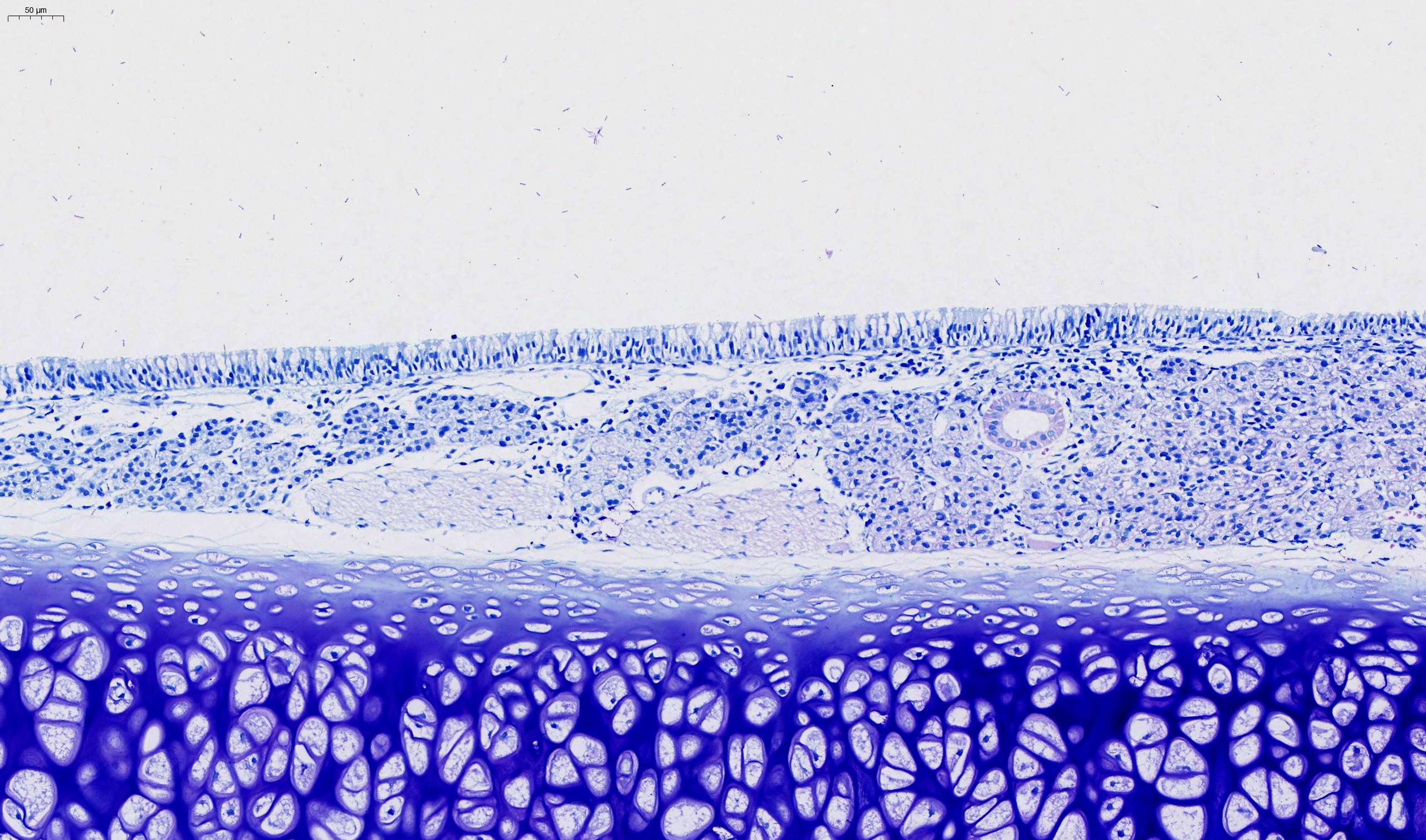

Supplement: Supplementary file 1 [file DataSheet3.ZIP › Microscopy images-Giemsa_200x_50um/Control/Control 1 Giemsa_200x_50um_1.jpeg]

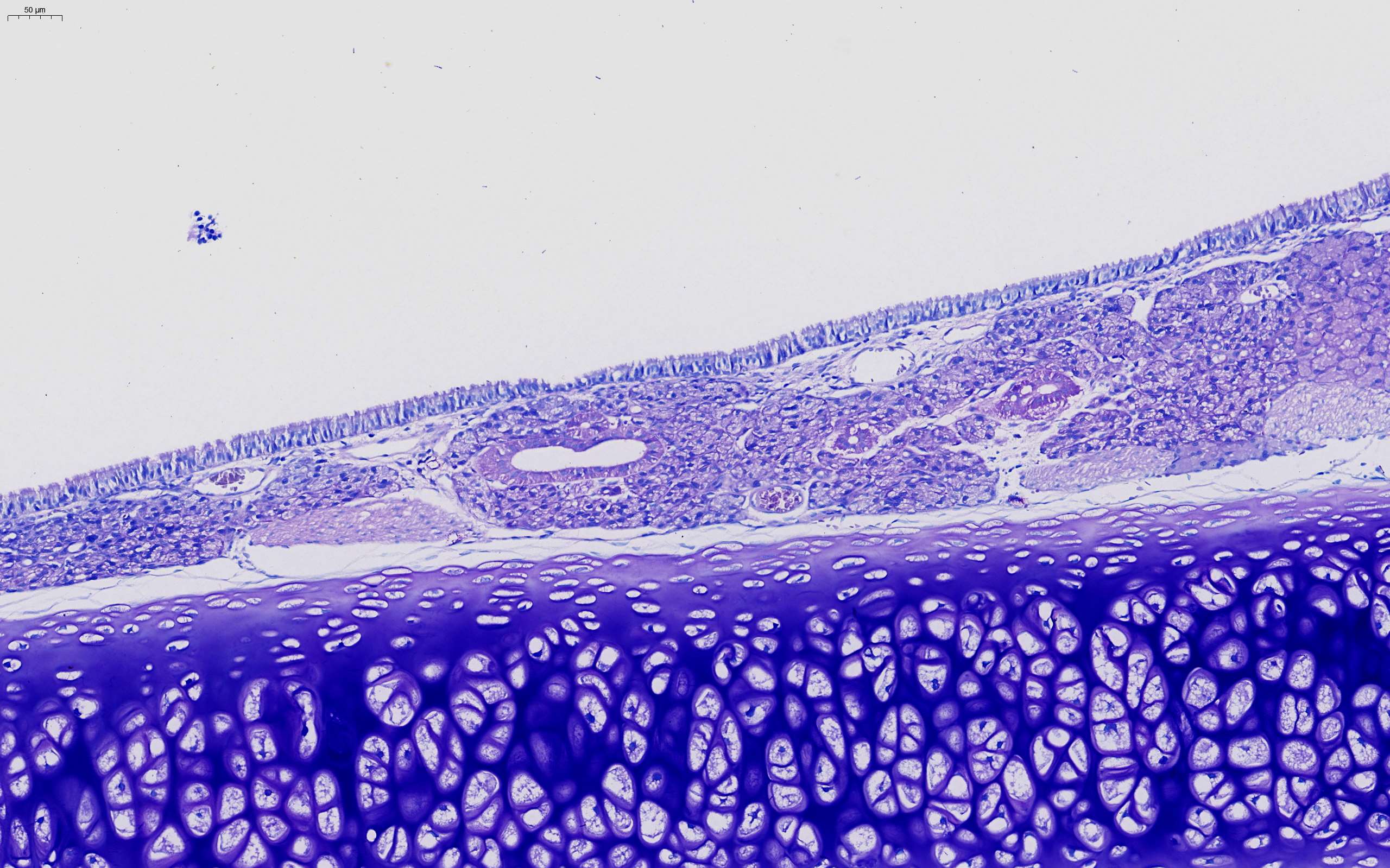

Supplement: Supplementary file 1 [file DataSheet3.ZIP › Microscopy images-Giemsa_200x_50um/Control/Control 2 Giemsa_200x_50um_1.jpeg]

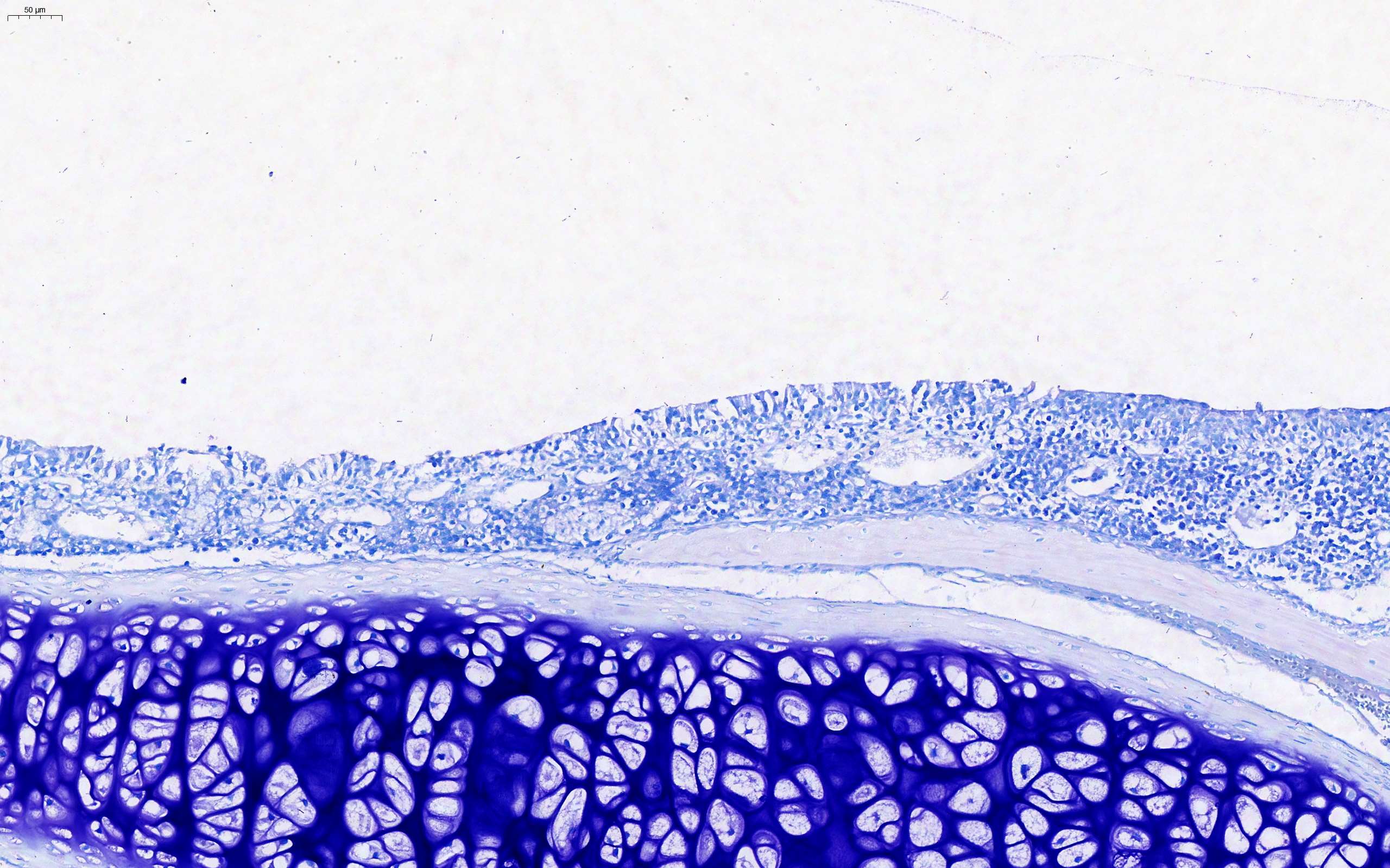

Supplement: Supplementary file 1 [file DataSheet3.ZIP › Microscopy images-Giemsa_200x_50um/Control/Control 3 Giemsa_200x_50um_1.jpeg]

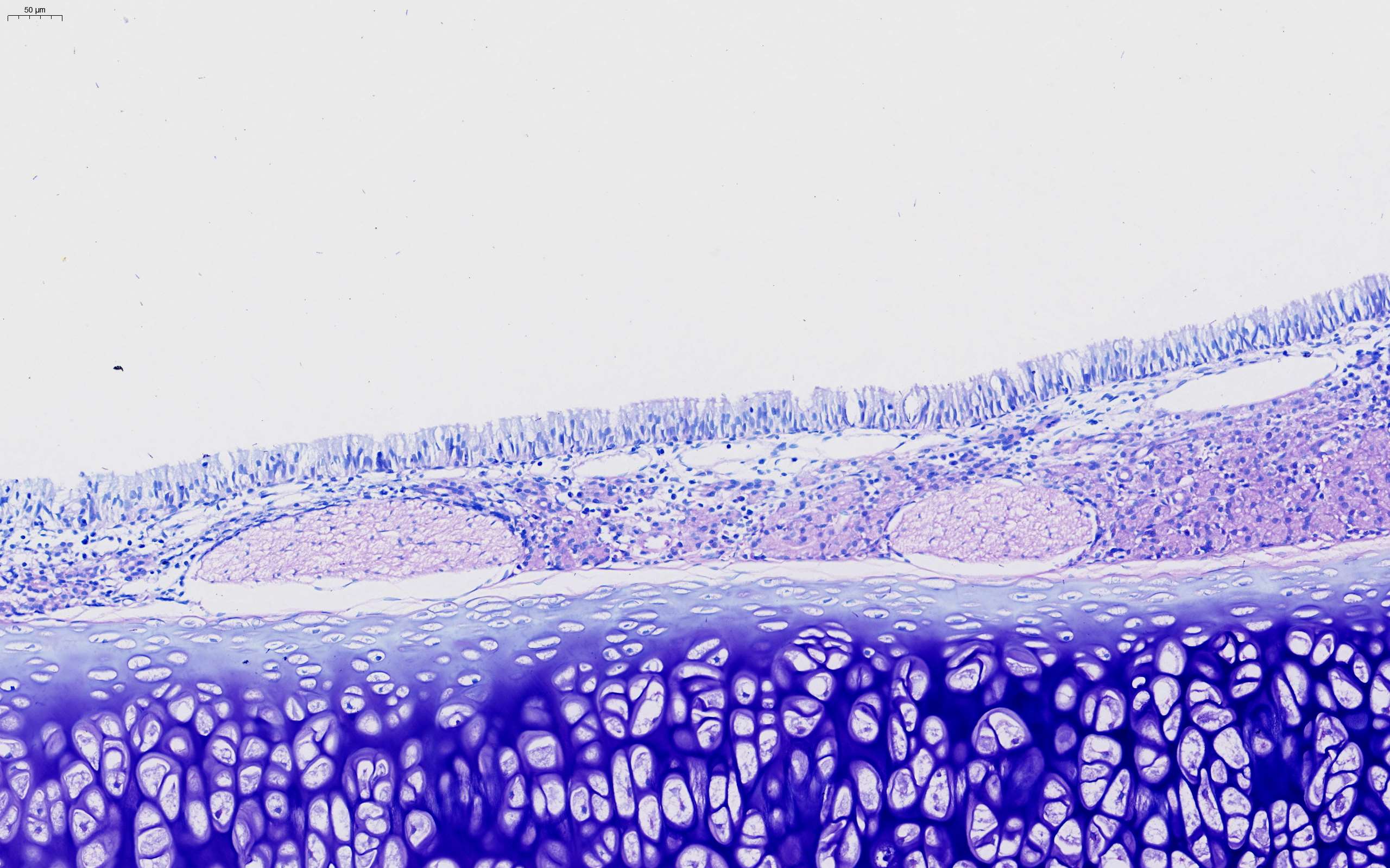

Supplement: Supplementary file 1 [file DataSheet3.ZIP › Microscopy images-Giemsa_200x_50um/Control/Control 4 Giemsa_200x_50um_1.jpeg]

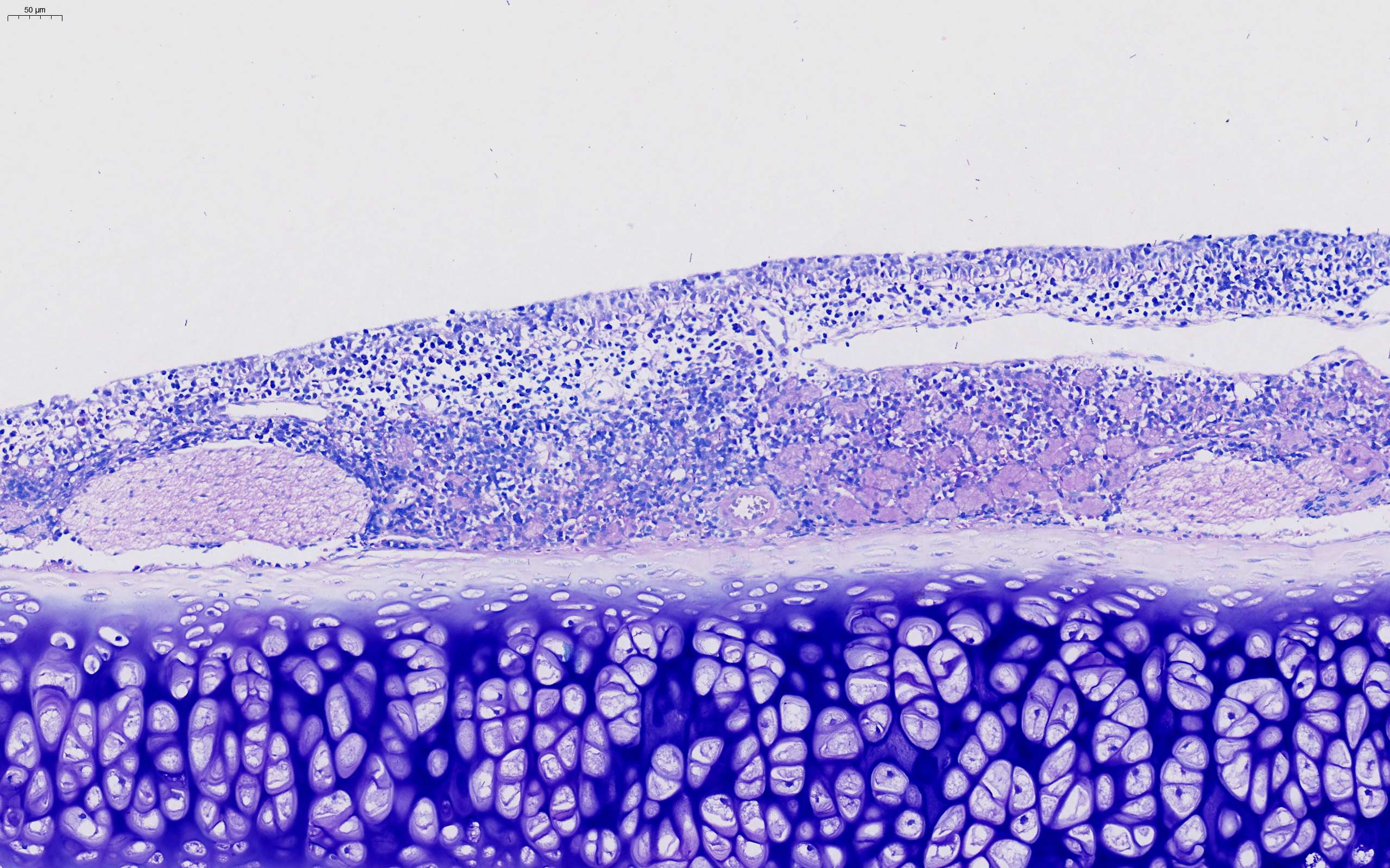

Supplement: Supplementary file 1 [file DataSheet3.ZIP › Microscopy images-Giemsa_200x_50um/Control/Control 5 Giemsa_200x_50um_1.jpeg]

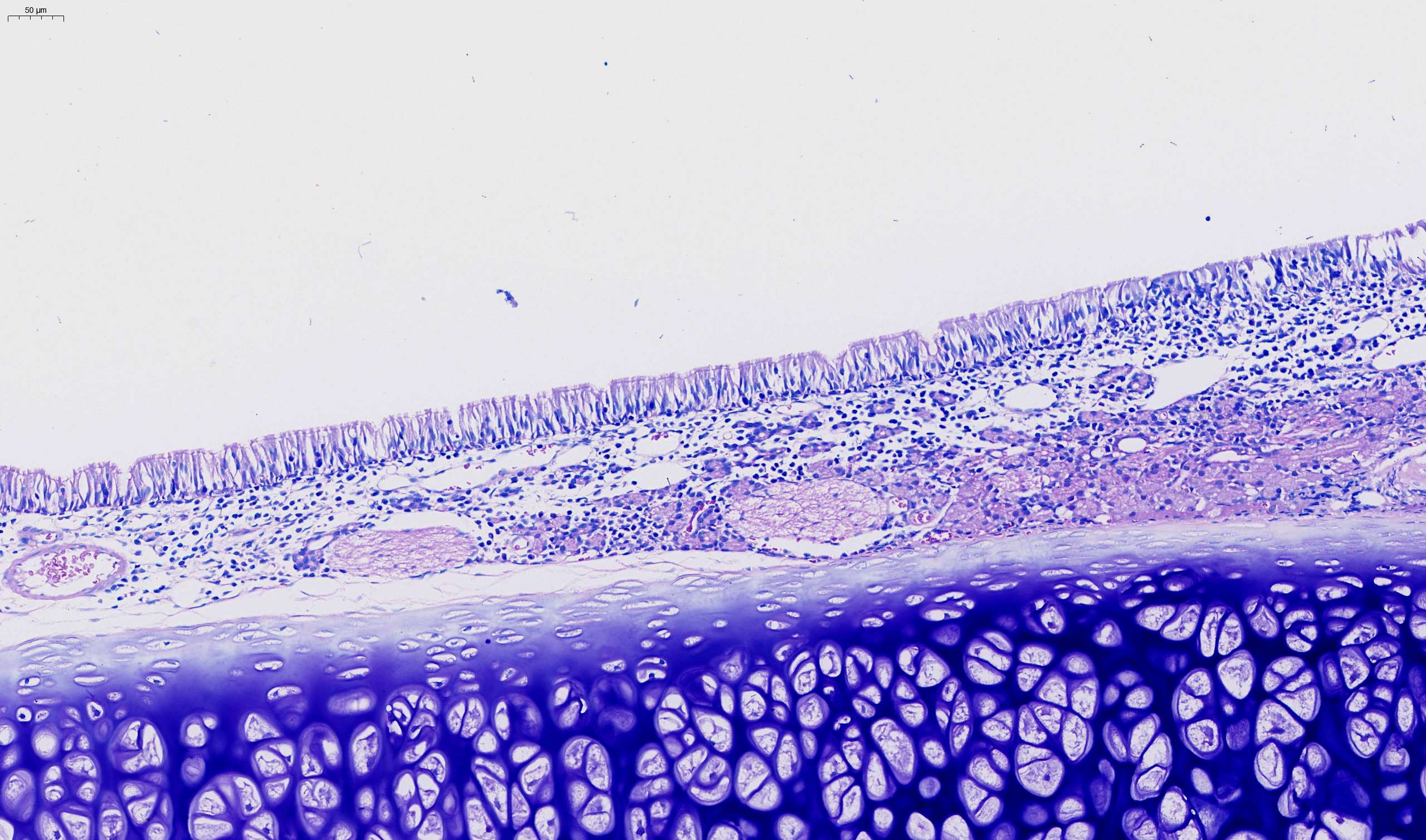

Supplement: Supplementary file 1 [file DataSheet3.ZIP › Microscopy images-Giemsa_200x_50um/Loratadine/Loratadine 1 Giemsa_200x_50um_1.jpeg]

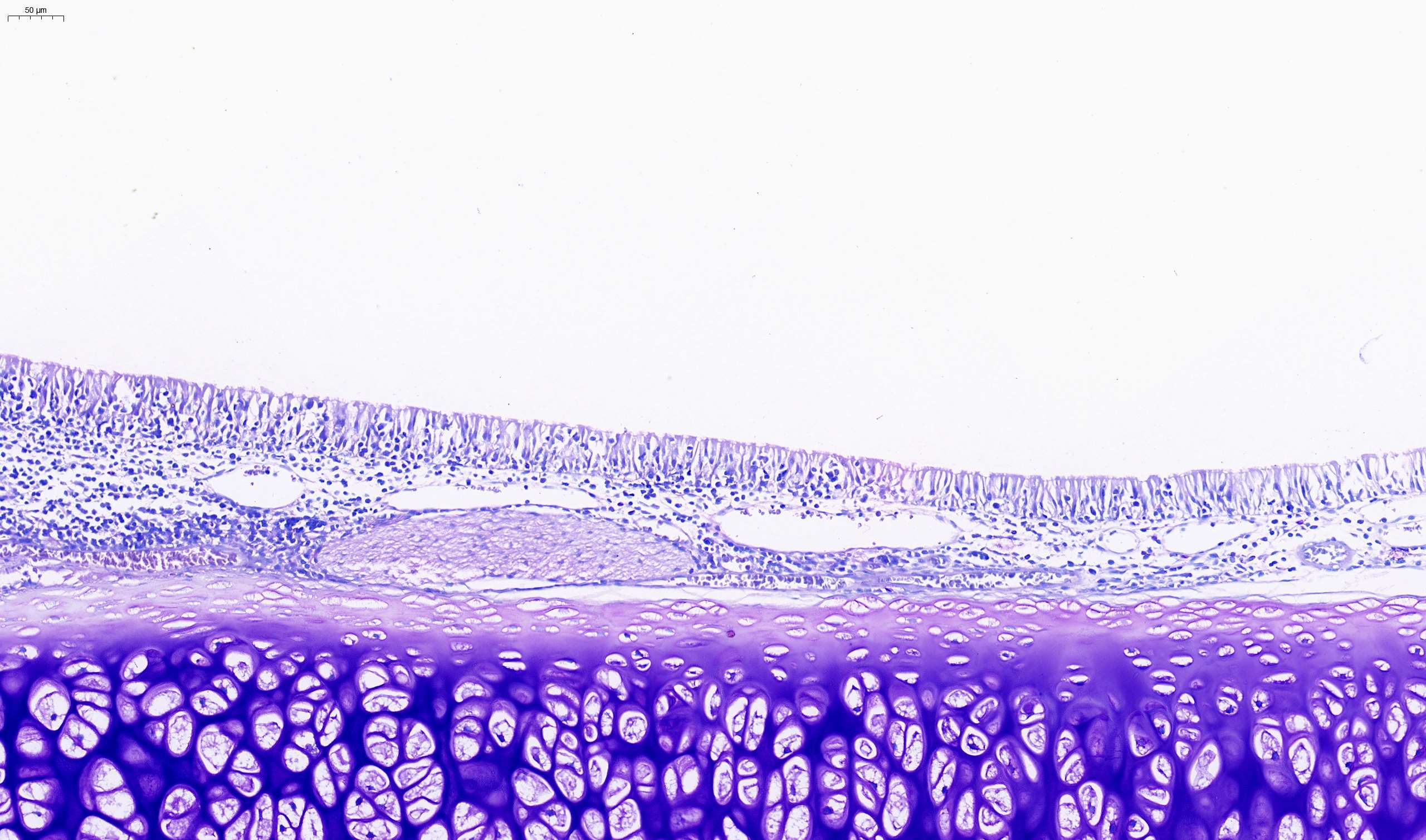

Supplement: Supplementary file 1 [file DataSheet3.ZIP › Microscopy images-Giemsa_200x_50um/Loratadine/Loratadine 2 Giemsa_200x_50um_1.jpeg]

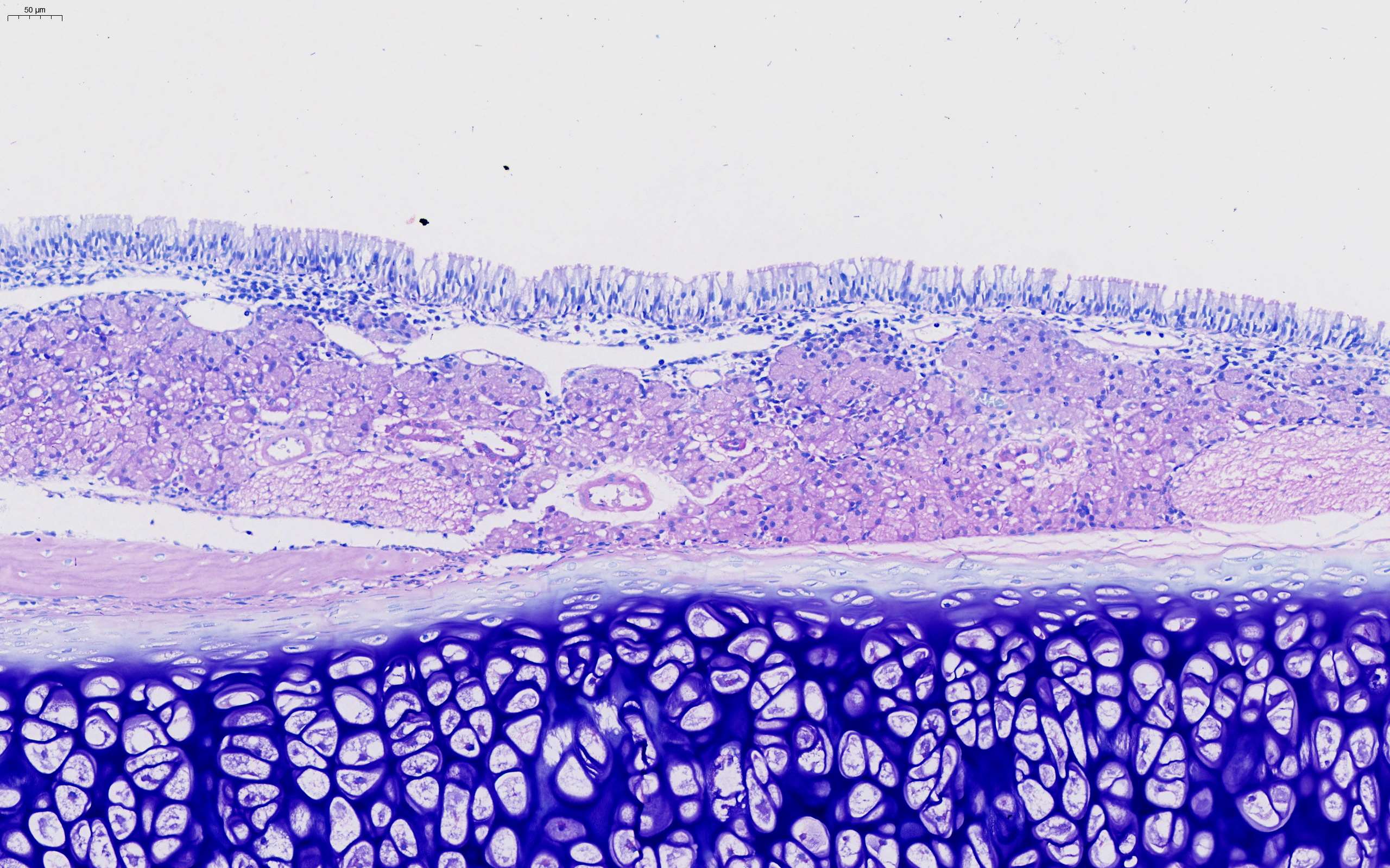

Supplement: Supplementary file 1 [file DataSheet3.ZIP › Microscopy images-Giemsa_200x_50um/Loratadine/Loratadine 4 Giemsa_200x_50um_1.jpeg]

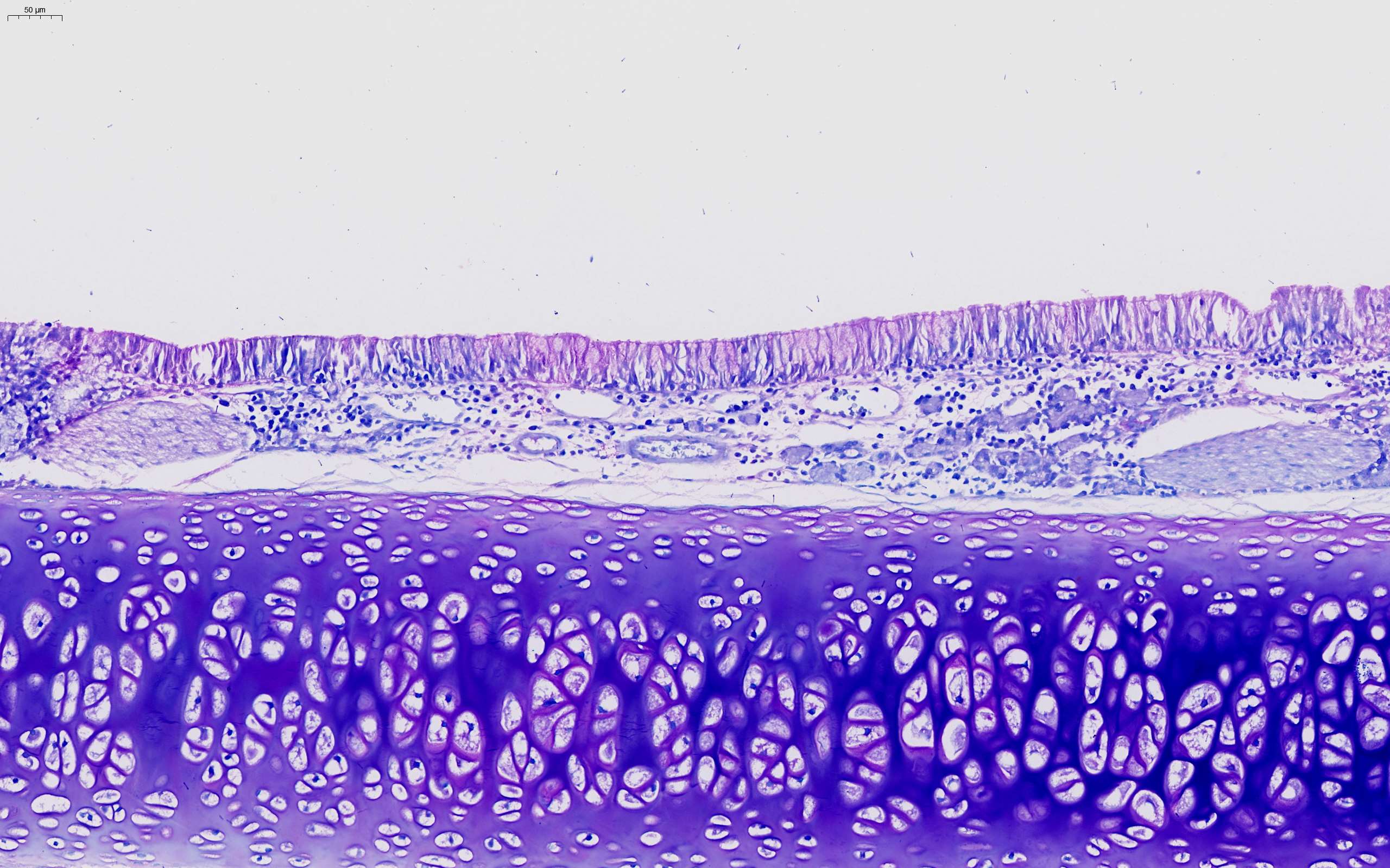

Supplement: Supplementary file 1 [file DataSheet3.ZIP › Microscopy images-Giemsa_200x_50um/Loratadine/Loratadine3 Giemsa_200x_50um_1.jpeg]

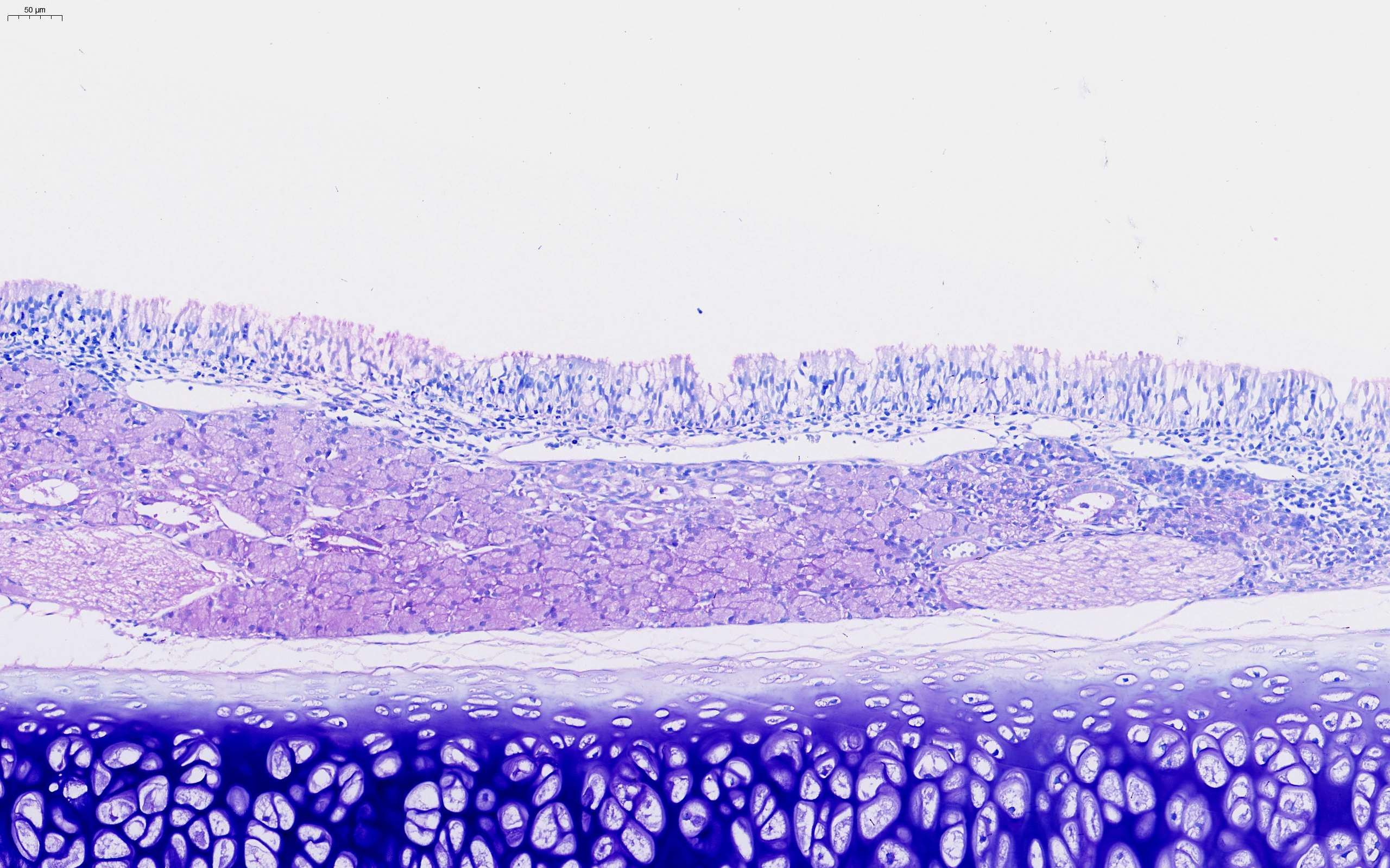

Supplement: Supplementary file 1 [file DataSheet3.ZIP › Microscopy images-Giemsa_200x_50um/Loratadine/Loratadine5 Giemsa_200x_50um_1.jpeg]

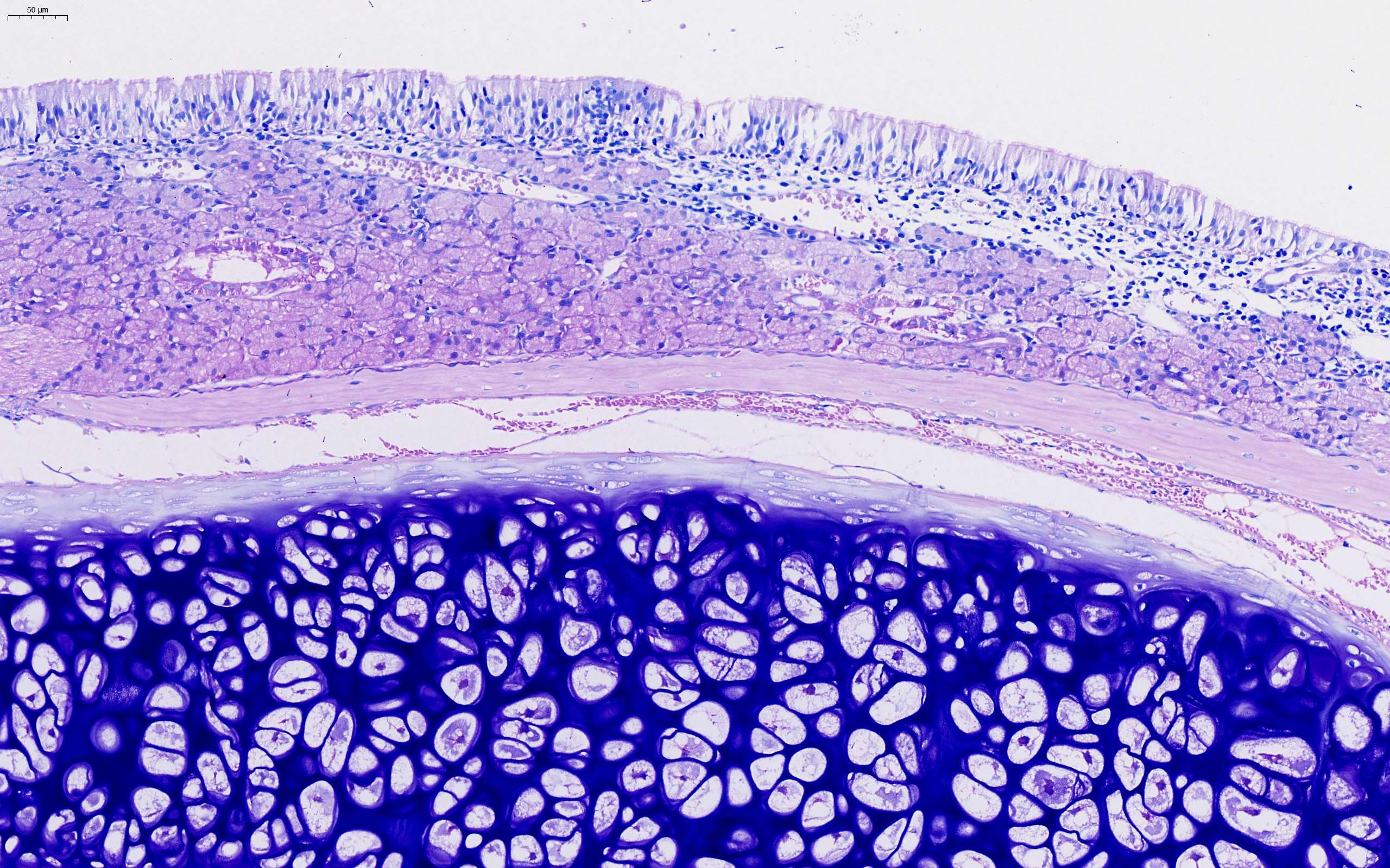

Supplement: Supplementary file 1 [file DataSheet3.ZIP › Microscopy images-Giemsa_200x_50um/Model/Model 1 Giemsa_200x_50um_1.jpeg]

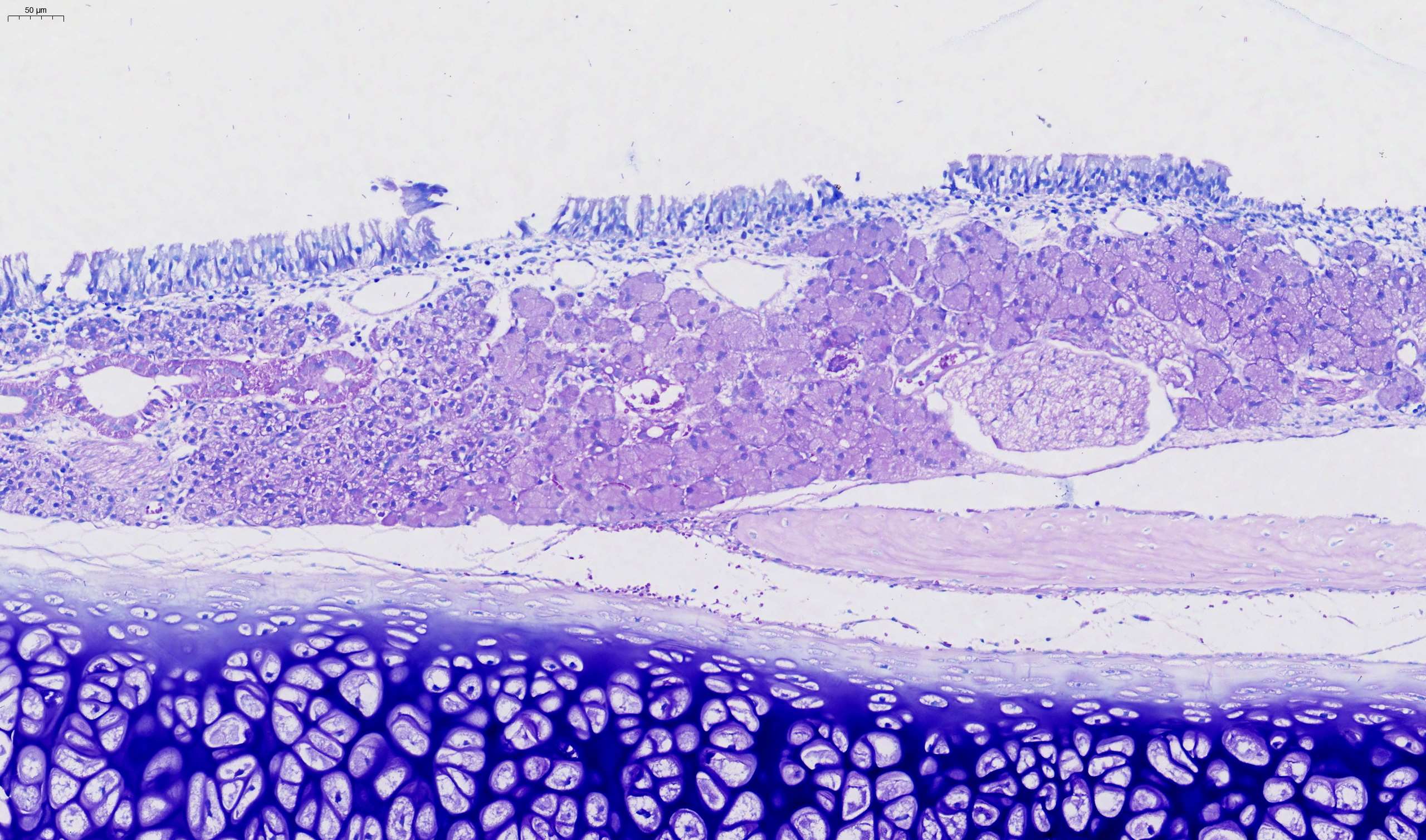

Supplement: Supplementary file 1 [file DataSheet3.ZIP › Microscopy images-Giemsa_200x_50um/Model/Model 2 Giemsa_200x_50um_1.jpeg]

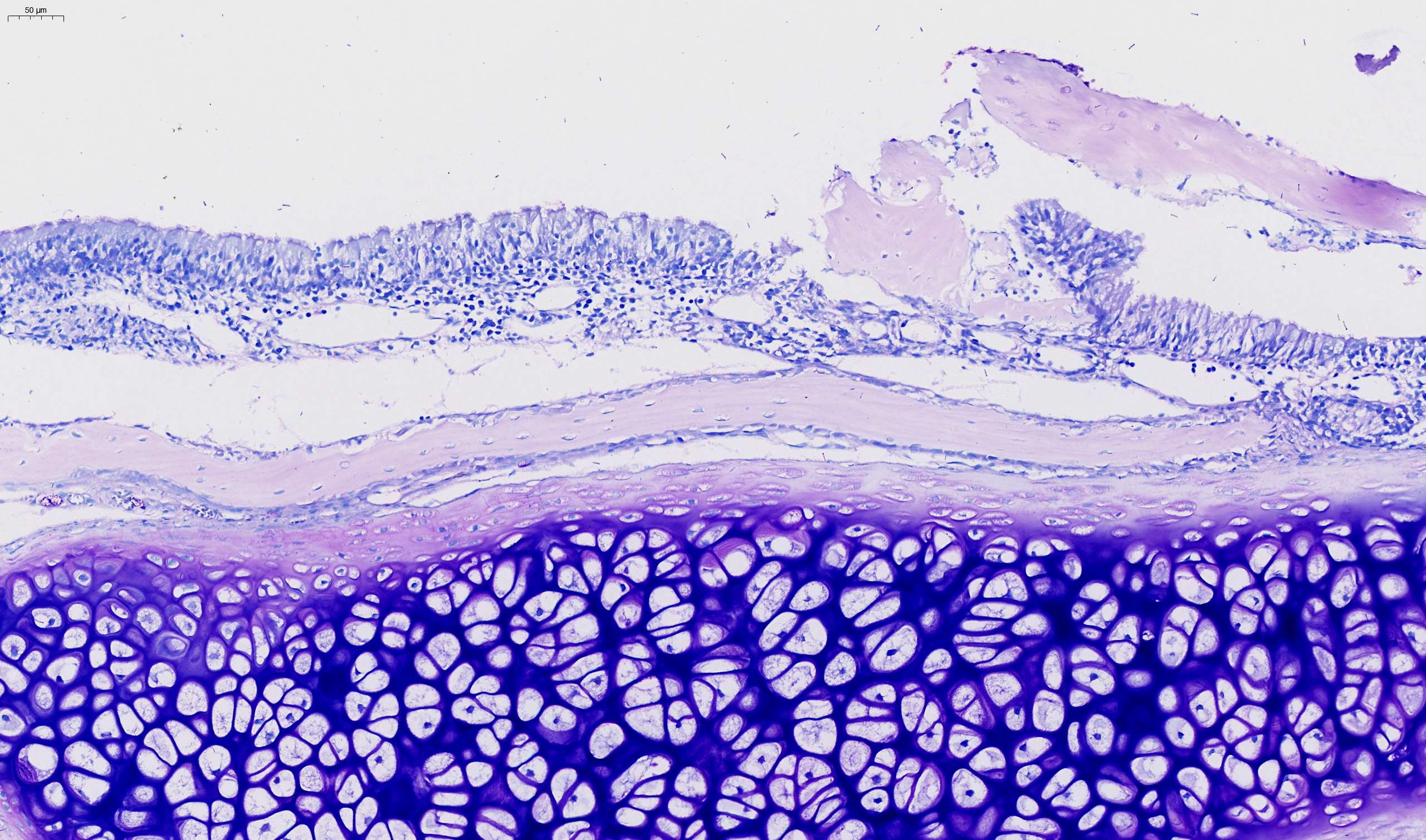

Supplement: Supplementary file 1 [file DataSheet3.ZIP › Microscopy images-Giemsa_200x_50um/Model/Model 3 Giemsa_200x_50um_1.jpeg]

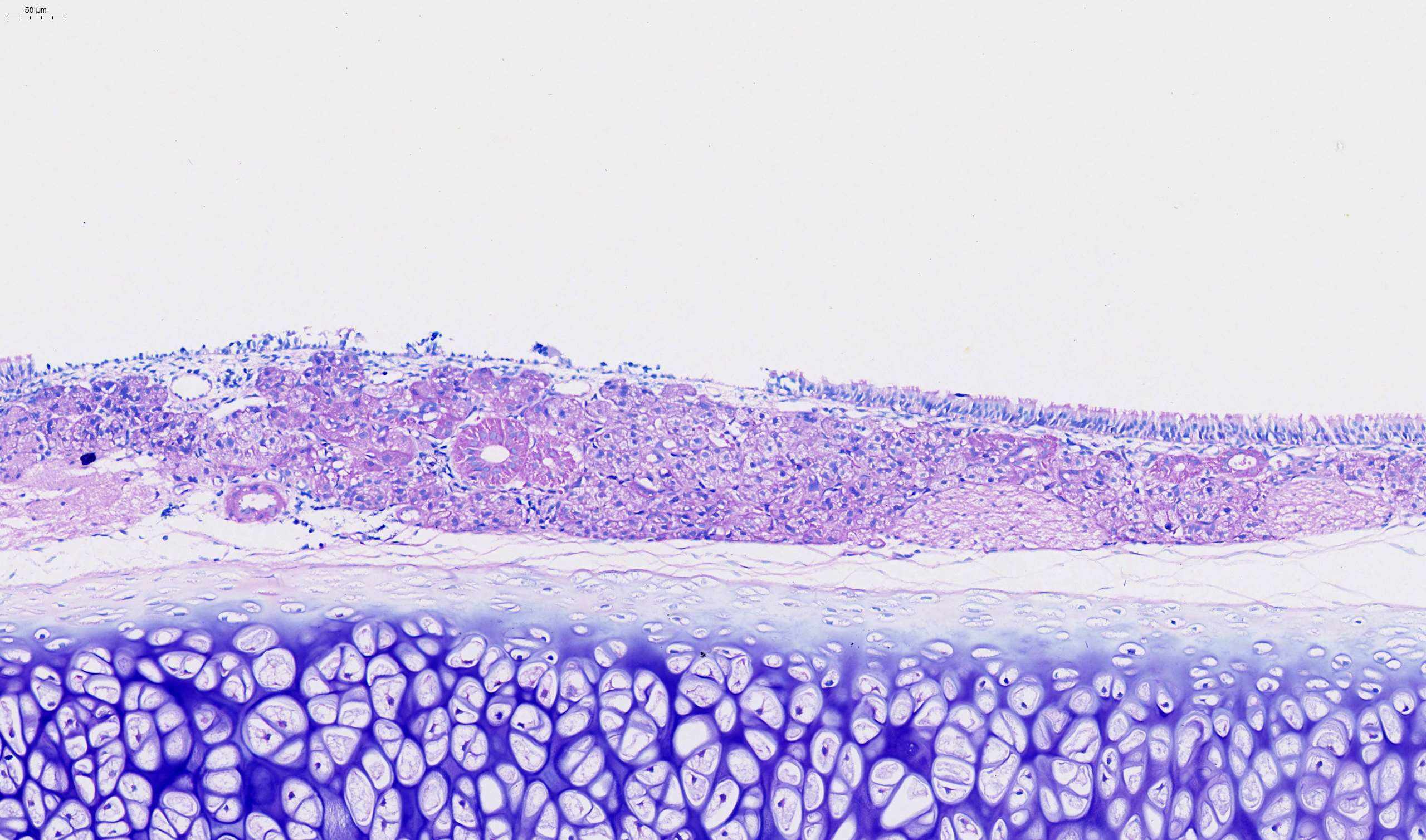

Supplement: Supplementary file 1 [file DataSheet3.ZIP › Microscopy images-Giemsa_200x_50um/Model/Model 4 Giemsa_200x_50um_1.jpeg]

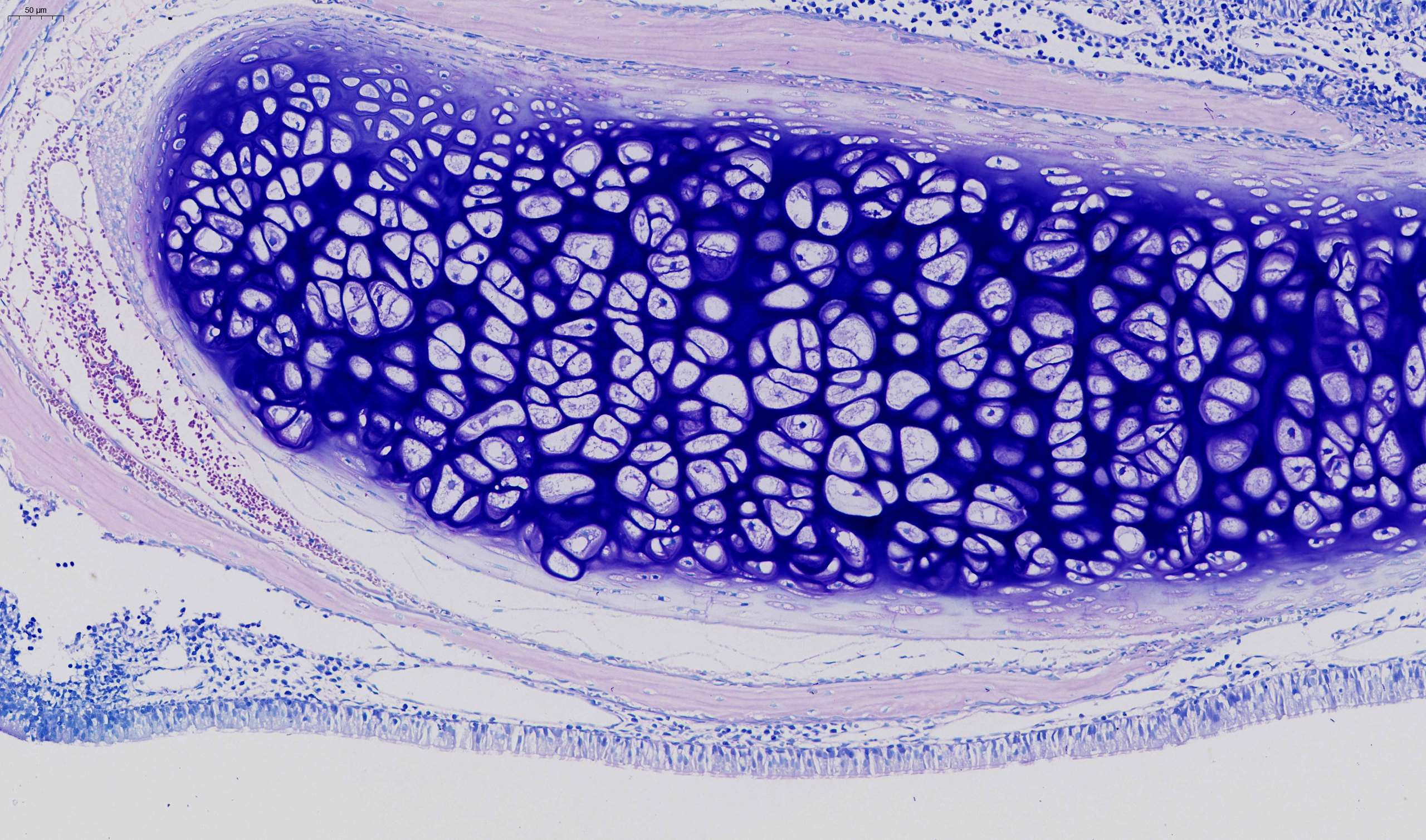

Supplement: Supplementary file 1 [file DataSheet3.ZIP › Microscopy images-Giemsa_200x_50um/Model/Model 5 Giemsa_200x_50um_1.jpeg]

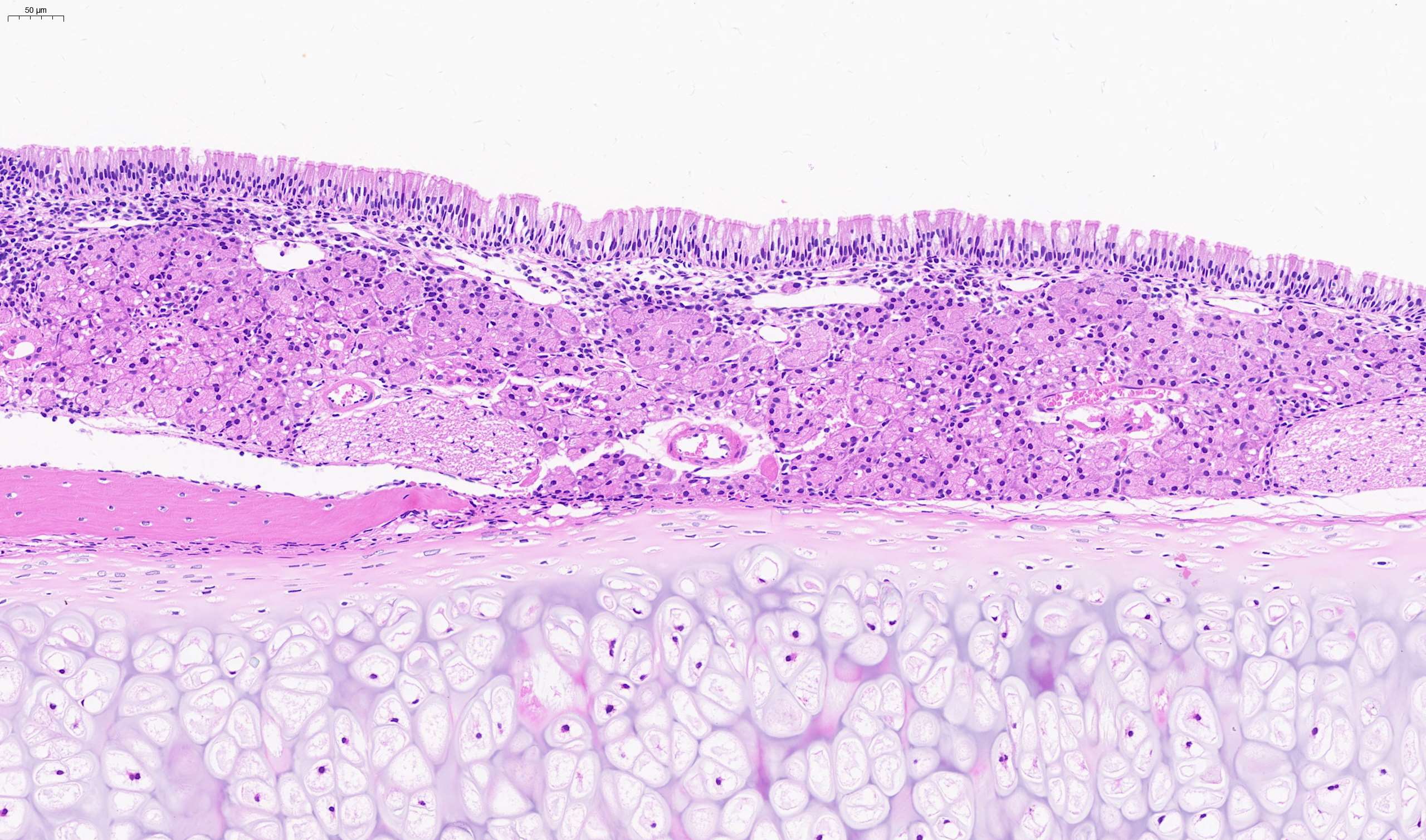

Supplement: Supplementary file 2 [file DataSheet4.ZIP › Microscopy images-H&E_200x_50um/CAVO-H/CAVO-H1 H&E_200x_50um_1.jpeg]

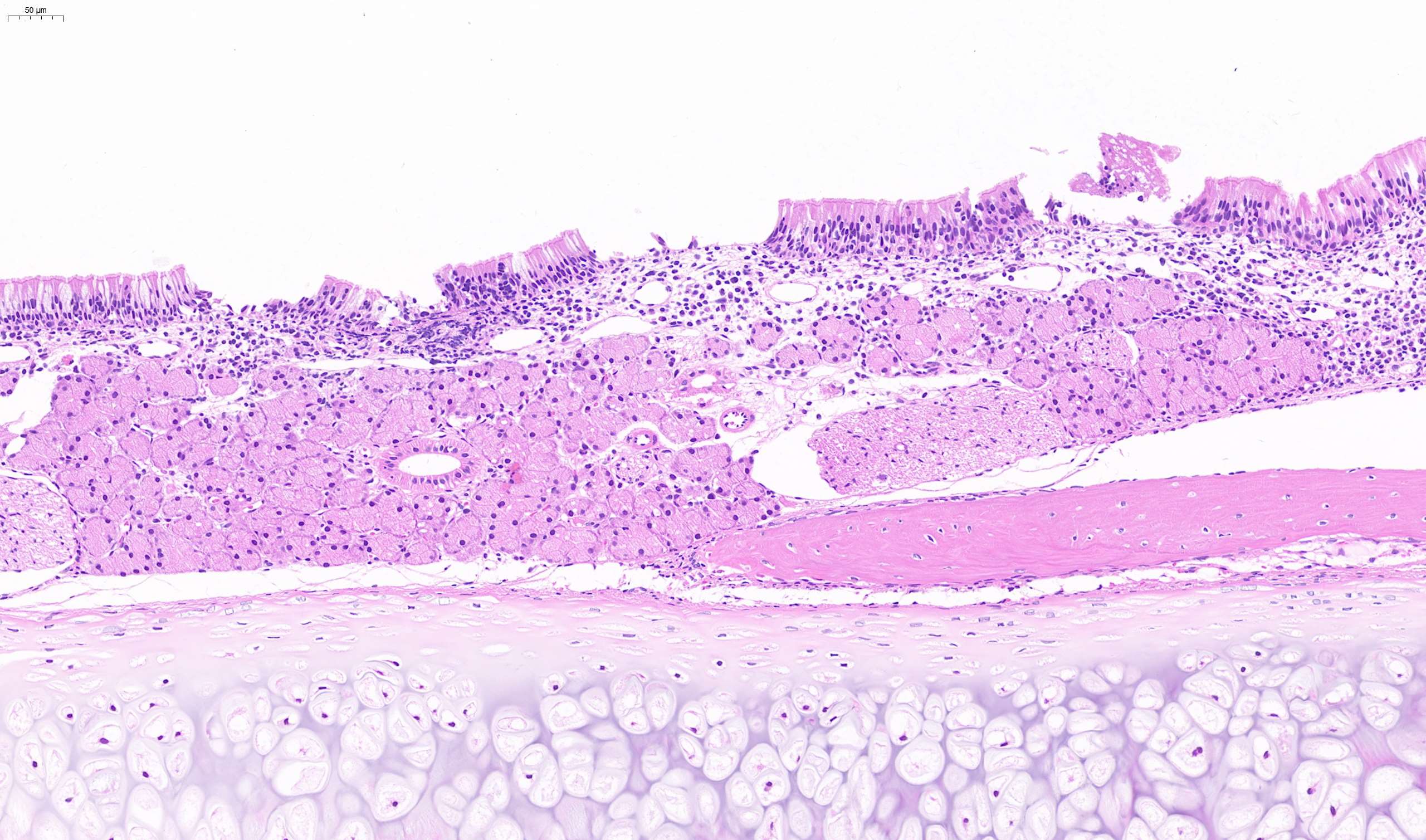

Supplement: Supplementary file 2 [file DataSheet4.ZIP › Microscopy images-H&E_200x_50um/CAVO-H/CAVO-H2 H&E_200x_50um_1.jpeg]

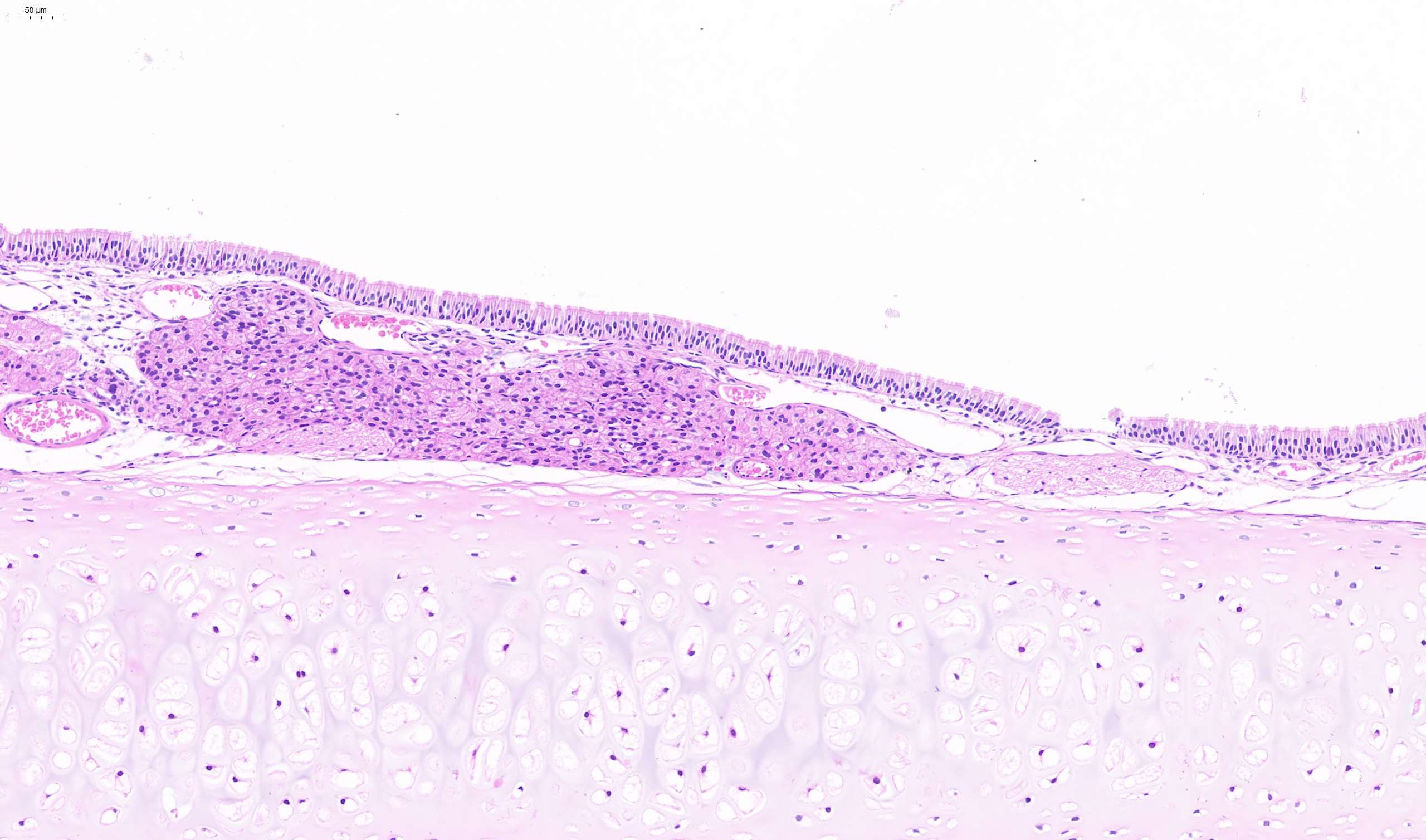

Supplement: Supplementary file 2 [file DataSheet4.ZIP › Microscopy images-H&E_200x_50um/CAVO-H/CAVO-H3 H&E_200x_50um_1.jpeg]

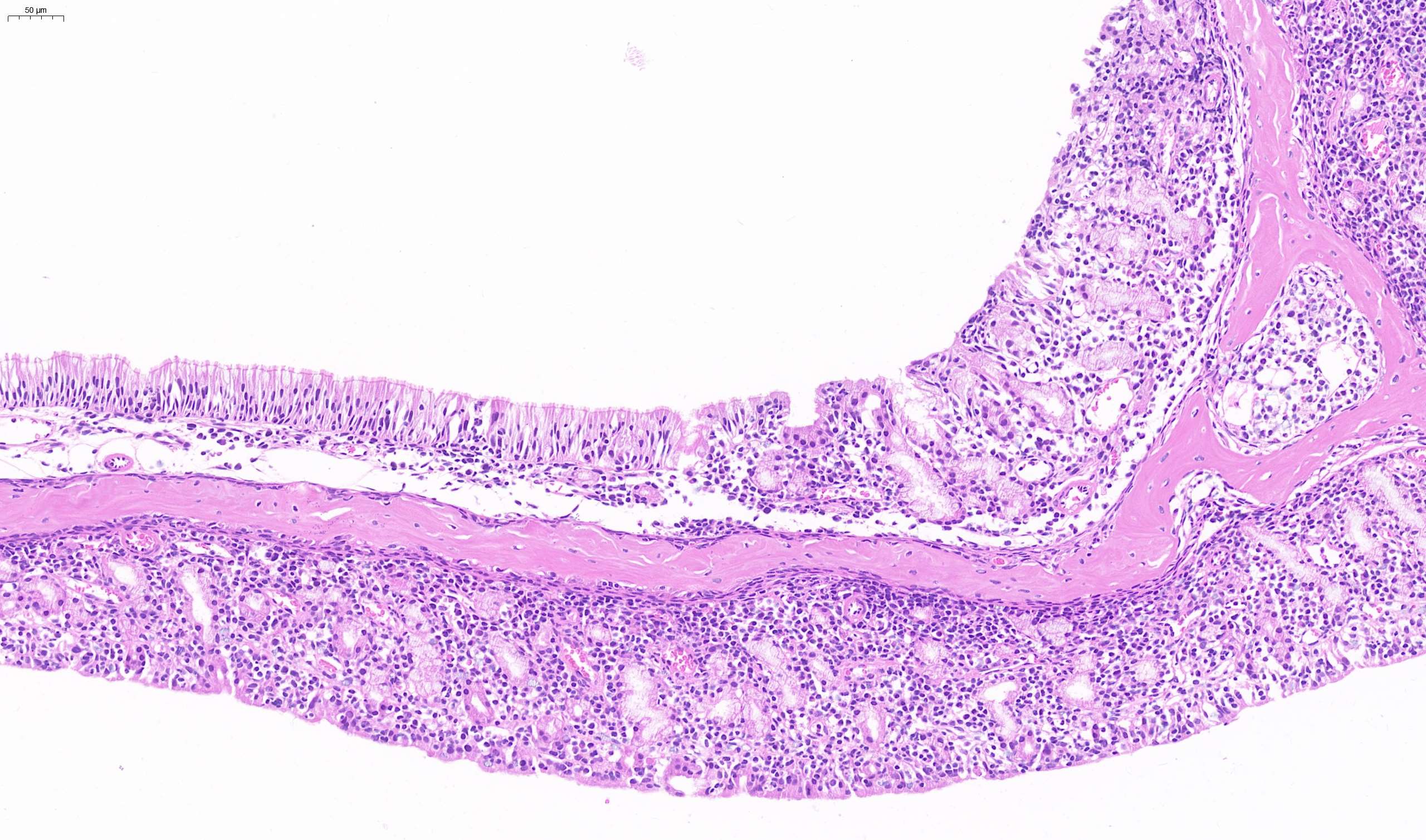

Supplement: Supplementary file 2 [file DataSheet4.ZIP › Microscopy images-H&E_200x_50um/CAVO-H/CAVO-H4 H&E_200x_50um_1.jpeg]

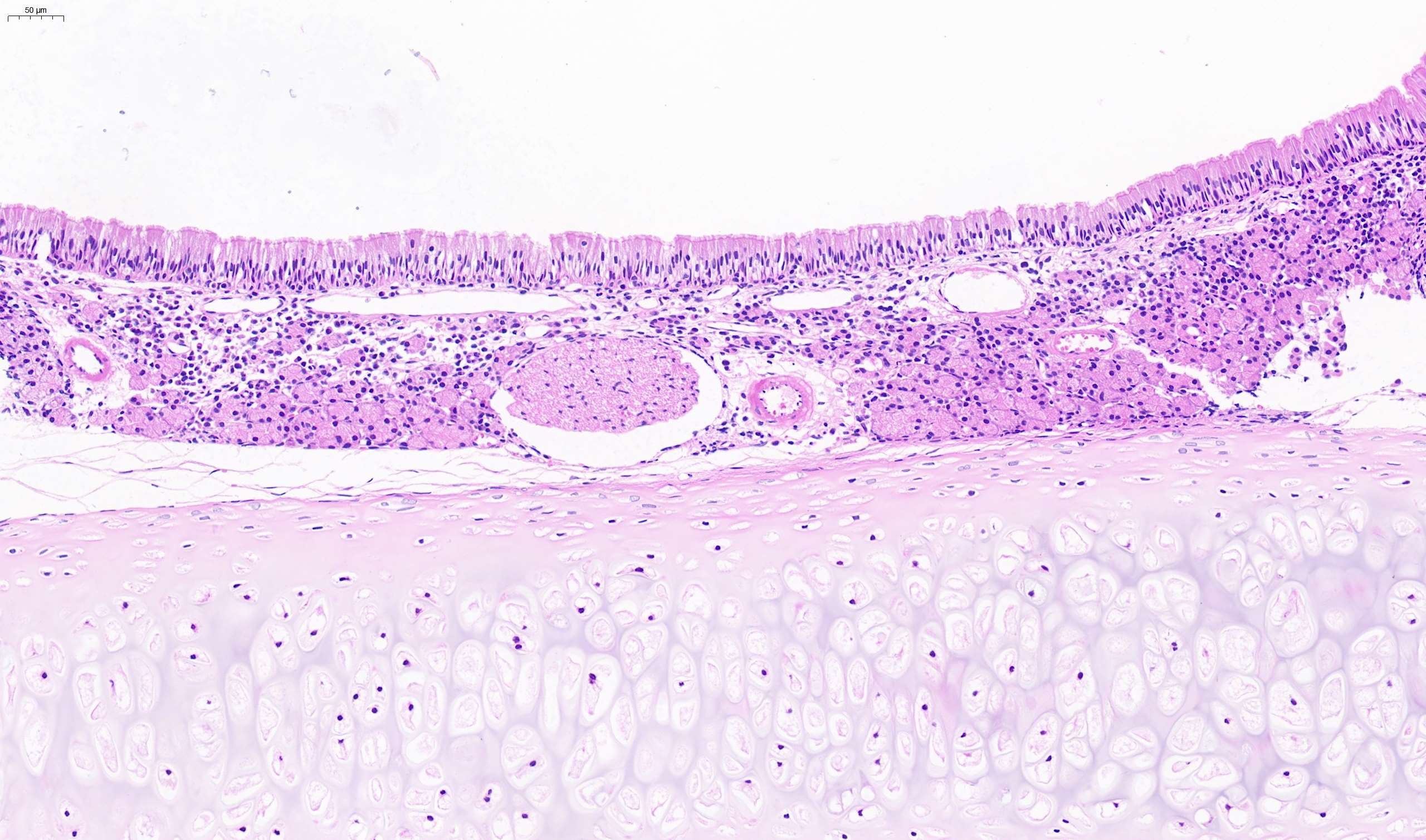

Supplement: Supplementary file 2 [file DataSheet4.ZIP › Microscopy images-H&E_200x_50um/CAVO-H/CAVO-H5 H&E_200x_50um_1.jpeg]

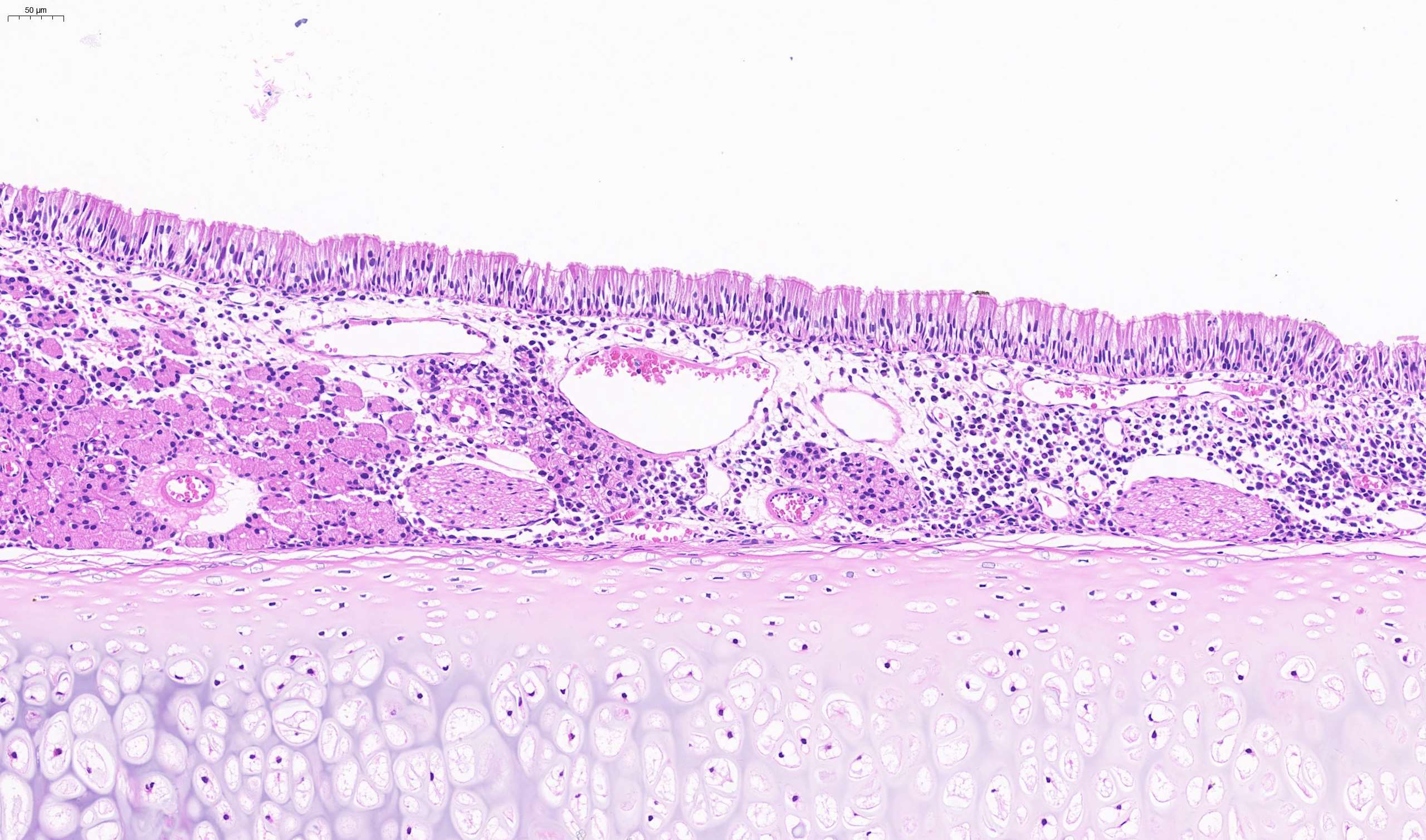

Supplement: Supplementary file 2 [file DataSheet4.ZIP › Microscopy images-H&E_200x_50um/CAVO-L/CAVO-L1 H&E_200x_50um_1.jpeg]

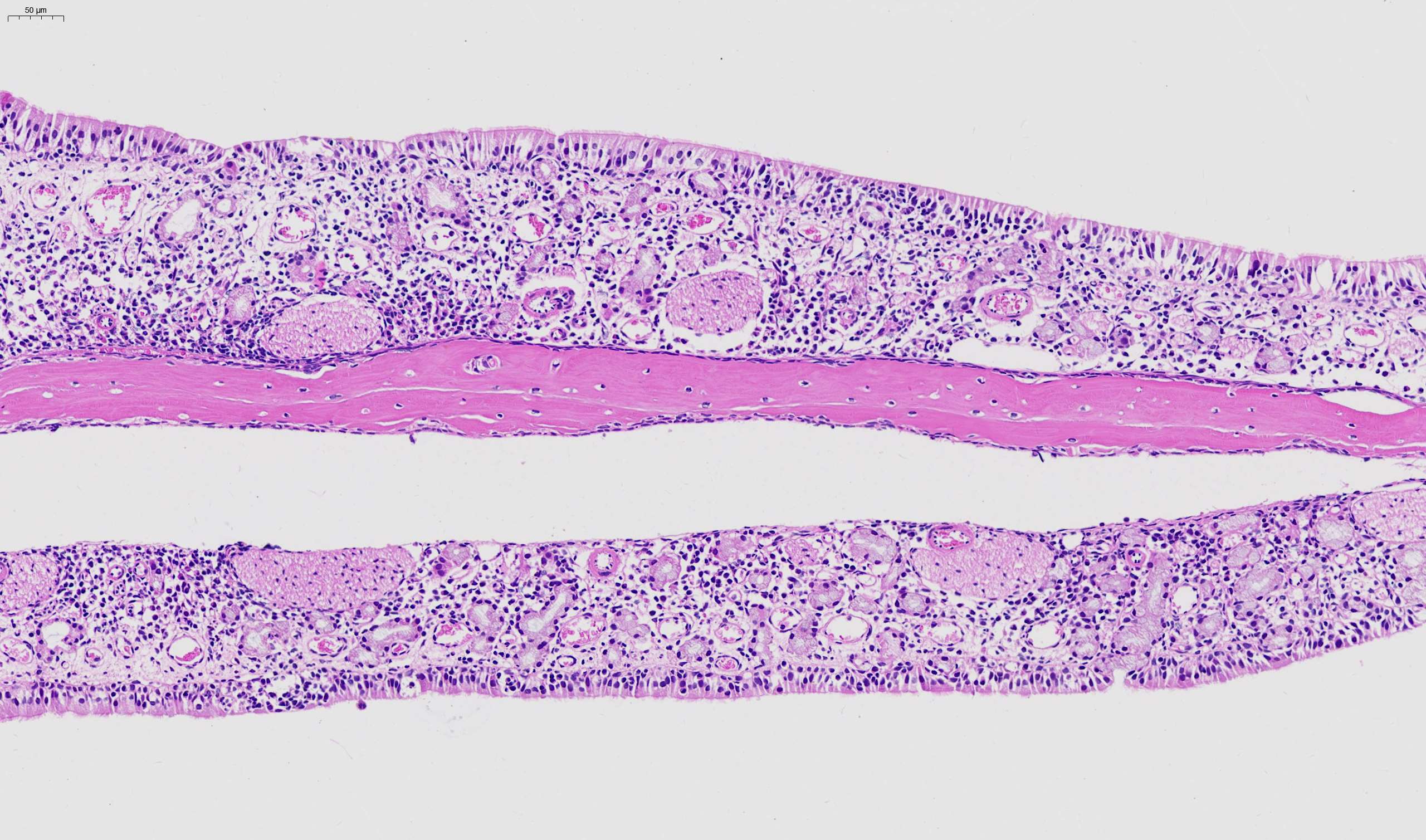

Supplement: Supplementary file 2 [file DataSheet4.ZIP › Microscopy images-H&E_200x_50um/CAVO-L/CAVO-L2 H&E_200x_50um_1.jpeg]

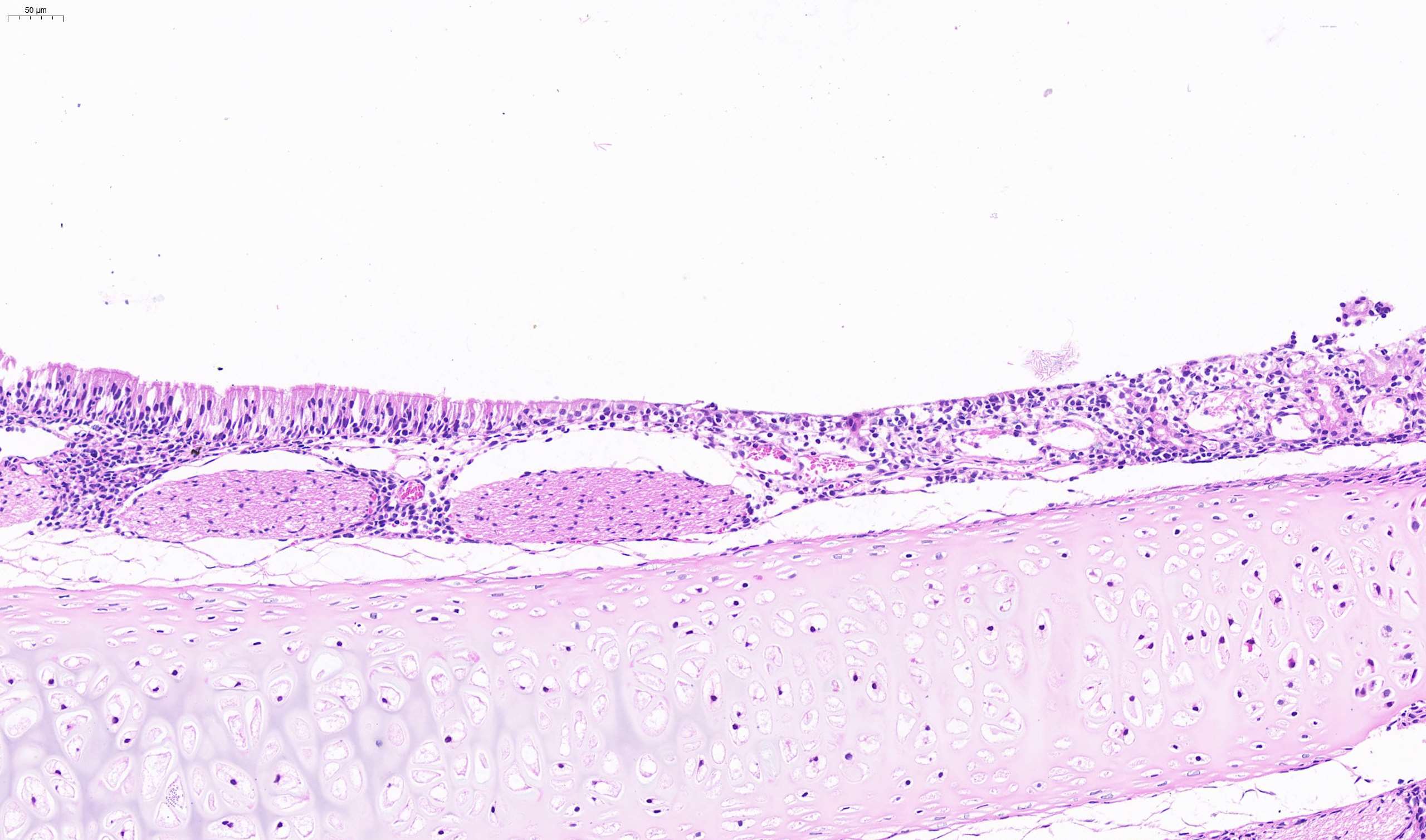

Supplement: Supplementary file 2 [file DataSheet4.ZIP › Microscopy images-H&E_200x_50um/CAVO-L/CAVO-L3 H&E_200x_50um_1.jpeg]

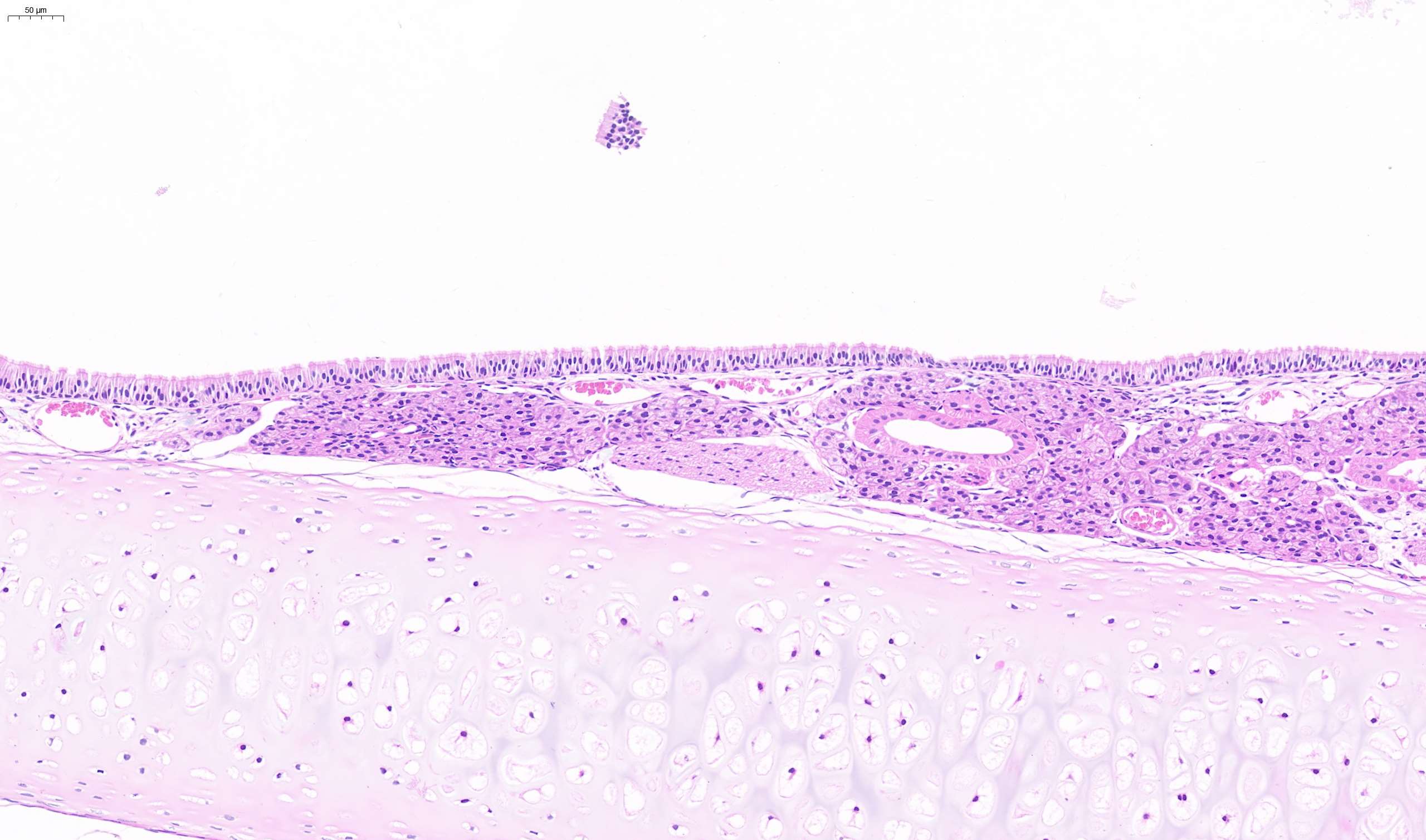

Supplement: Supplementary file 2 [file DataSheet4.ZIP › Microscopy images-H&E_200x_50um/CAVO-L/CAVO-L4 H&E_200x_50um_1.jpeg]

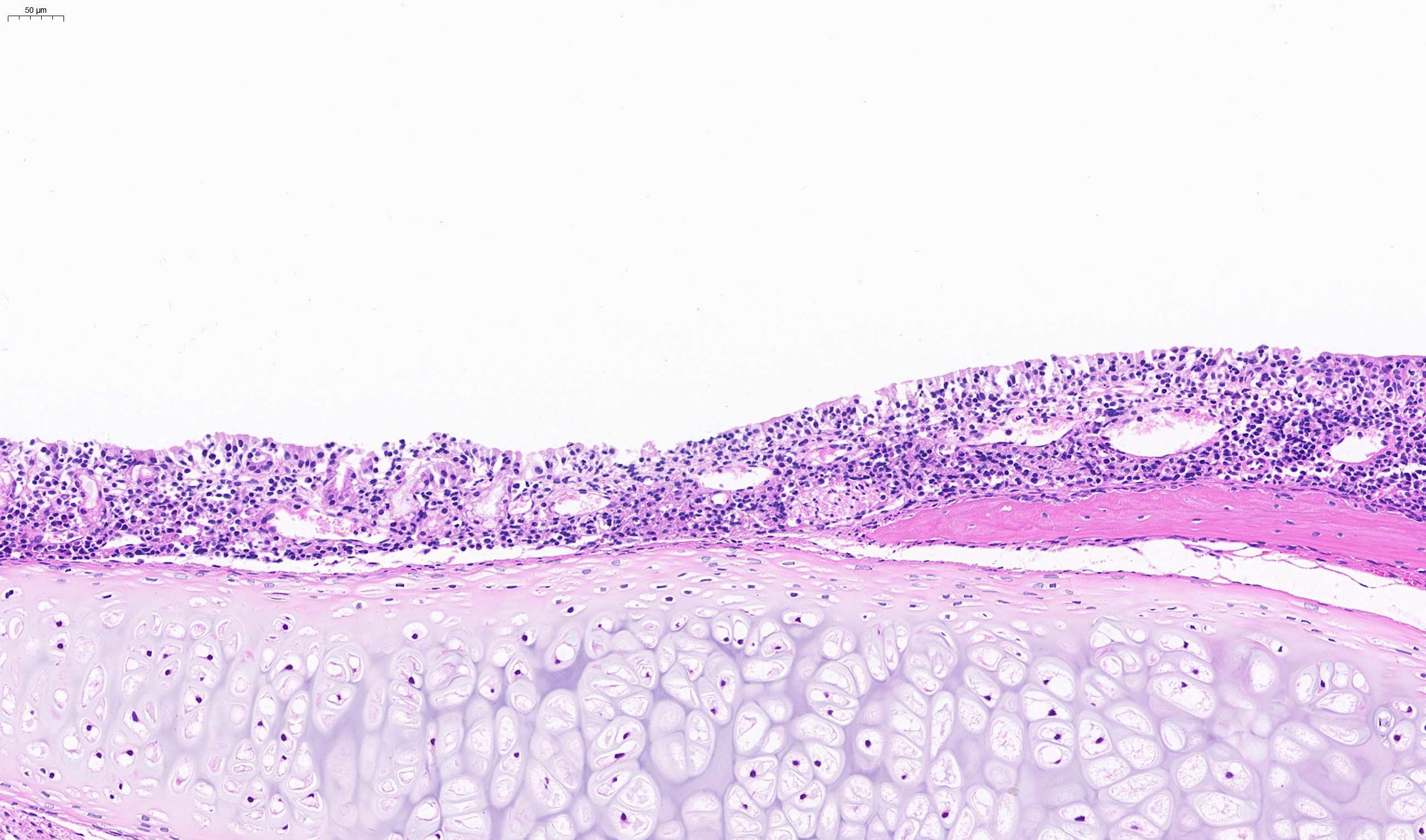

Supplement: Supplementary file 2 [file DataSheet4.ZIP › Microscopy images-H&E_200x_50um/CAVO-L/CAVO-L5 H&E_200x_50um_1.jpeg]

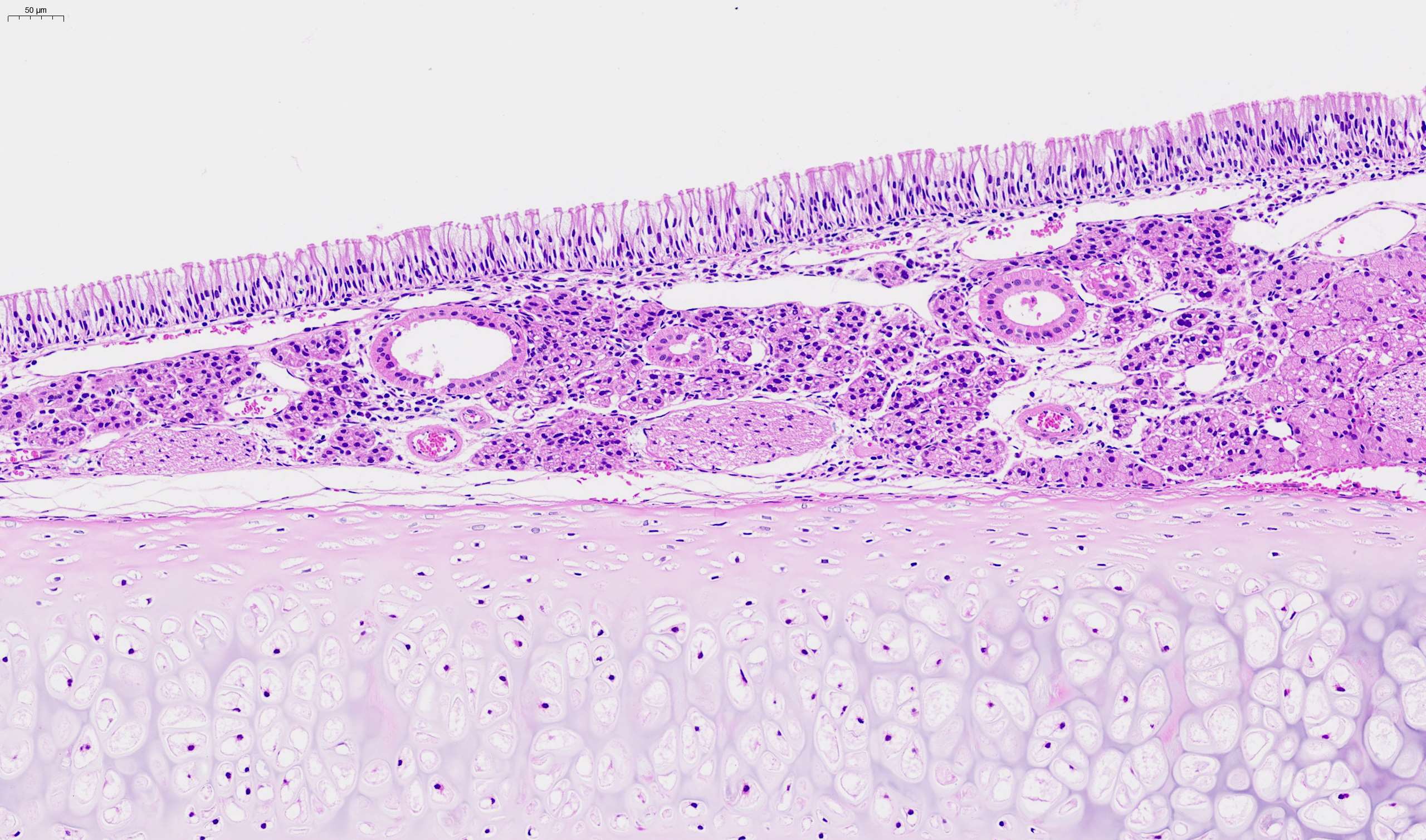

Supplement: Supplementary file 2 [file DataSheet4.ZIP › Microscopy images-H&E_200x_50um/CAVO-M/CAVO-M1 H&E_200x_50um_1.jpeg]

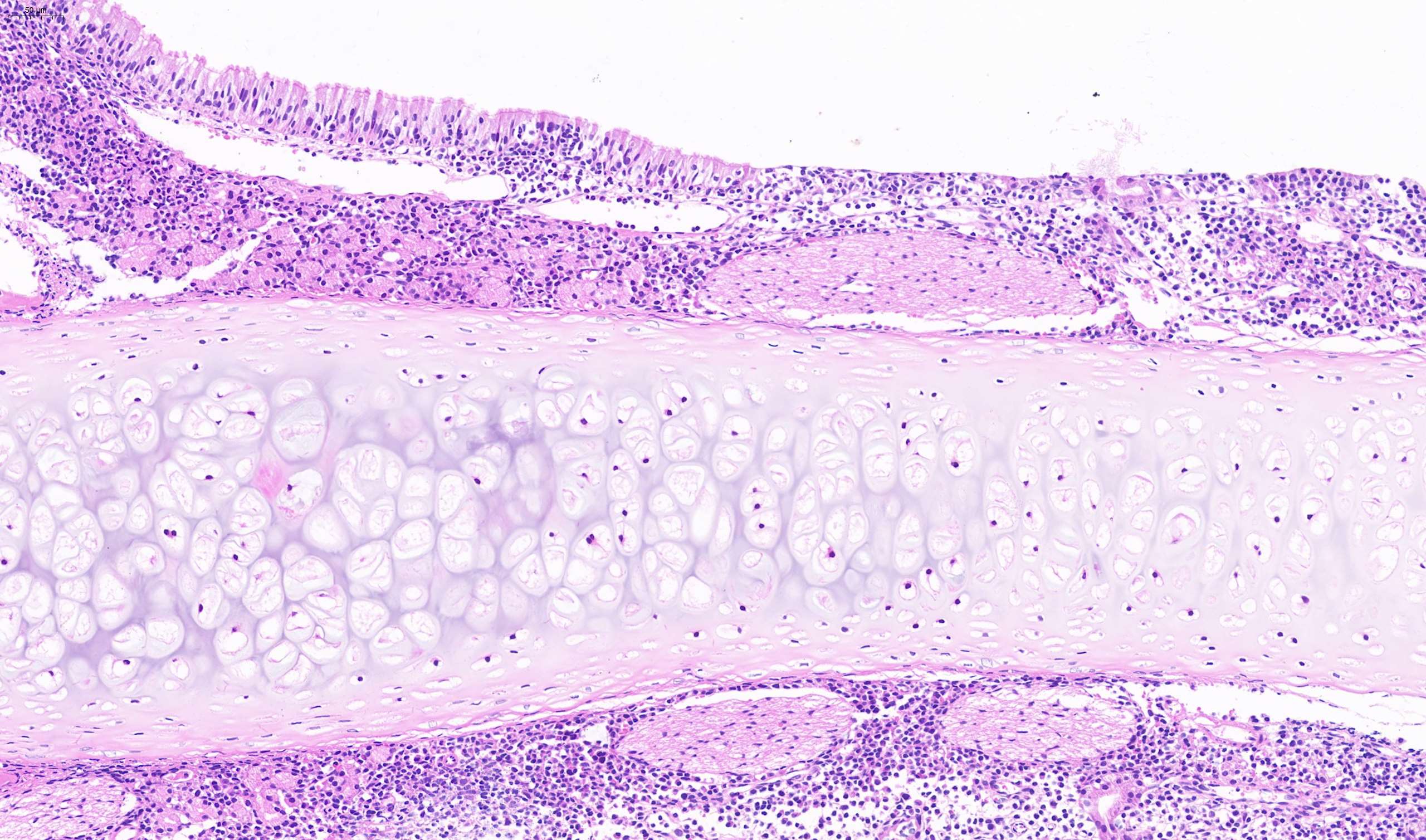

Supplement: Supplementary file 2 [file DataSheet4.ZIP › Microscopy images-H&E_200x_50um/CAVO-M/CAVO-M2 H&E_200x_50um_1.jpeg]

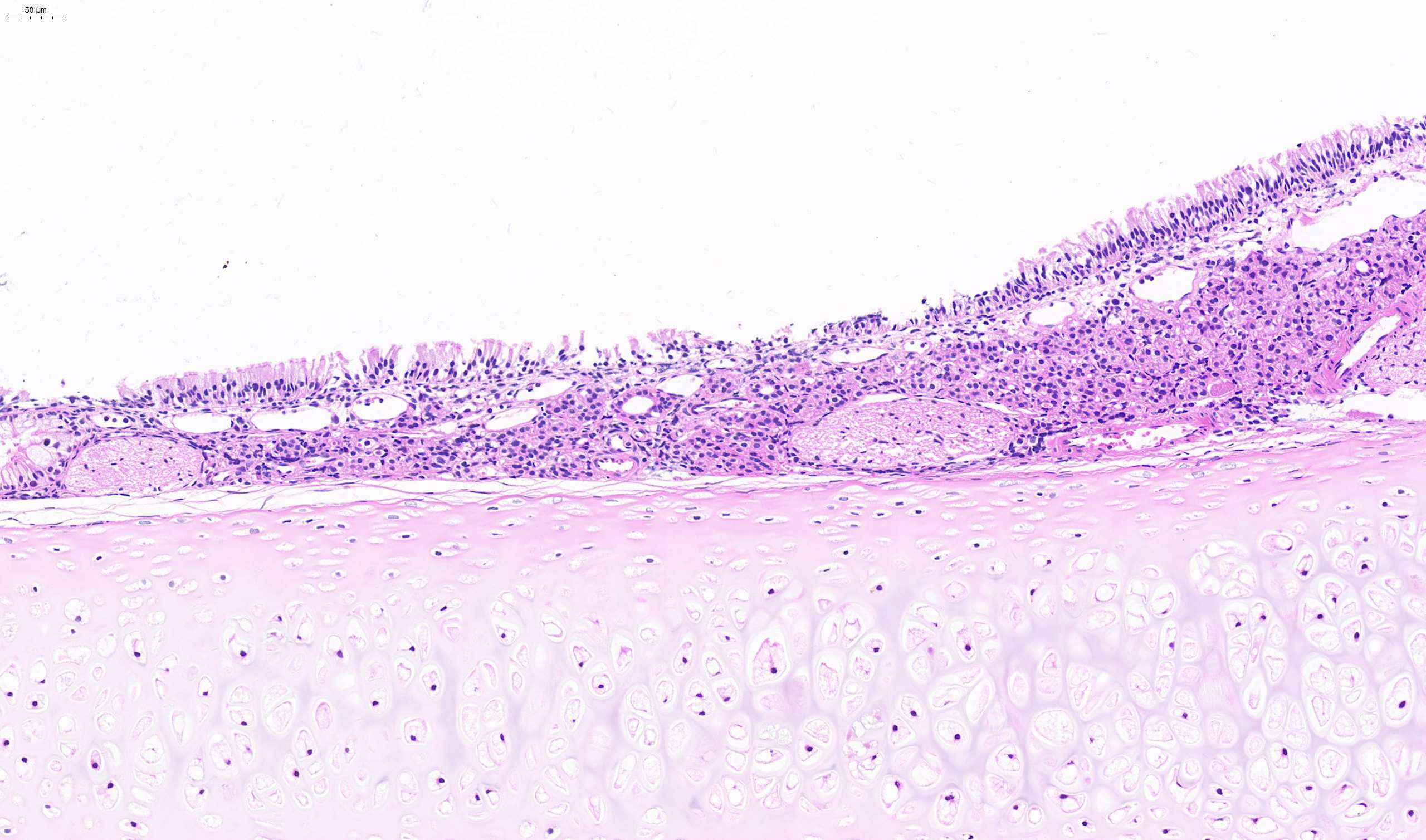

Supplement: Supplementary file 2 [file DataSheet4.ZIP › Microscopy images-H&E_200x_50um/CAVO-M/CAVO-M3 H&E_200x_50um_1.jpeg]

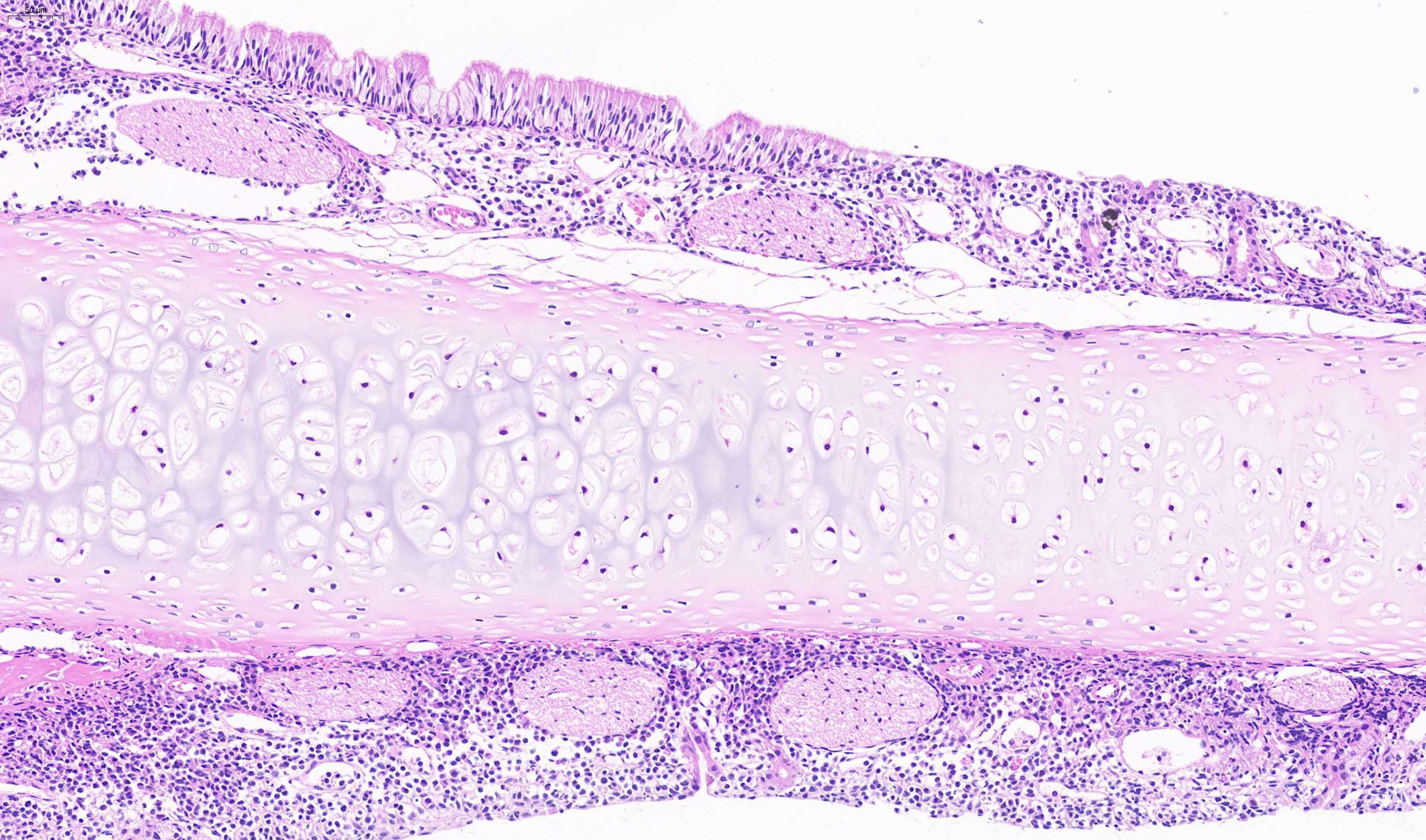

Supplement: Supplementary file 2 [file DataSheet4.ZIP › Microscopy images-H&E_200x_50um/CAVO-M/CAVO-M4 H&E_200x_50um_1.jpeg]

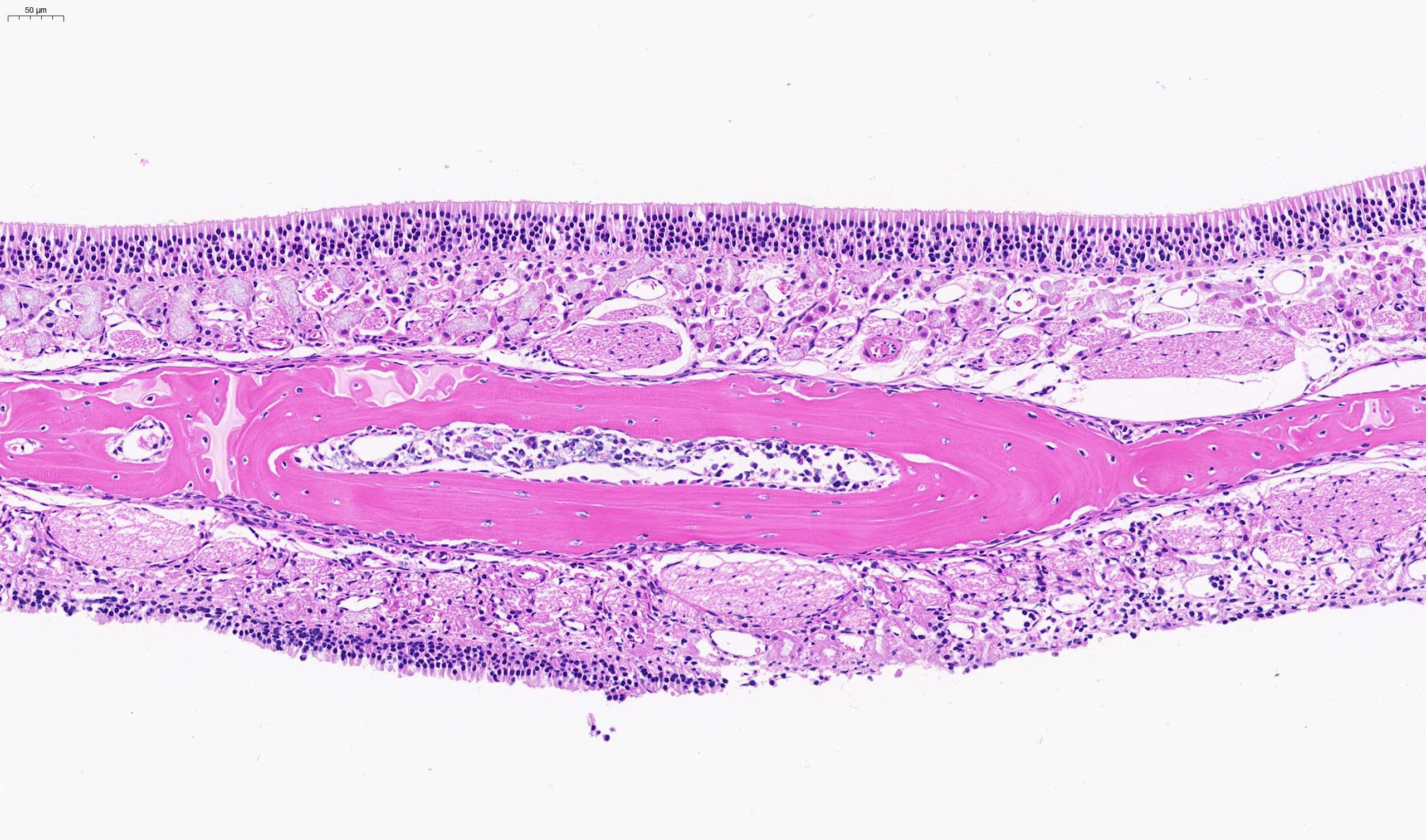

Supplement: Supplementary file 2 [file DataSheet4.ZIP › Microscopy images-H&E_200x_50um/CAVO-M/CAVO-M5 H&E_200x_50um_1.jpeg]

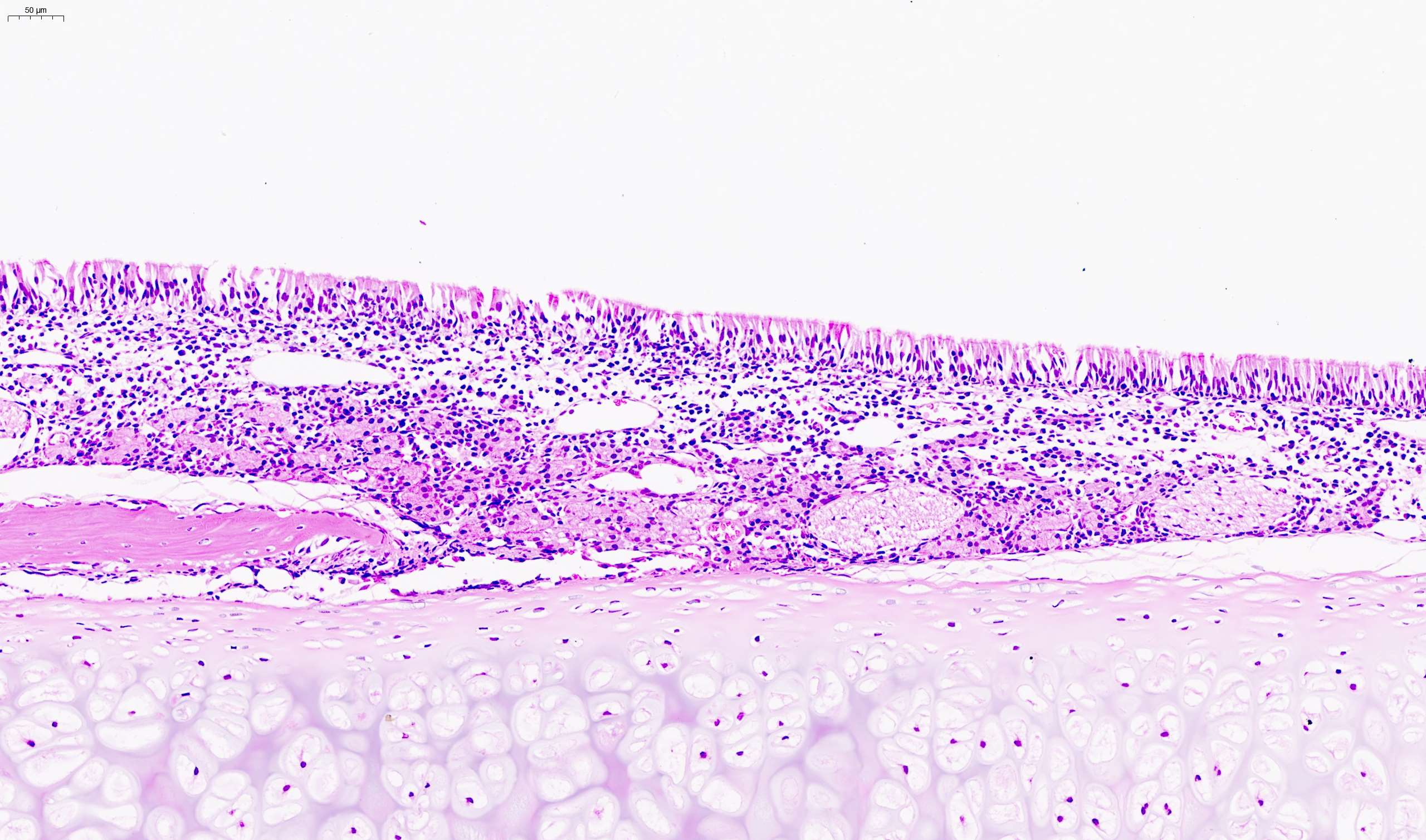

Supplement: Supplementary file 2 [file DataSheet4.ZIP › Microscopy images-H&E_200x_50um/Control/Control 1 H&E_200x_50um_1.jpeg]

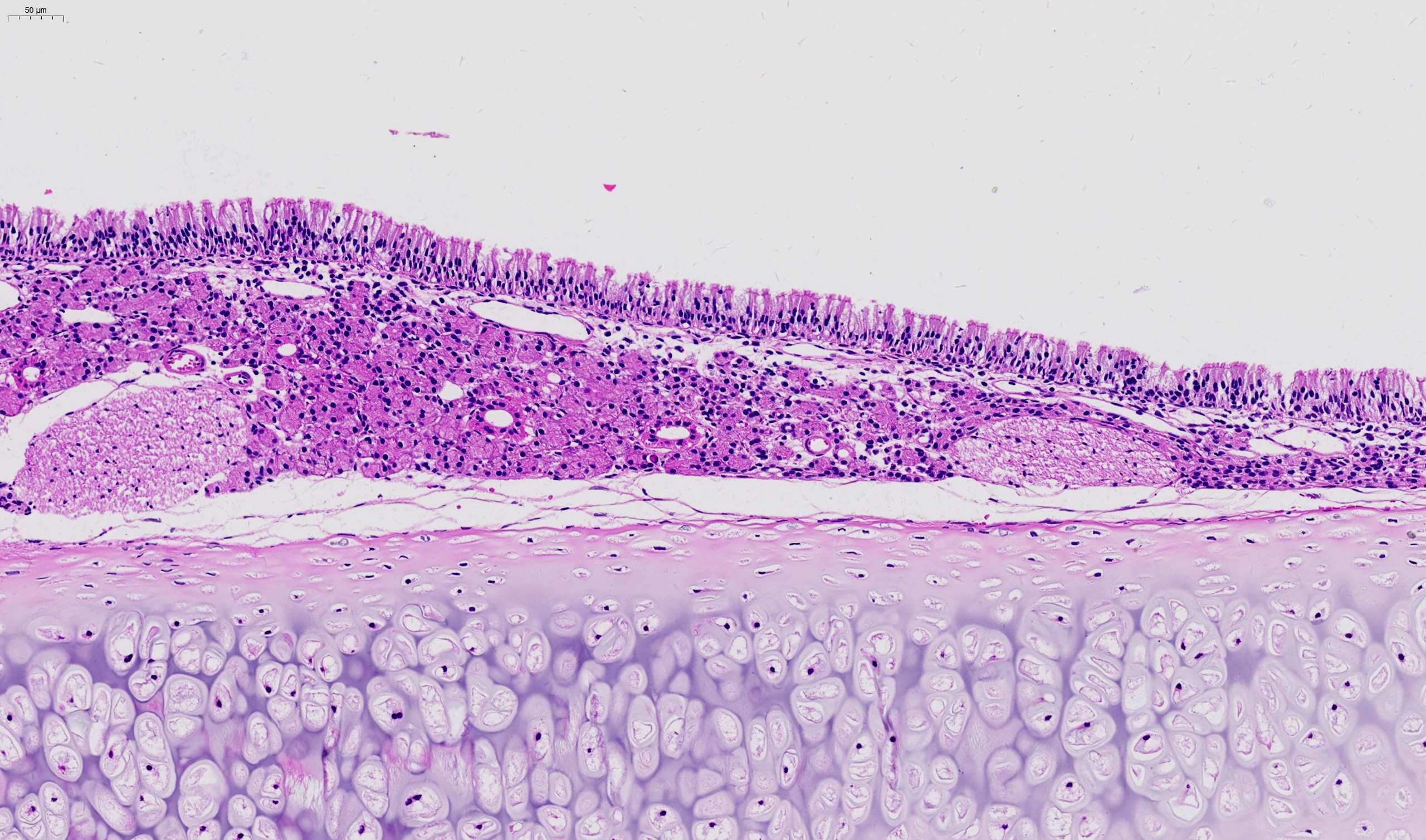

Supplement: Supplementary file 2 [file DataSheet4.ZIP › Microscopy images-H&E_200x_50um/Control/Control 2 H&E_200x_50um_1.jpeg]

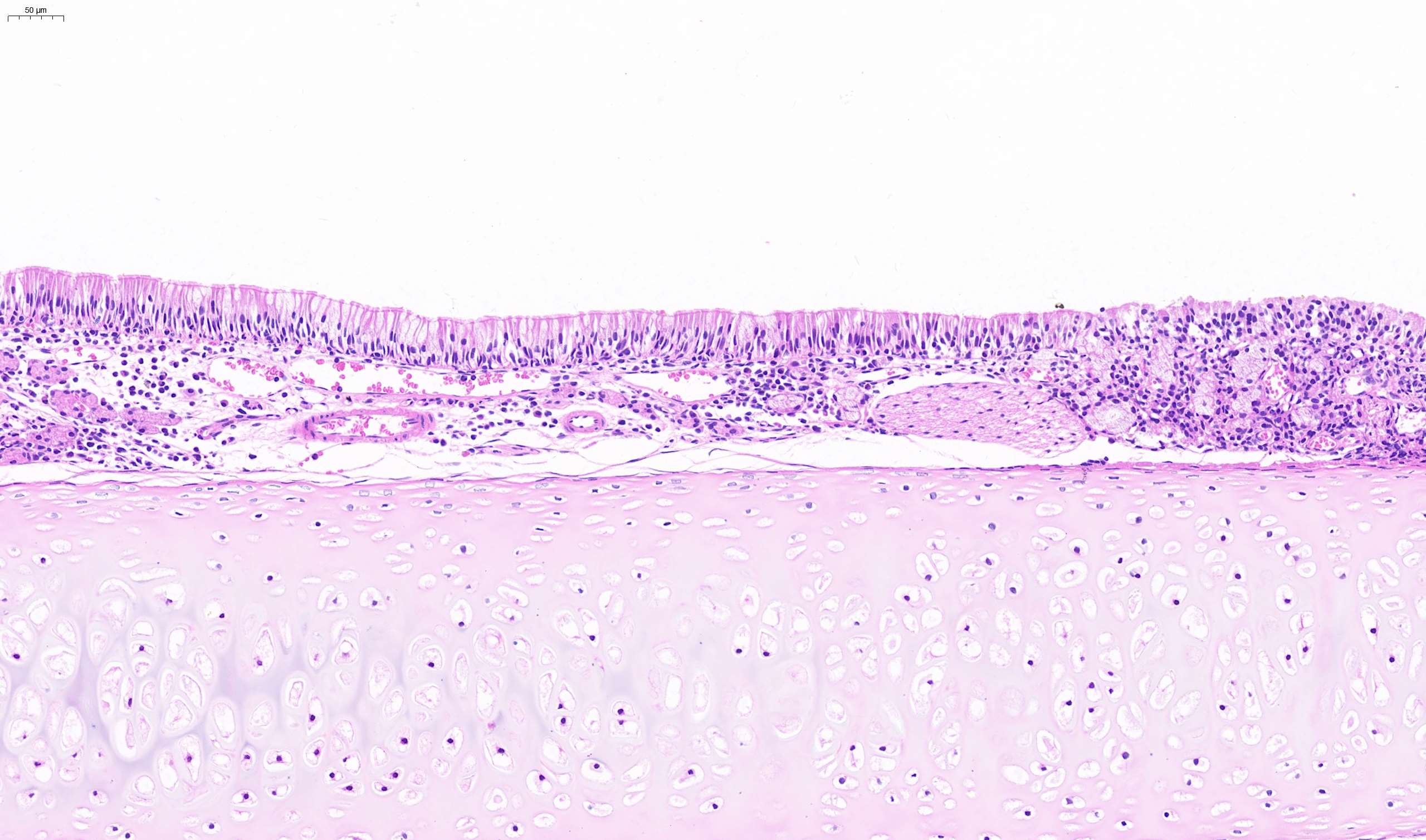

Supplement: Supplementary file 2 [file DataSheet4.ZIP › Microscopy images-H&E_200x_50um/Control/Control3 H&E_200x_50um_1.jpeg]

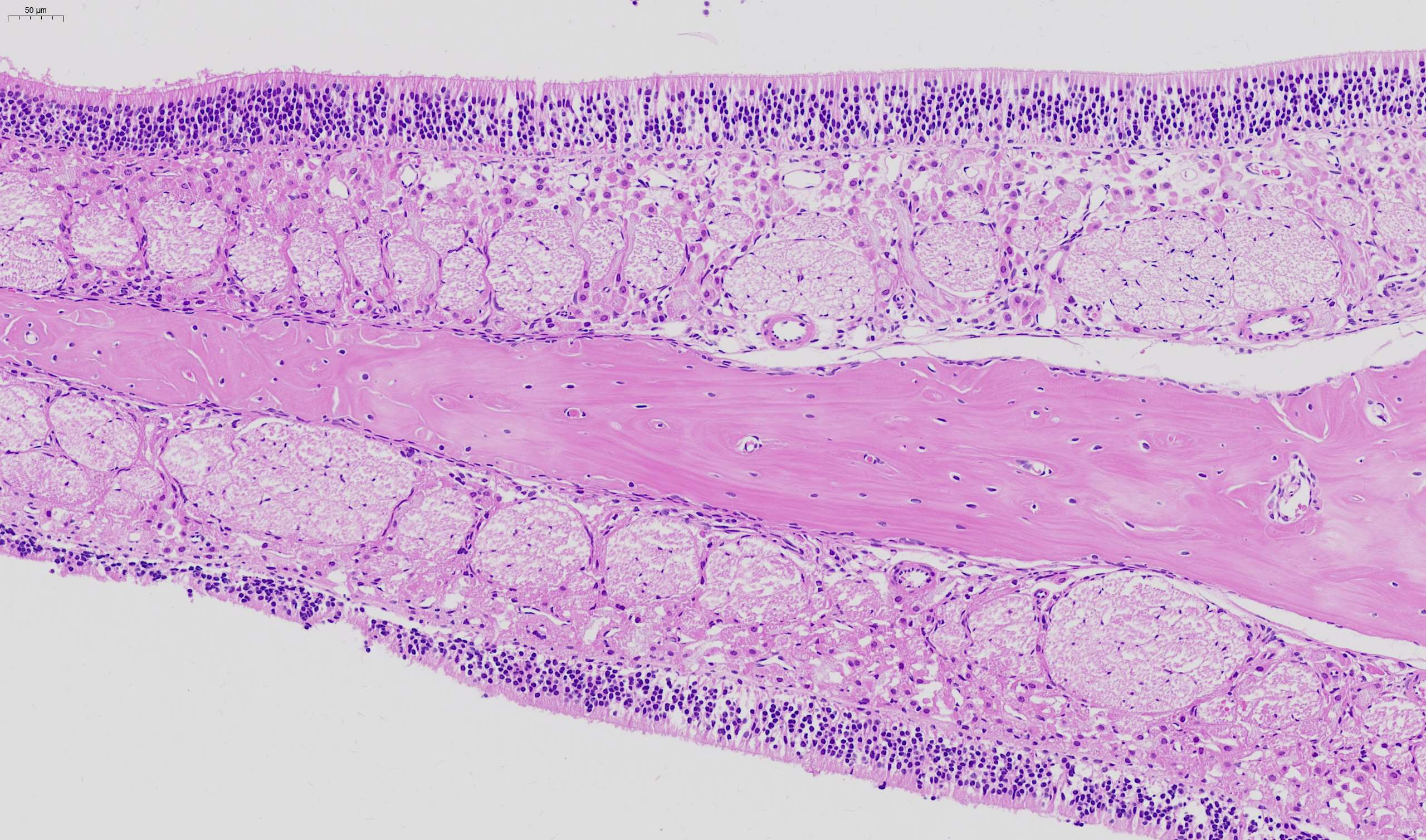

Supplement: Supplementary file 2 [file DataSheet4.ZIP › Microscopy images-H&E_200x_50um/Control/Control4 H&E_200x_50um_1.jpeg]

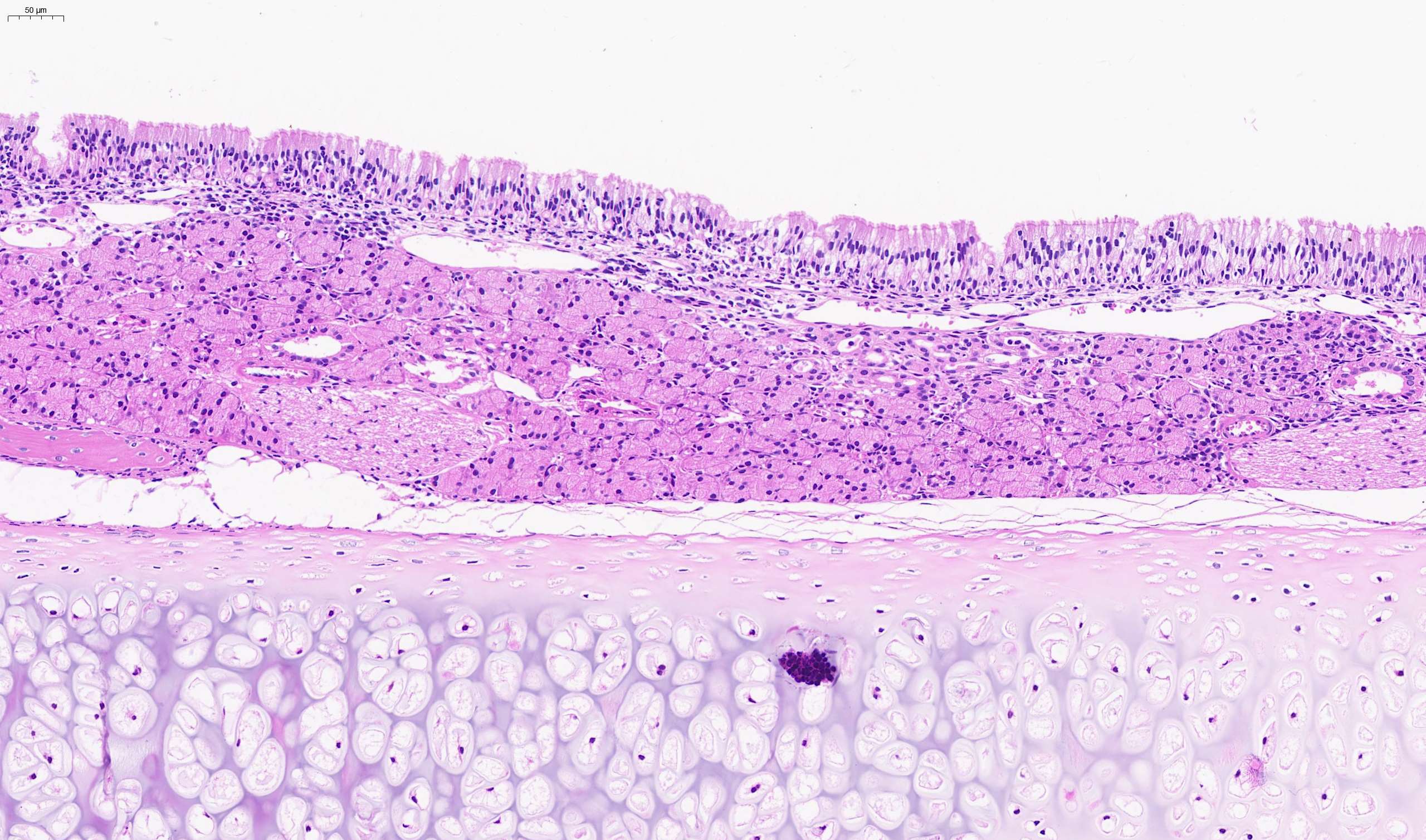

Supplement: Supplementary file 2 [file DataSheet4.ZIP › Microscopy images-H&E_200x_50um/Control/Control5 H&E_200x_50um_1.jpeg]

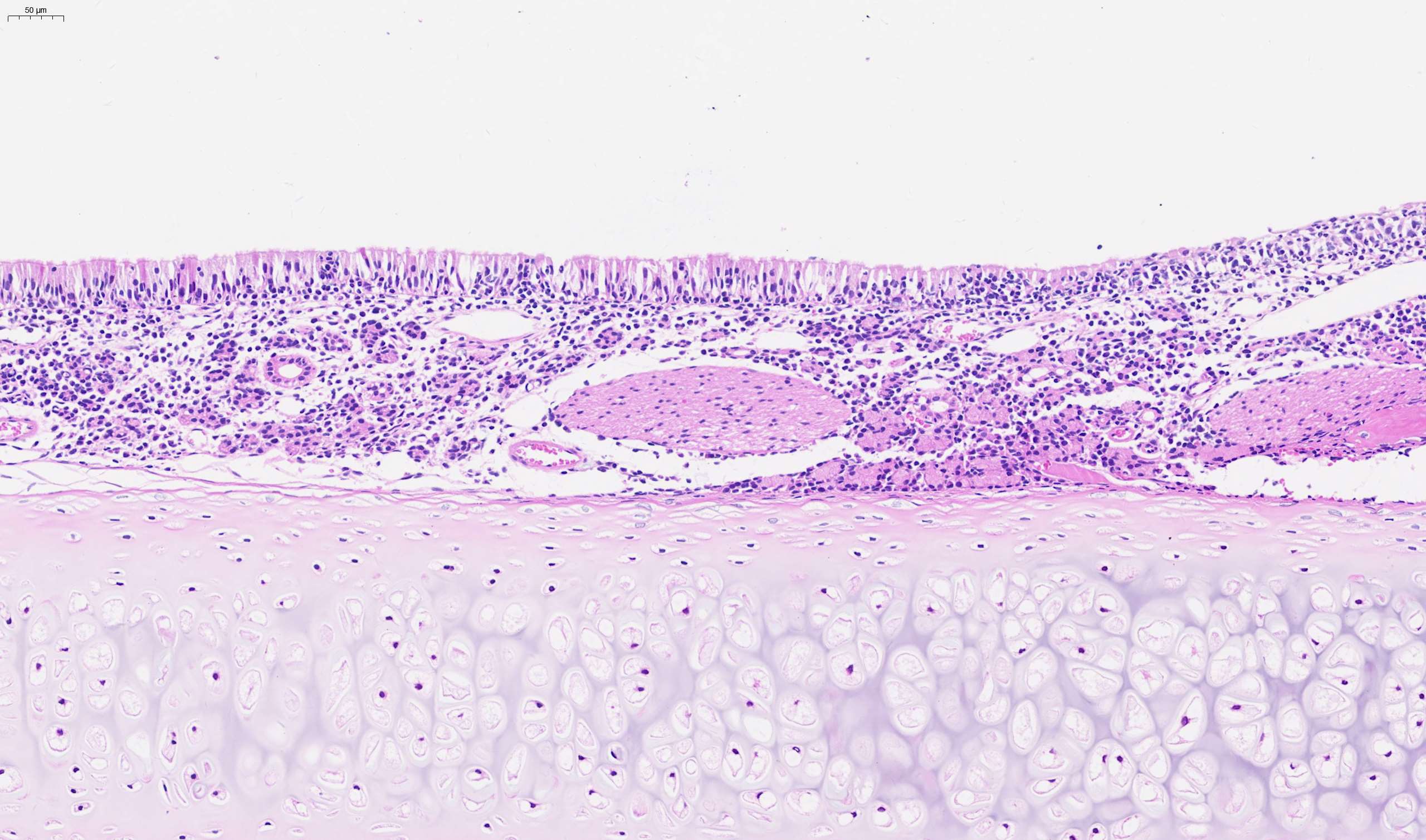

Supplement: Supplementary file 2 [file DataSheet4.ZIP › Microscopy images-H&E_200x_50um/Loratadine/Loratadine1 H&E_200x_50um_1.jpeg]

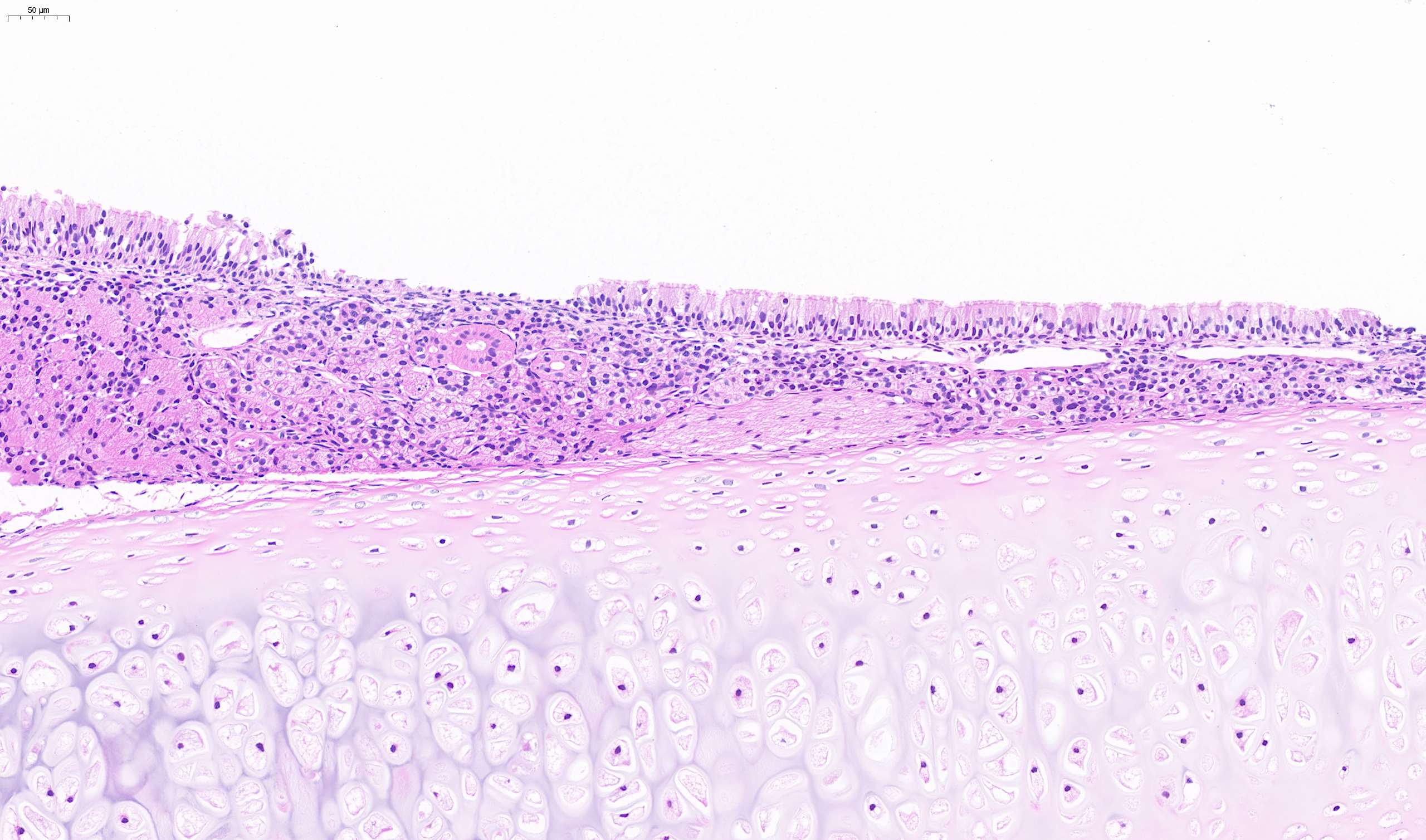

Supplement: Supplementary file 2 [file DataSheet4.ZIP › Microscopy images-H&E_200x_50um/Loratadine/Loratadine2 H&E_200x_50um_1.jpeg]

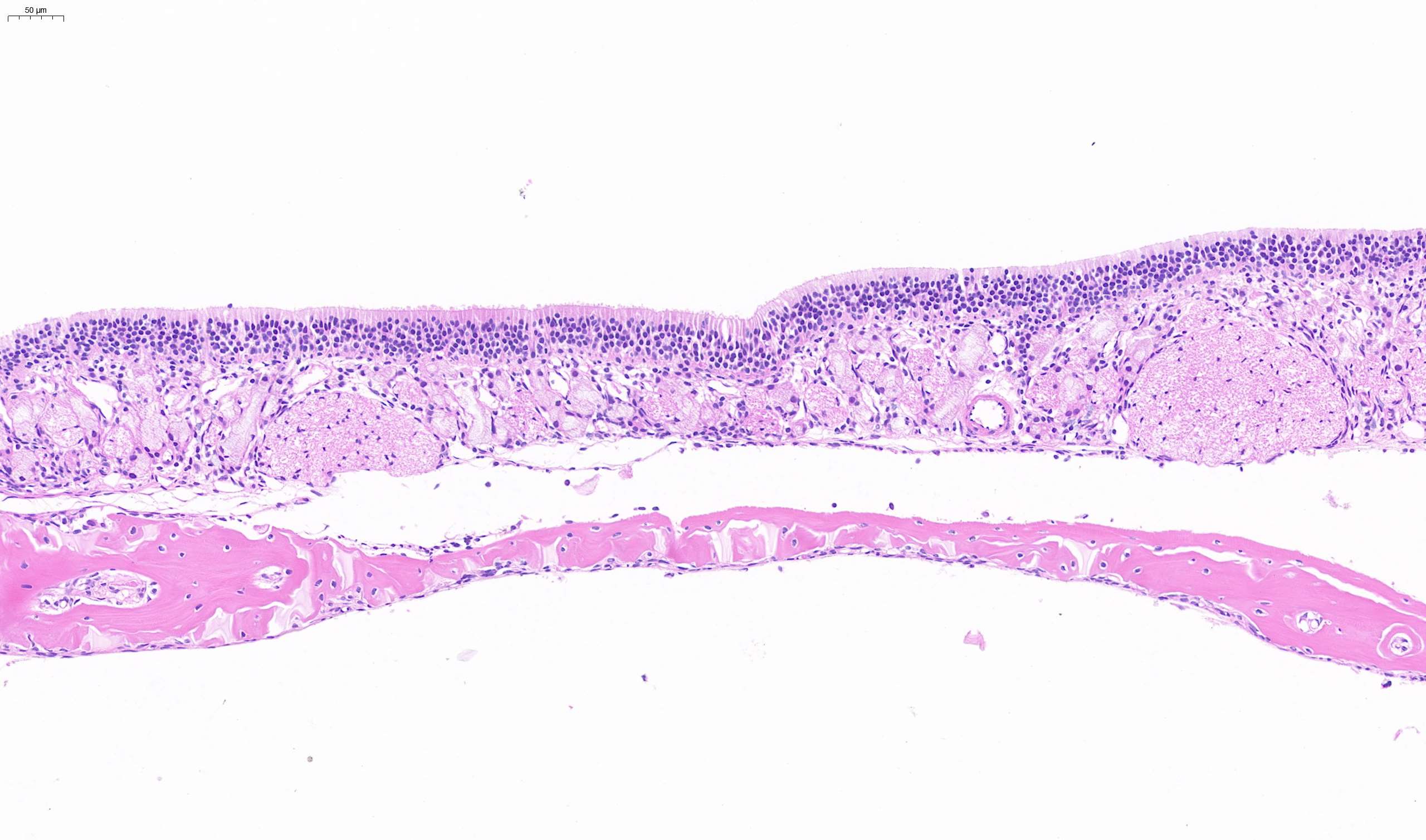

Supplement: Supplementary file 2 [file DataSheet4.ZIP › Microscopy images-H&E_200x_50um/Loratadine/Loratadine3 H&E_200x_50um_1.jpeg]

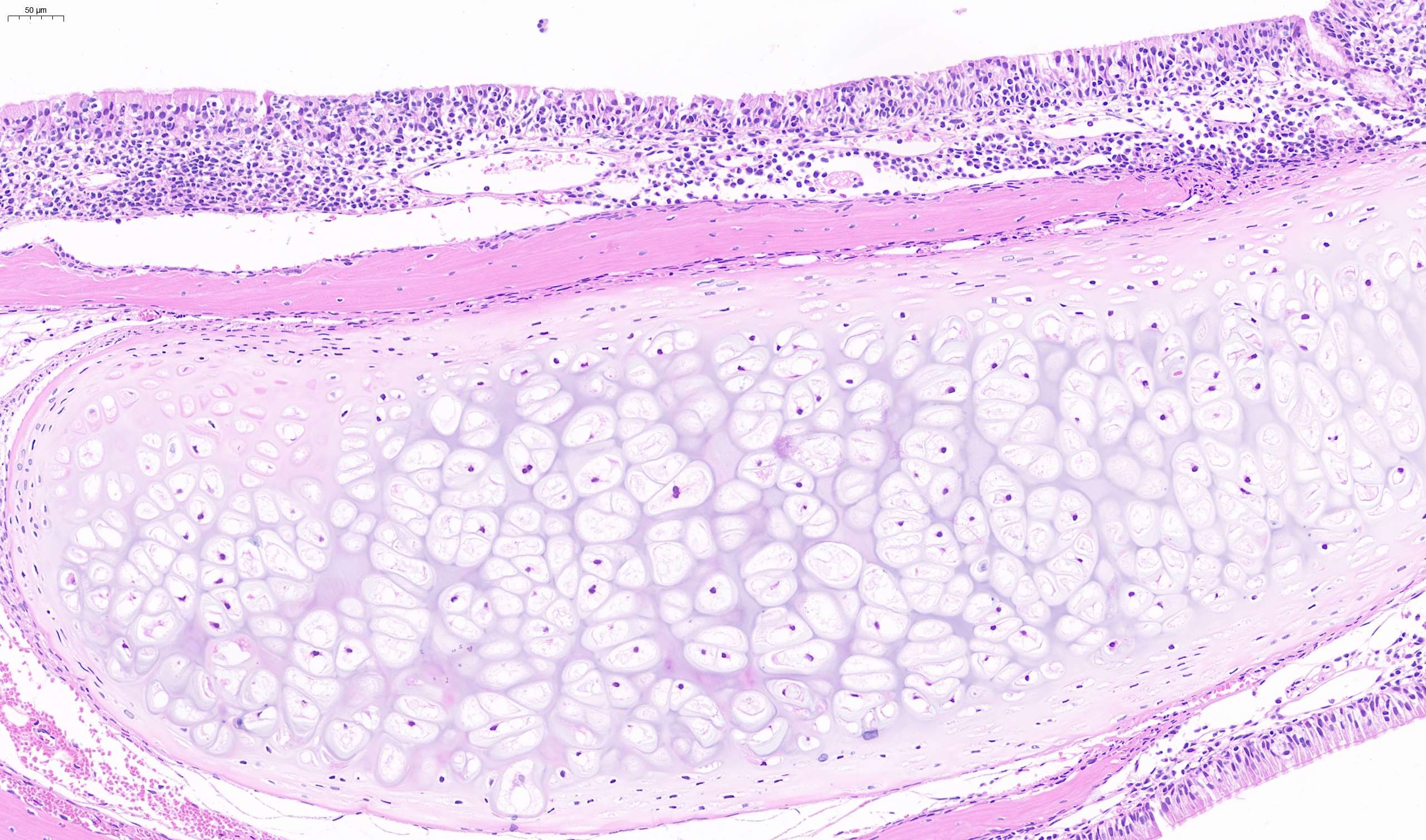

Supplement: Supplementary file 2 [file DataSheet4.ZIP › Microscopy images-H&E_200x_50um/Loratadine/Loratadine4 H&E_200x_50um_1.jpeg]

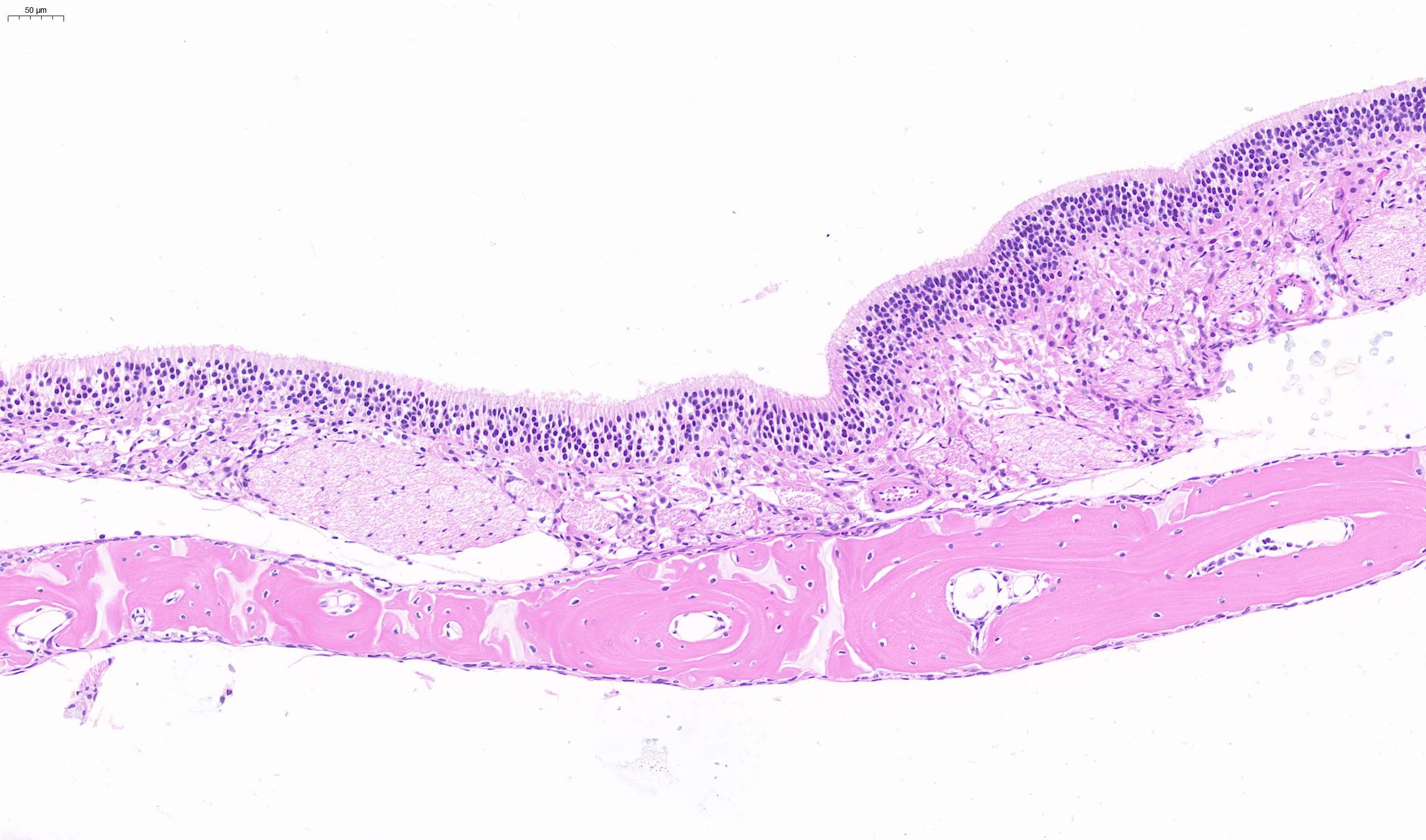

Supplement: Supplementary file 2 [file DataSheet4.ZIP › Microscopy images-H&E_200x_50um/Loratadine/Loratadine5 H&E_200x_50um_1.jpeg]

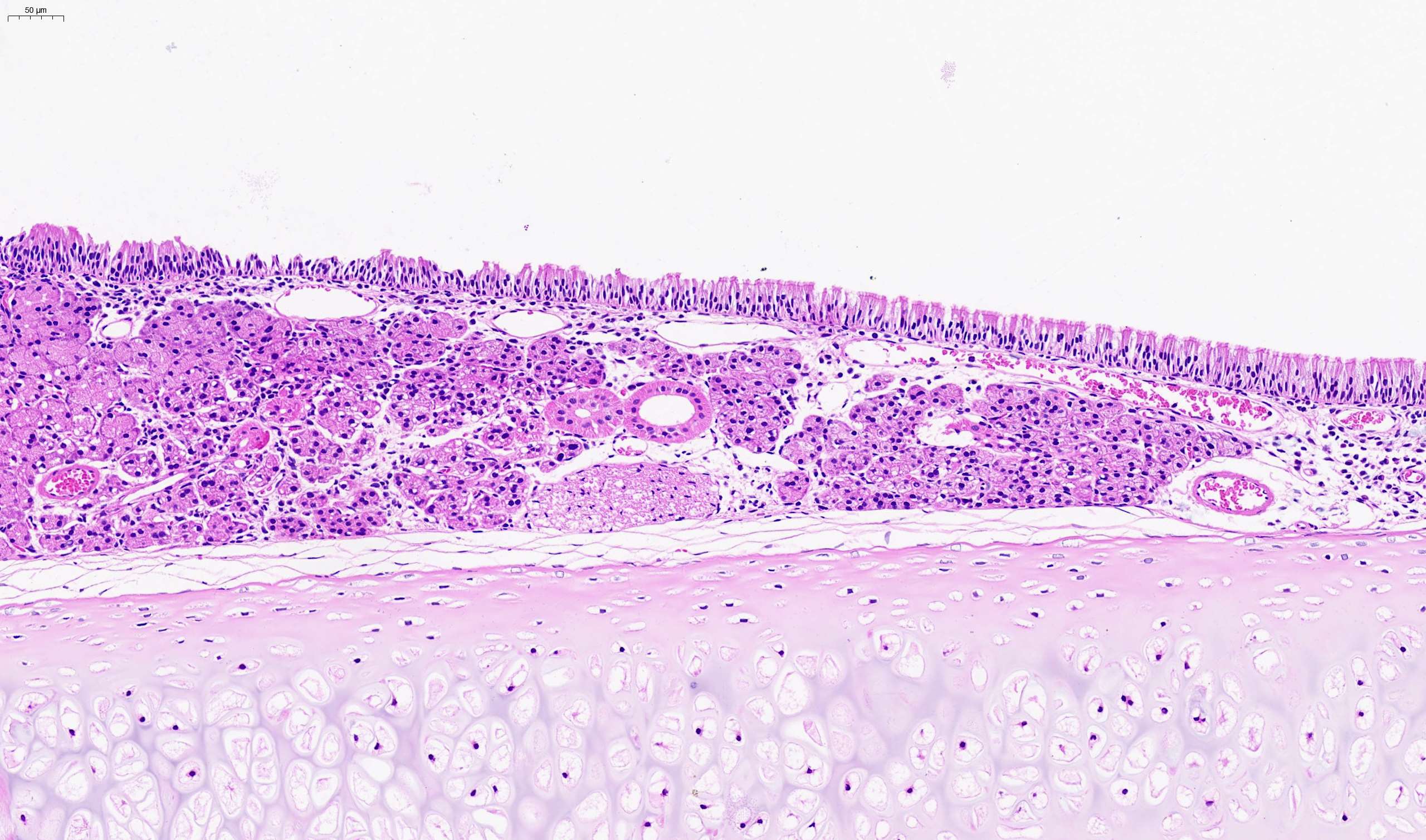

Supplement: Supplementary file 2 [file DataSheet4.ZIP › Microscopy images-H&E_200x_50um/Model/Model1 H&E_200x_50um_1.jpeg]

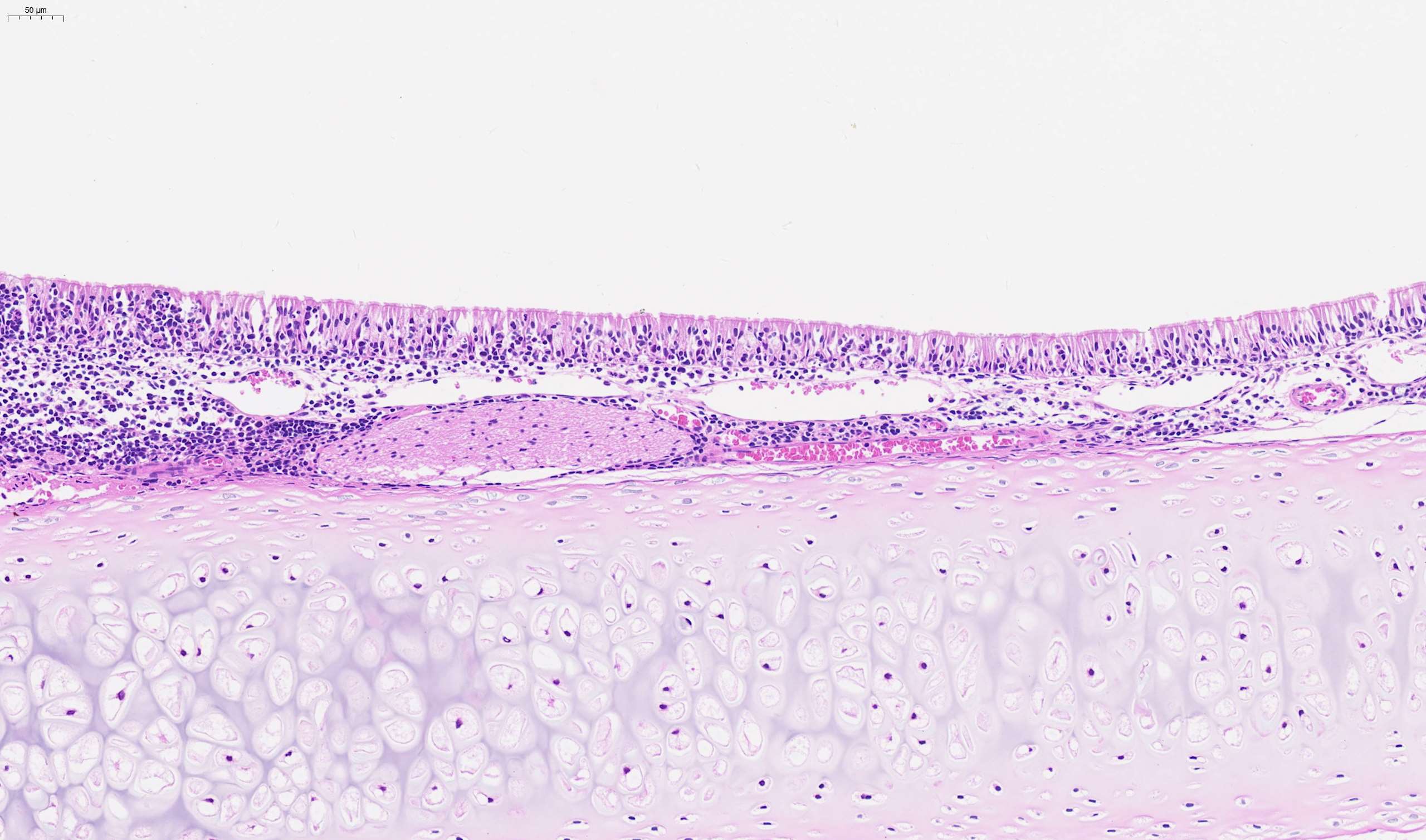

Supplement: Supplementary file 2 [file DataSheet4.ZIP › Microscopy images-H&E_200x_50um/Model/Model2 H&E_200x_50um_1.jpeg]

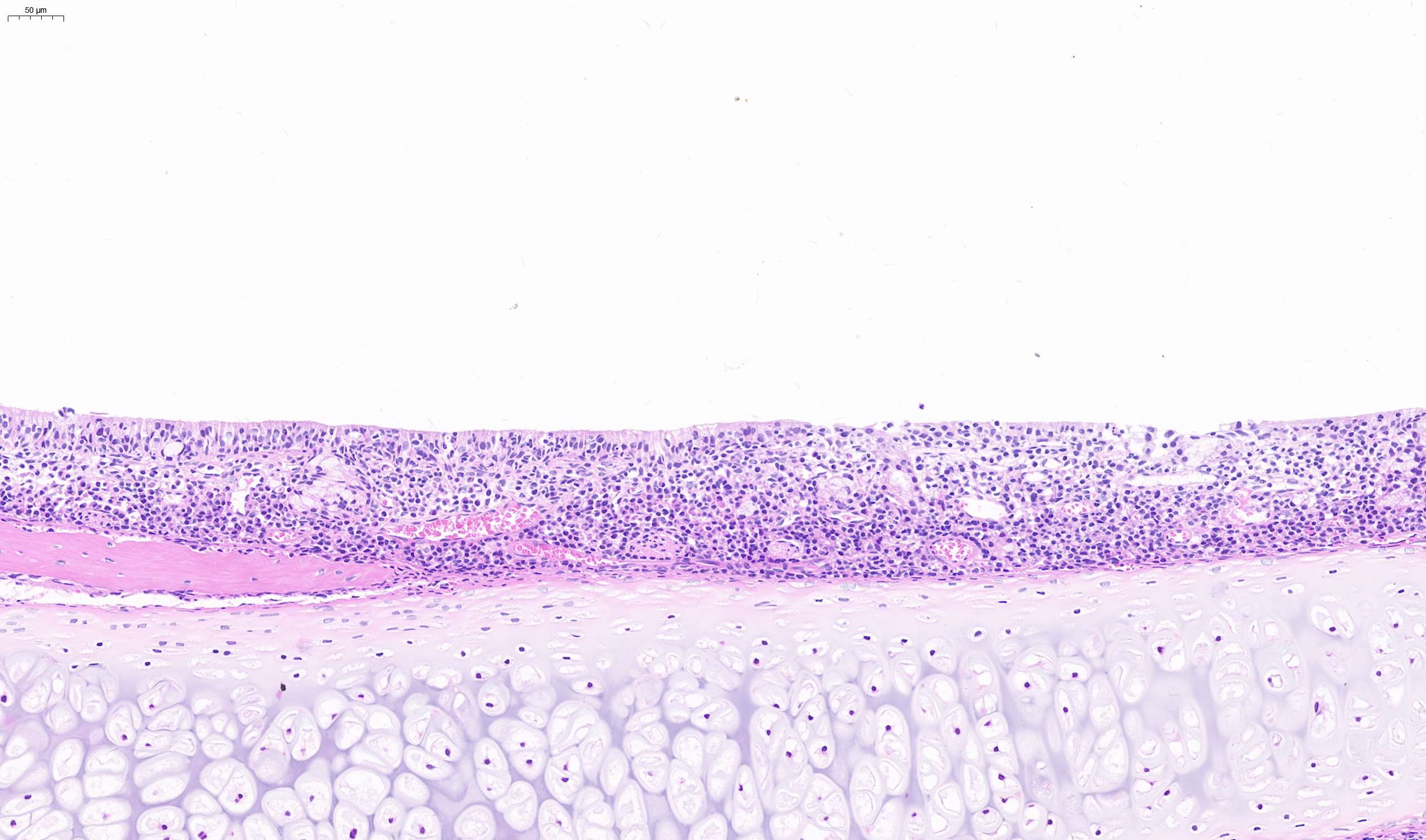

Supplement: Supplementary file 2 [file DataSheet4.ZIP › Microscopy images-H&E_200x_50um/Model/Model3 H&E_200x_50um_1.jpeg]

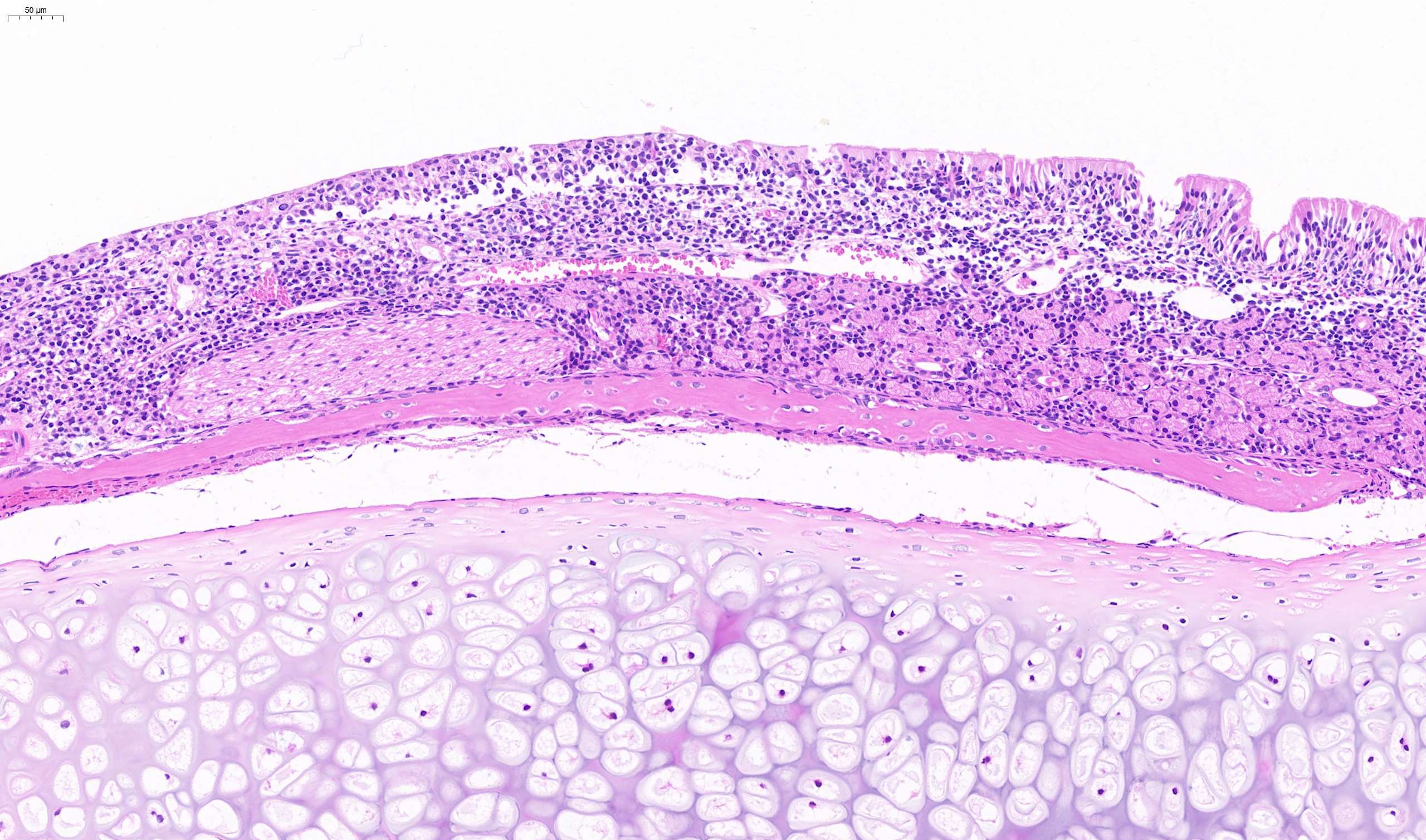

Supplement: Supplementary file 2 [file DataSheet4.ZIP › Microscopy images-H&E_200x_50um/Model/Model4 H&E_200x_50um_1.jpeg]

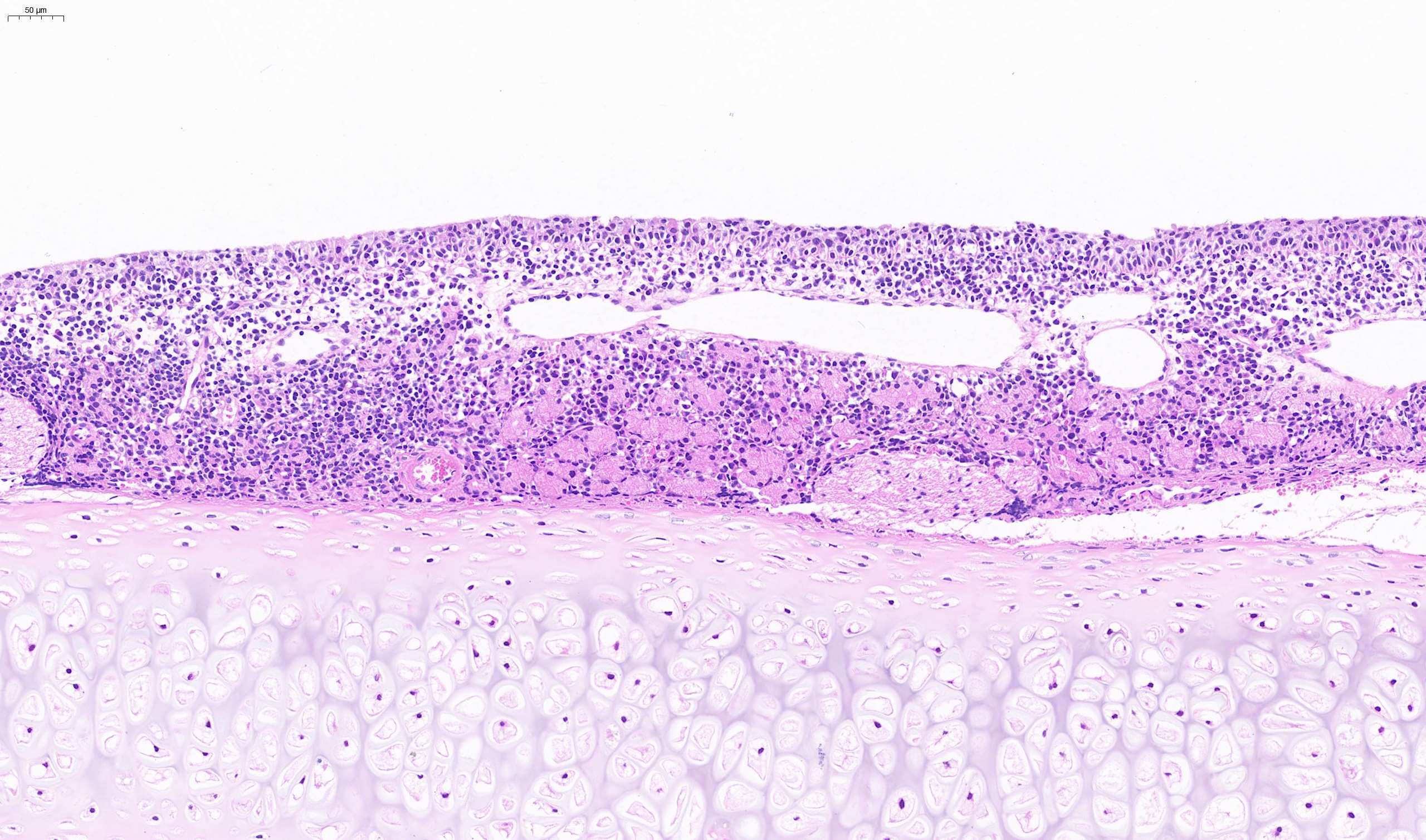

Supplement: Supplementary file 2 [file DataSheet4.ZIP › Microscopy images-H&E_200x_50um/Model/Model5 H&E_200x_50um_1.jpeg]

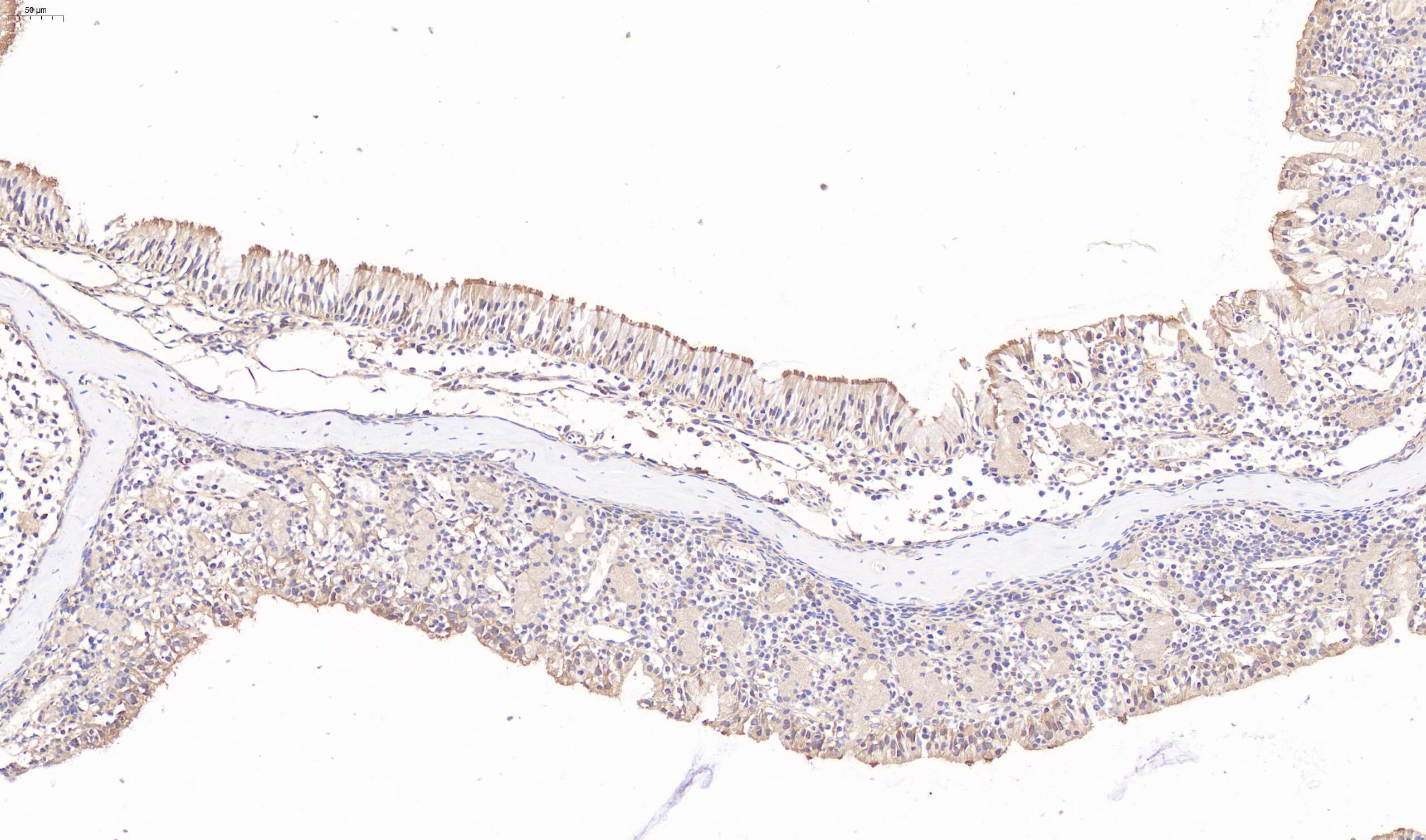

Supplement: Supplementary file 4 [file DataSheet6.ZIP › Microscopy images-Immunohistochemistry-T-bet_200x_50um/CAVO-H/1 T-bet_200x_50um_1.jpeg]

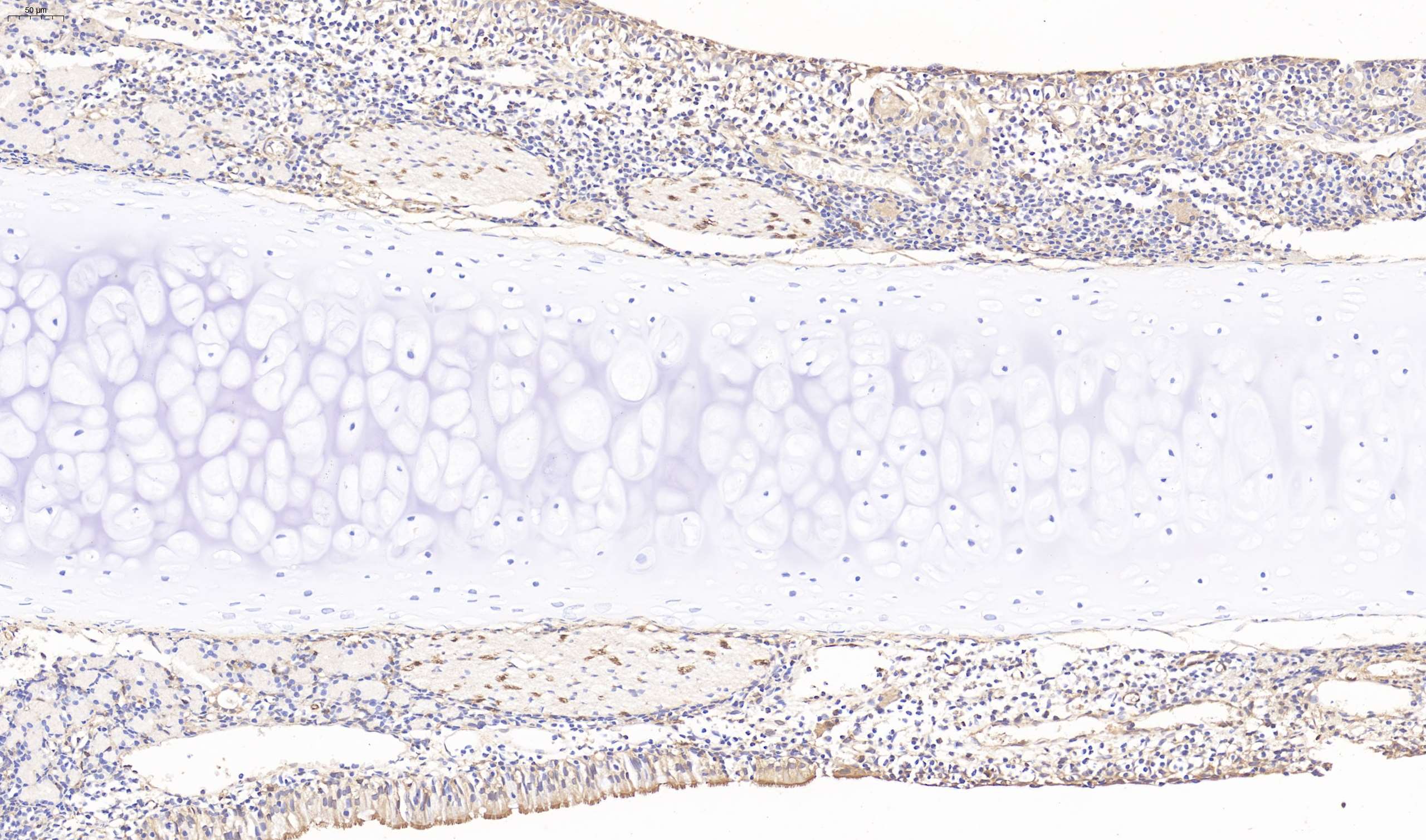

Supplement: Supplementary file 4 [file DataSheet6.ZIP › Microscopy images-Immunohistochemistry-T-bet_200x_50um/CAVO-H/2 T-bet_200x_50um_1.jpeg]

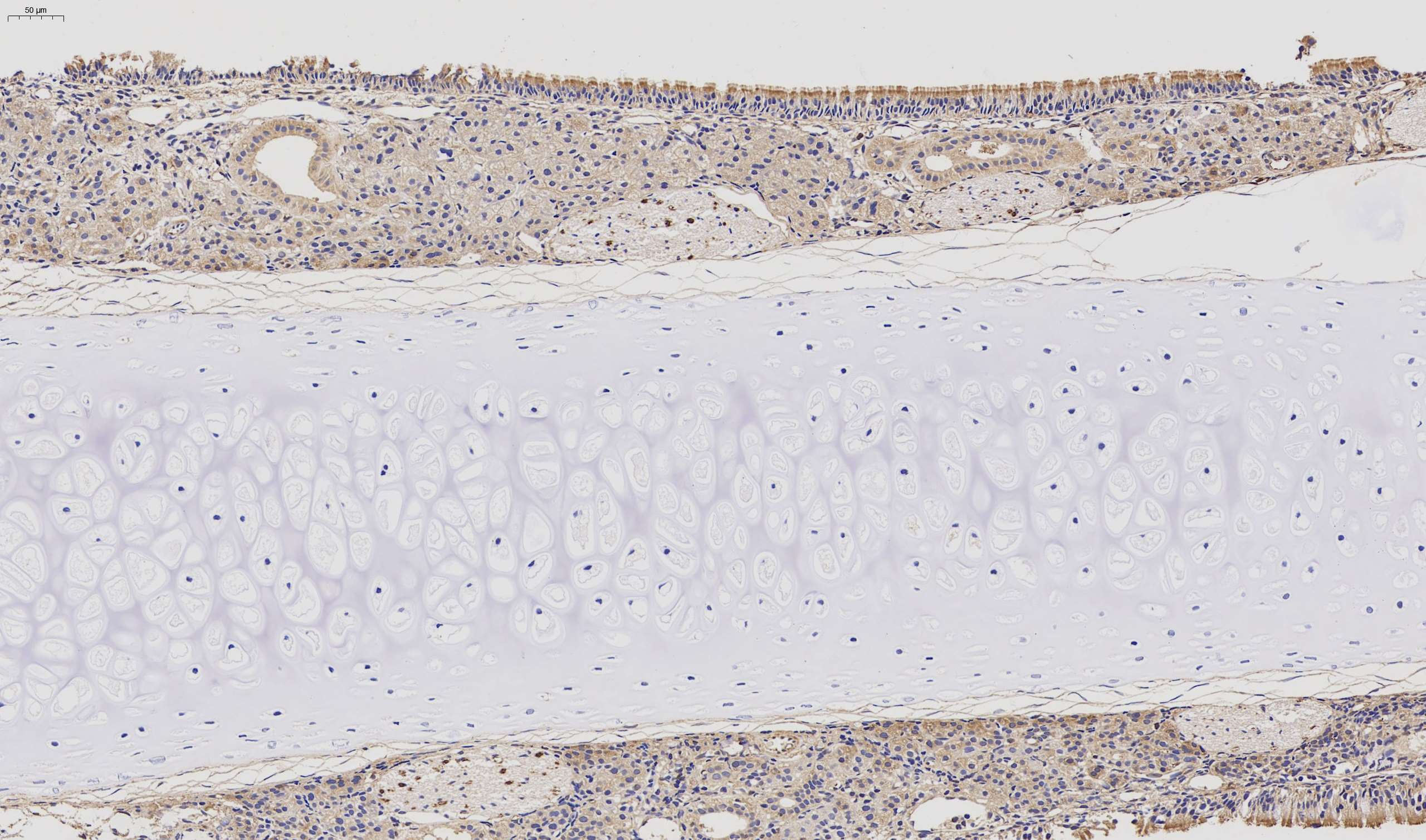

Supplement: Supplementary file 4 [file DataSheet6.ZIP › Microscopy images-Immunohistochemistry-T-bet_200x_50um/CAVO-H/3 T-bet_200x_50um_1.jpeg]

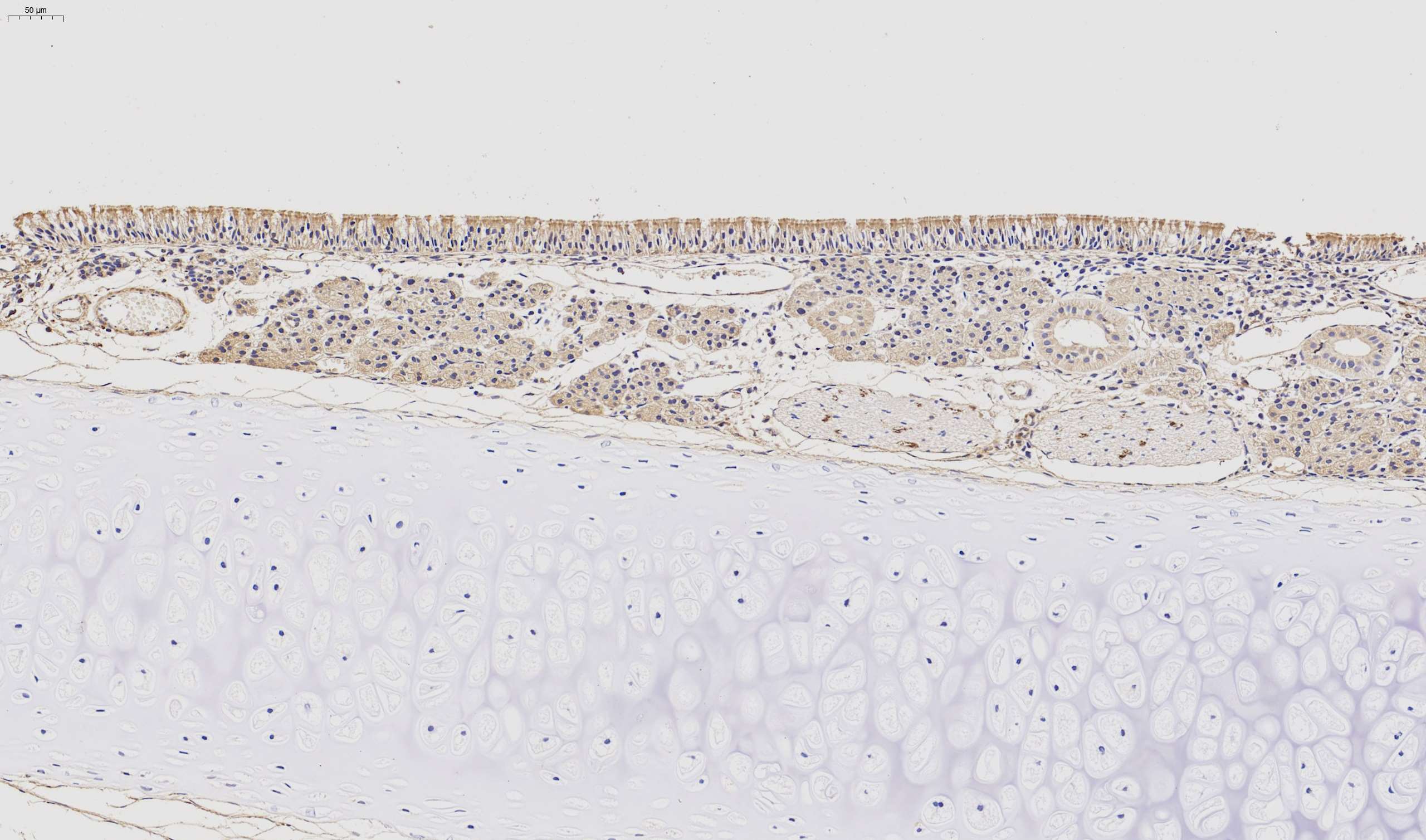

Supplement: Supplementary file 4 [file DataSheet6.ZIP › Microscopy images-Immunohistochemistry-T-bet_200x_50um/CAVO-H/4 T-bet_200x_50um_1.jpeg]

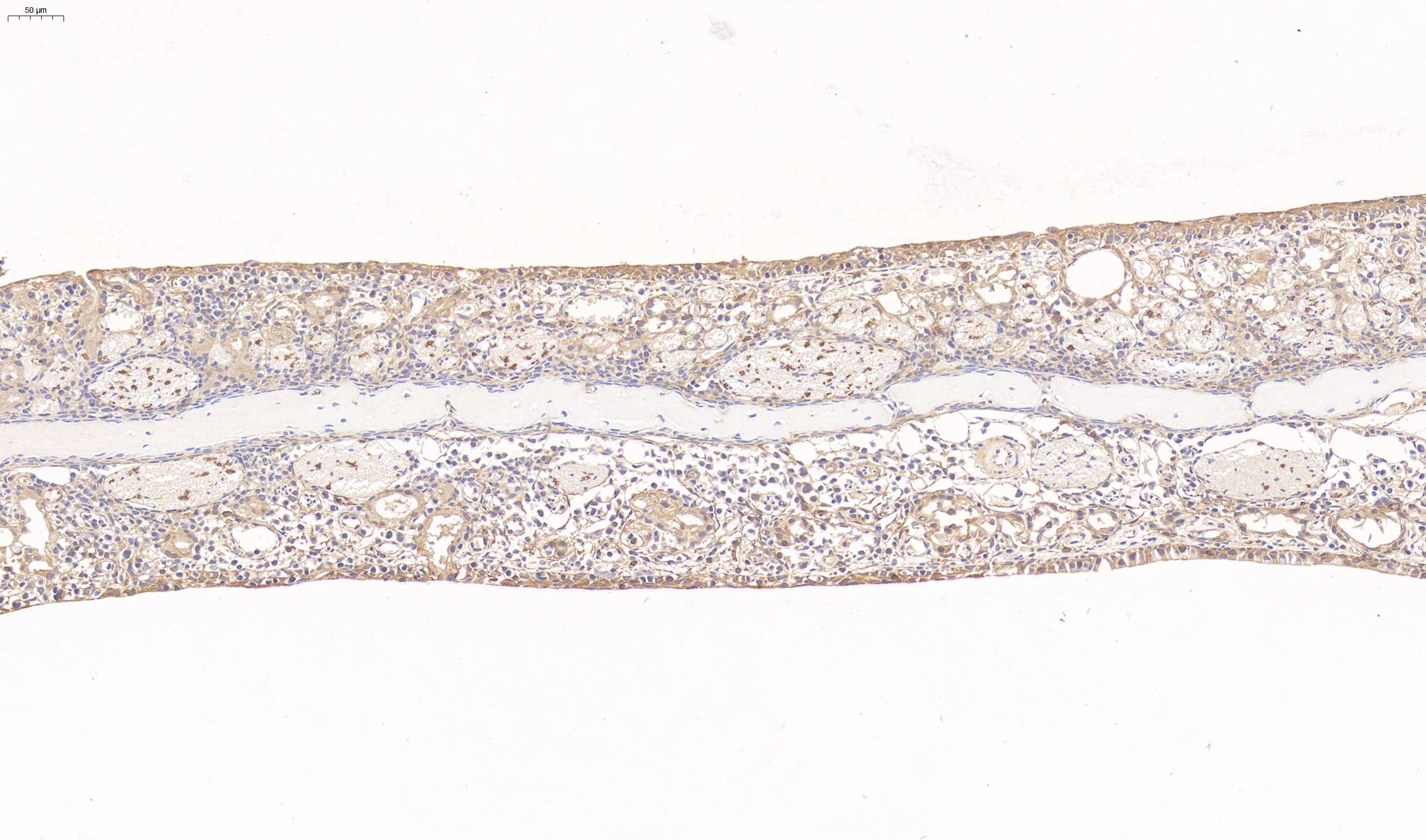

Supplement: Supplementary file 4 [file DataSheet6.ZIP › Microscopy images-Immunohistochemistry-T-bet_200x_50um/CAVO-H/5 T-bet_200x_50um_1.jpeg]

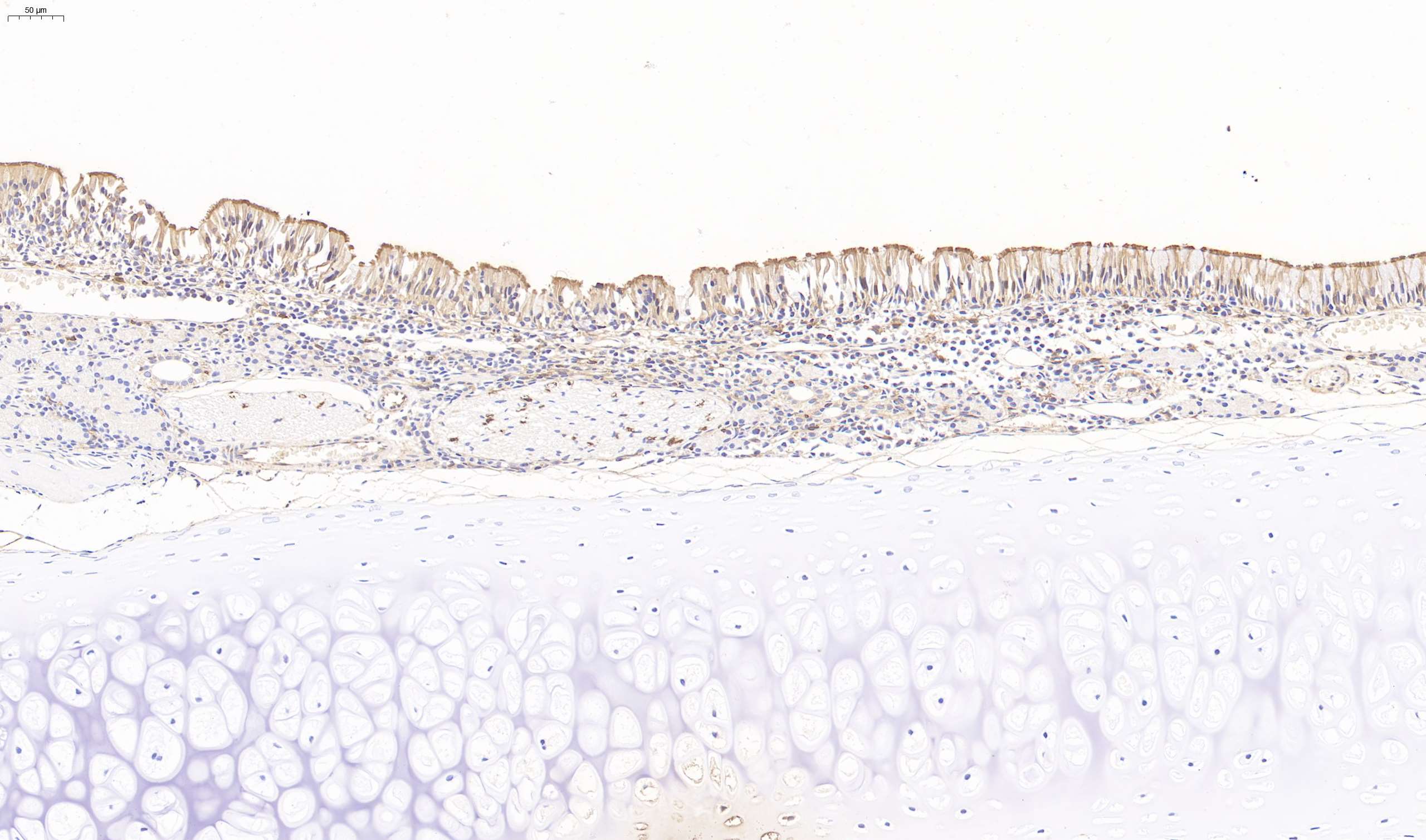

Supplement: Supplementary file 4 [file DataSheet6.ZIP › Microscopy images-Immunohistochemistry-T-bet_200x_50um/CAVO-L/1 T-bet_200x_50um_1.jpeg]

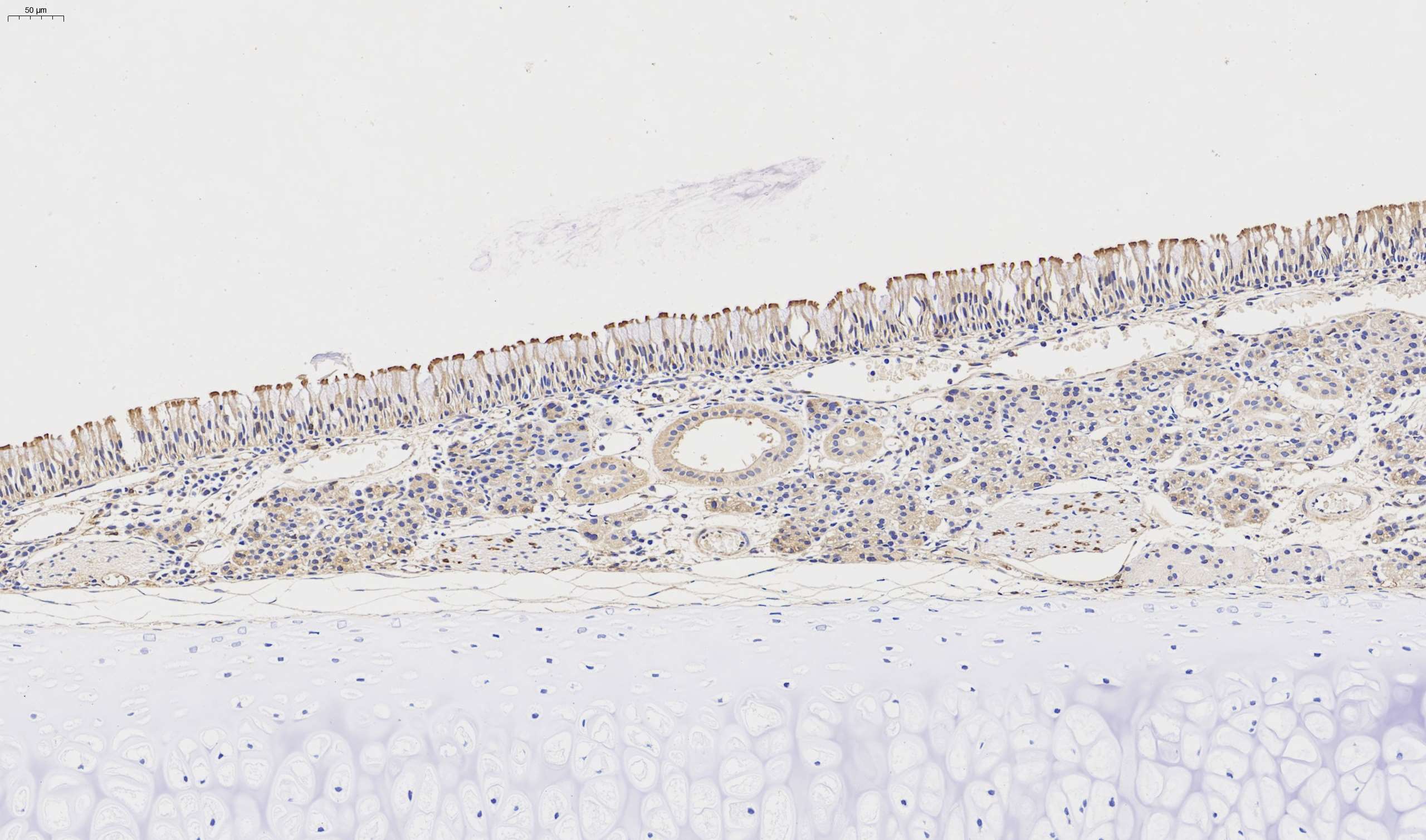

Supplement: Supplementary file 4 [file DataSheet6.ZIP › Microscopy images-Immunohistochemistry-T-bet_200x_50um/CAVO-L/2 T-bet_200x_50um_1.jpeg]

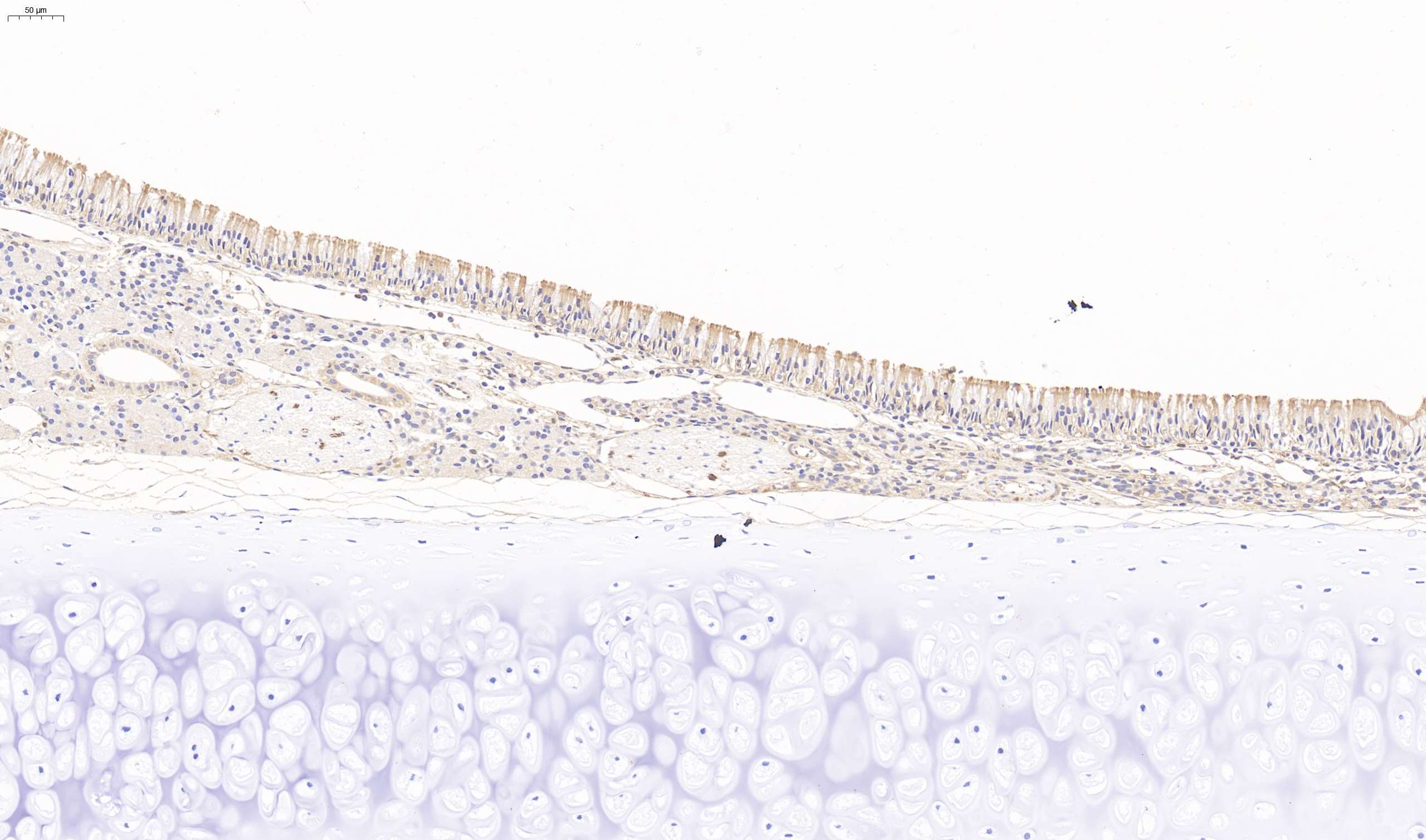

Supplement: Supplementary file 4 [file DataSheet6.ZIP › Microscopy images-Immunohistochemistry-T-bet_200x_50um/CAVO-L/3 T-bet_200x_50um_1.jpeg]

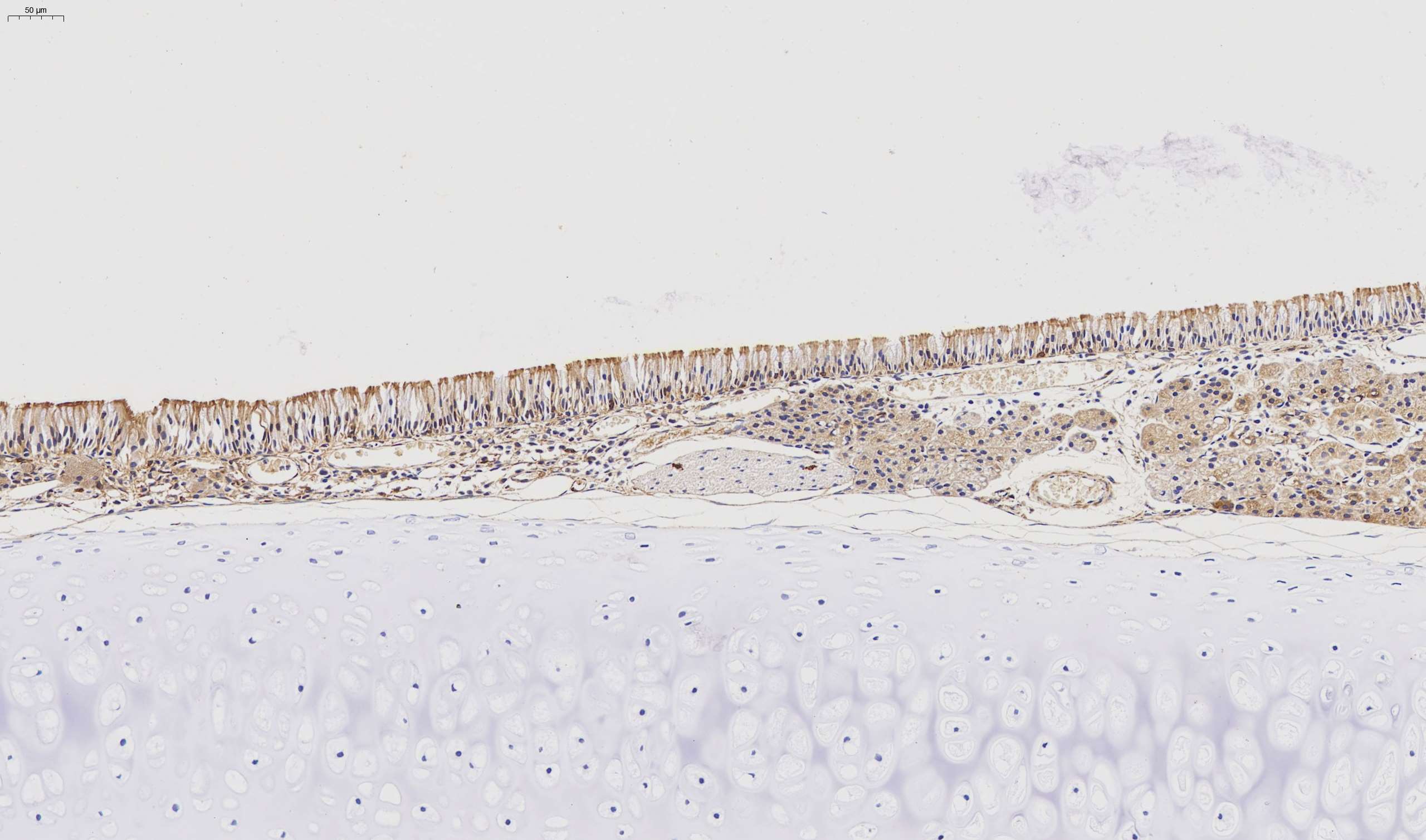

Supplement: Supplementary file 4 [file DataSheet6.ZIP › Microscopy images-Immunohistochemistry-T-bet_200x_50um/CAVO-L/4 T-bet_200x_50um_1.jpeg]

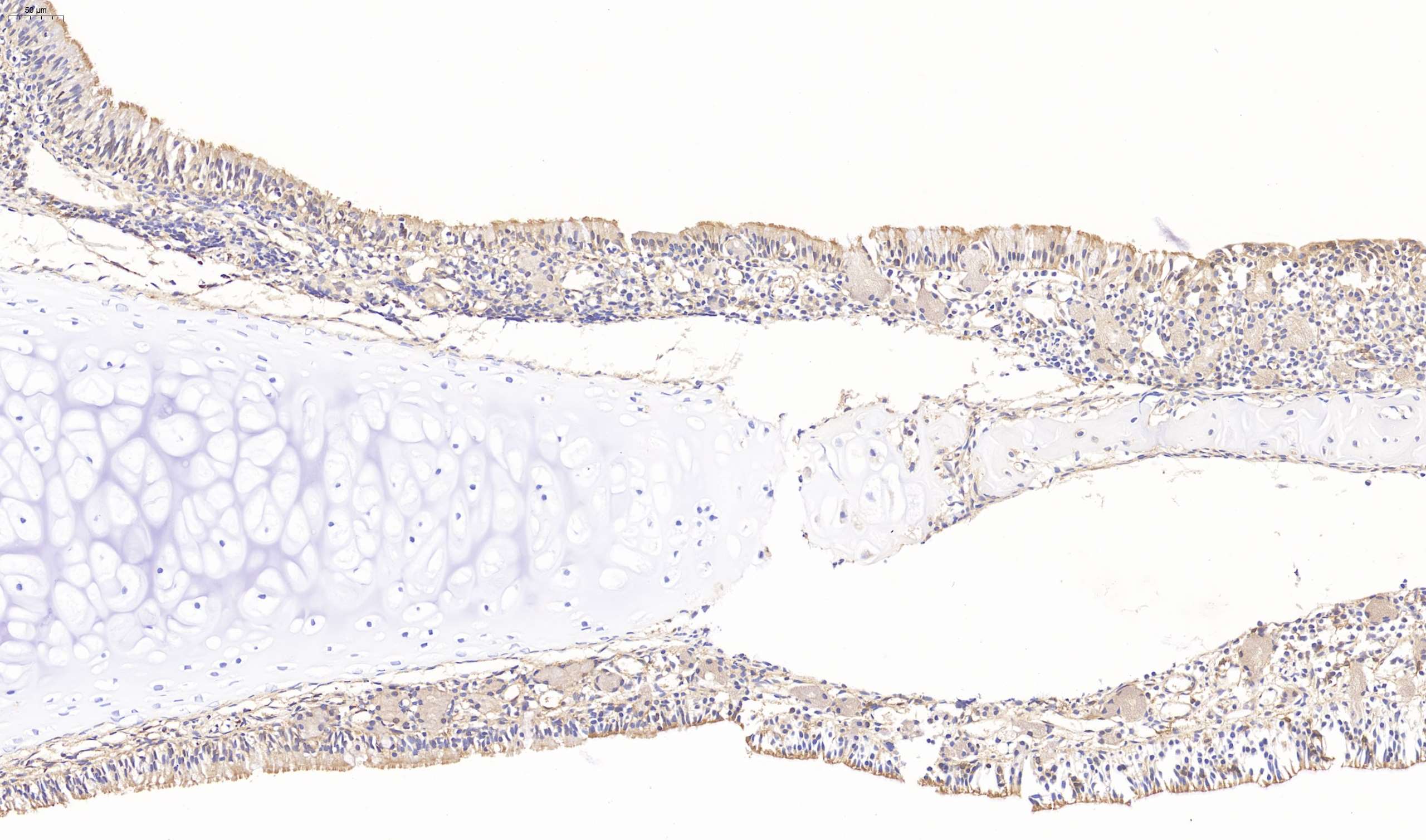

Supplement: Supplementary file 4 [file DataSheet6.ZIP › Microscopy images-Immunohistochemistry-T-bet_200x_50um/CAVO-L/5 T-bet_200x_50um_1.jpeg]

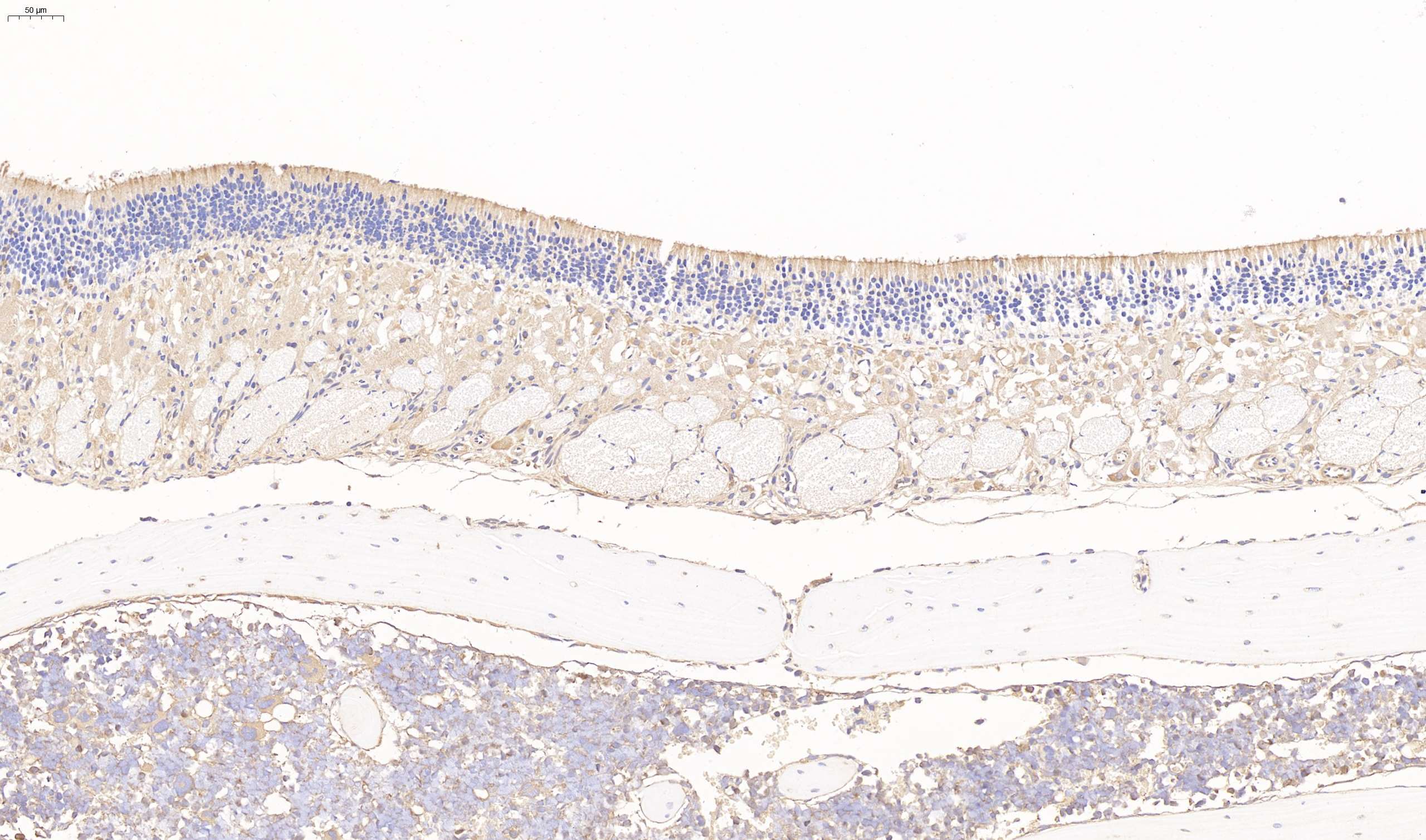

Supplement: Supplementary file 4 [file DataSheet6.ZIP › Microscopy images-Immunohistochemistry-T-bet_200x_50um/Control/1 T-bet_200x_1.jpeg]

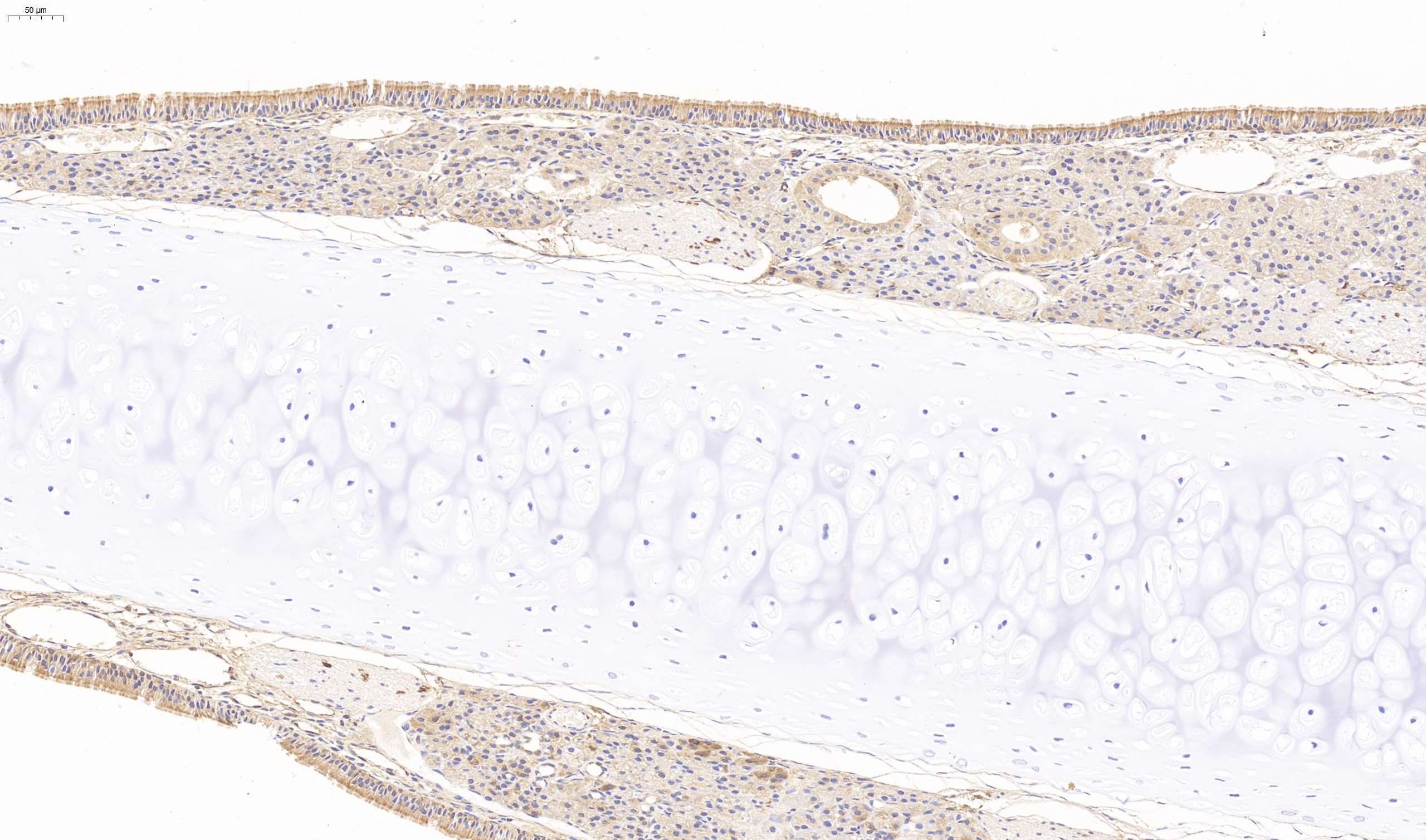

Supplement: Supplementary file 4 [file DataSheet6.ZIP › Microscopy images-Immunohistochemistry-T-bet_200x_50um/Control/2 T-bet_200x_50um_1.jpeg]

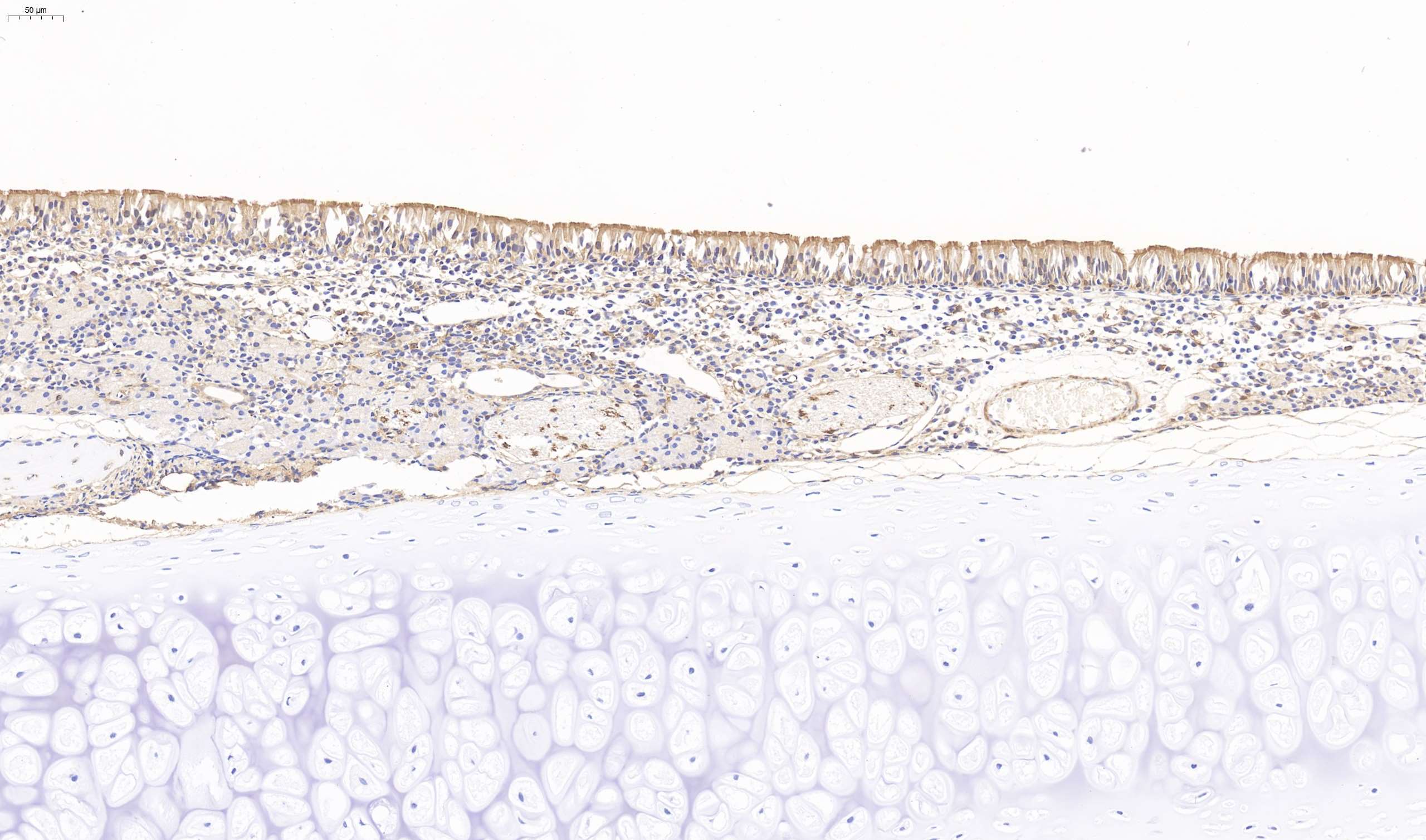

Supplement: Supplementary file 4 [file DataSheet6.ZIP › Microscopy images-Immunohistochemistry-T-bet_200x_50um/Control/3 T-bet_200x_50um_1.jpeg]

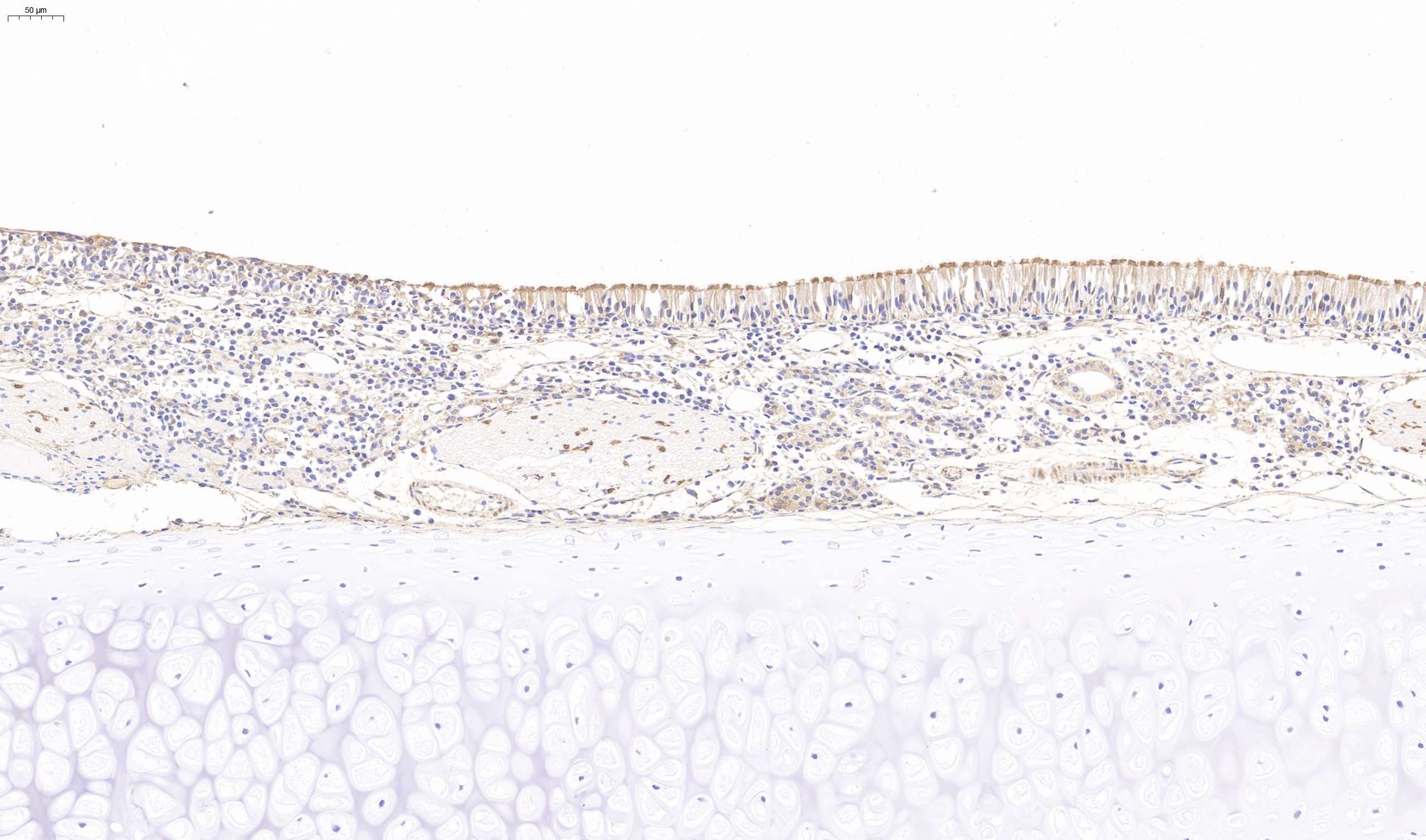

Supplement: Supplementary file 4 [file DataSheet6.ZIP › Microscopy images-Immunohistochemistry-T-bet_200x_50um/Control/4 T-bet_200x_50um_1.jpeg]

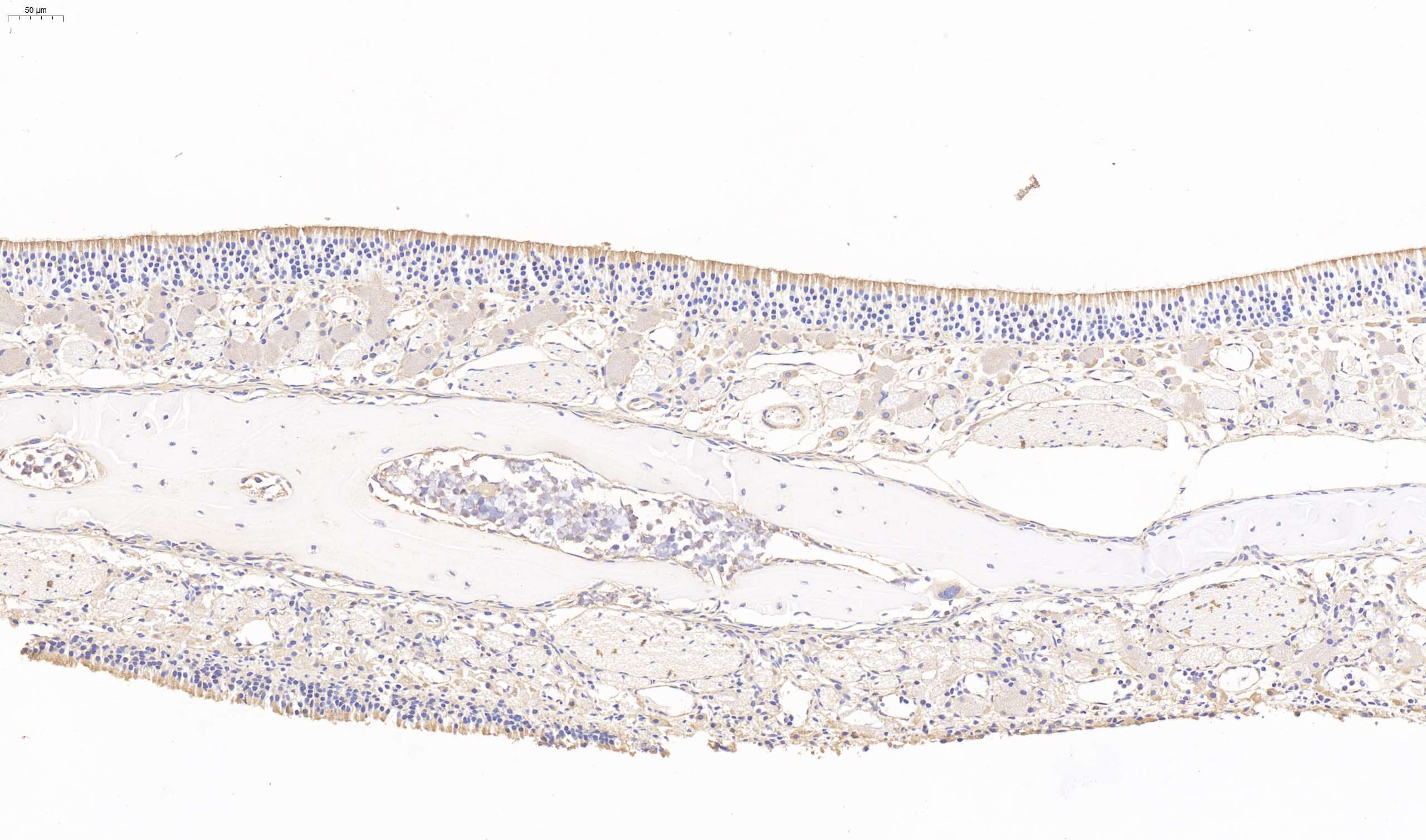

Supplement: Supplementary file 4 [file DataSheet6.ZIP › Microscopy images-Immunohistochemistry-T-bet_200x_50um/Control/5 T-bet_200x_50um_1.jpeg]

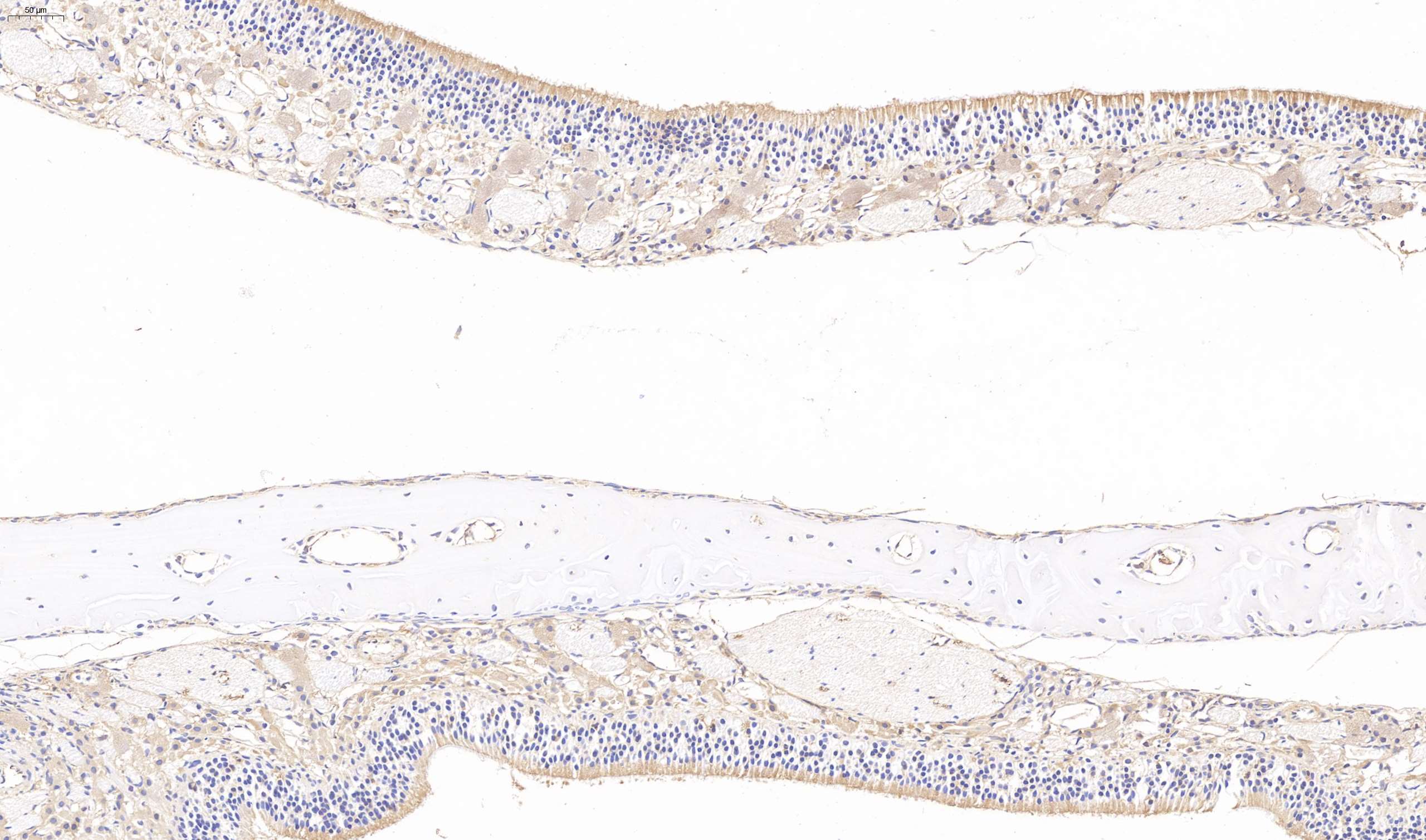

Supplement: Supplementary file 4 [file DataSheet6.ZIP › Microscopy images-Immunohistochemistry-T-bet_200x_50um/Loratadine/1 T-bet_200x_50um_1.jpeg]

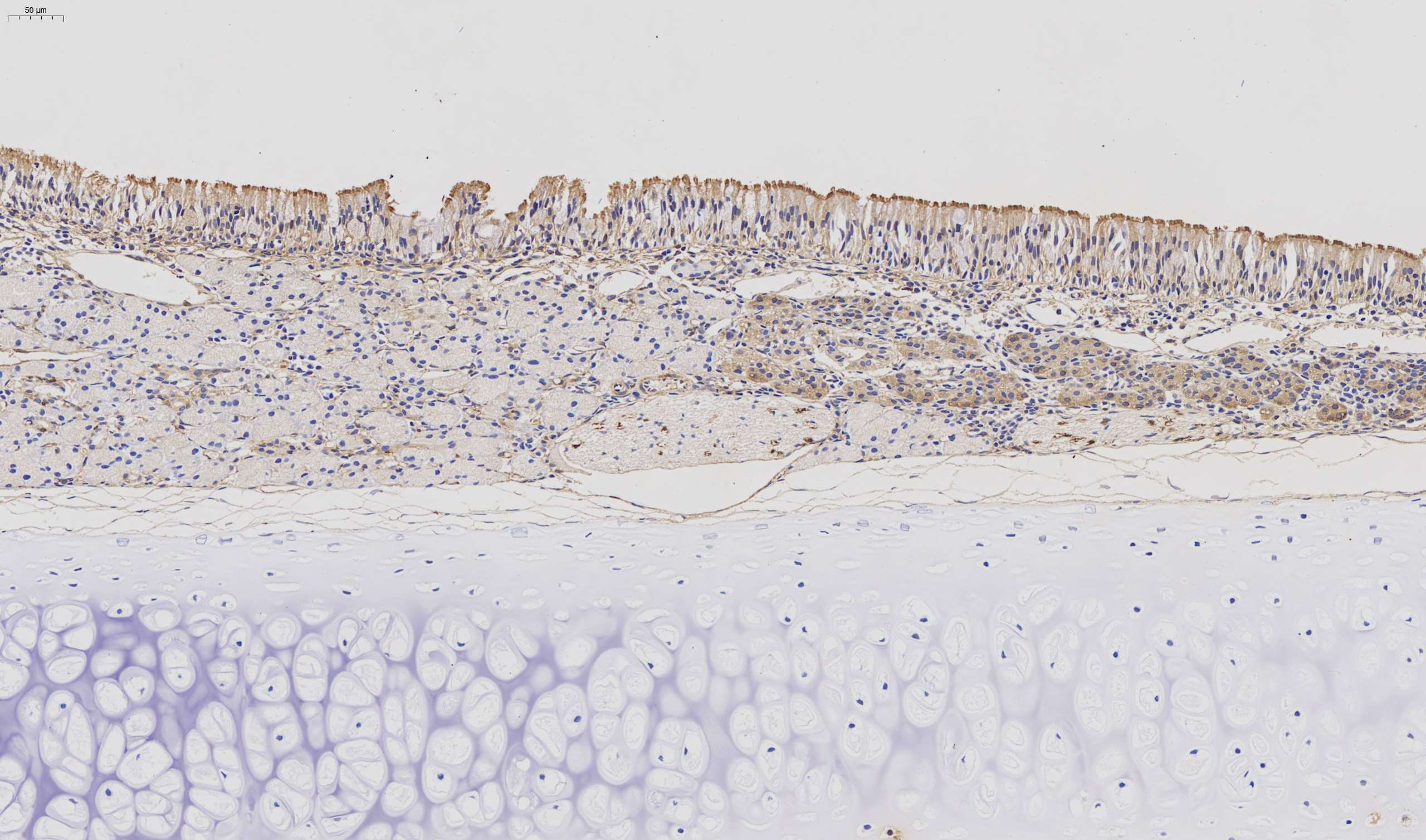

Supplement: Supplementary file 4 [file DataSheet6.ZIP › Microscopy images-Immunohistochemistry-T-bet_200x_50um/Loratadine/2 T-bet_200x_50um_1.jpeg]

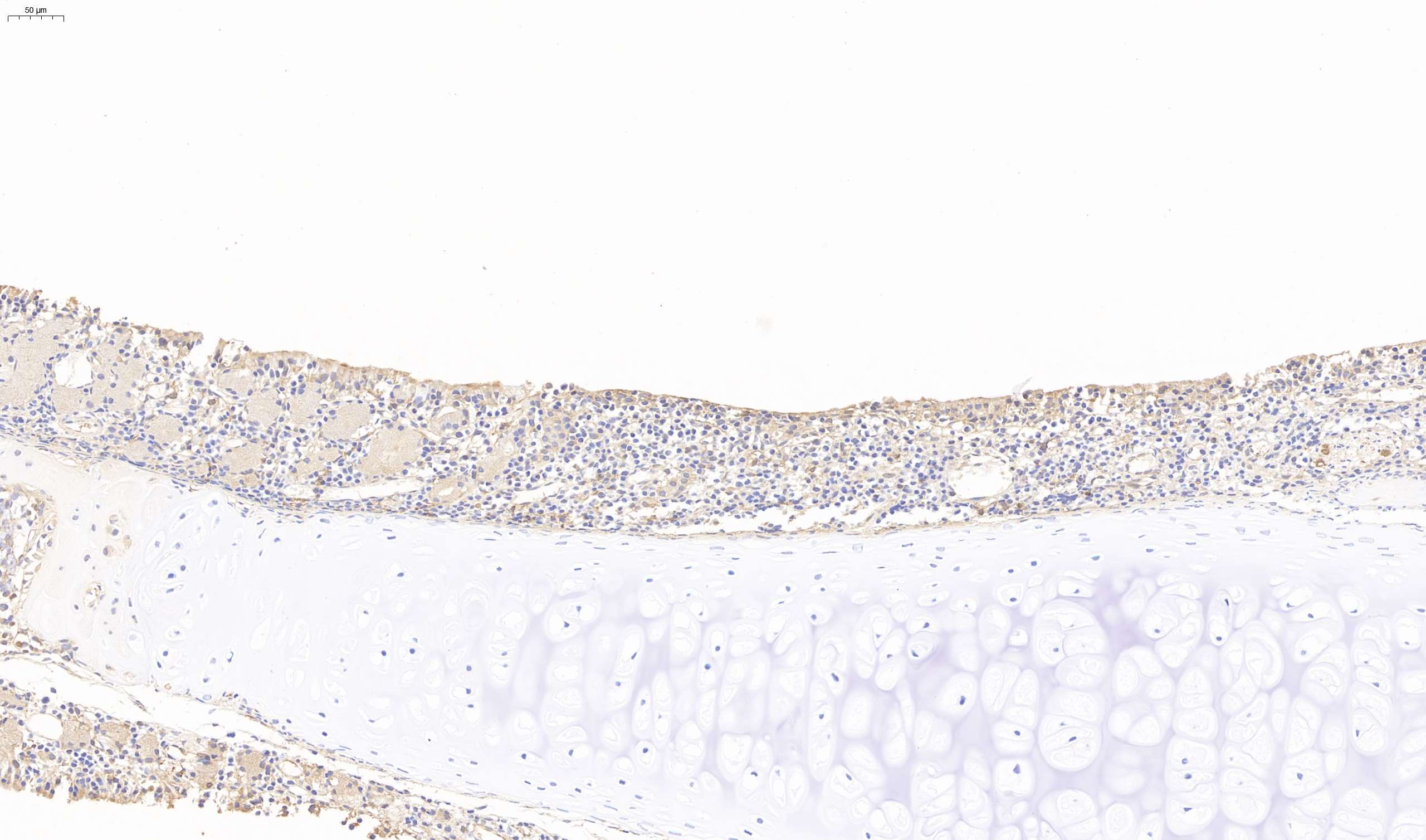

Supplement: Supplementary file 4 [file DataSheet6.ZIP › Microscopy images-Immunohistochemistry-T-bet_200x_50um/Loratadine/3 T-bet_200x_50um_1.jpeg]

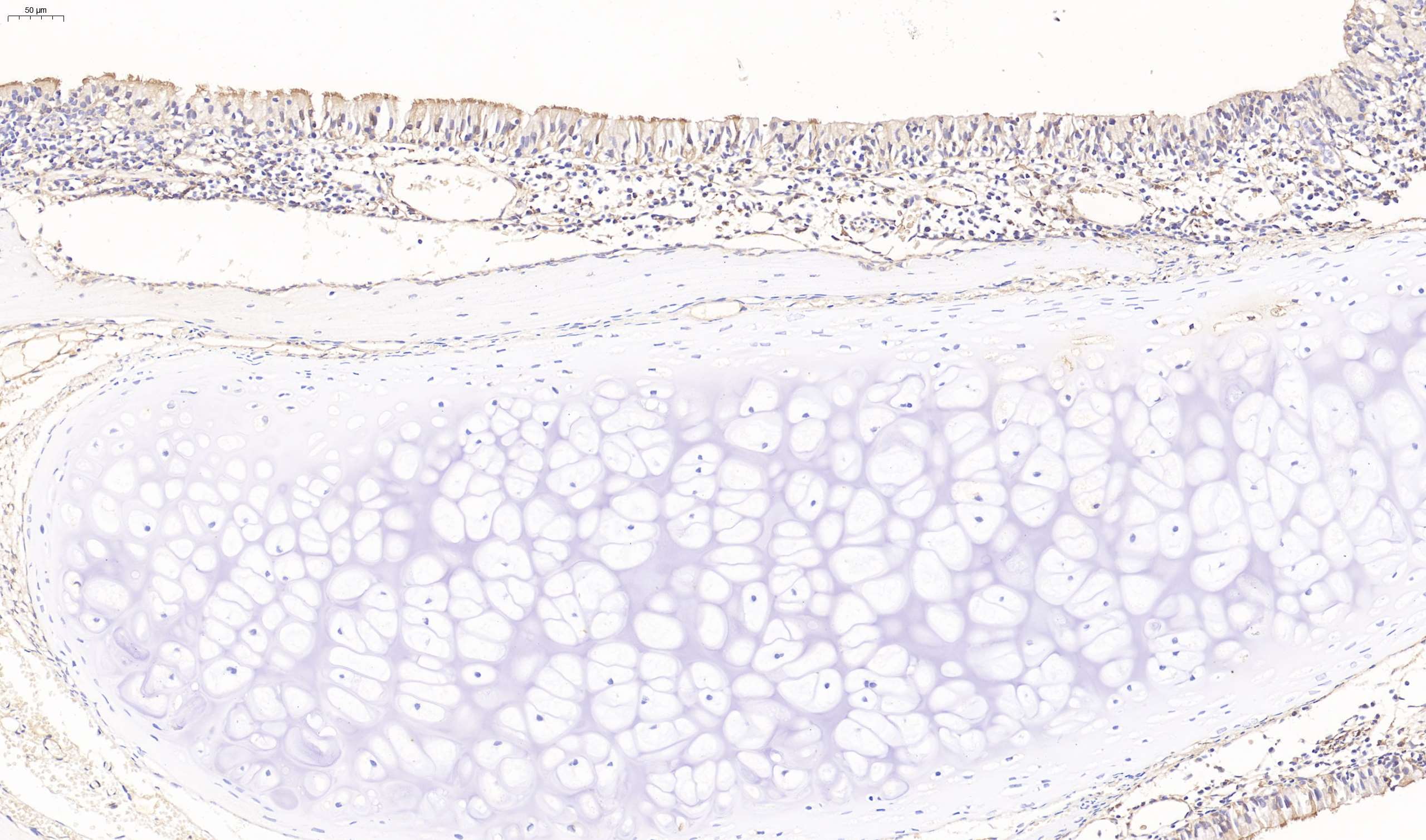

Supplement: Supplementary file 4 [file DataSheet6.ZIP › Microscopy images-Immunohistochemistry-T-bet_200x_50um/Loratadine/4 T-bet_200x_50um_1.jpeg]

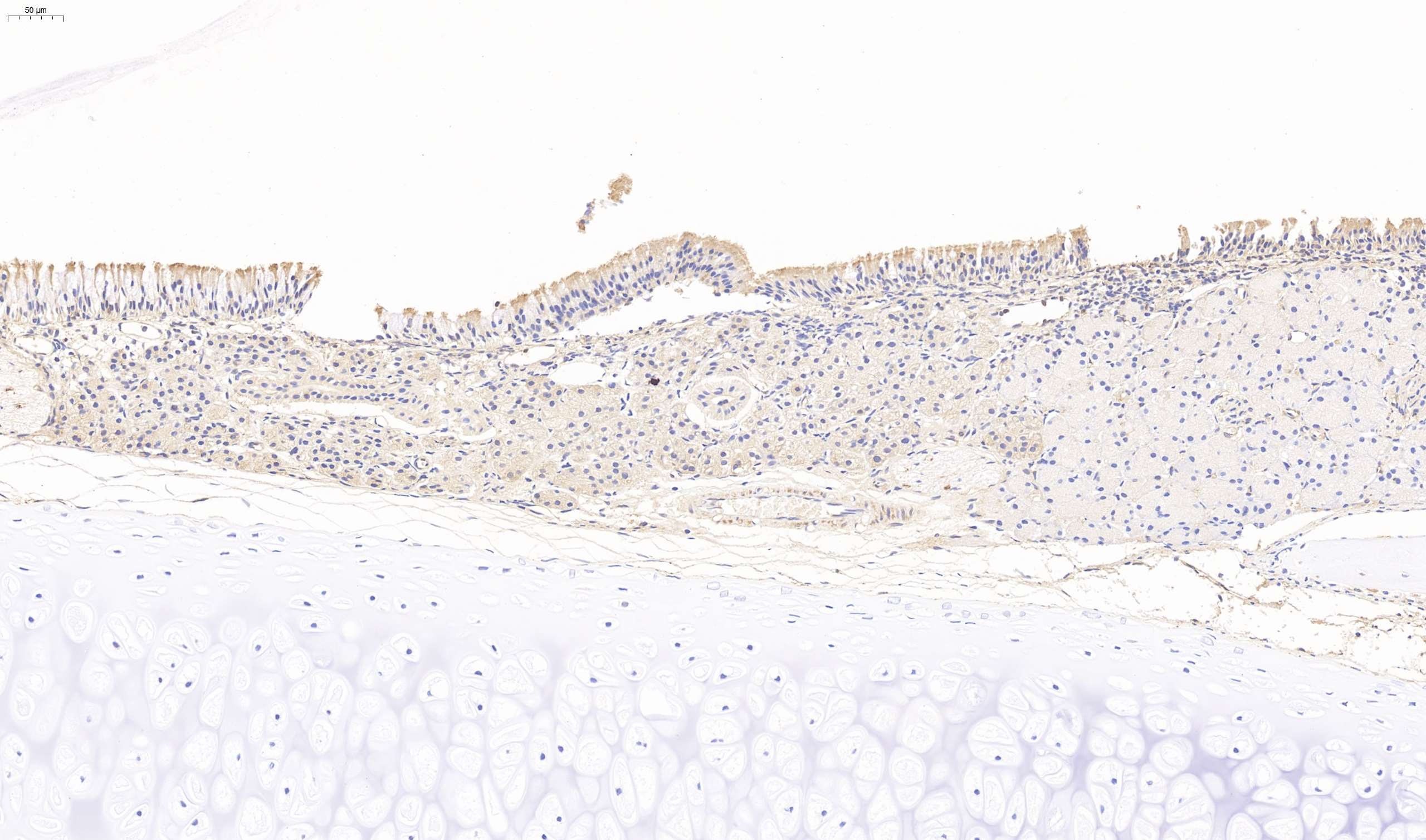

Supplement: Supplementary file 4 [file DataSheet6.ZIP › Microscopy images-Immunohistochemistry-T-bet_200x_50um/Loratadine/5 T-bet_200x_50um_1.jpeg]

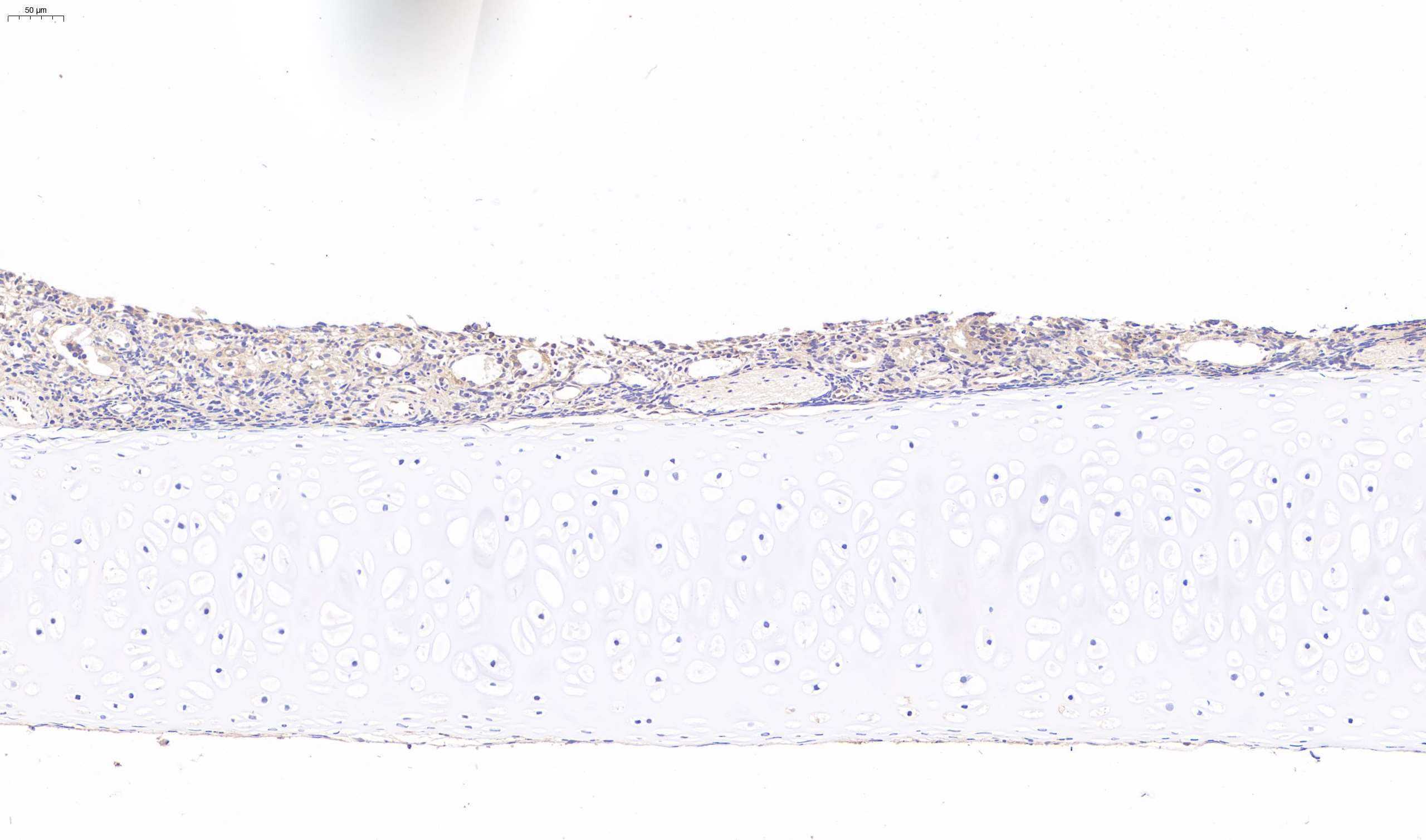

Supplement: Supplementary file 4 [file DataSheet6.ZIP › Microscopy images-Immunohistochemistry-T-bet_200x_50um/Model/1 T-bet_200x_50um_1.jpeg]

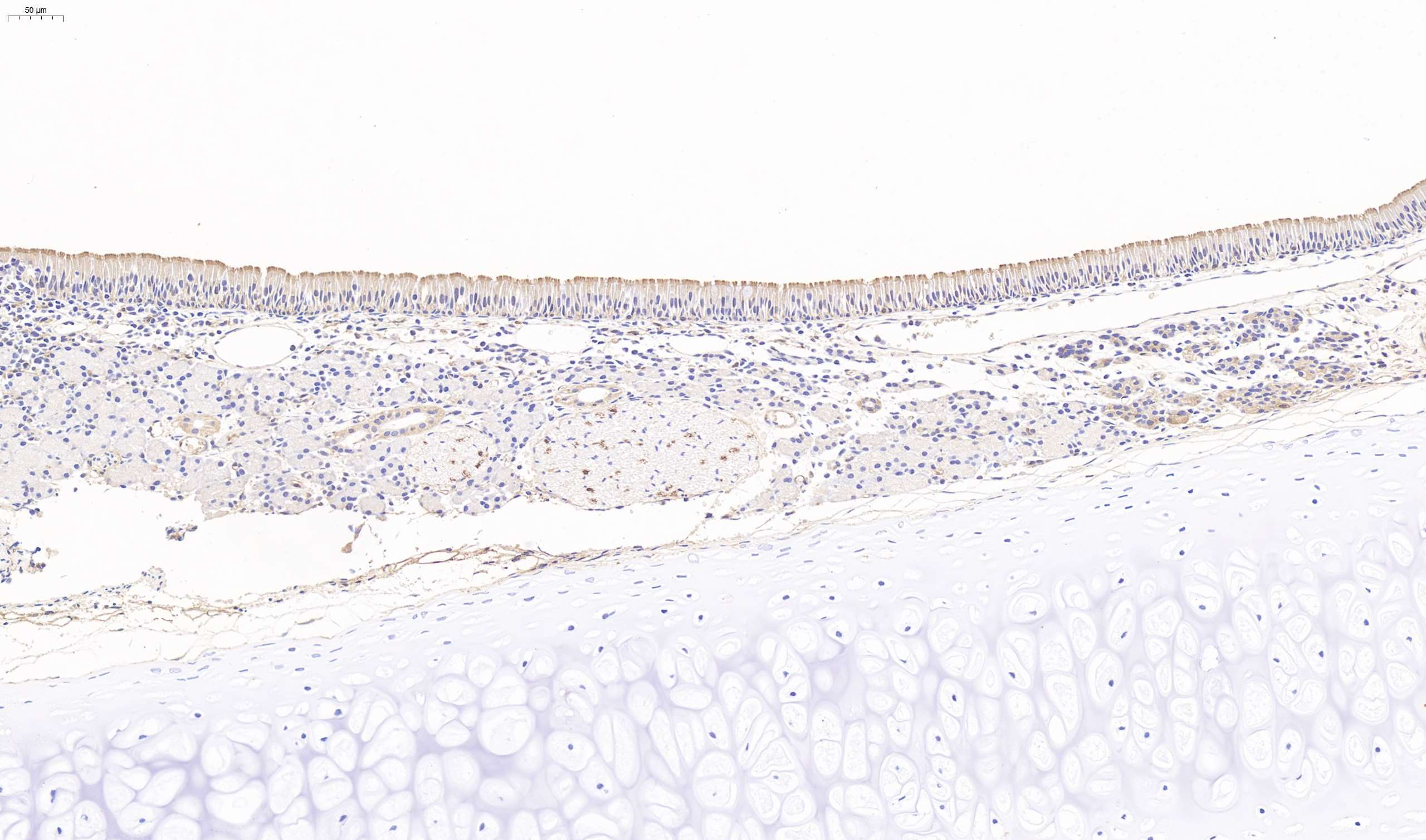

Supplement: Supplementary file 4 [file DataSheet6.ZIP › Microscopy images-Immunohistochemistry-T-bet_200x_50um/Model/2 T-bet_200x_50um_1.jpeg]

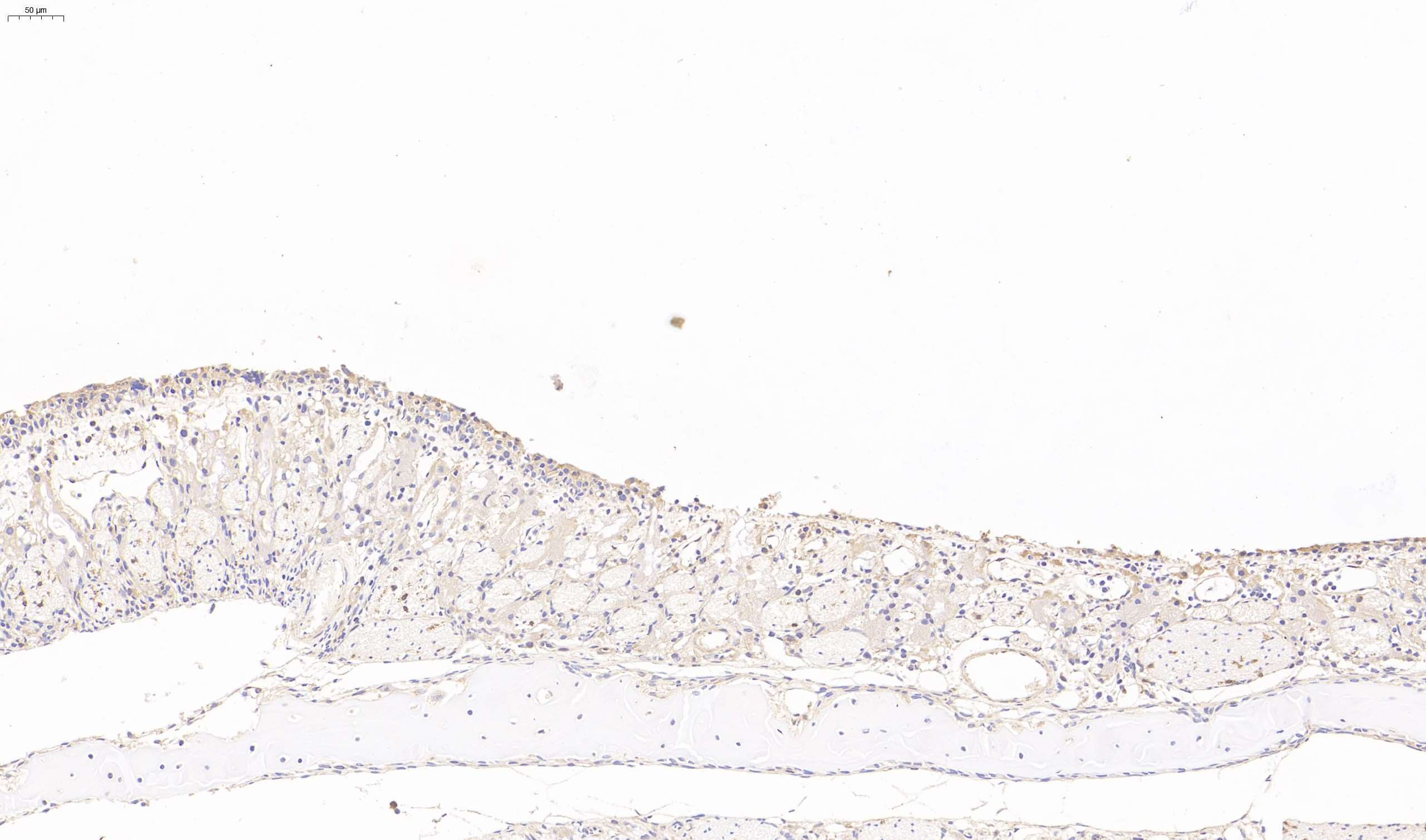

Supplement: Supplementary file 4 [file DataSheet6.ZIP › Microscopy images-Immunohistochemistry-T-bet_200x_50um/Model/3 T-bet_200x_50um_1.jpeg]

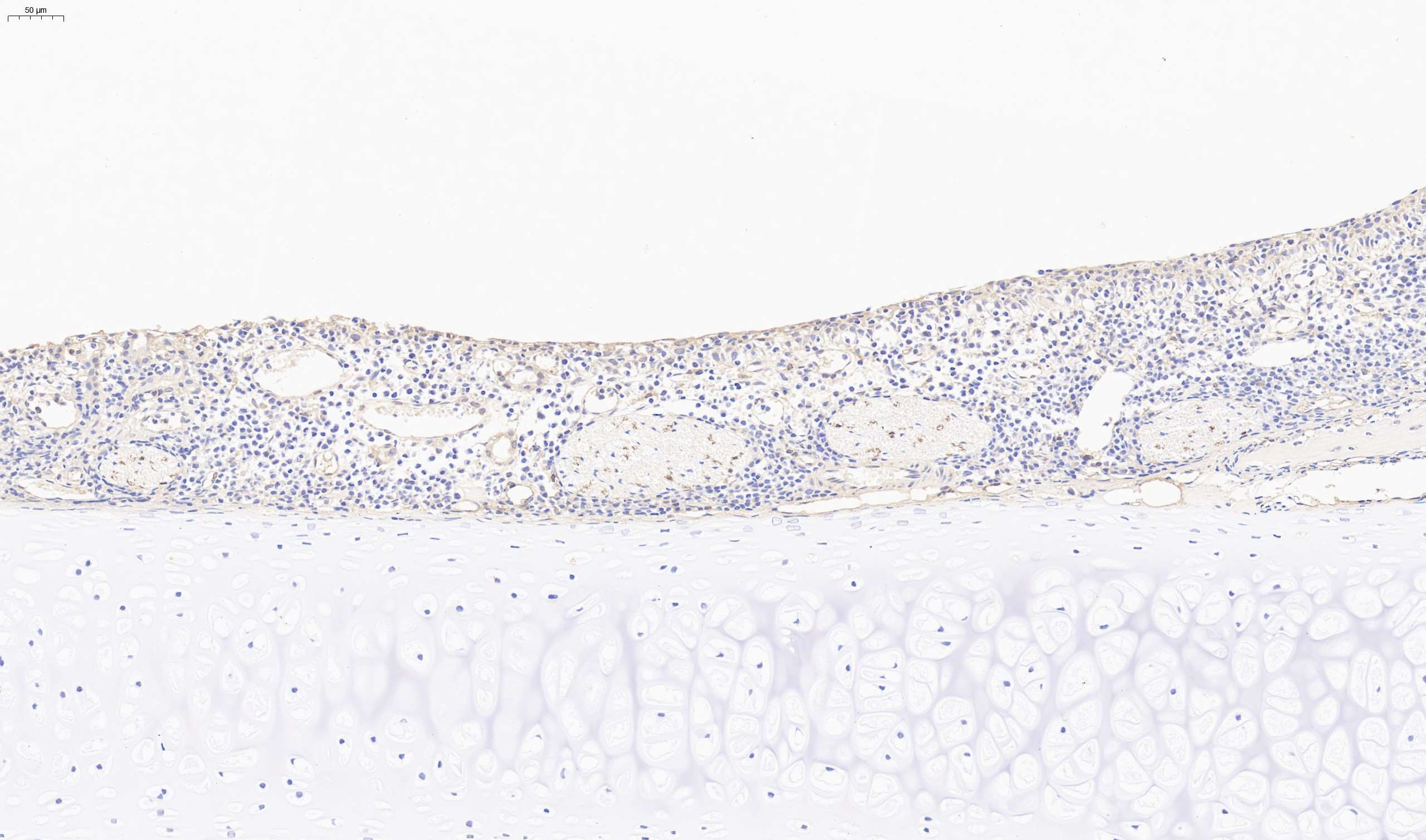

Supplement: Supplementary file 4 [file DataSheet6.ZIP › Microscopy images-Immunohistochemistry-T-bet_200x_50um/Model/4 T-bet_200x_50um_1.jpeg]

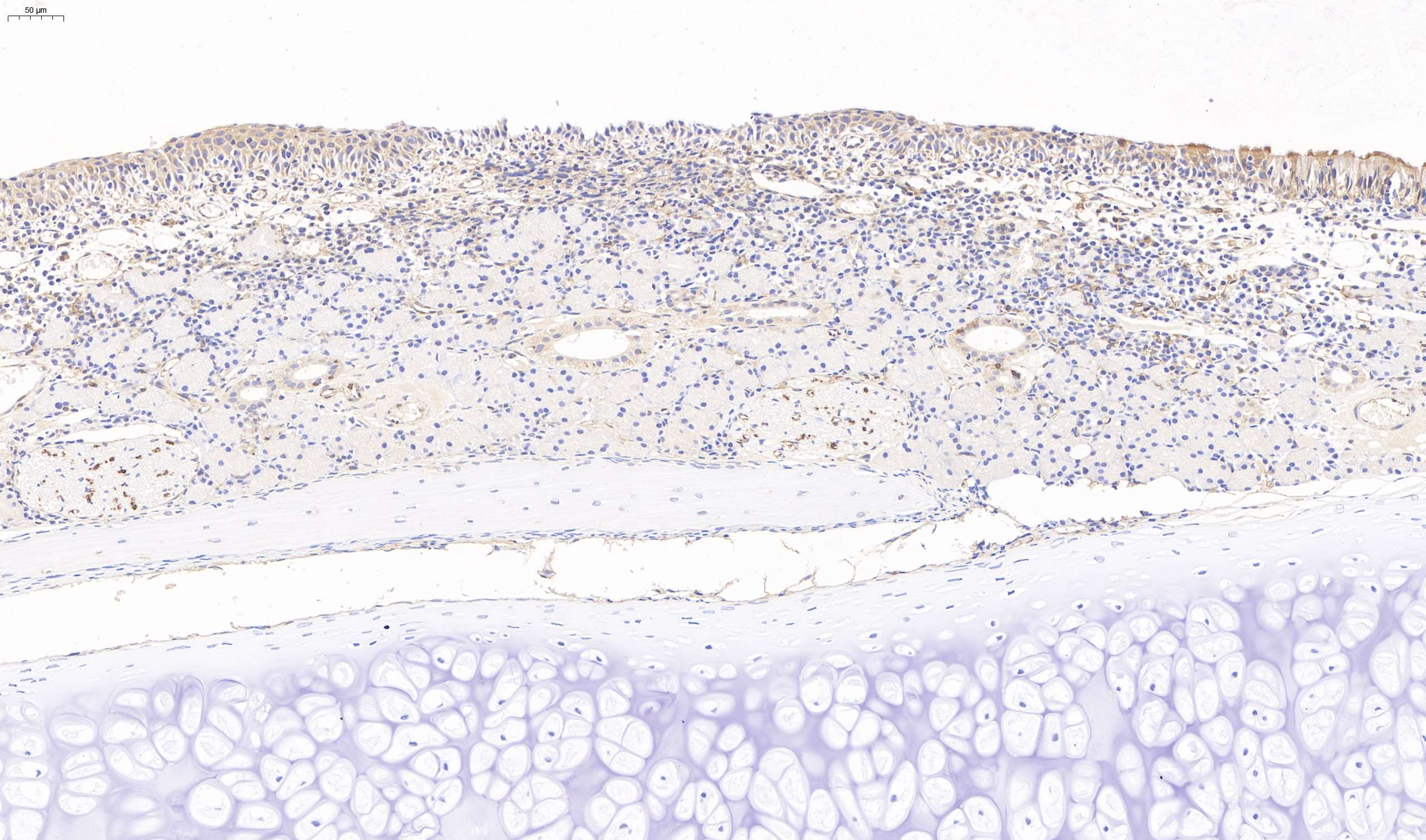

Supplement: Supplementary file 4 [file DataSheet6.ZIP › Microscopy images-Immunohistochemistry-T-bet_200x_50um/Model/5 T-bet_200x_50um_1.jpeg]

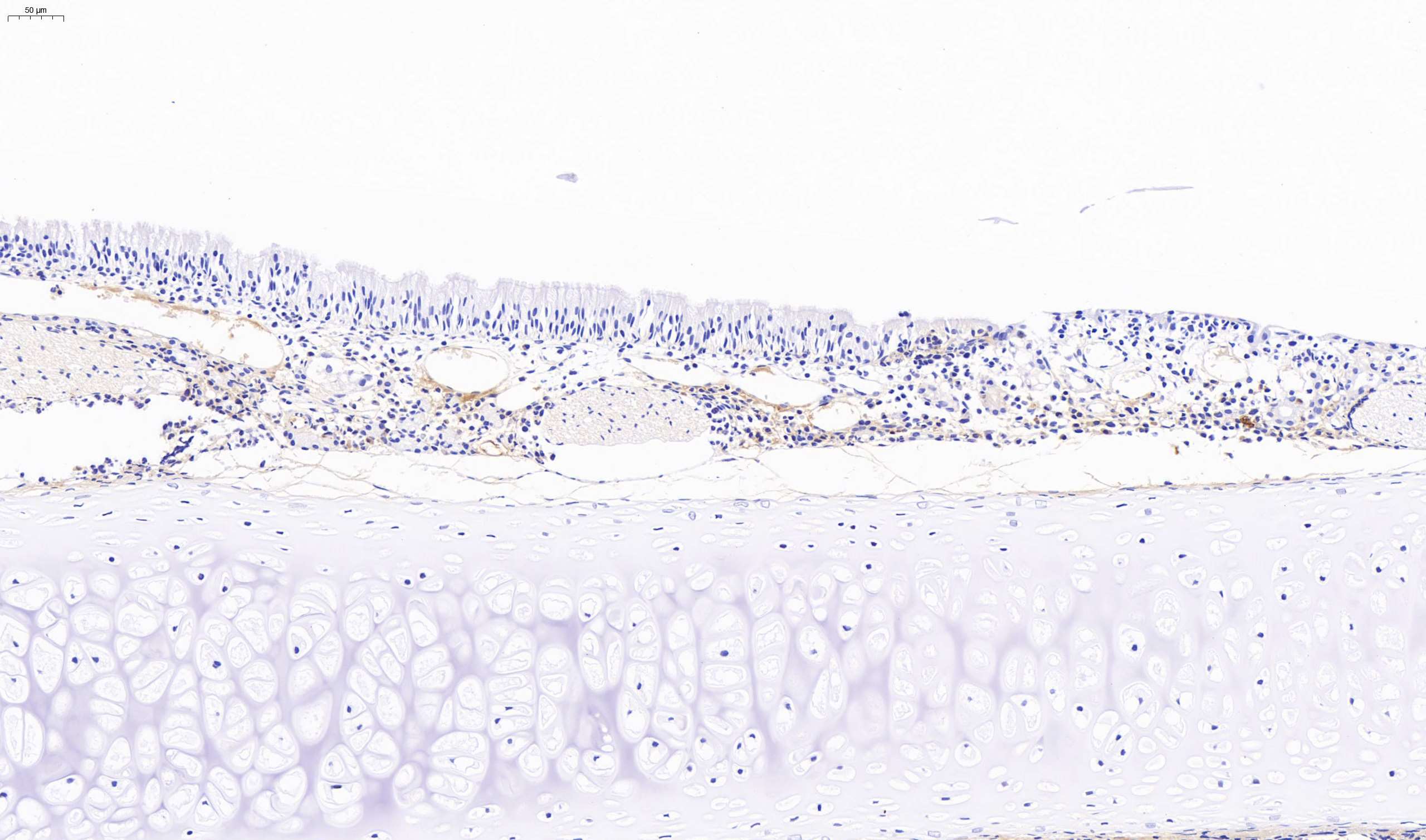

Supplement: Supplementary file 7 [file DataSheet5.ZIP › Microscopy images-Immunohistochemistry-GATA-3_200x_50um/CAVO-H/1 GATA-3_200x_50um_1.jpeg]

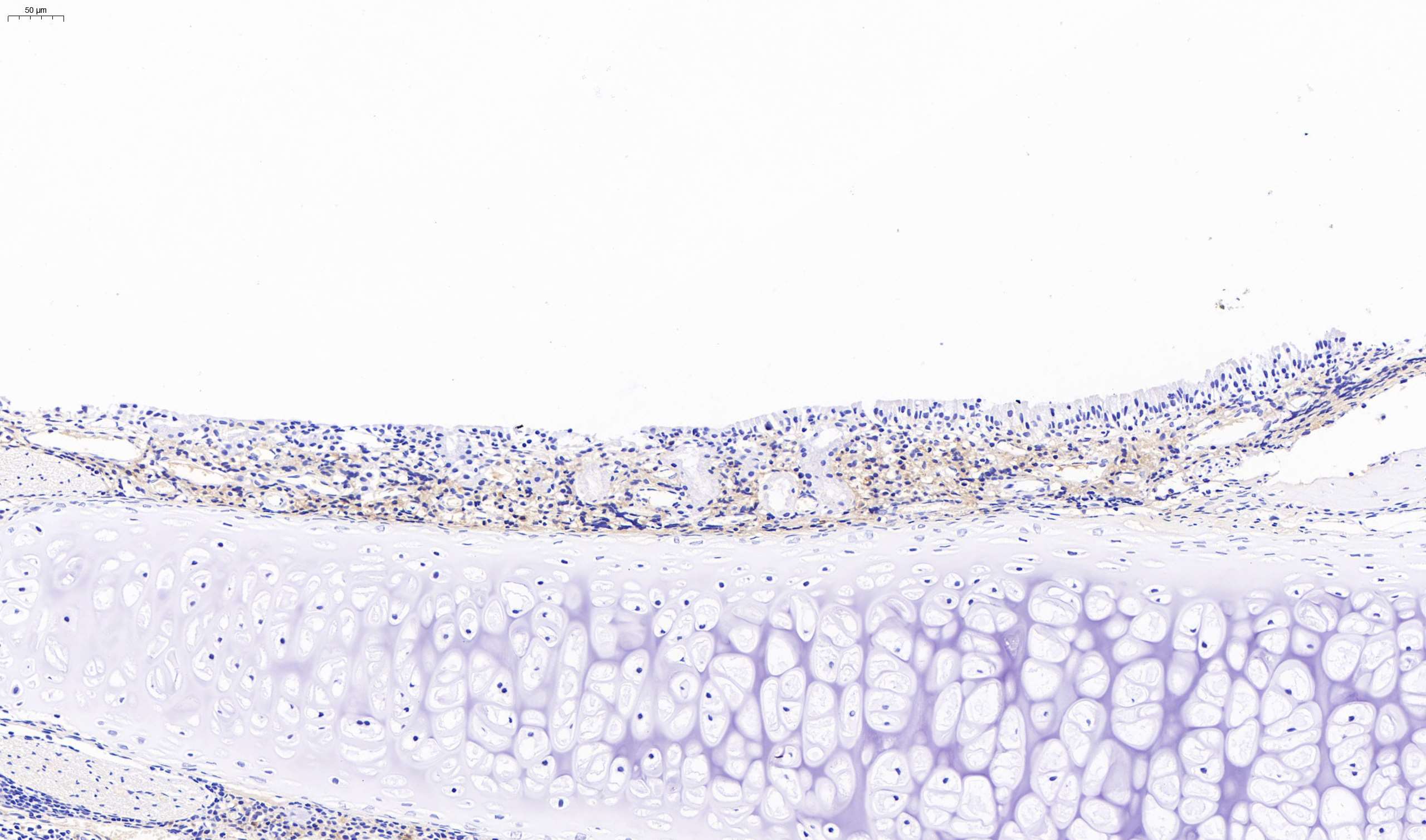

Supplement: Supplementary file 7 [file DataSheet5.ZIP › Microscopy images-Immunohistochemistry-GATA-3_200x_50um/CAVO-H/2 GATA-3_200x_50um_1.jpeg]

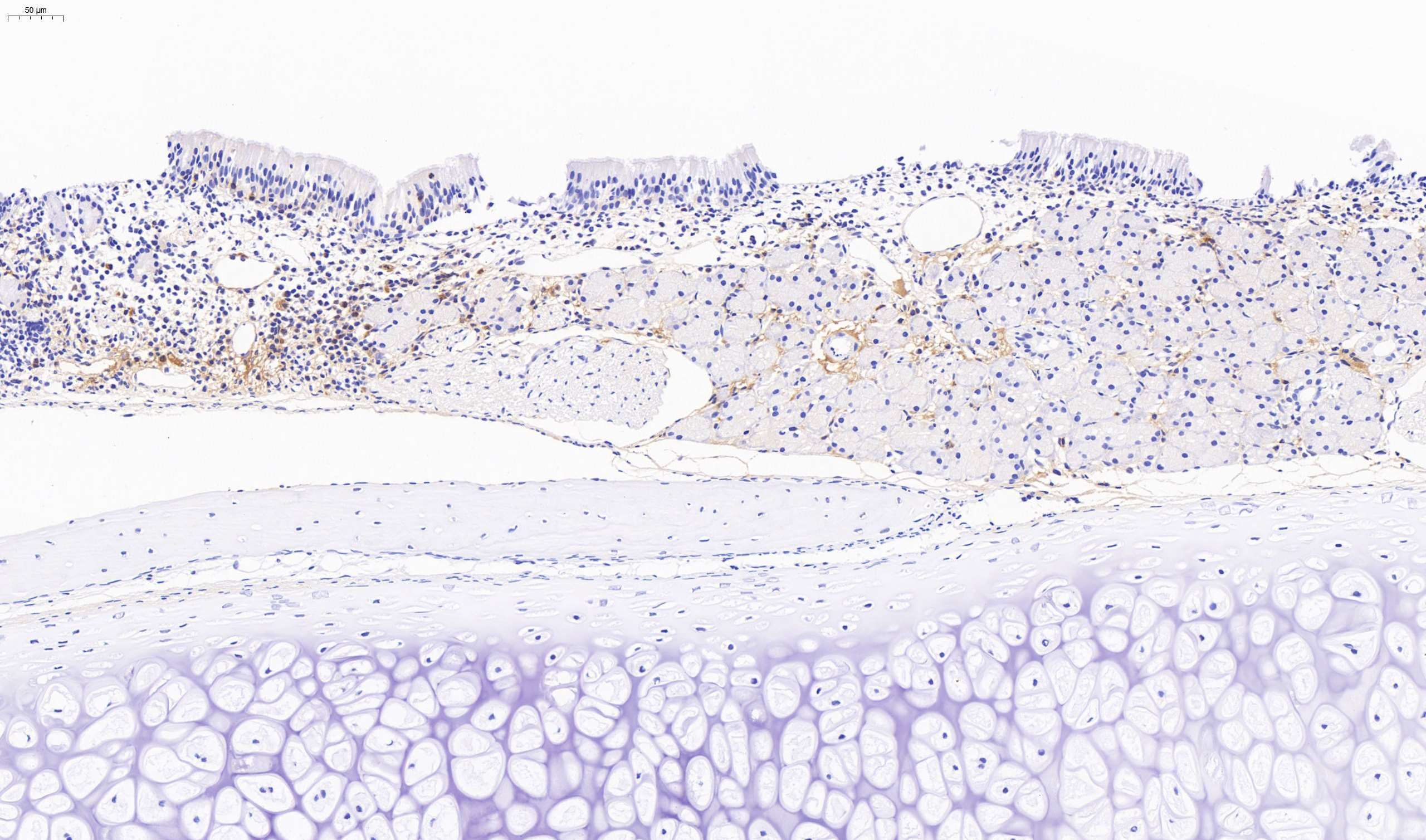

Supplement: Supplementary file 7 [file DataSheet5.ZIP › Microscopy images-Immunohistochemistry-GATA-3_200x_50um/CAVO-H/3 GATA-3_200x_50um_1.jpeg]

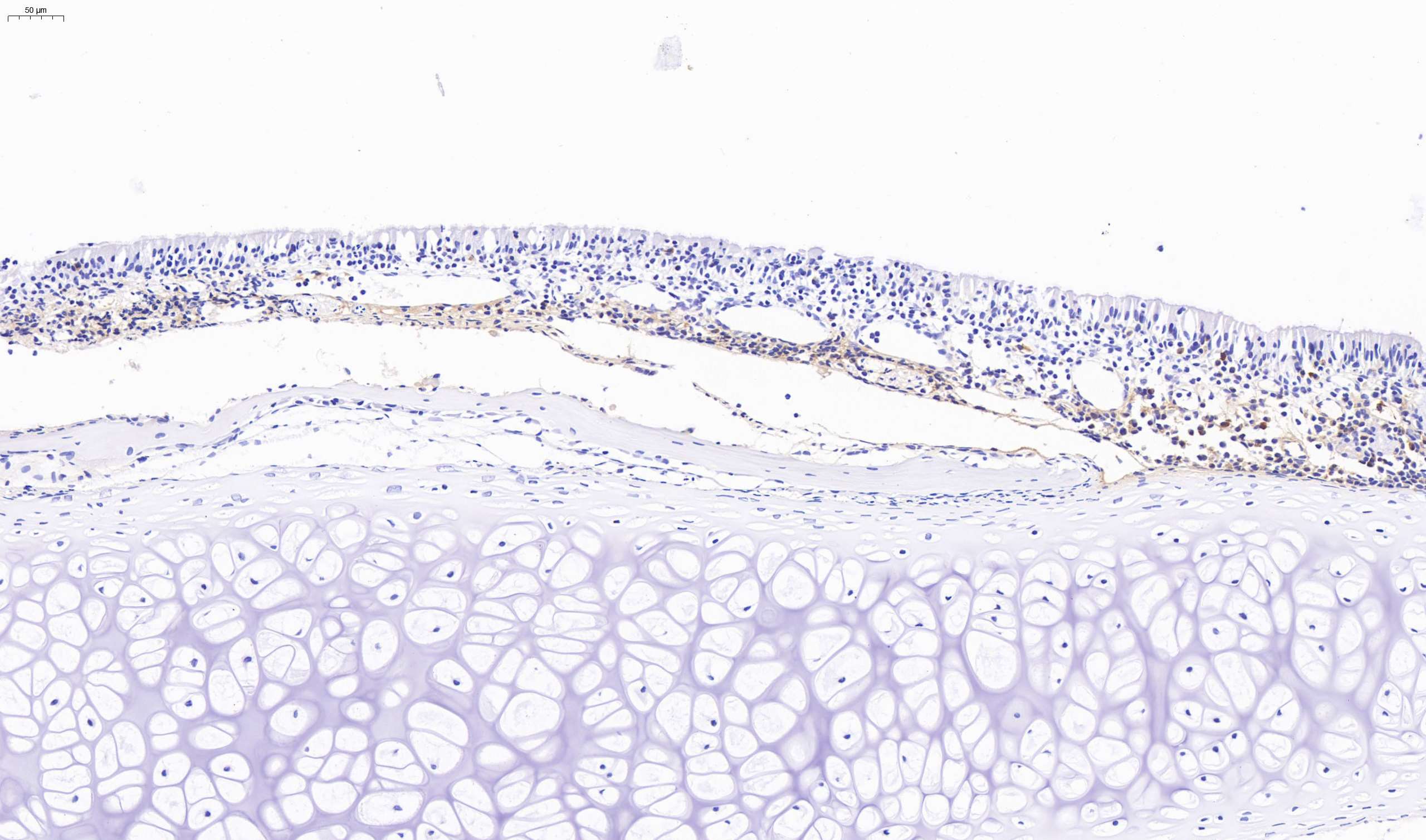

Supplement: Supplementary file 7 [file DataSheet5.ZIP › Microscopy images-Immunohistochemistry-GATA-3_200x_50um/CAVO-H/4 GATA-3_200x_50um_1.jpeg]

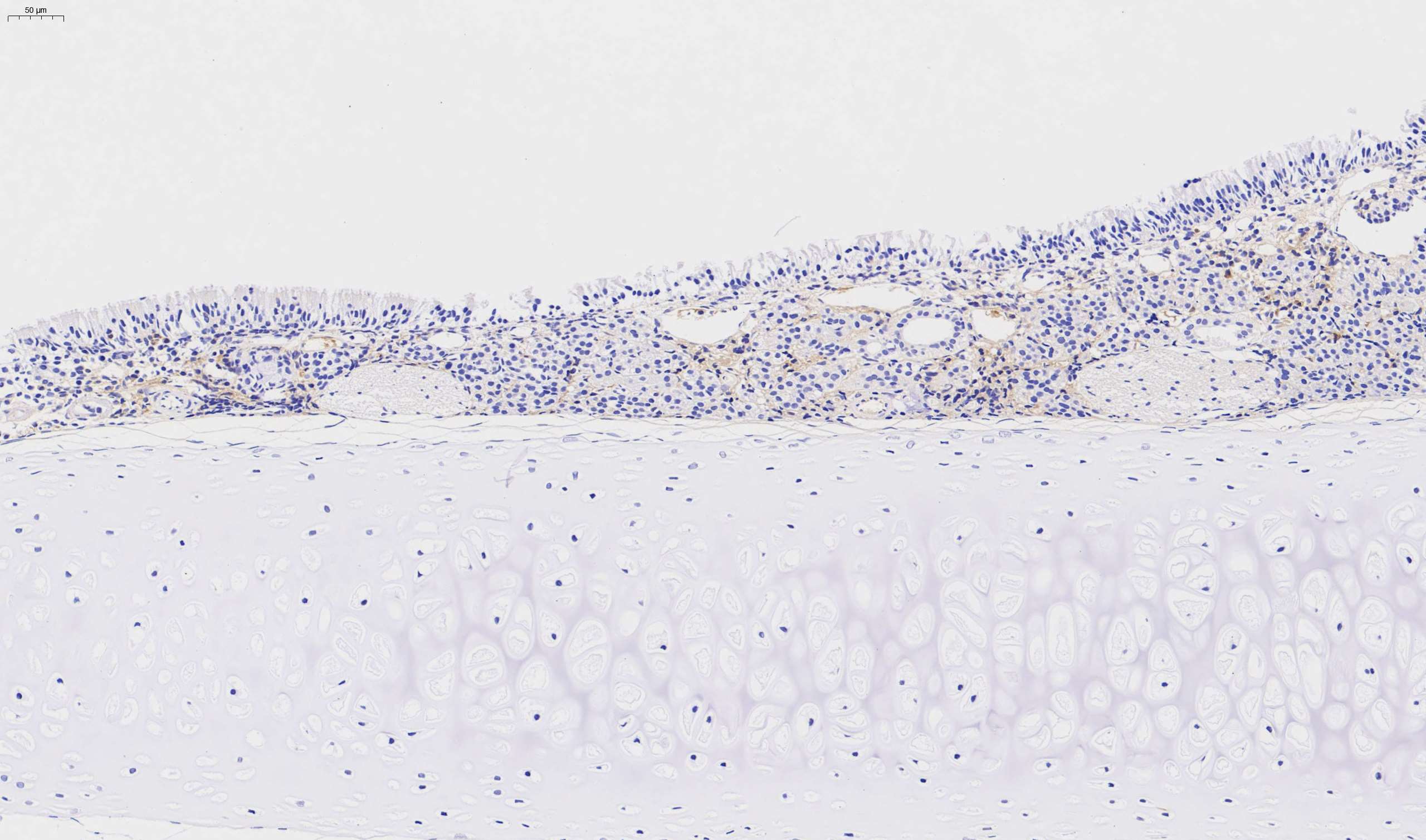

Supplement: Supplementary file 7 [file DataSheet5.ZIP › Microscopy images-Immunohistochemistry-GATA-3_200x_50um/CAVO-H/5 GATA-3_200x_50um_1.jpeg]

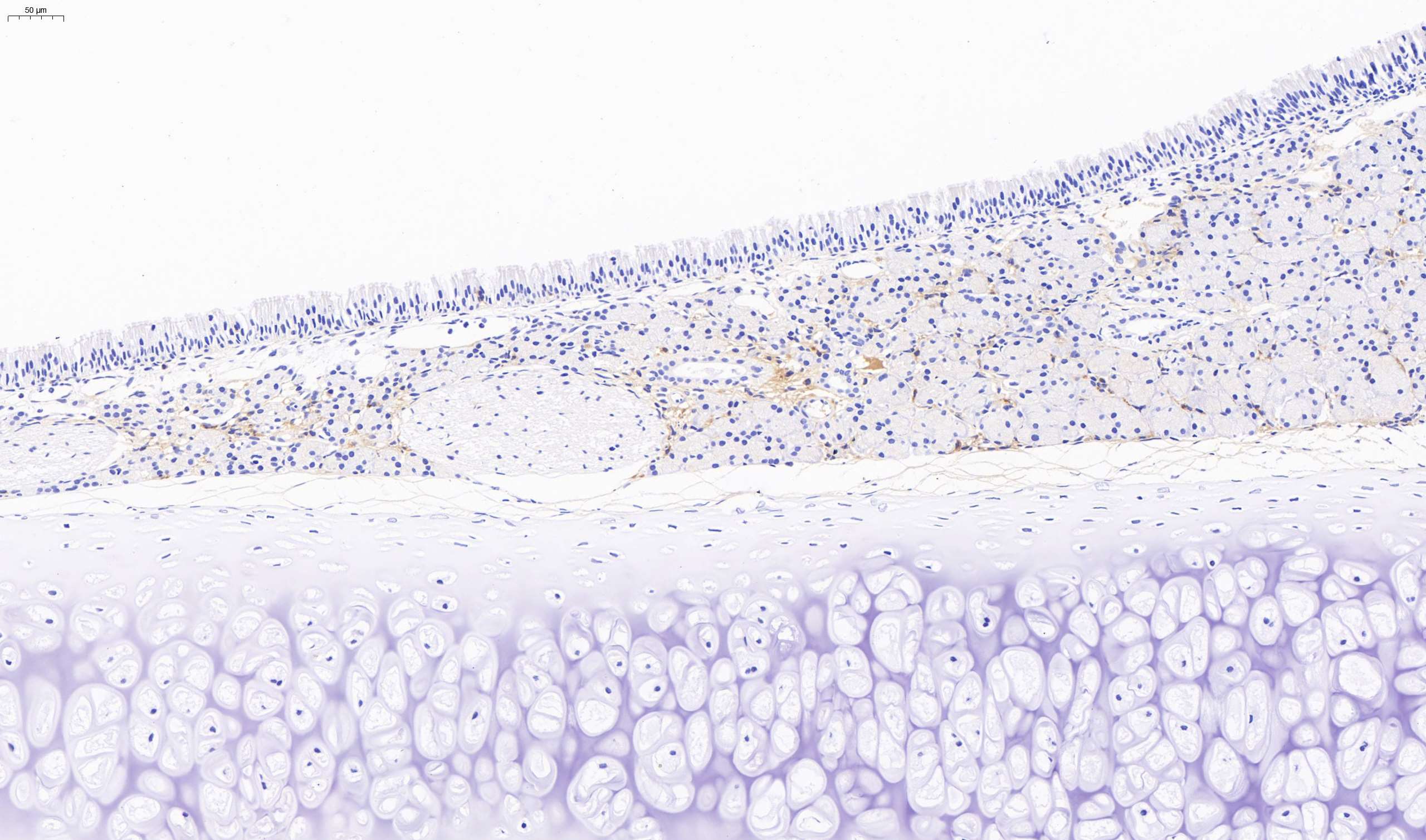

Supplement: Supplementary file 7 [file DataSheet5.ZIP › Microscopy images-Immunohistochemistry-GATA-3_200x_50um/CAVO-L/1 GATA-3_200x_50um_1.jpeg]

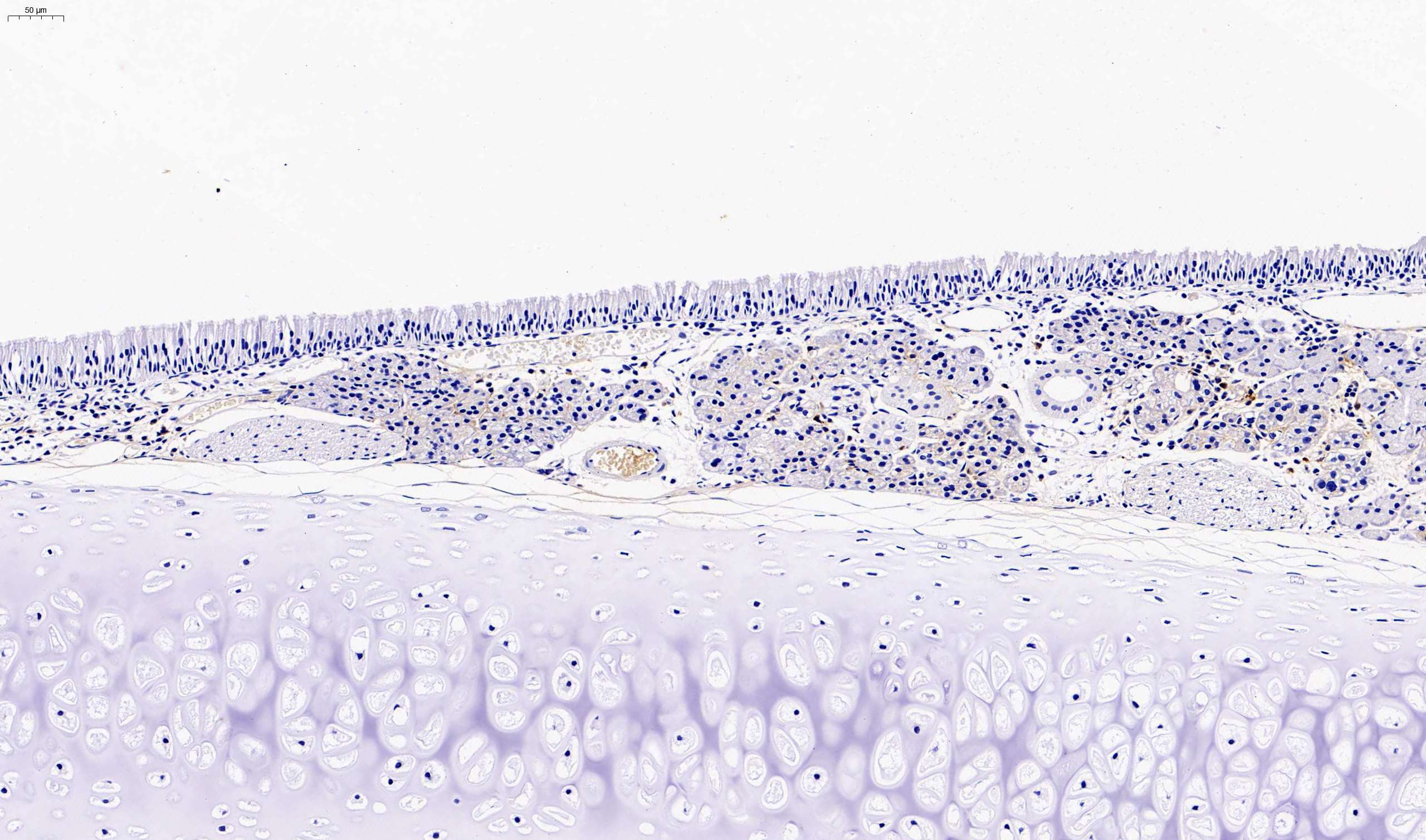

Supplement: Supplementary file 7 [file DataSheet5.ZIP › Microscopy images-Immunohistochemistry-GATA-3_200x_50um/CAVO-L/2 GATA-3_200x_50um_1.jpeg]

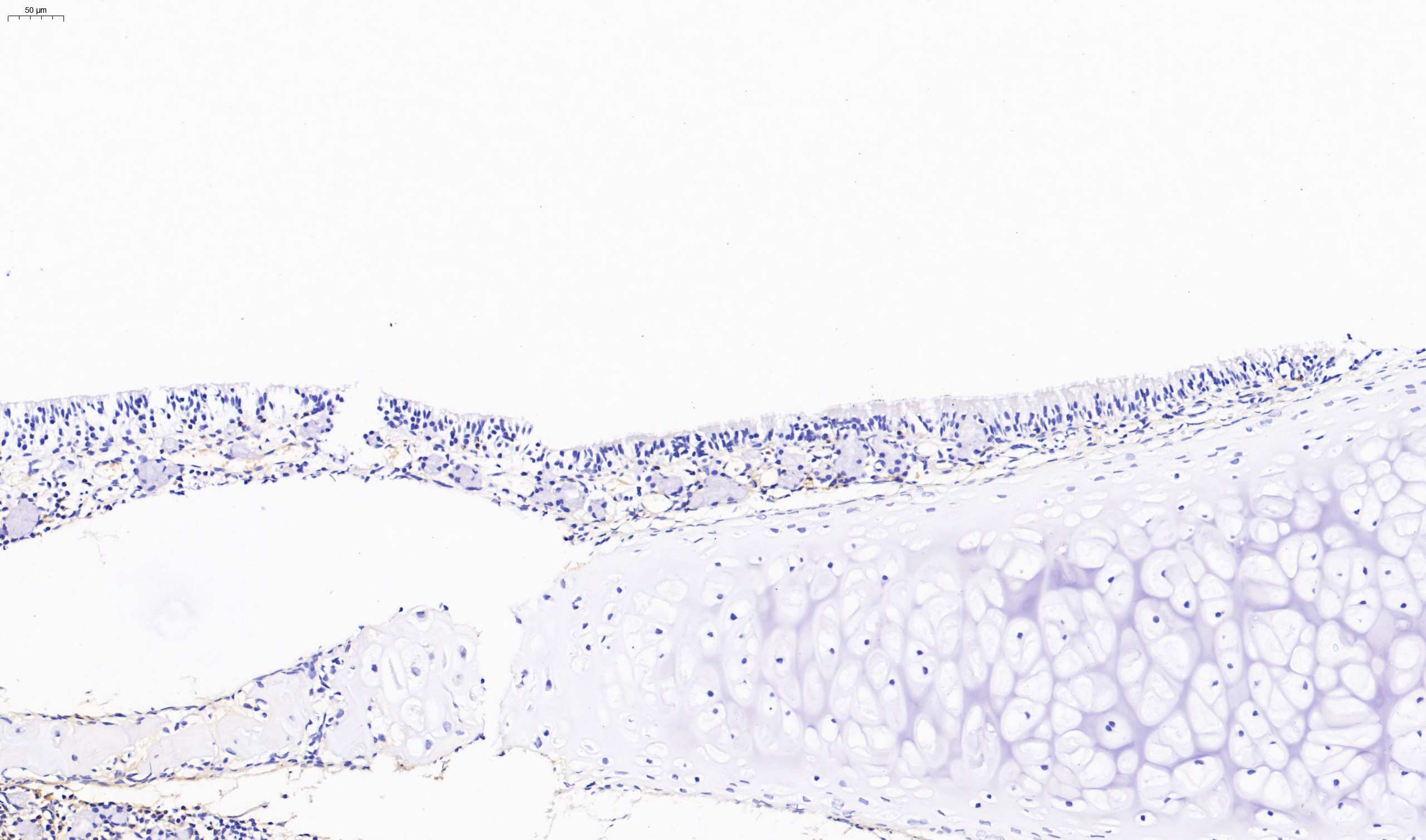

Supplement: Supplementary file 7 [file DataSheet5.ZIP › Microscopy images-Immunohistochemistry-GATA-3_200x_50um/CAVO-L/3 GATA-3_200x_50um_1.jpeg]

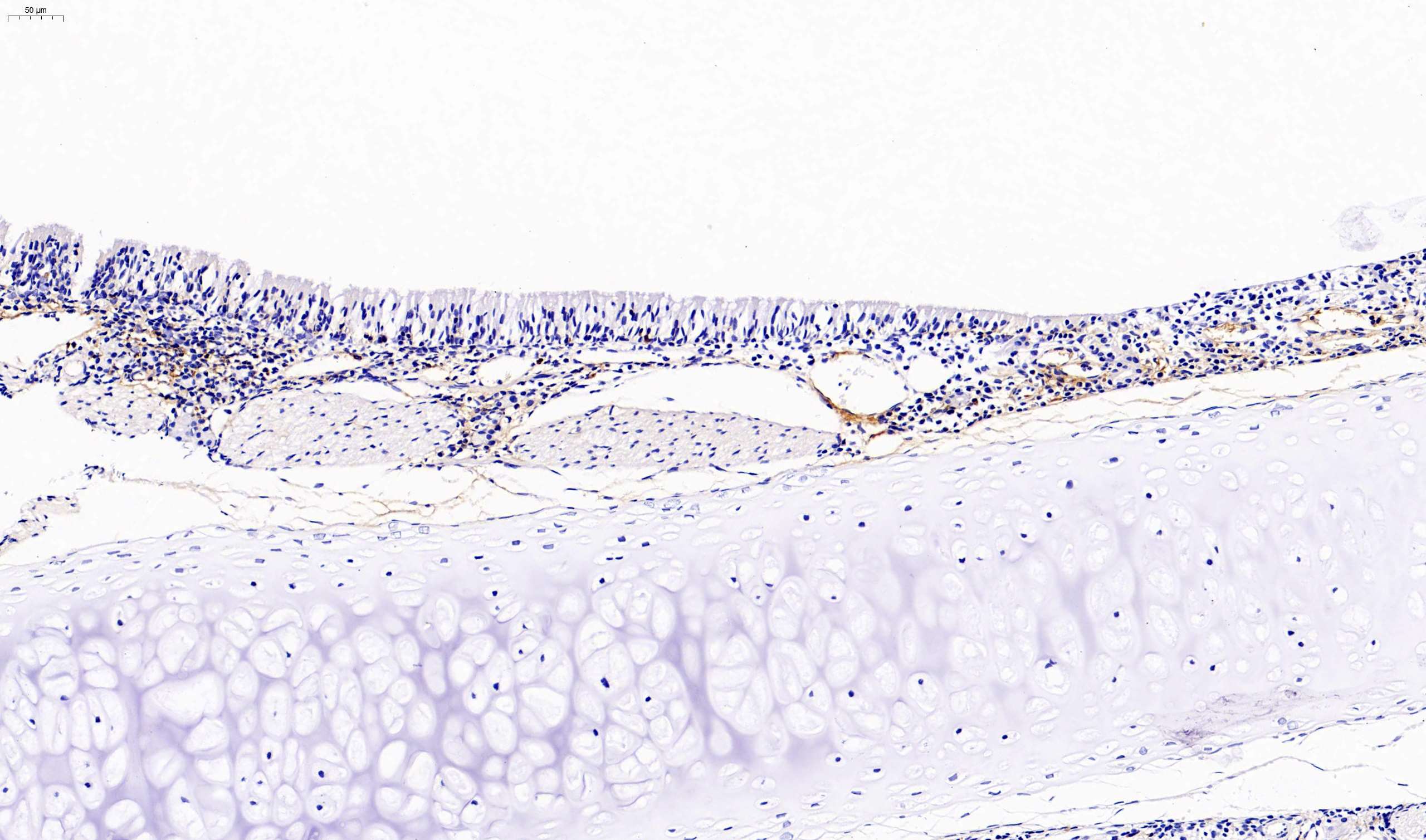

Supplement: Supplementary file 7 [file DataSheet5.ZIP › Microscopy images-Immunohistochemistry-GATA-3_200x_50um/CAVO-L/4 GATA-3_200x_50um_1.jpeg]

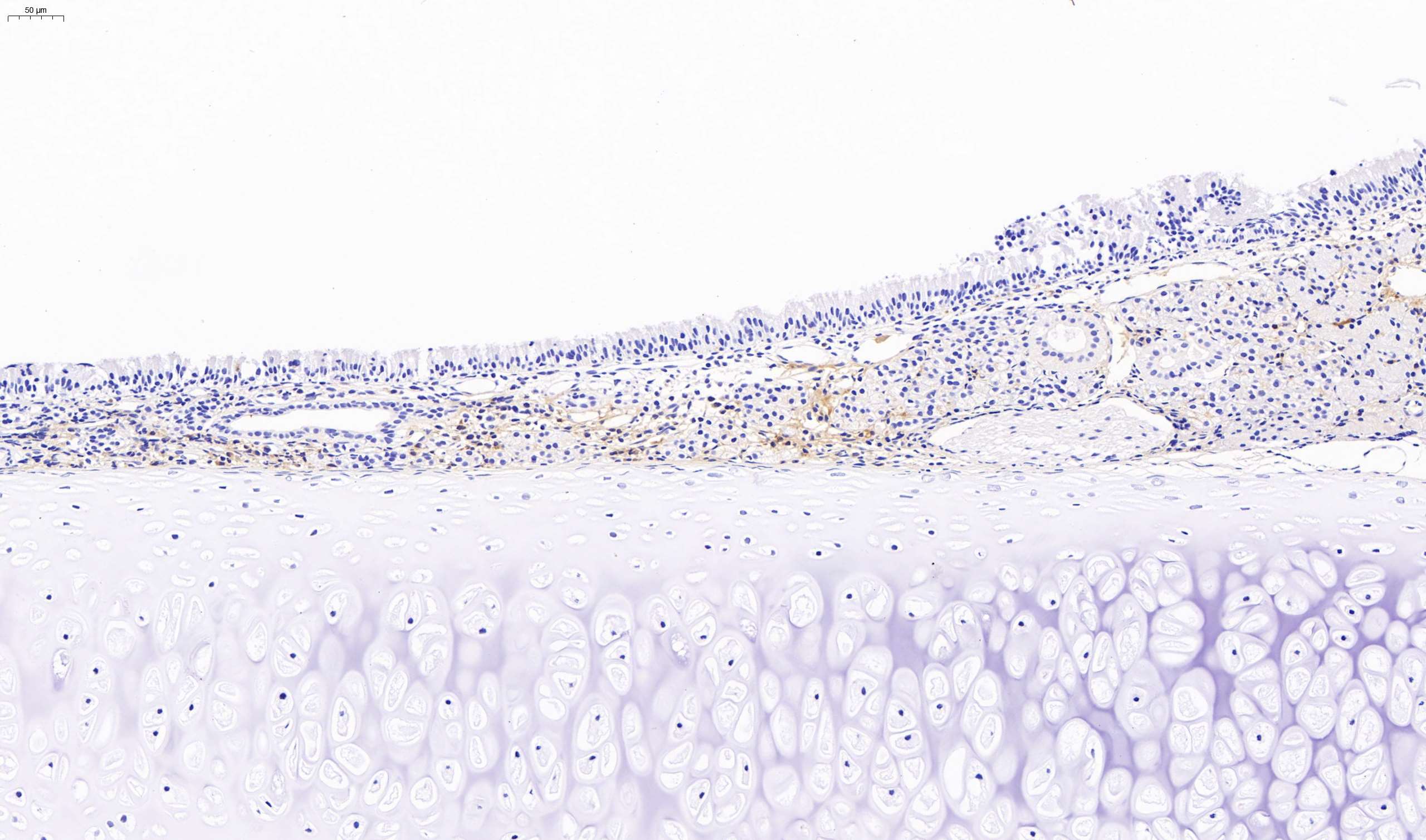

Supplement: Supplementary file 7 [file DataSheet5.ZIP › Microscopy images-Immunohistochemistry-GATA-3_200x_50um/CAVO-L/5 GATA-3_200x_50um_1.jpeg]

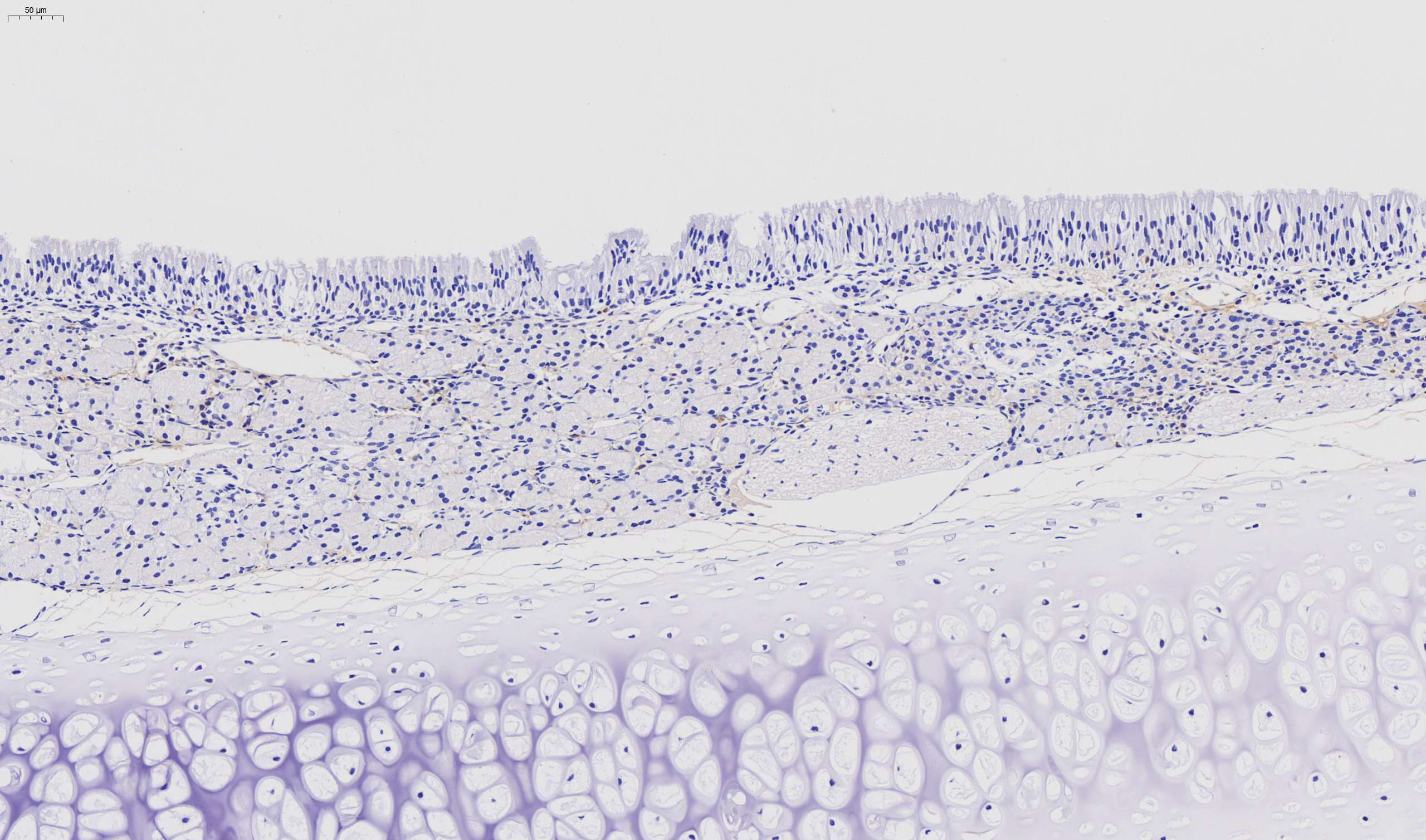

Supplement: Supplementary file 7 [file DataSheet5.ZIP › Microscopy images-Immunohistochemistry-GATA-3_200x_50um/CAVO-M/1 GATA-3_200x_50um_1.jpeg]

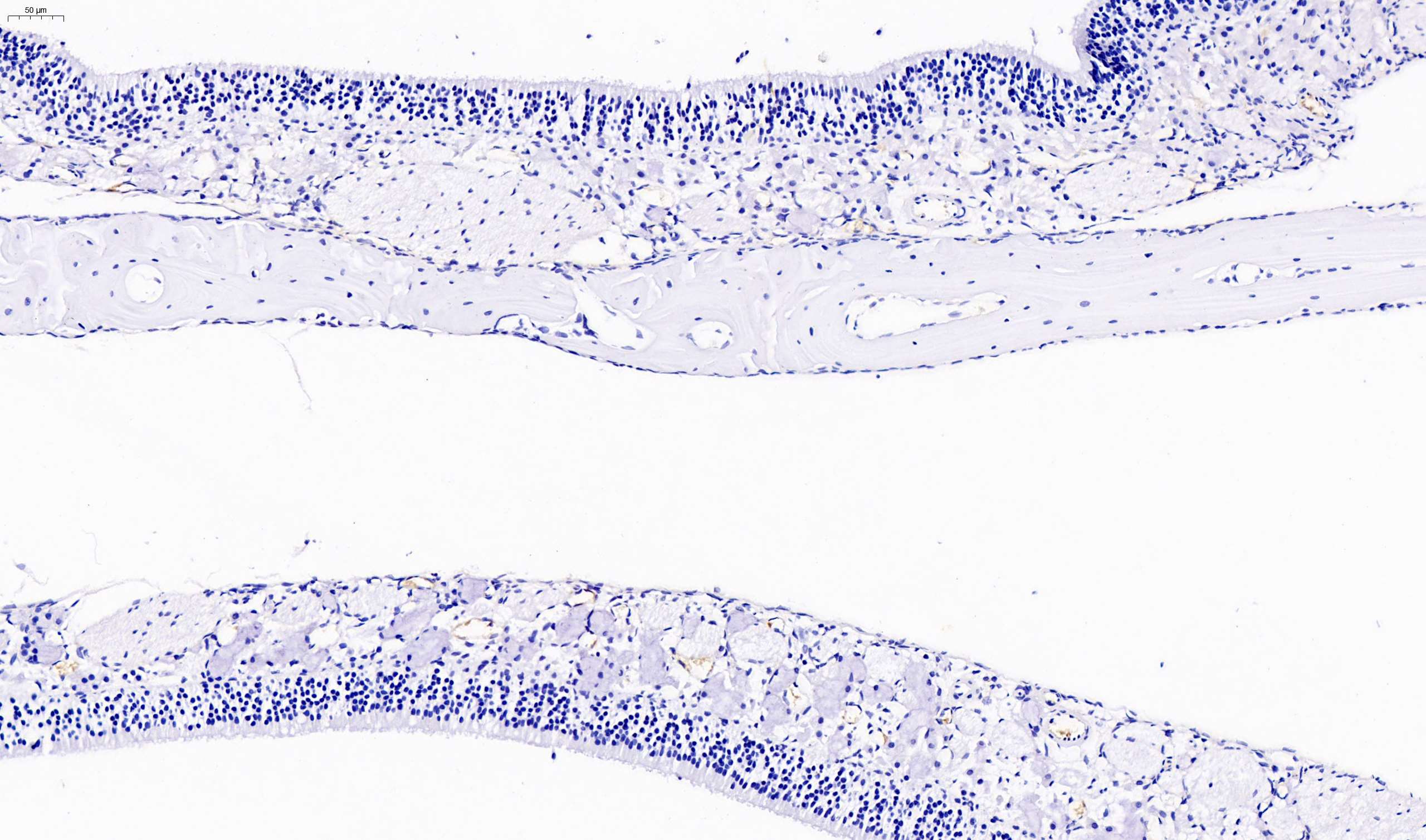

Supplement: Supplementary file 7 [file DataSheet5.ZIP › Microscopy images-Immunohistochemistry-GATA-3_200x_50um/CAVO-M/2 GATA-3_200x_50um_1.jpeg]

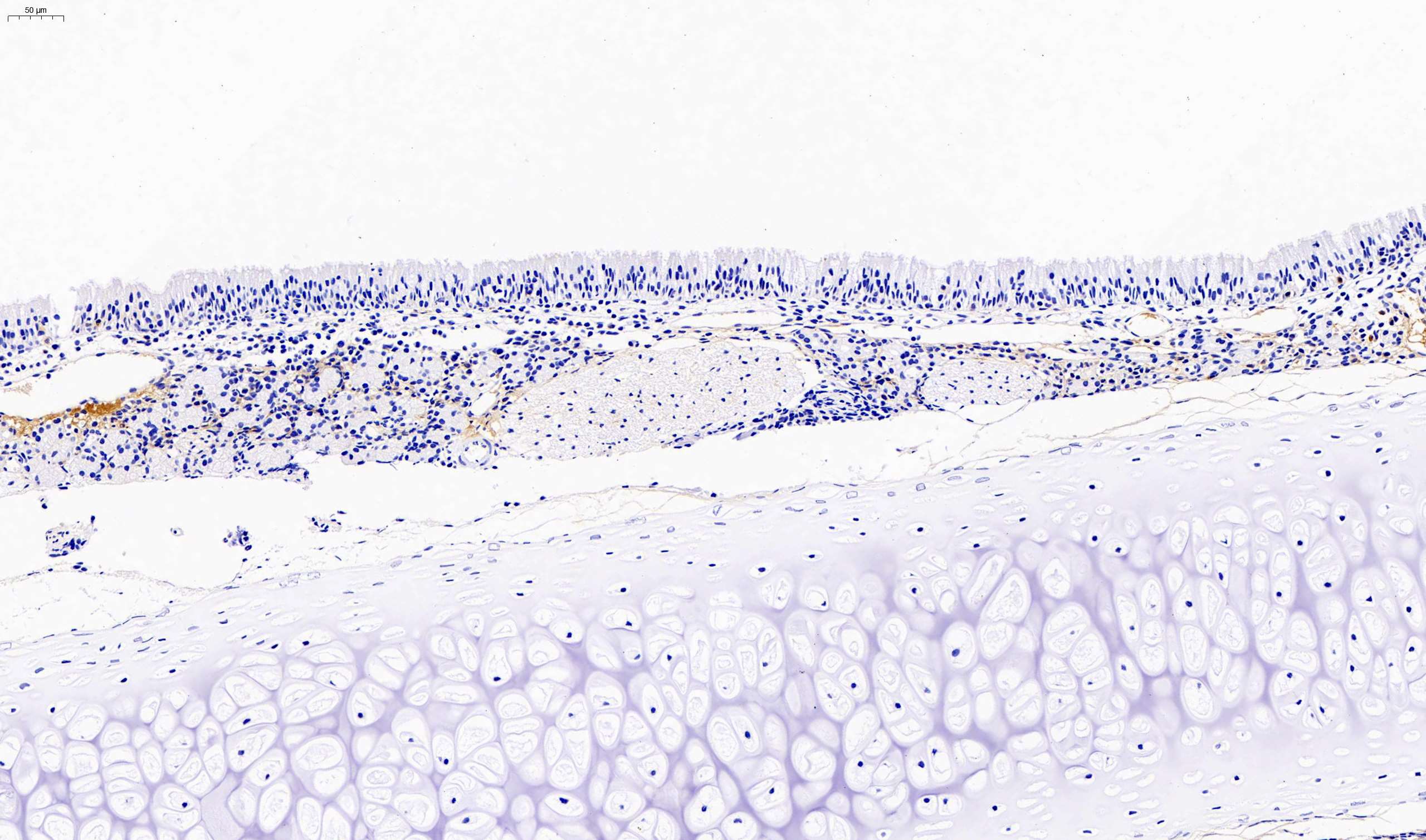

Supplement: Supplementary file 7 [file DataSheet5.ZIP › Microscopy images-Immunohistochemistry-GATA-3_200x_50um/CAVO-M/3 GATA-3_200x_50um_1.jpeg]

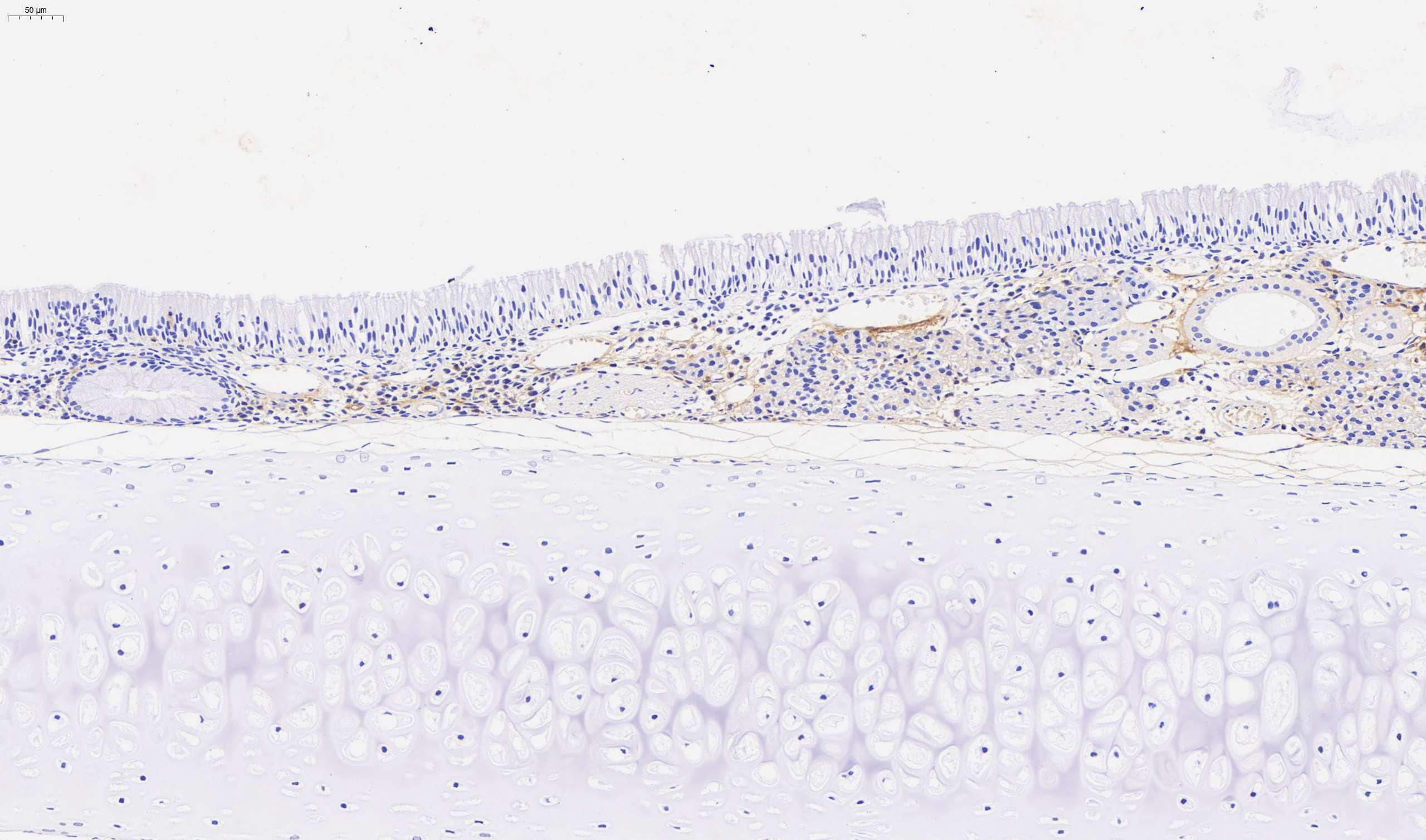

Supplement: Supplementary file 7 [file DataSheet5.ZIP › Microscopy images-Immunohistochemistry-GATA-3_200x_50um/CAVO-M/4 GATA-3_200x_50um _1.jpeg]

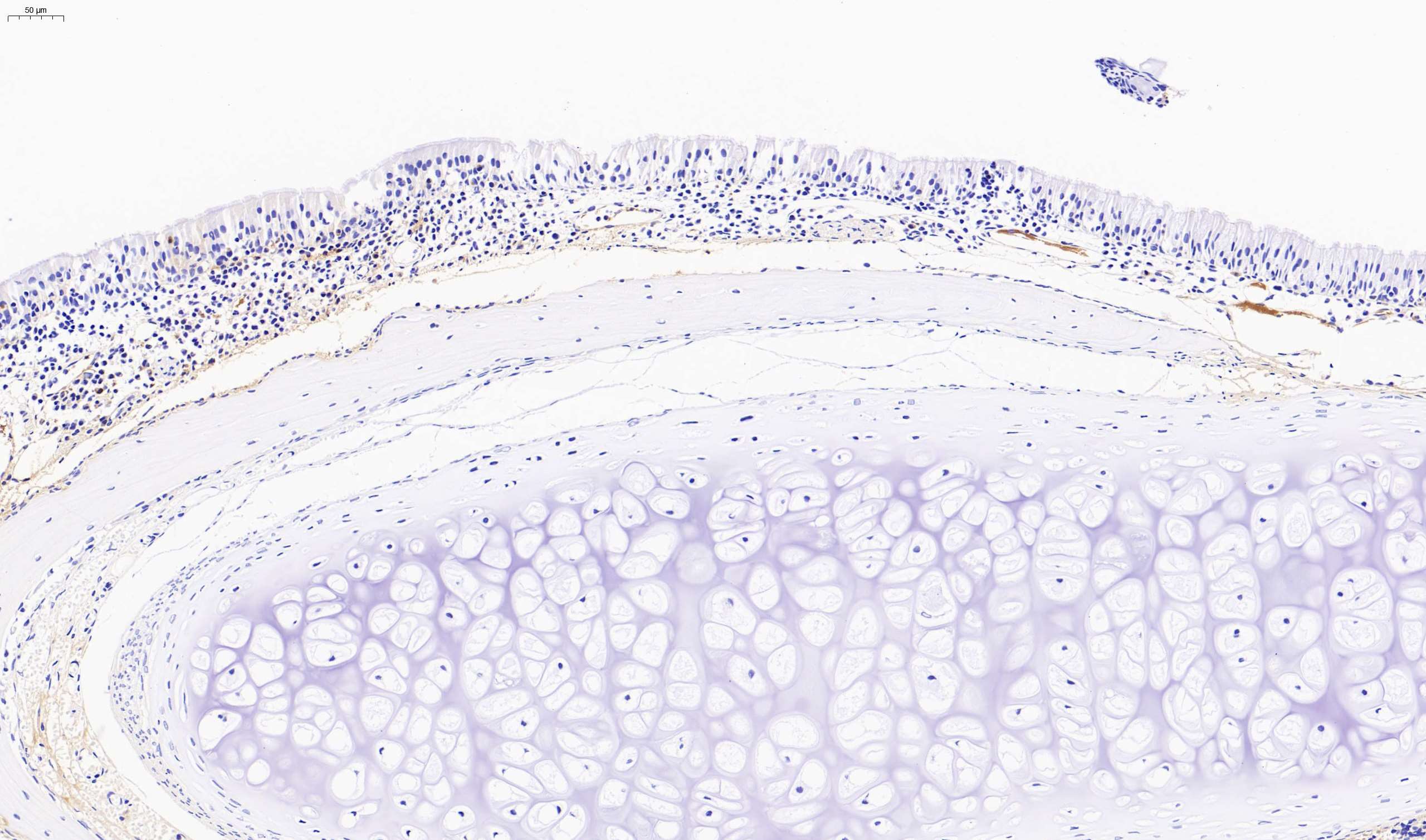

Supplement: Supplementary file 7 [file DataSheet5.ZIP › Microscopy images-Immunohistochemistry-GATA-3_200x_50um/CAVO-M/5 GATA-3_200x_50um_1.jpeg]
