# Supplementary material for: Cang-ai volatile oil alleviates nasal inflammation via Th1/Th2 cell imbalance regulation in a rat model of ovalbumin-induced allergic rhinitis
Source: Front Pharmacol. 2024 May 21;15:1332036. doi: 10.3389/fphar.2024.1332036 (PMC11148258; doi:10.3389/fphar.2024.1332036)
Supplement: Supplementary file 6 [file DataSheet8.PDF]

IFN-γ and IL-4

Control

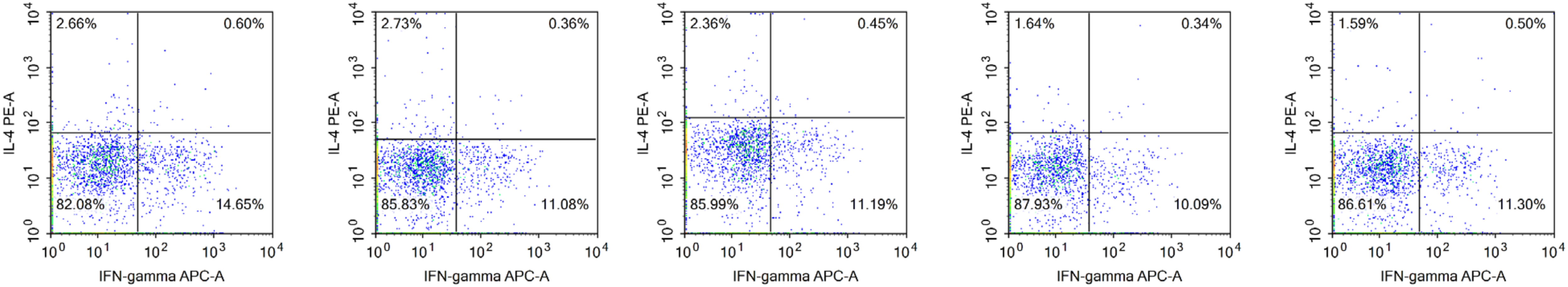

Model

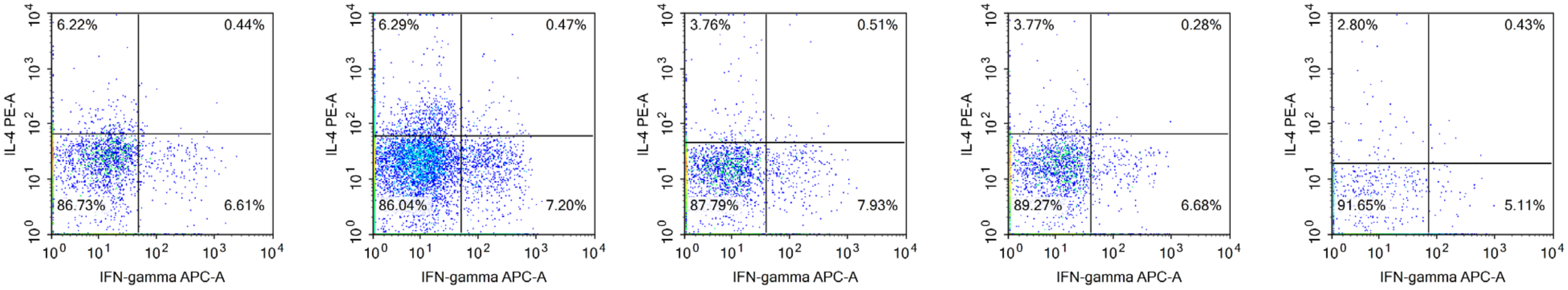

IL-4 PE-A
IFN-gamma APC-A
6.22%
0.44%
86.73%
6.61%
6.29%
0.47%
86.04%
7.20%
3.76%
0.51%
87.79%
7.93%
3.77%
0.28%
89.27%
6.68%
2.80%
0.43%
91.65%
5.11%

CAVO-L

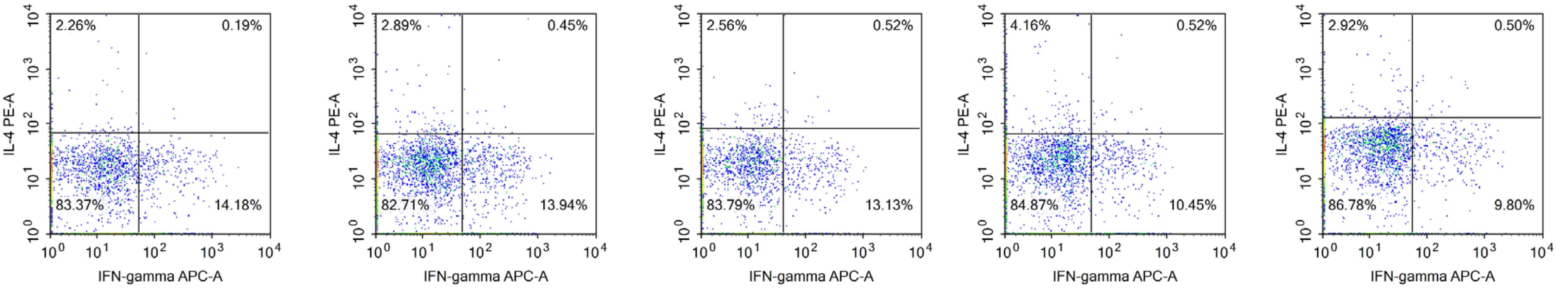

IL-4 PE-A
IFN-gamma APC-A
2.26%
0.19%
83.37%
14.18%
2.89%
0.45%
82.71%
13.94%
2.56%
0.52%
83.79%
13.13%
4.16%
0.52%
84.87%
10.45%
2.92%
0.50%
86.78%
9.80%

CAVO-M

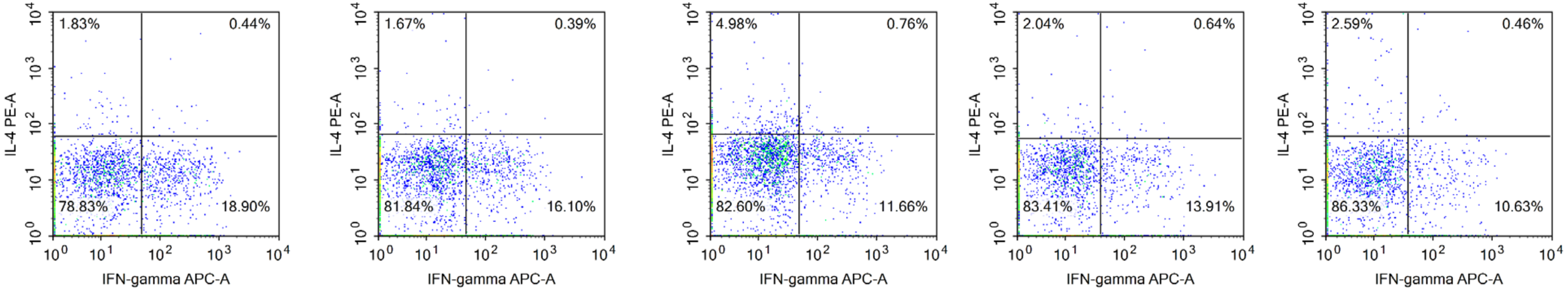

1.83%
0.44%
78.83%
18.90%
1.67%
0.39%
81.84%
16.10%
4.98%
0.76%
82.60%
11.66%
2.04%
0.64%
83.41%
13.91%
2.59%
0.46%
86.33%
10.63%
IL-4 PE-A
IFN-gamma APC-A

CAVO-H

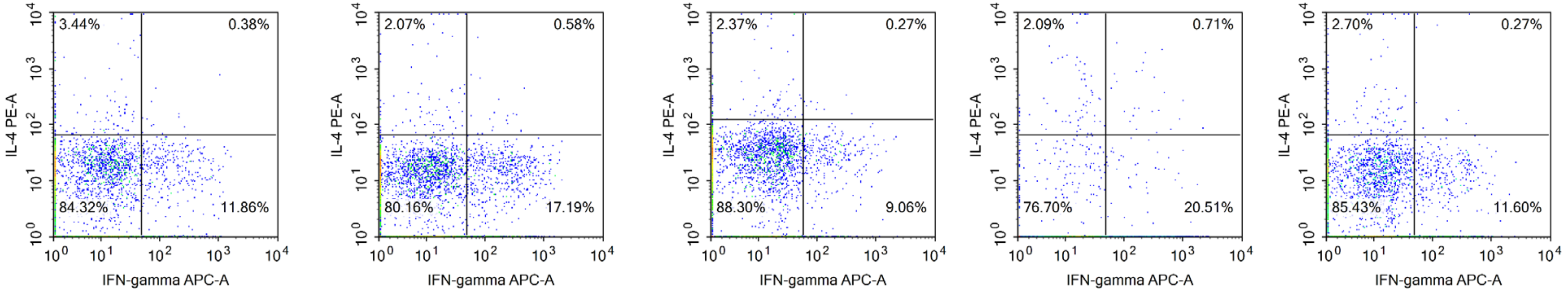

3.44%
0.38%
84.32%
11.86%
2.07%
0.58%
80.16%
17.19%
2.37%
0.27%
88.30%
9.06%
2.09%
0.71%
76.70%
20.51%
2.70%
0.27%
85.43%
11.60%
IL-4 PE-A
IFN-gamma APC-A

T-bet

Control

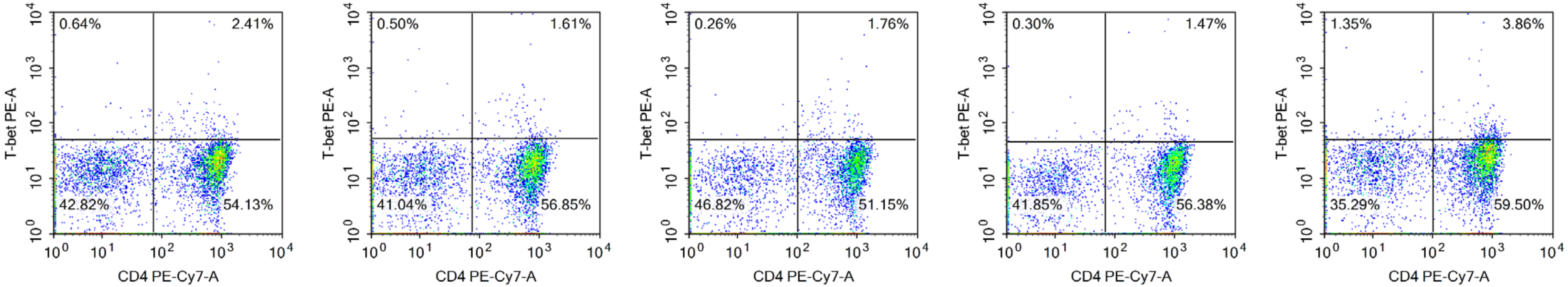

Model

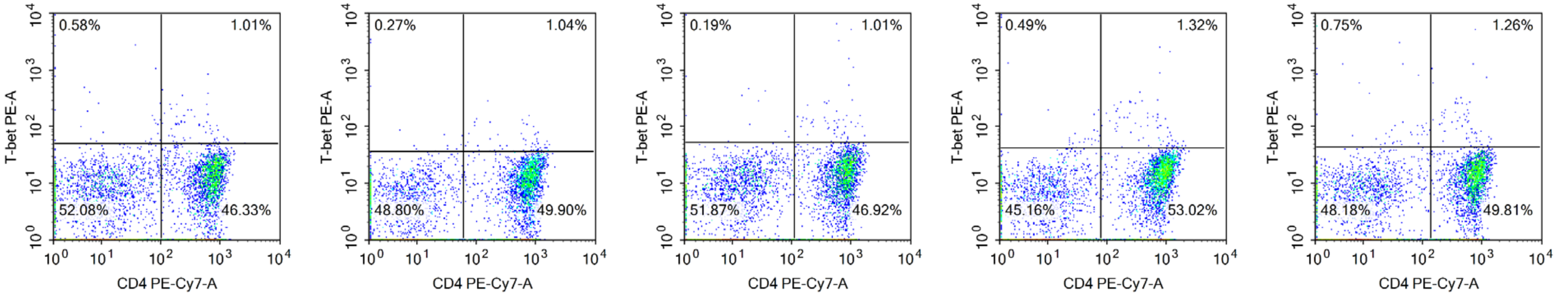
T-bet PE-A
CD4 PE-Cy7-A
0.58%
1.01%
52.08%
46.33%
0.27%
1.04%
48.80%
49.90%
0.19%
1.01%
51.87%
46.92%
0.49%
1.32%
45.16%
53.02%
0.75%
1.26%
48.18%
49.81%

CAVO-L

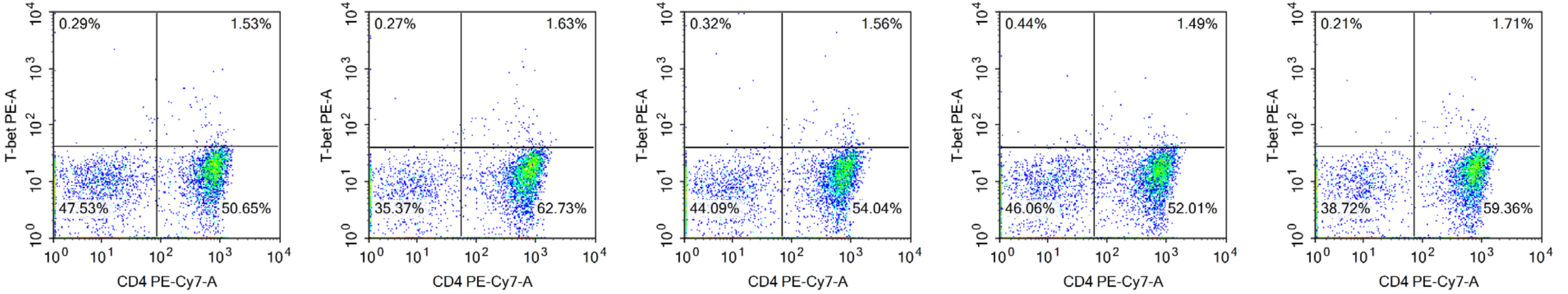
T-bet PE-A
CD4 PE-Cy7-A
0.29%
1.53%
47.53%
50.65%
0.27%
1.63%
35.37%
62.73%
0.32%
1.56%
44.09%
54.04%
0.44%
1.49%
46.06%
52.01%
0.21%
1.71%
38.72%
59.36%

CAVO-M

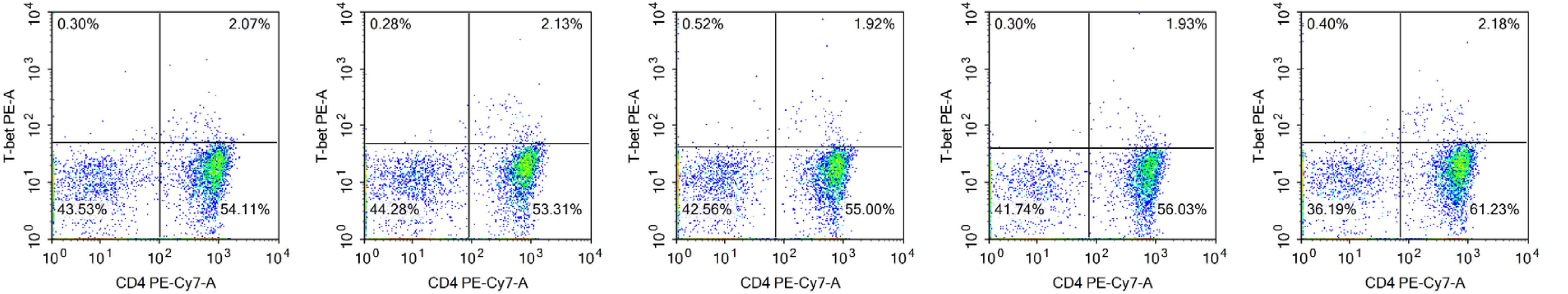
T-bet PE-A
CD4 PE-Cy7-A
0.30%
2.07%
43.53%
54.11%
0.28%
2.13%
44.28%
53.31%
0.52%
1.92%
42.56%
55.00%
0.30%
1.93%
41.74%
56.03%
0.40%
2.18%
36.19%
61.23%

CAVO-H

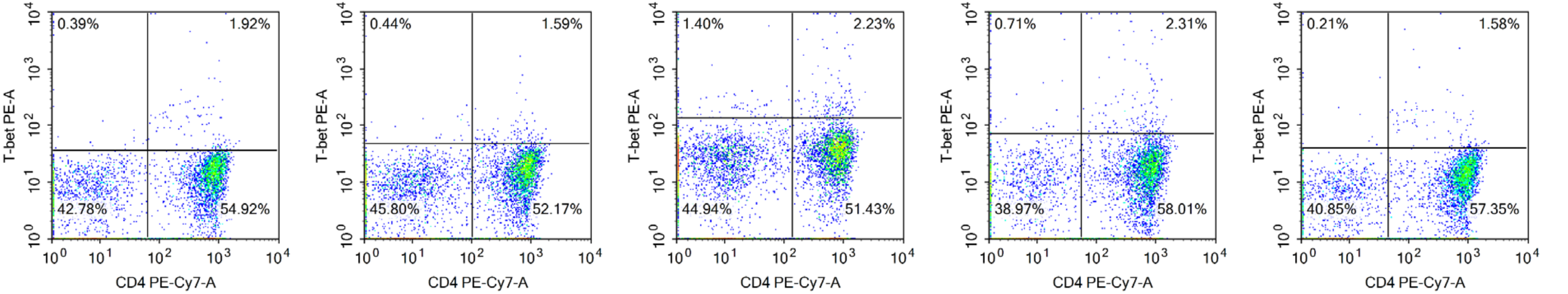
T-bet PE-A
CD4 PE-Cy7-A
0.39%
1.92%
42.78%
54.92%
0.44%
1.59%
45.80%
52.17%
1.40%
2.23%
44.94%
51.43%
0.71%
2.31%
38.97%
58.01%
0.21%
1.58%
40.85%
57.35%

GATA-3

Control

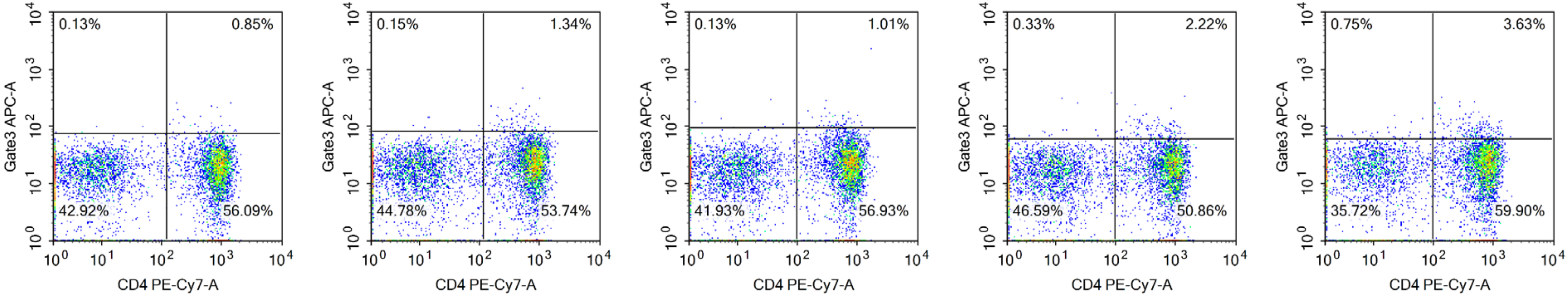

Model

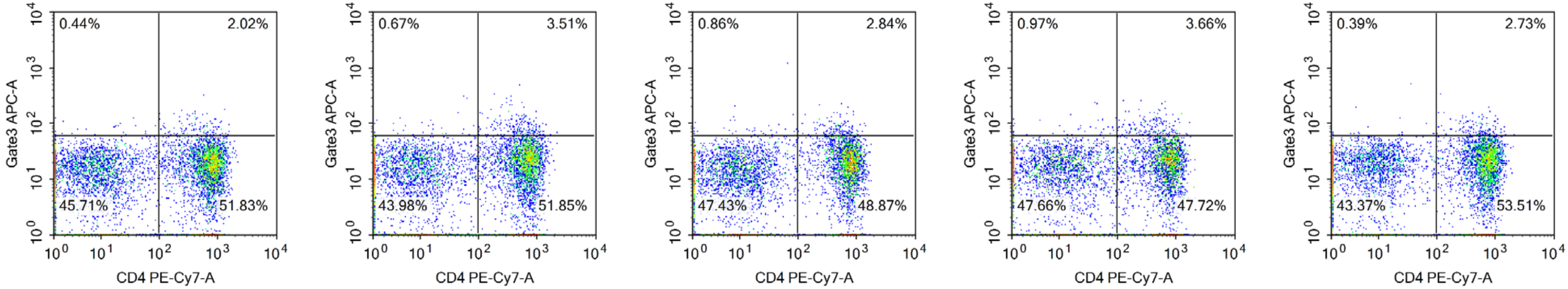

Gate3 APC-A
CD4 PE-Cy7-A
0.44%
2.02%
45.71%
51.83%
0.67%
3.51%
43.98%
51.85%
0.86%
2.84%
47.43%
48.87%
0.97%
3.66%
47.66%
47.72%
0.39%
2.73%
43.37%
53.51%

CAVO-L

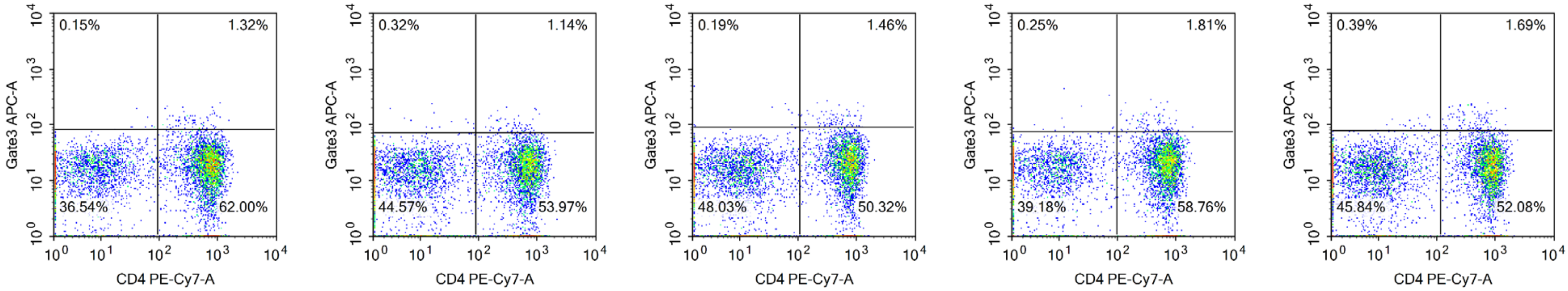

Gate3 APC-A
CD4 PE-Cy7-A
0.15%
1.32%
36.54%
62.00%
0.32%
1.14%
44.57%
53.97%
0.19%
1.46%
48.03%
50.32%
0.25%
1.81%
39.18%
58.76%
0.39%
1.69%
45.84%
52.08%

CAVO-M

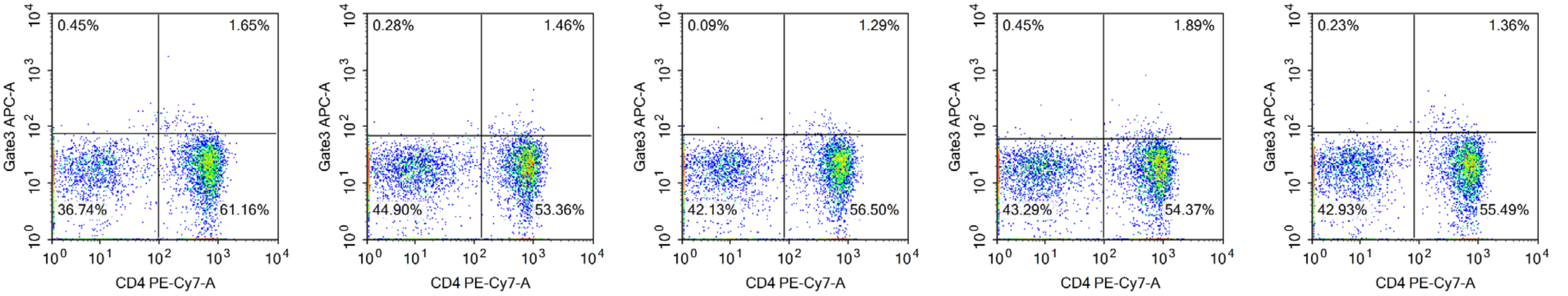
0.45%
1.65%
36.74%
61.16%
0.28%
1.46%
44.90%
53.36%
0.09%
1.29%
42.13%
56.50%
0.45%
1.89%
43.29%
54.37%
0.23%
1.36%
42.93%
55.49%
Gate3 APC-A
CD4 PE-Cy7-A

CAVO-H

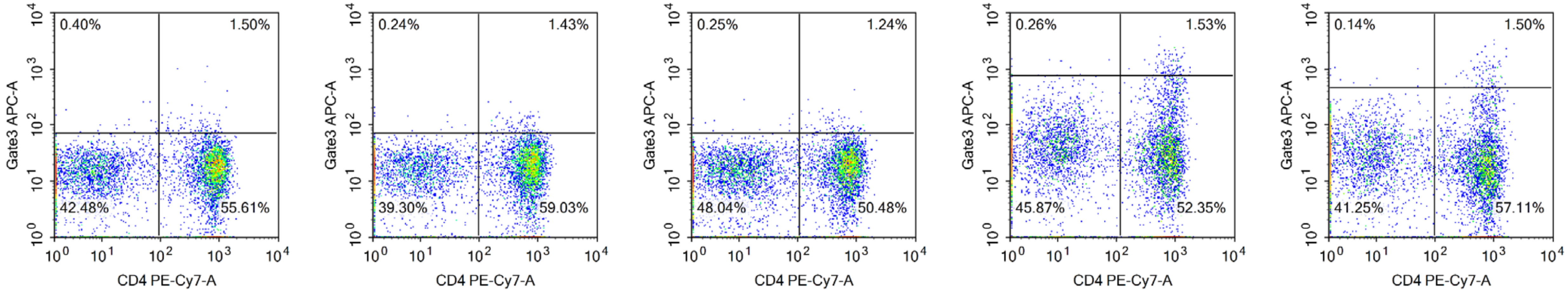
0.40%
1.50%
42.48%
55.61%
0.24%
1.43%
39.30%
59.03%
0.25%
1.24%
48.04%
50.48%
0.26%
1.53%
45.87%
52.35%
0.14%
1.50%
41.25%
57.11%
Gate3 APC-A
CD4 PE-Cy7-A
