# Supplementary figures and images for: Cang-ai volatile oil alleviates nasal inflammation via Th1/Th2 cell imbalance regulation in a rat model of ovalbumin-induced allergic rhinitis (part 2 of 2)
Source: Front Pharmacol. 2024 May 21;15:1332036. doi: 10.3389/fphar.2024.1332036 (PMC11148258; doi:10.3389/fphar.2024.1332036)

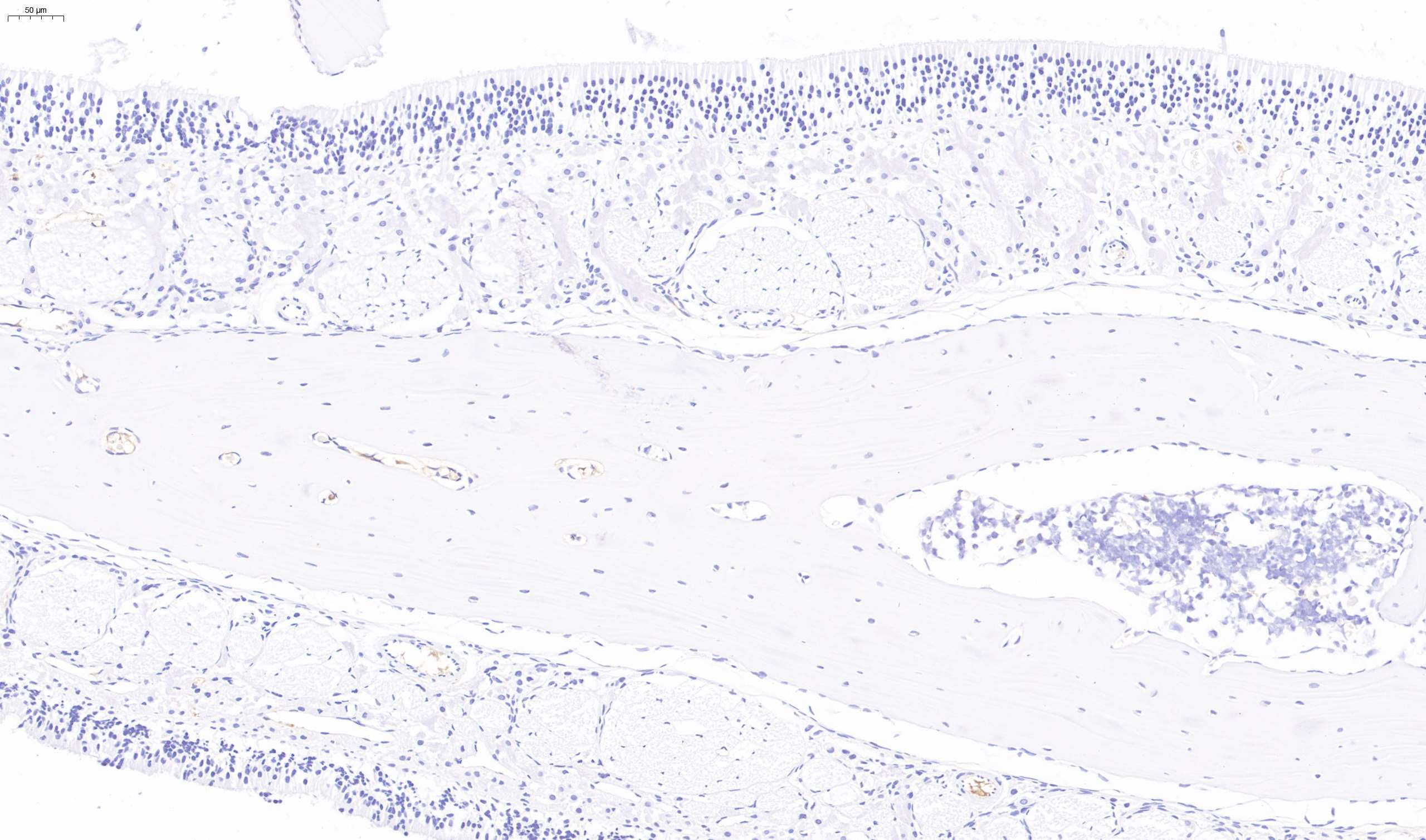

Supplement: Supplementary file 7 [file DataSheet5.ZIP › Microscopy images-Immunohistochemistry-GATA-3_200x_50um/Control/1 GATA3_200_50umx_1.jpeg]

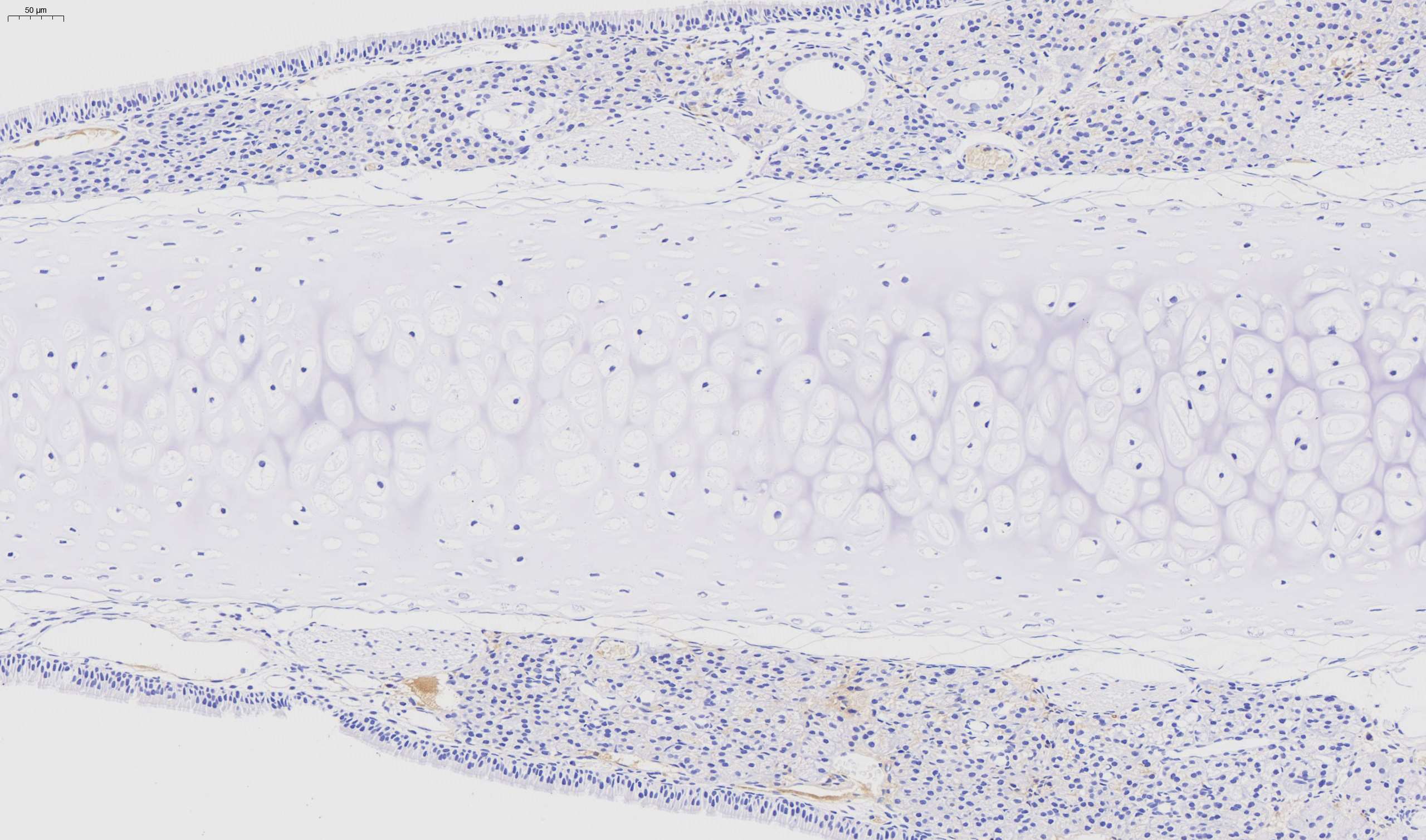

Supplement: Supplementary file 7 [file DataSheet5.ZIP › Microscopy images-Immunohistochemistry-GATA-3_200x_50um/Control/2 GATA3_200x_50um_1.jpeg]

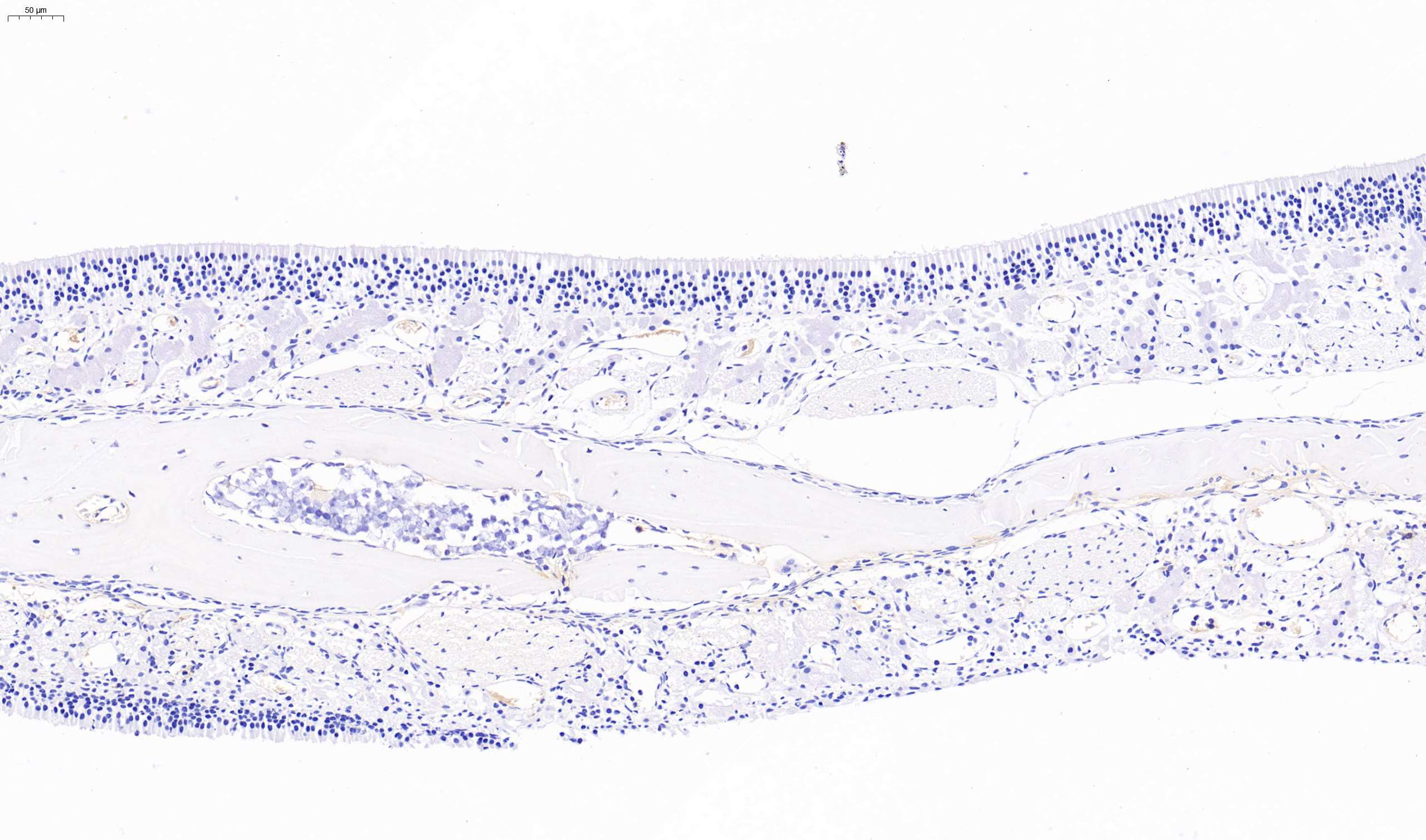

Supplement: Supplementary file 7 [file DataSheet5.ZIP › Microscopy images-Immunohistochemistry-GATA-3_200x_50um/Control/3 GATA3_200x_50um_1.jpeg]

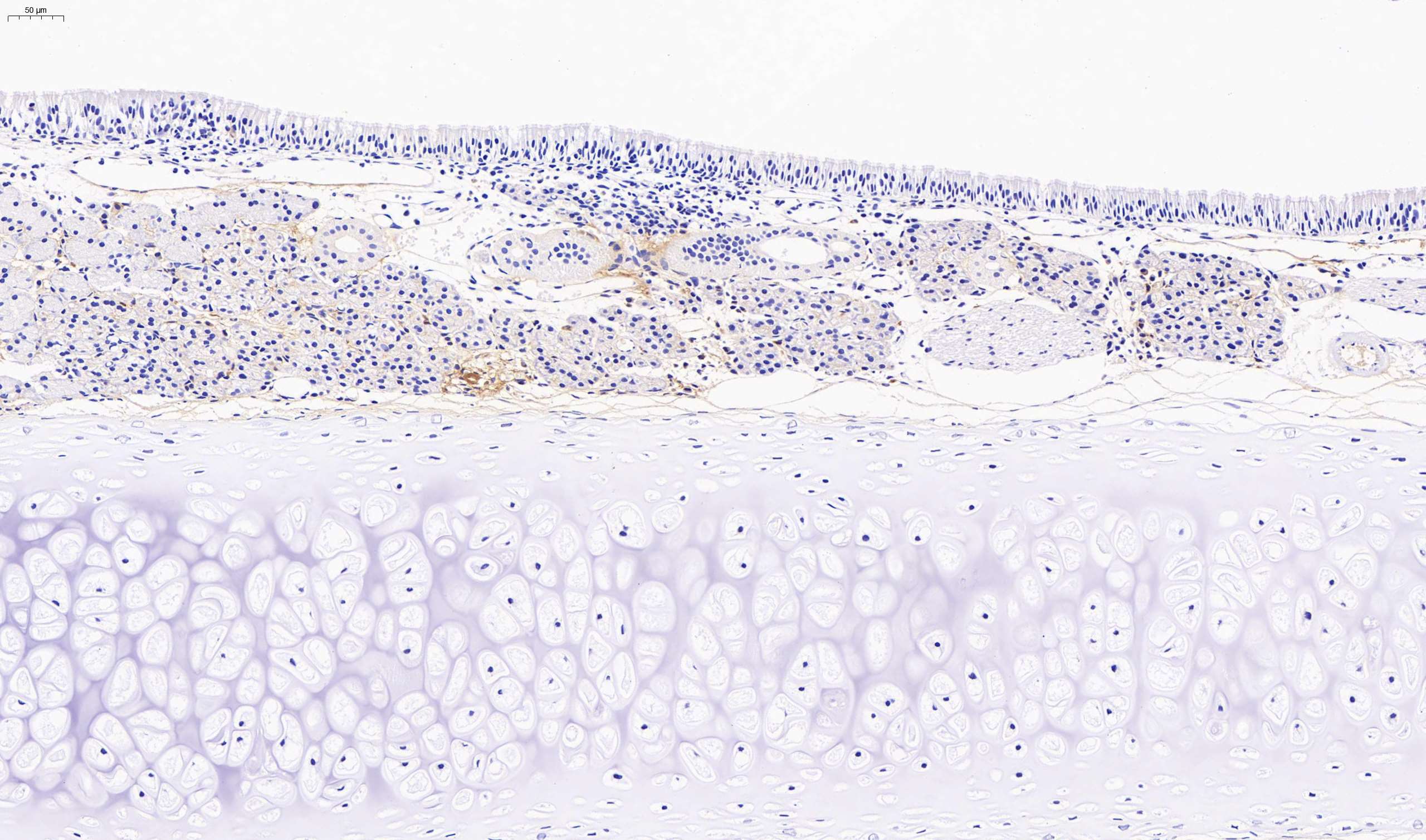

Supplement: Supplementary file 7 [file DataSheet5.ZIP › Microscopy images-Immunohistochemistry-GATA-3_200x_50um/Control/4 GATA3_200x_50um_1.jpeg]

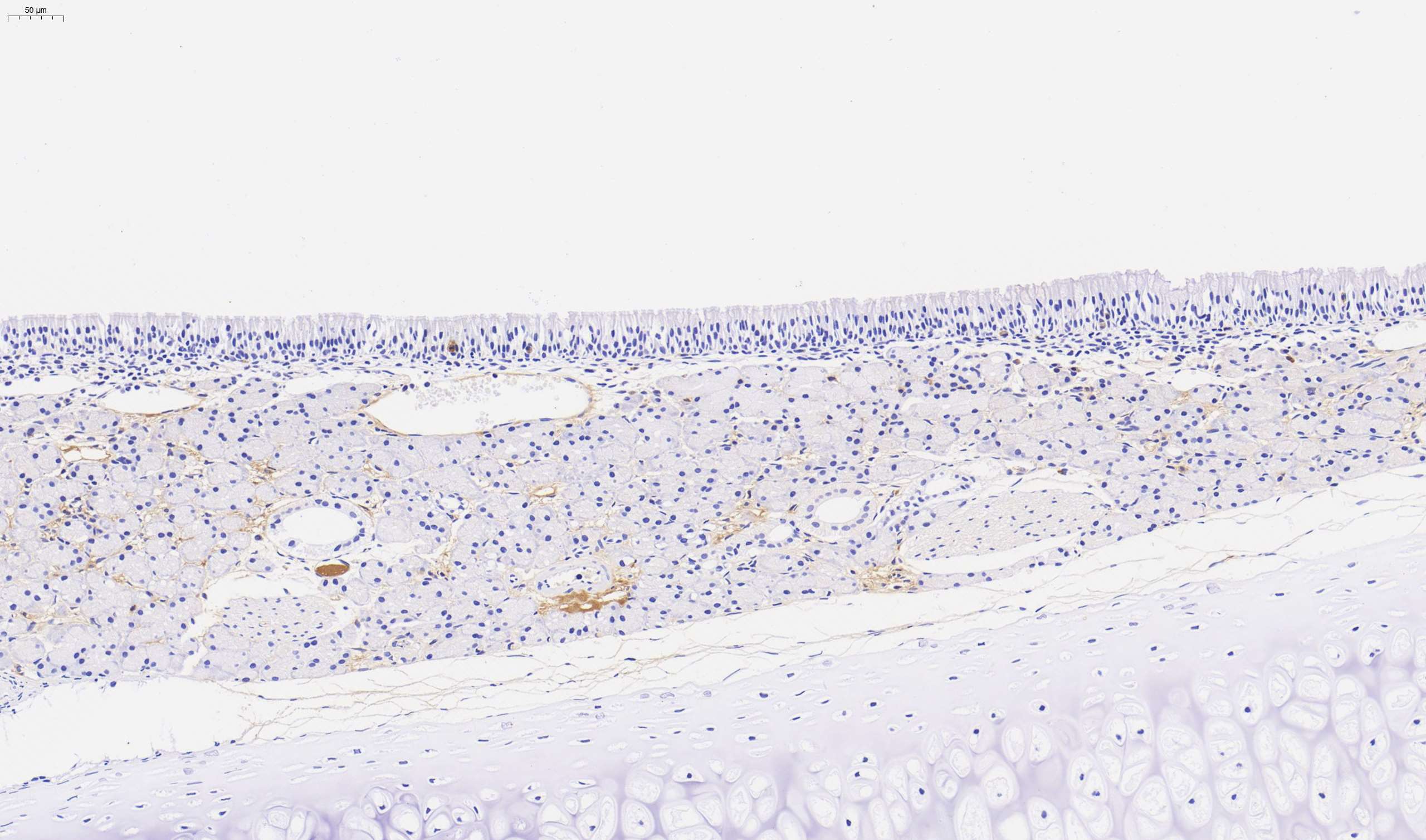

Supplement: Supplementary file 7 [file DataSheet5.ZIP › Microscopy images-Immunohistochemistry-GATA-3_200x_50um/Control/5 GATA-3_200x_50um_1.jpeg]

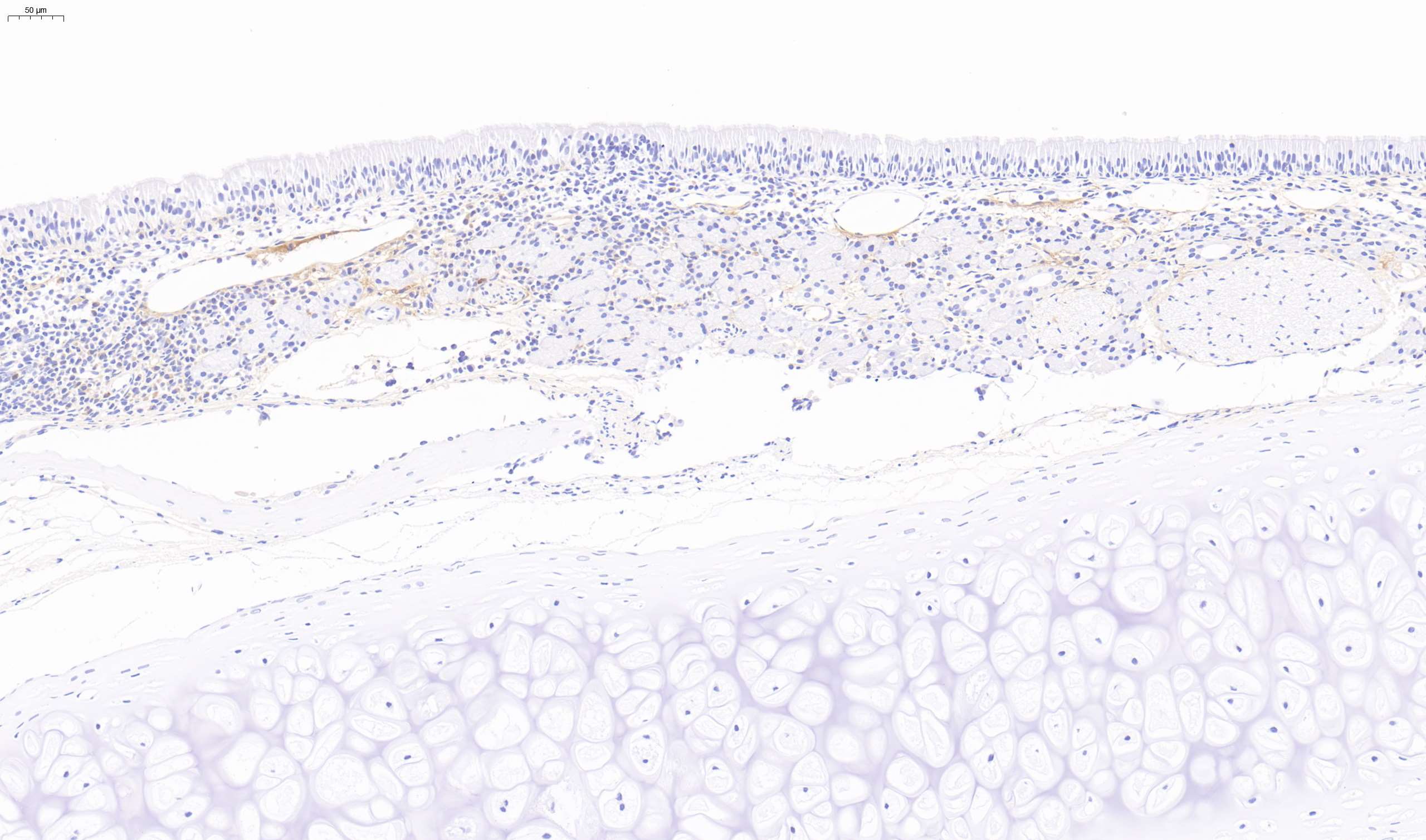

Supplement: Supplementary file 7 [file DataSheet5.ZIP › Microscopy images-Immunohistochemistry-GATA-3_200x_50um/Loratadine/1 GATA-3_200x_50um_1.jpeg]

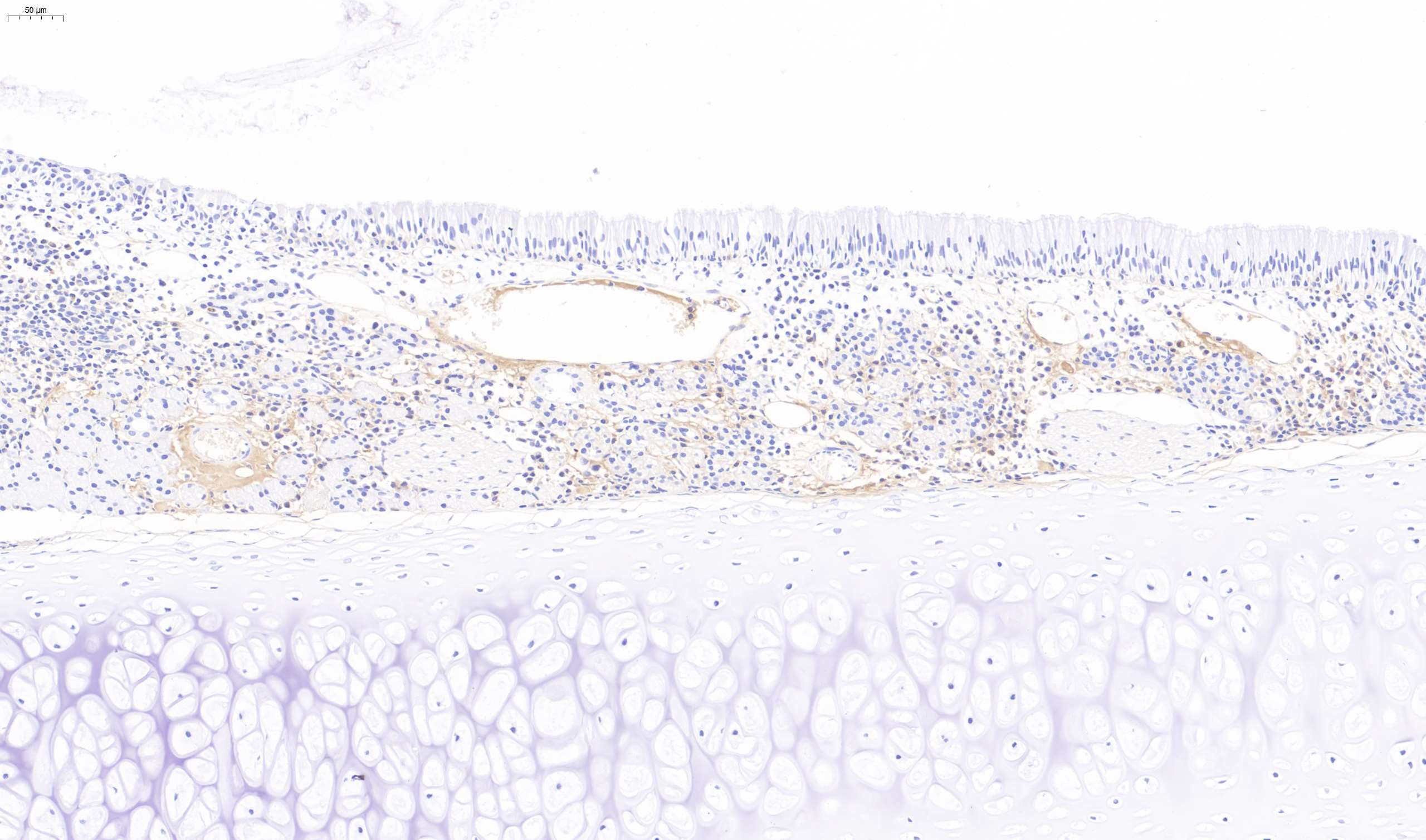

Supplement: Supplementary file 7 [file DataSheet5.ZIP › Microscopy images-Immunohistochemistry-GATA-3_200x_50um/Loratadine/2 GATA-3_200x_50um_1.jpeg]

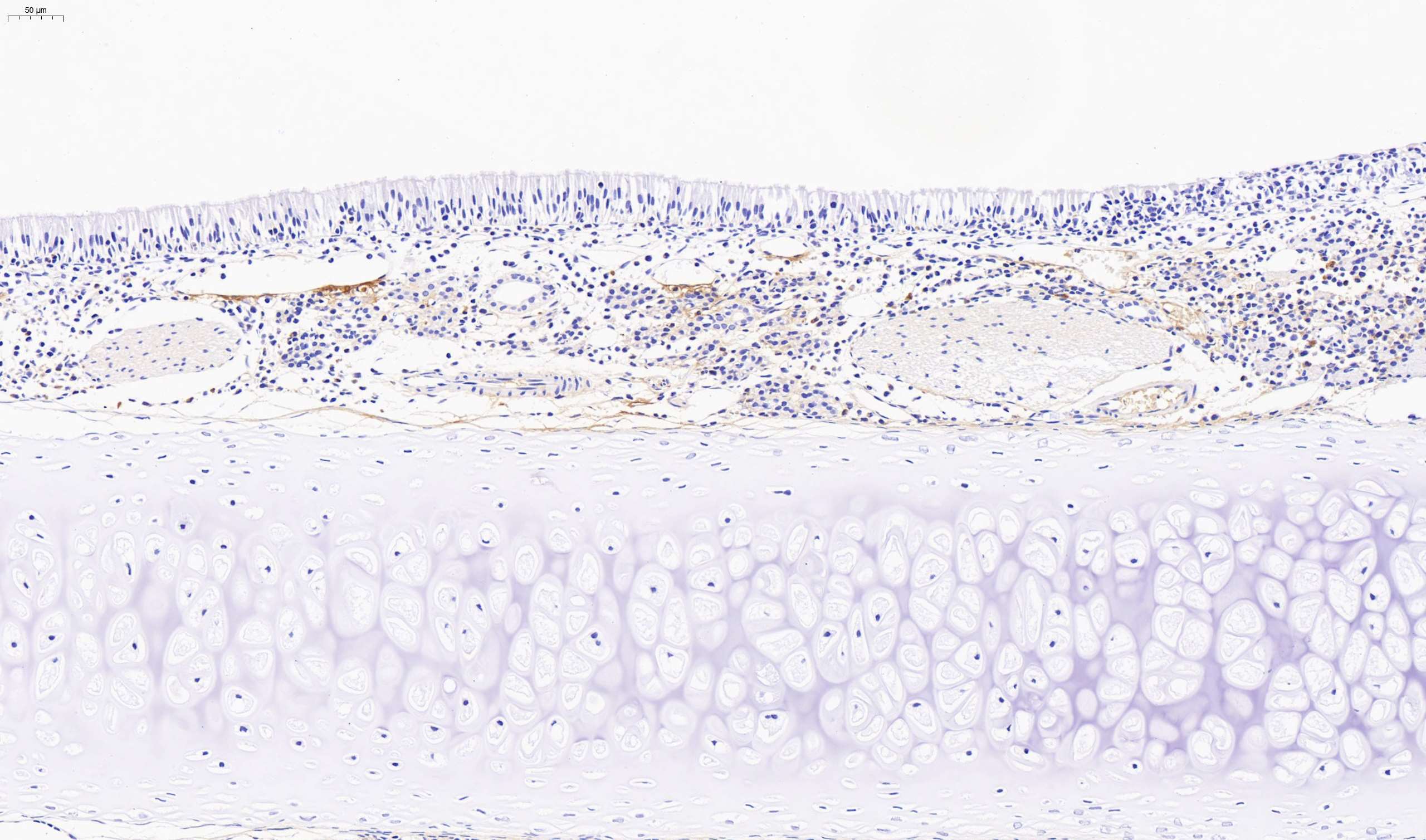

Supplement: Supplementary file 7 [file DataSheet5.ZIP › Microscopy images-Immunohistochemistry-GATA-3_200x_50um/Loratadine/3 GATA-3_200x_50um_1.jpeg]

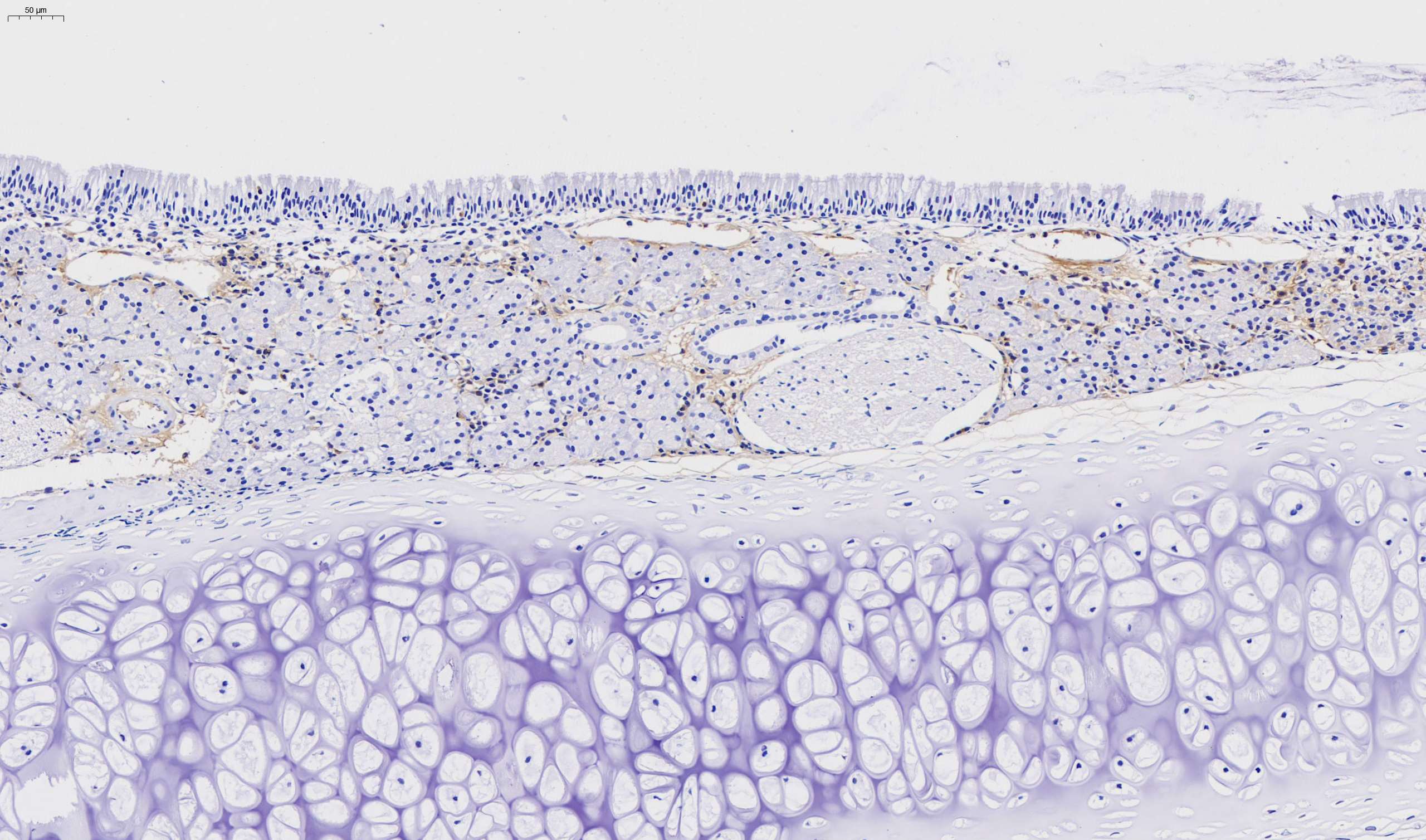

Supplement: Supplementary file 7 [file DataSheet5.ZIP › Microscopy images-Immunohistochemistry-GATA-3_200x_50um/Loratadine/4 GATA-3_200x_50um_1.jpeg]

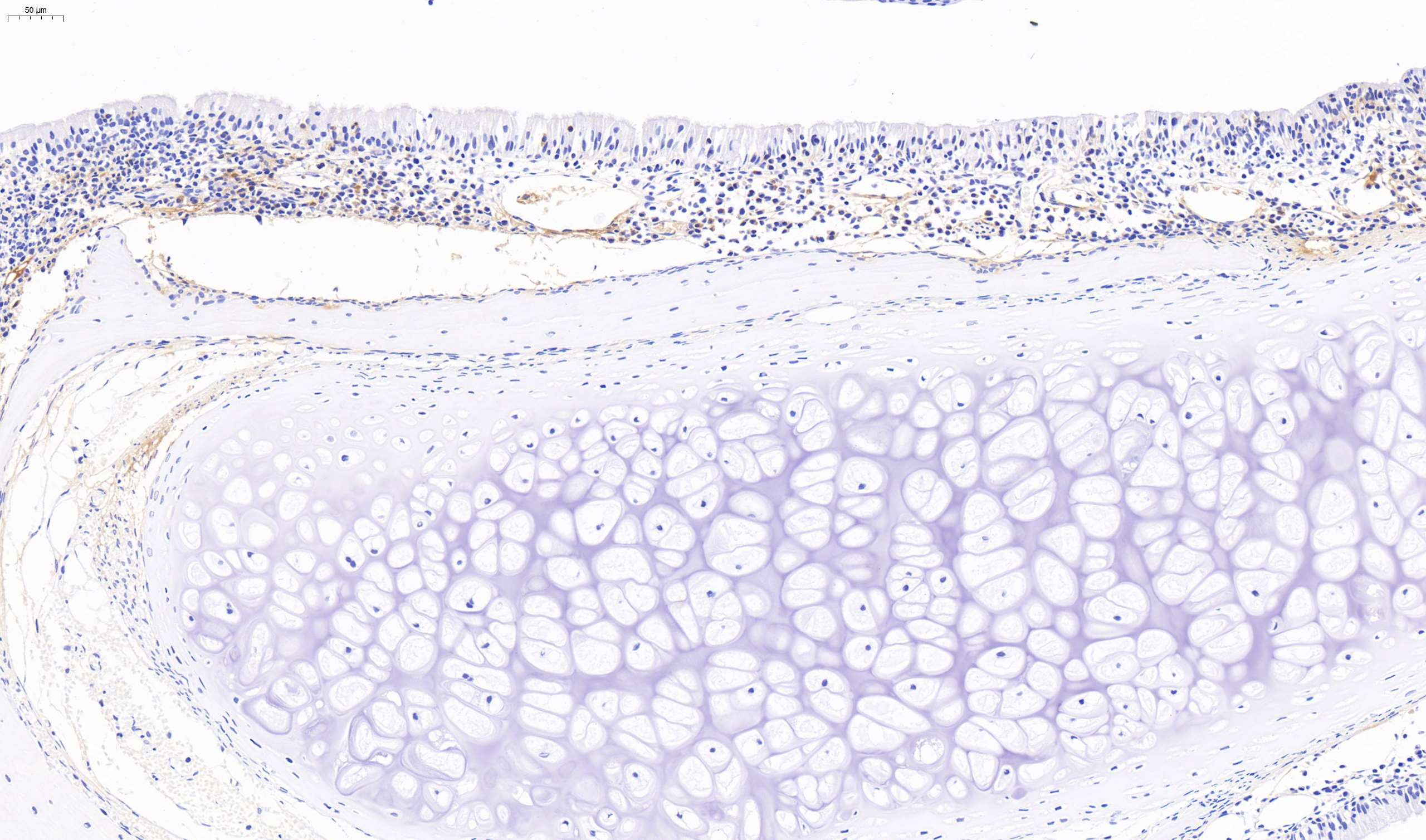

Supplement: Supplementary file 7 [file DataSheet5.ZIP › Microscopy images-Immunohistochemistry-GATA-3_200x_50um/Loratadine/5 GATA-3_200x_50um_1.jpeg]

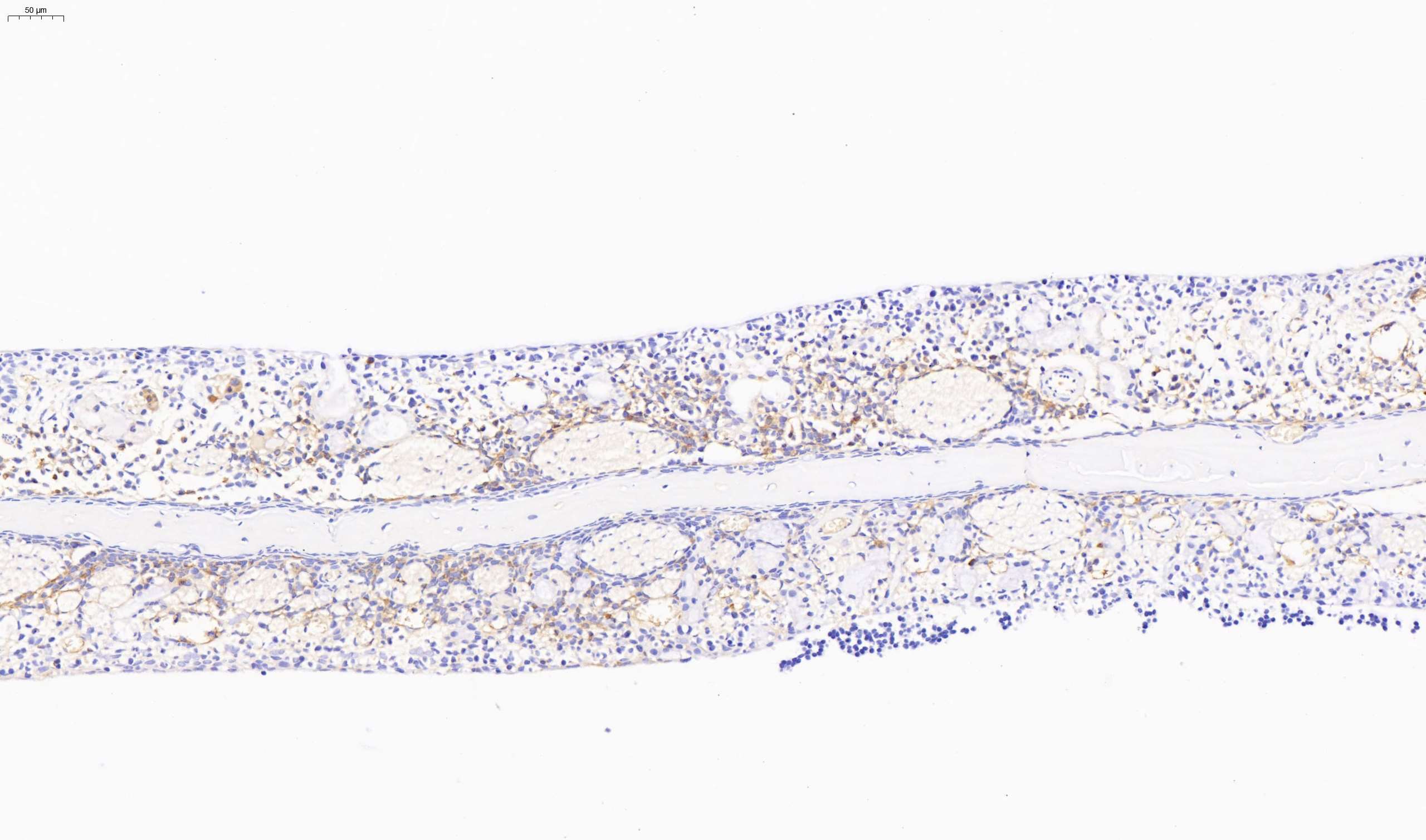

Supplement: Supplementary file 7 [file DataSheet5.ZIP › Microscopy images-Immunohistochemistry-GATA-3_200x_50um/Model/1 GATA3_200x_50um_1.jpeg]

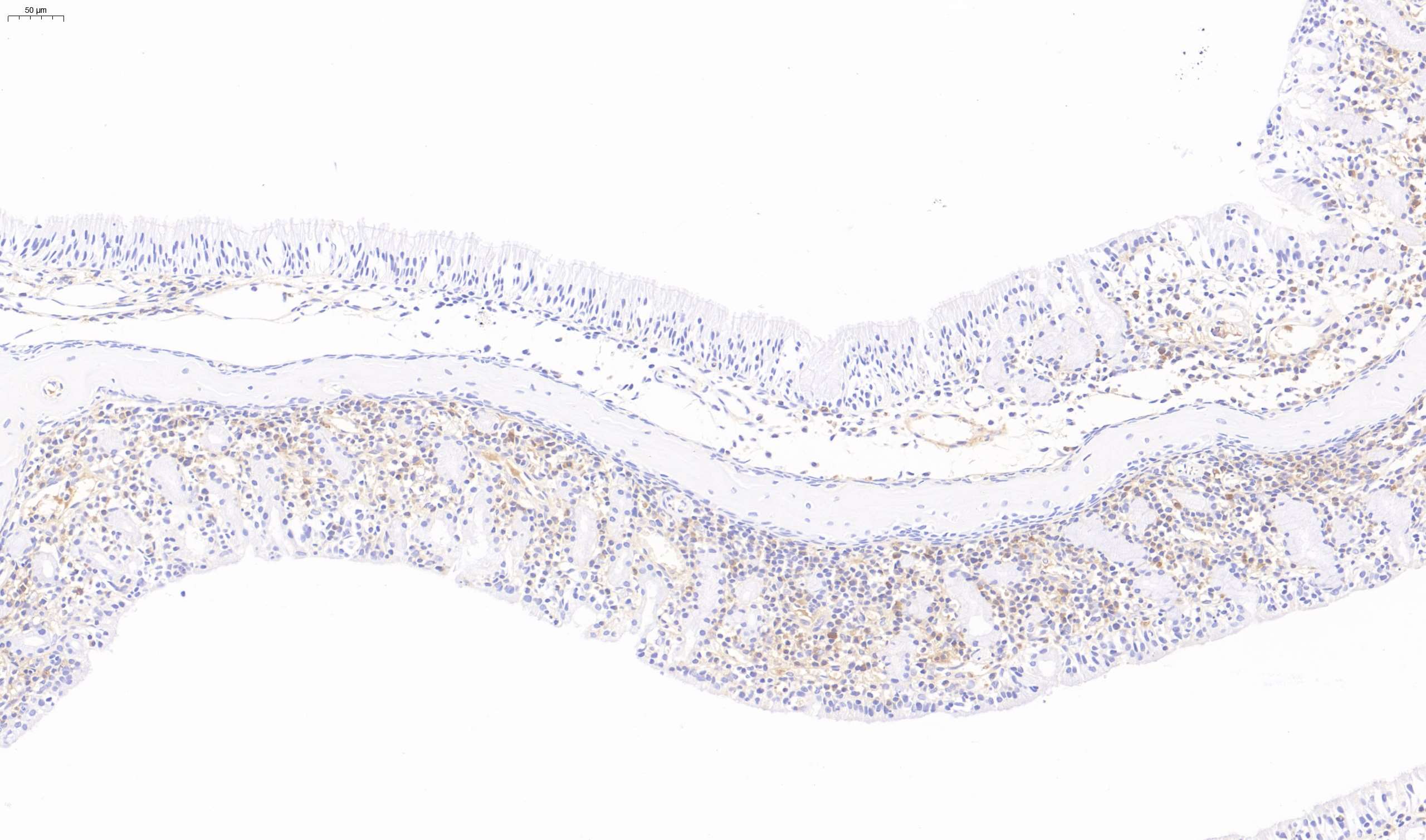

Supplement: Supplementary file 7 [file DataSheet5.ZIP › Microscopy images-Immunohistochemistry-GATA-3_200x_50um/Model/2 GATA-3_200x_50um_1.jpeg]

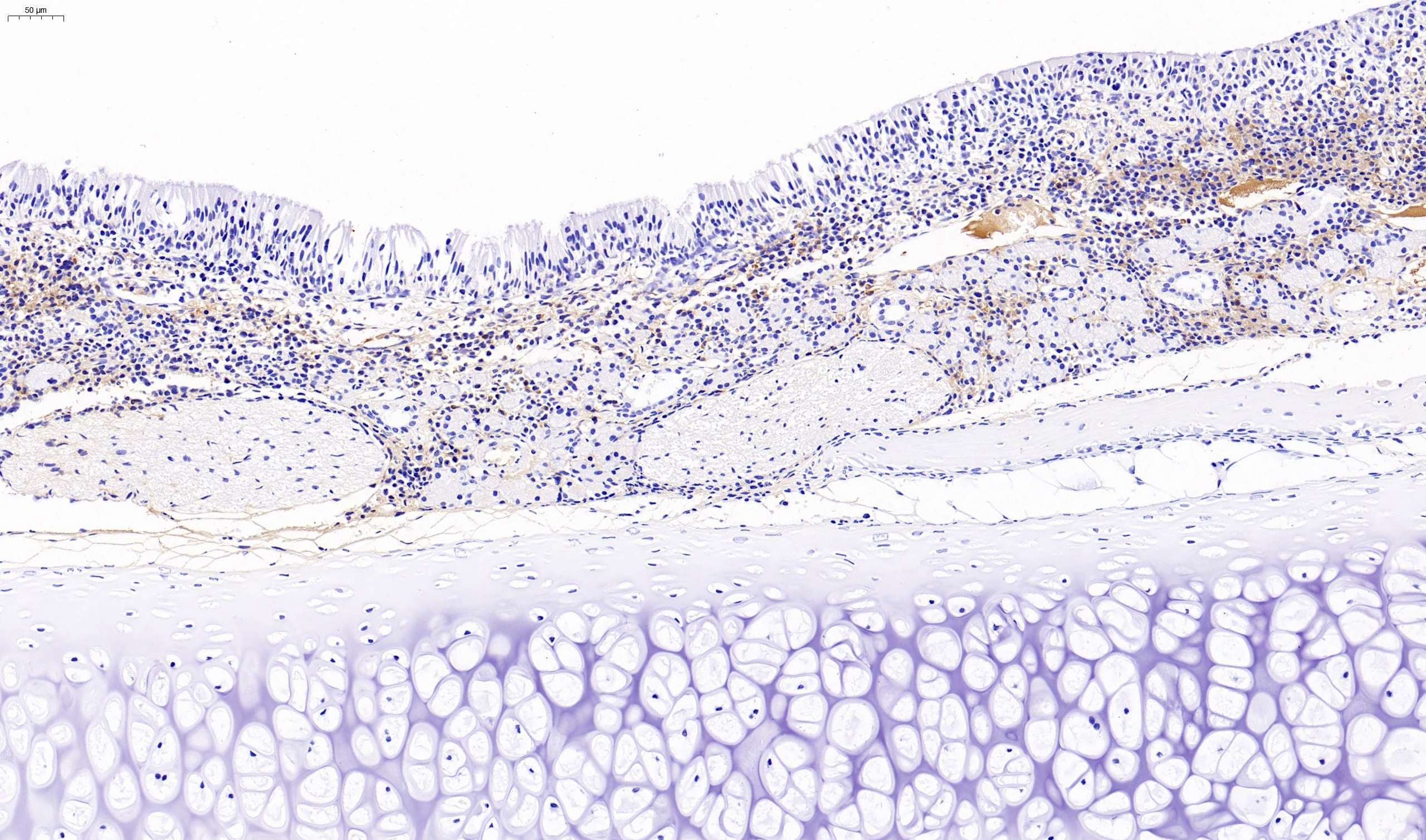

Supplement: Supplementary file 7 [file DataSheet5.ZIP › Microscopy images-Immunohistochemistry-GATA-3_200x_50um/Model/3 GATA-3_200x_50um_1.jpeg]

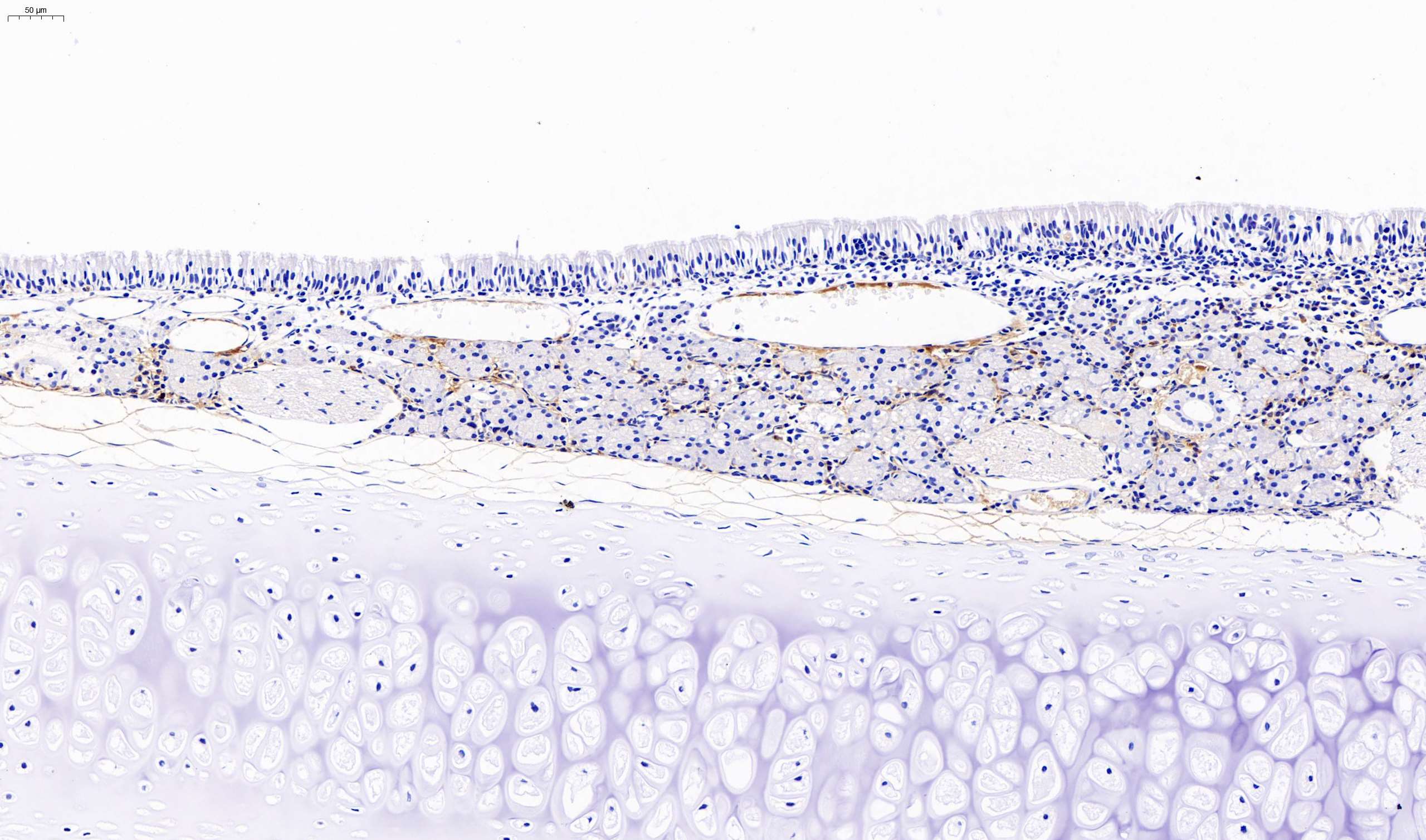

Supplement: Supplementary file 7 [file DataSheet5.ZIP › Microscopy images-Immunohistochemistry-GATA-3_200x_50um/Model/4 GATA-3_200x_50um_1.jpeg]

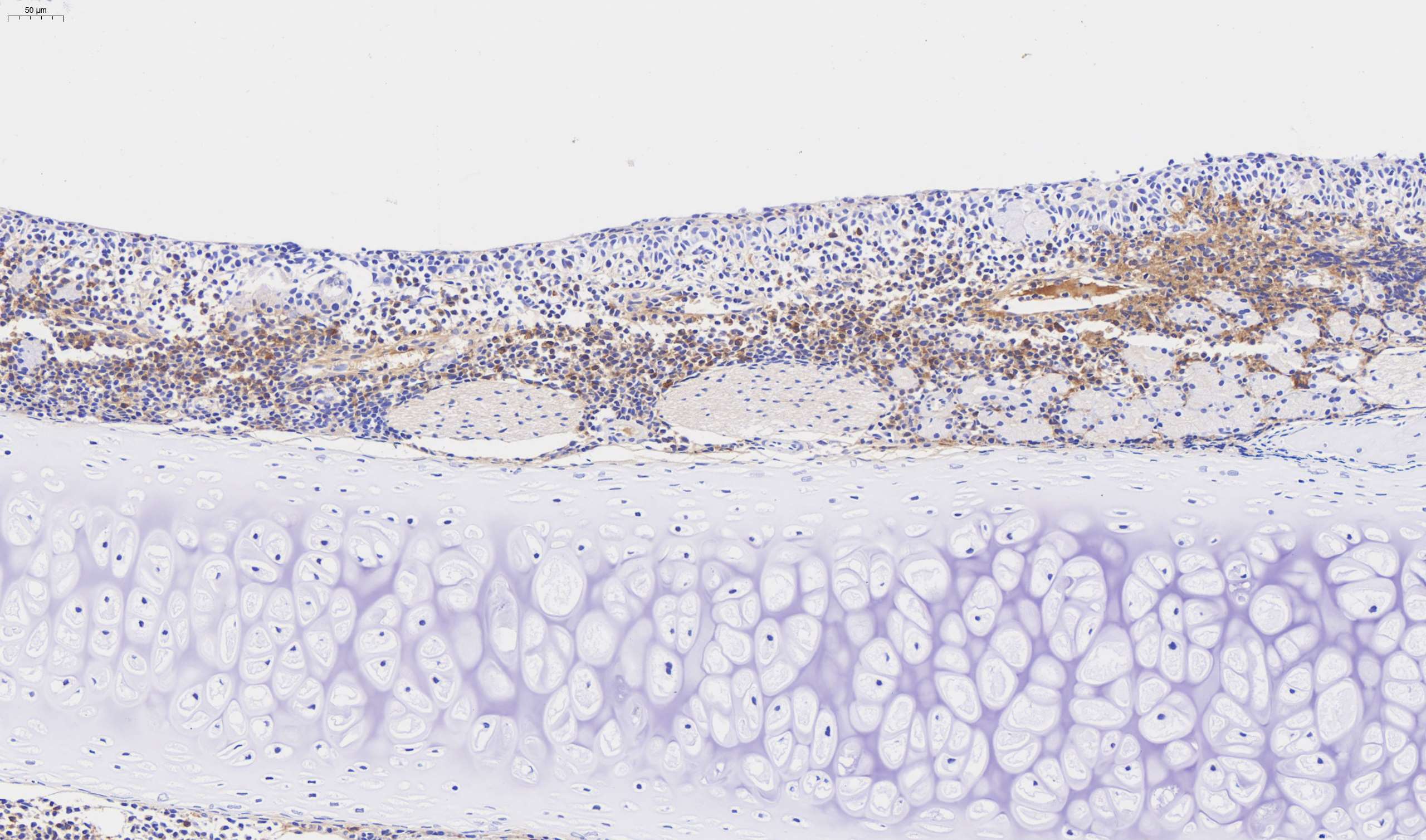

Supplement: Supplementary file 7 [file DataSheet5.ZIP › Microscopy images-Immunohistochemistry-GATA-3_200x_50um/Model/5 GATA-3_200x_50um_1.jpeg]

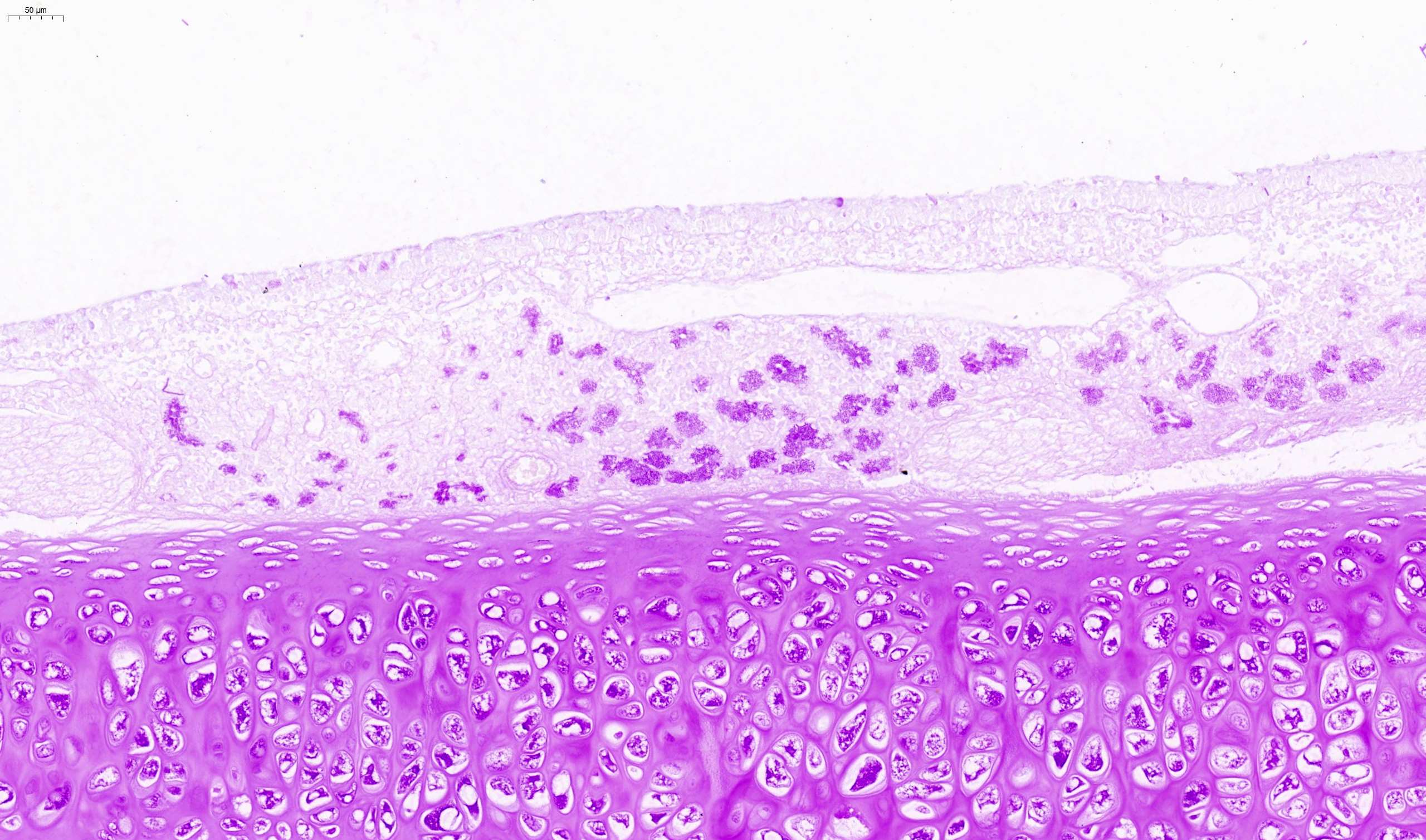

Supplement: Supplementary file 8 [file DataSheet7.ZIP › Microscopy images-PAS_200x_50um/CAVO-H/CAVO-H1 PAS_200x_50um_1.jpeg]

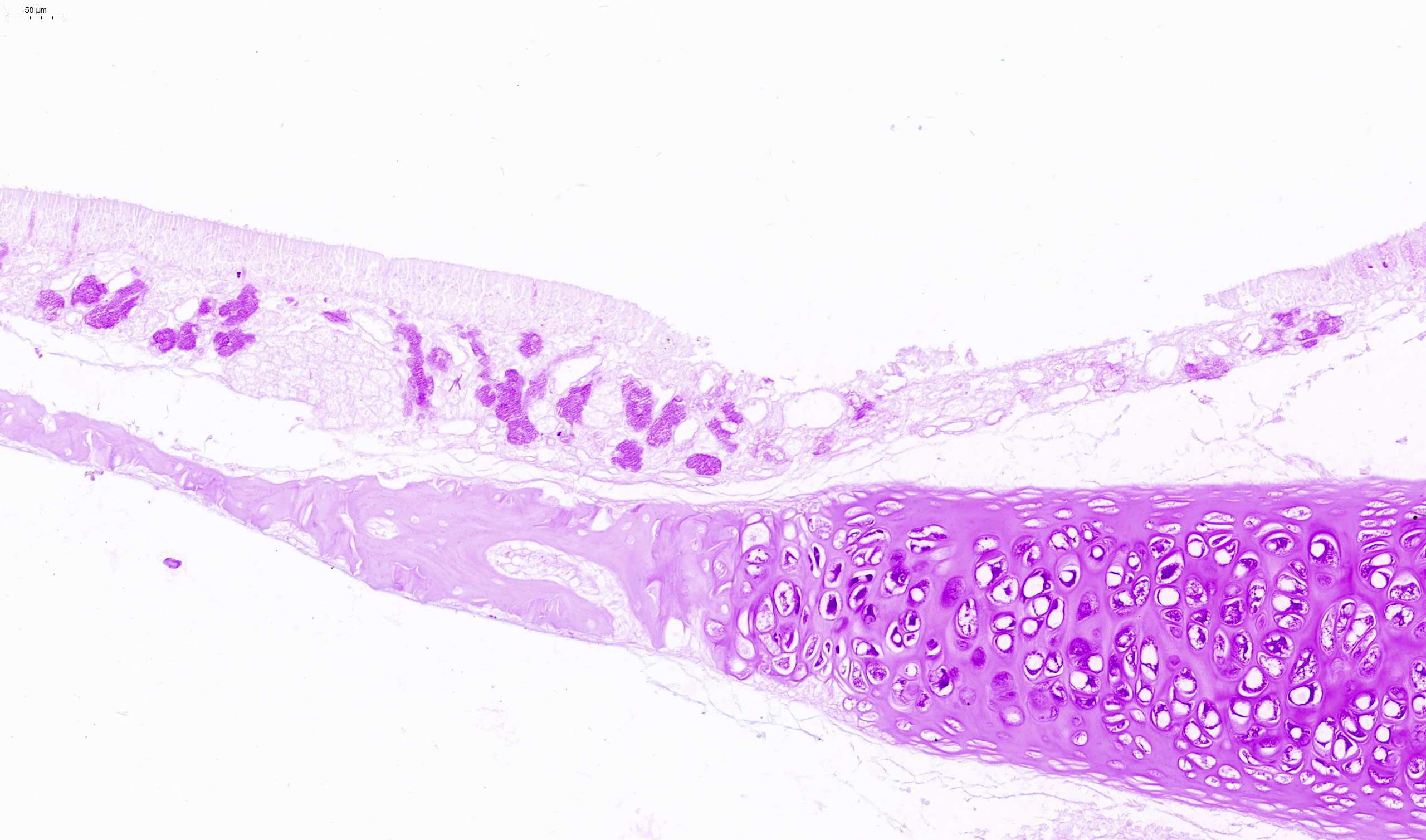

Supplement: Supplementary file 8 [file DataSheet7.ZIP › Microscopy images-PAS_200x_50um/CAVO-H/CAVO-H2 PAS_200x_50um_1.jpeg]

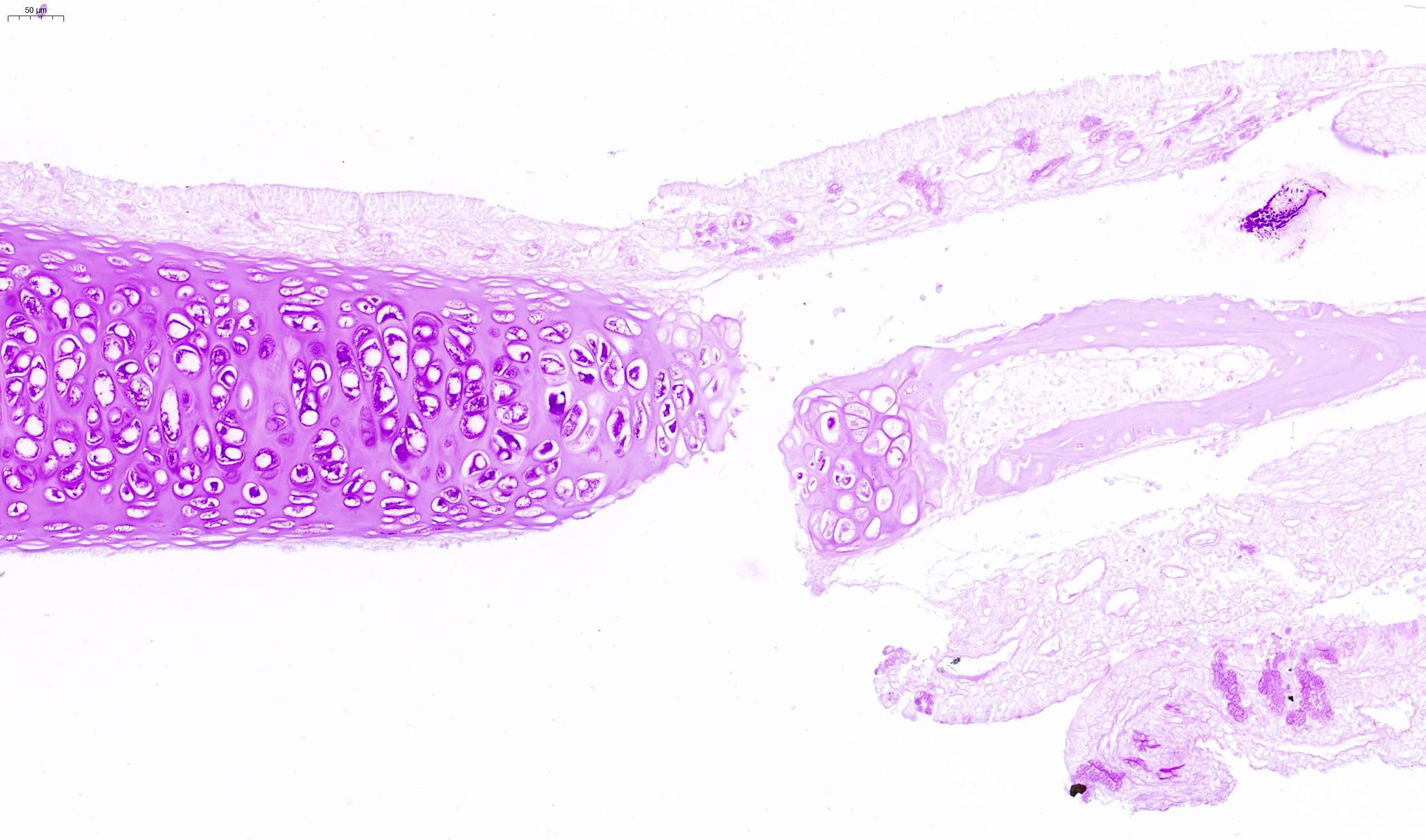

Supplement: Supplementary file 8 [file DataSheet7.ZIP › Microscopy images-PAS_200x_50um/CAVO-H/CAVO-H3 PAS_200x_50um_1.jpeg]

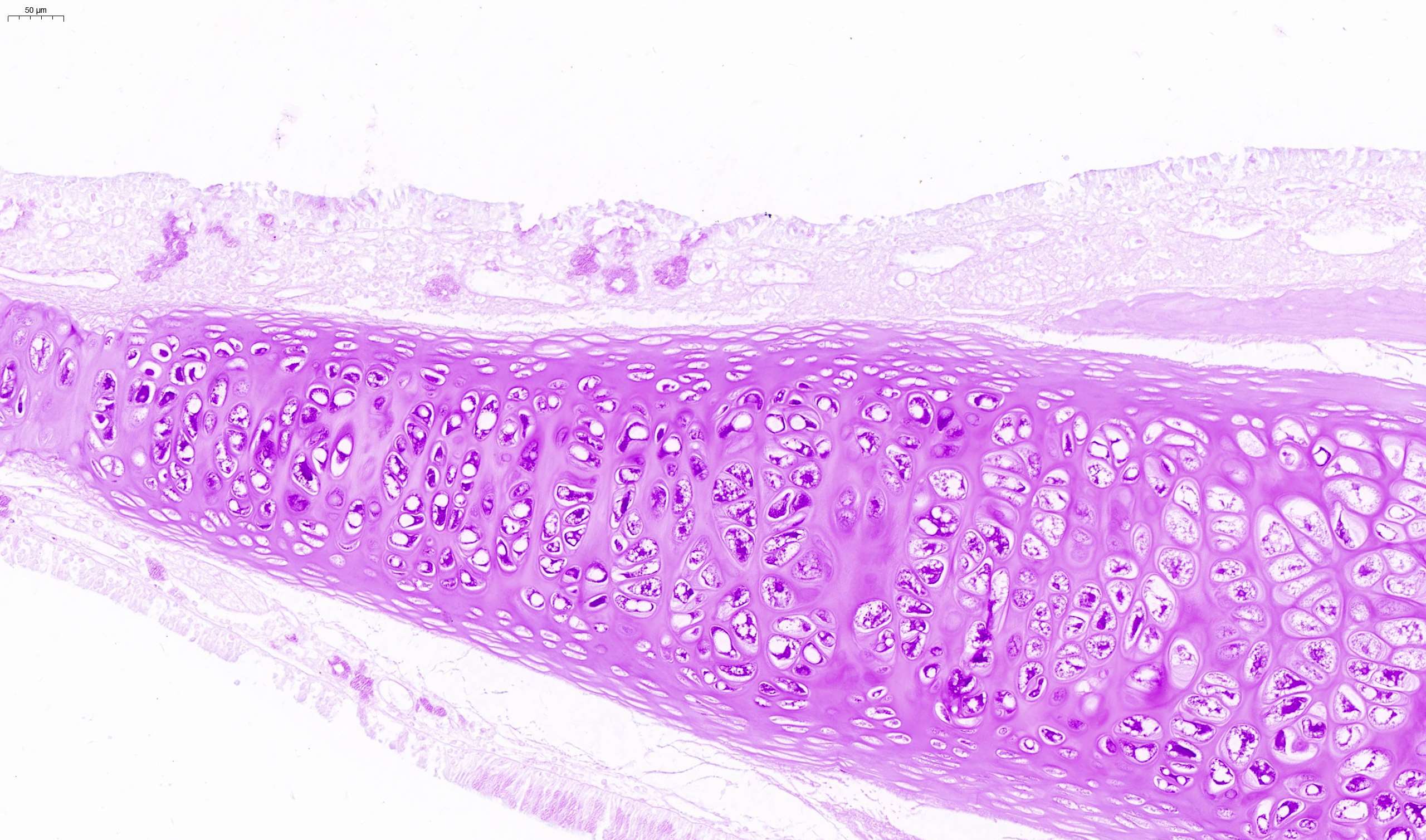

Supplement: Supplementary file 8 [file DataSheet7.ZIP › Microscopy images-PAS_200x_50um/CAVO-H/CAVO-H4 PAS_200x_50um_1.jpeg]

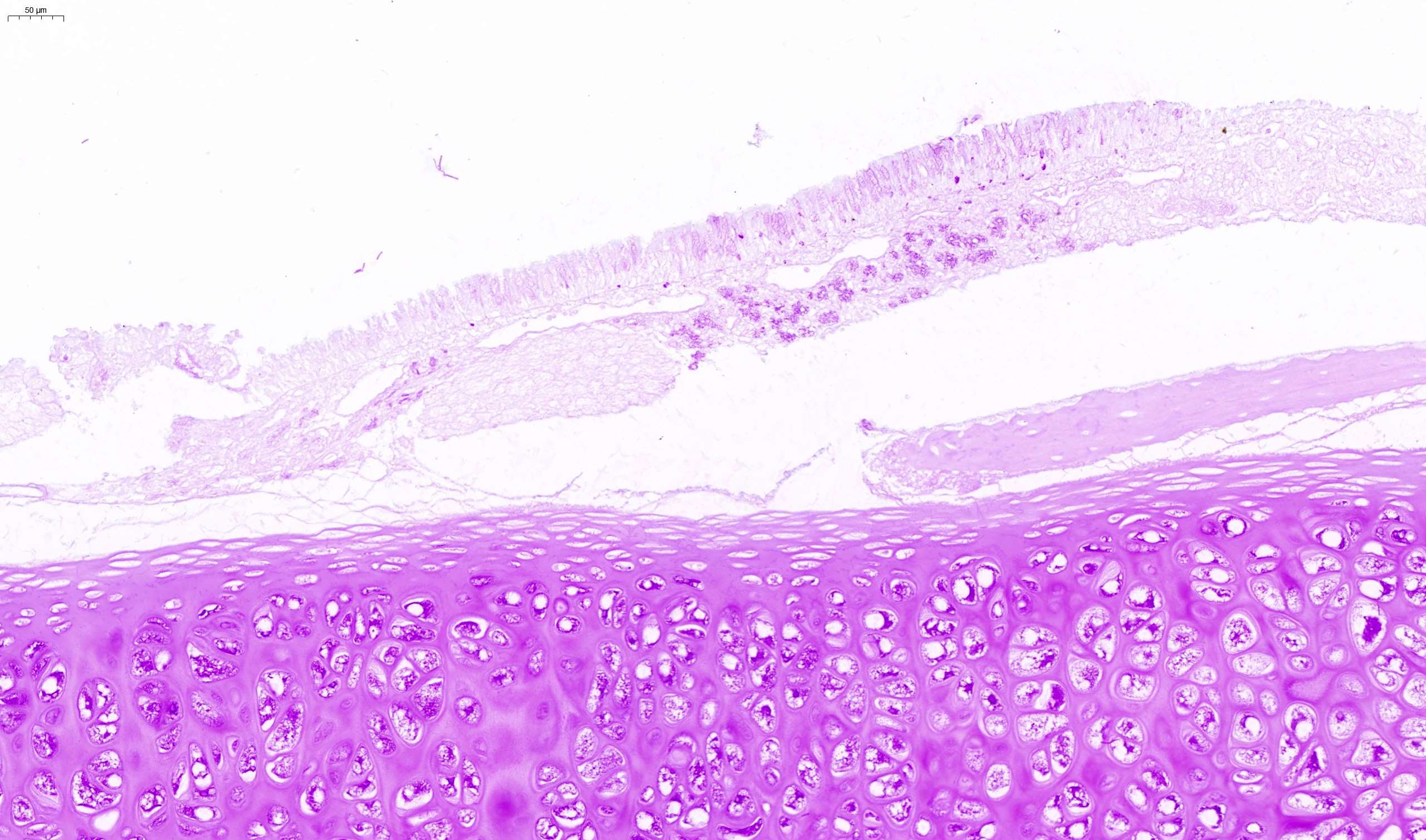

Supplement: Supplementary file 8 [file DataSheet7.ZIP › Microscopy images-PAS_200x_50um/CAVO-H/CAVO-H5 PAS_200x_50um_1.jpeg]

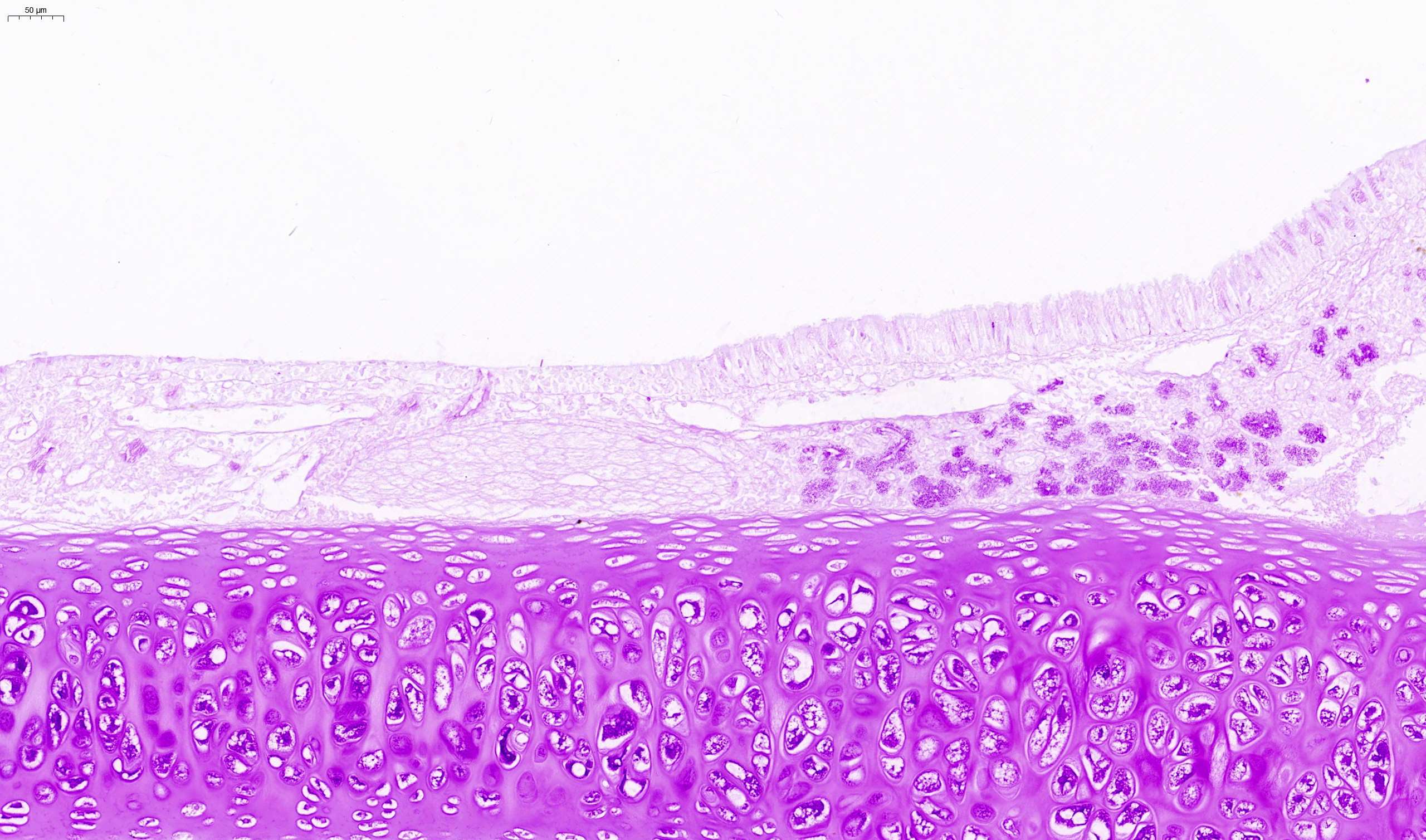

Supplement: Supplementary file 8 [file DataSheet7.ZIP › Microscopy images-PAS_200x_50um/CAVO-L/CAVO-L1 PAS_200x_50um_1.jpeg]

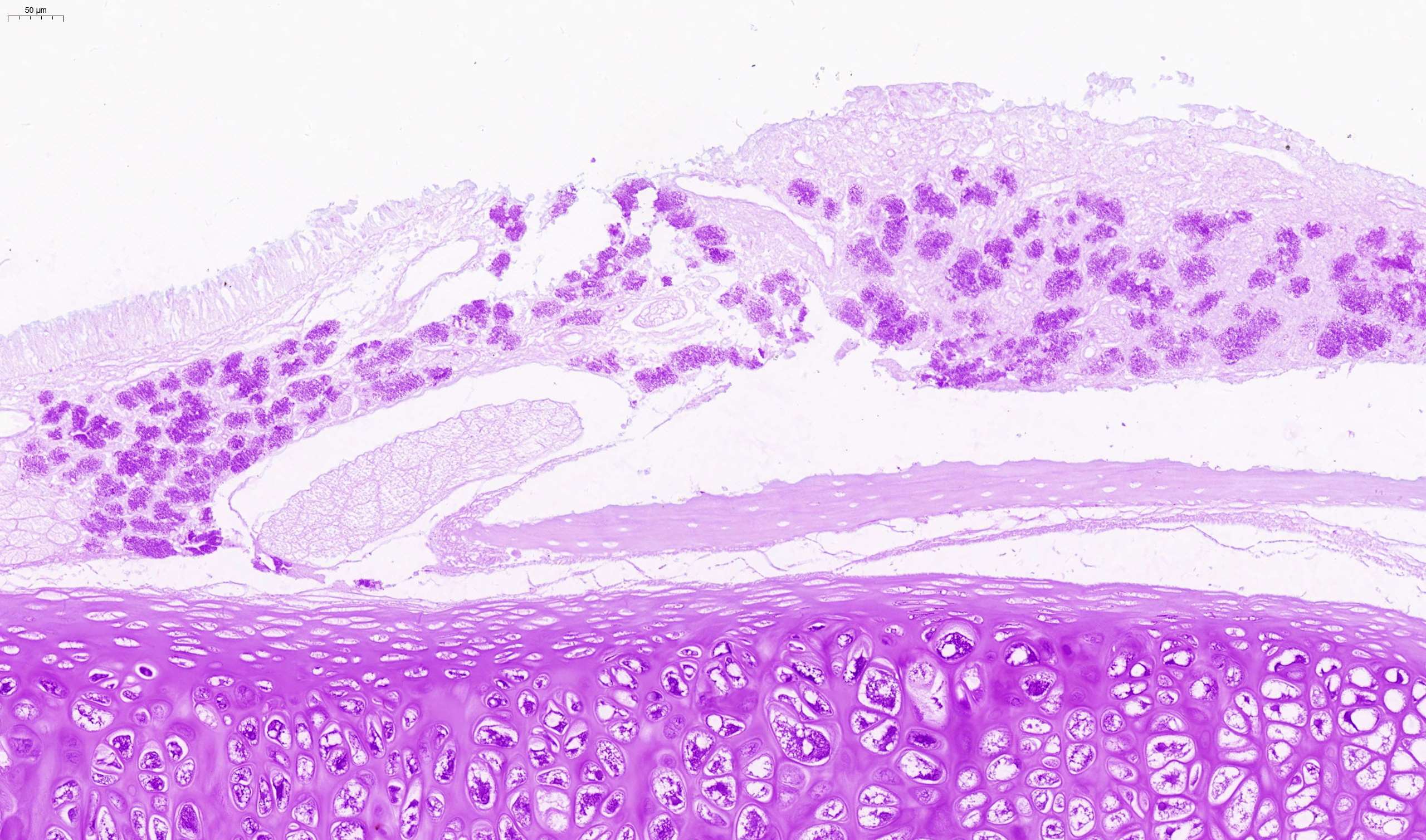

Supplement: Supplementary file 8 [file DataSheet7.ZIP › Microscopy images-PAS_200x_50um/CAVO-L/CAVO-L2 PAS_200x_50um_1.jpeg]

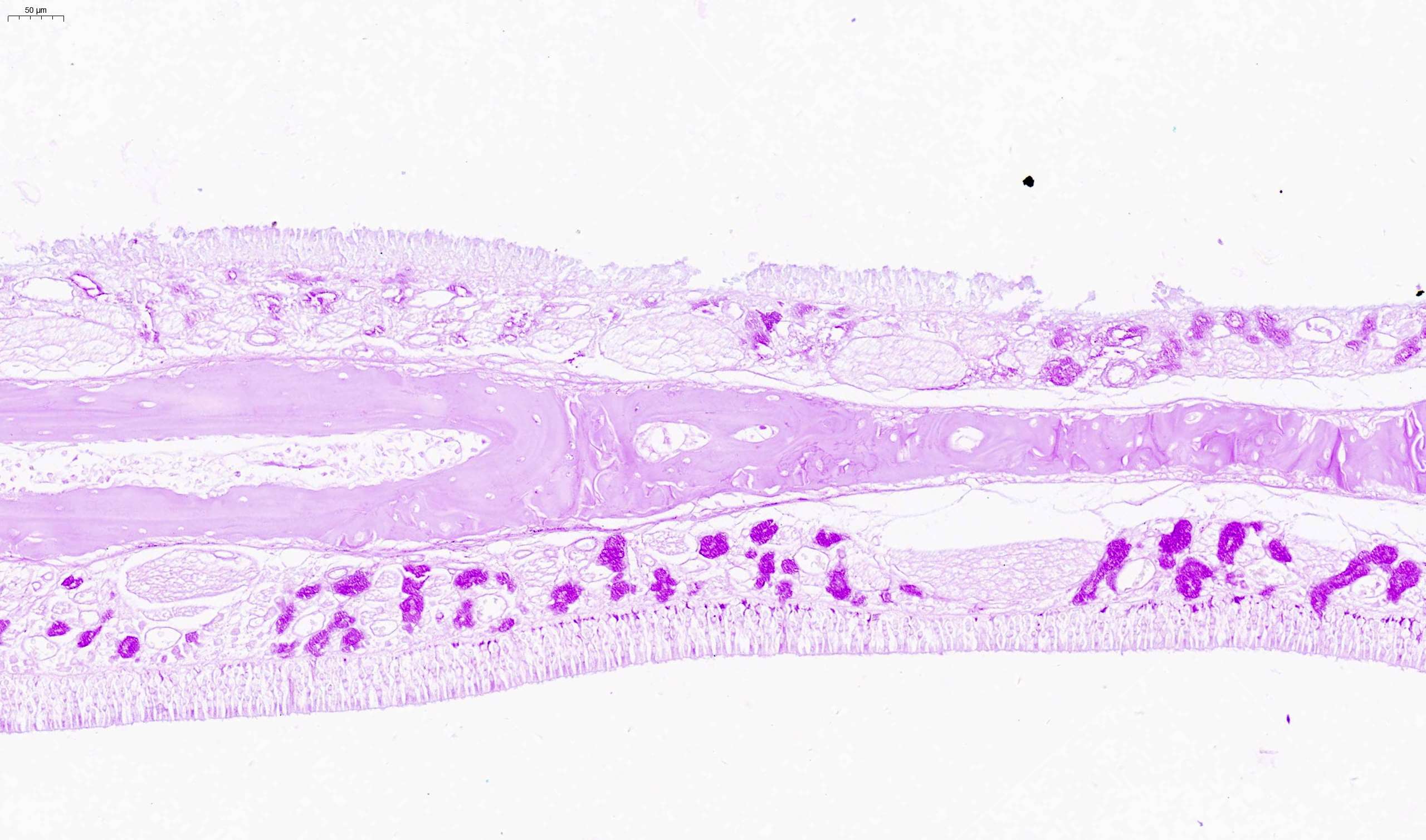

Supplement: Supplementary file 8 [file DataSheet7.ZIP › Microscopy images-PAS_200x_50um/CAVO-L/CAVO-L3 PAS_200x_50um_1.jpeg]

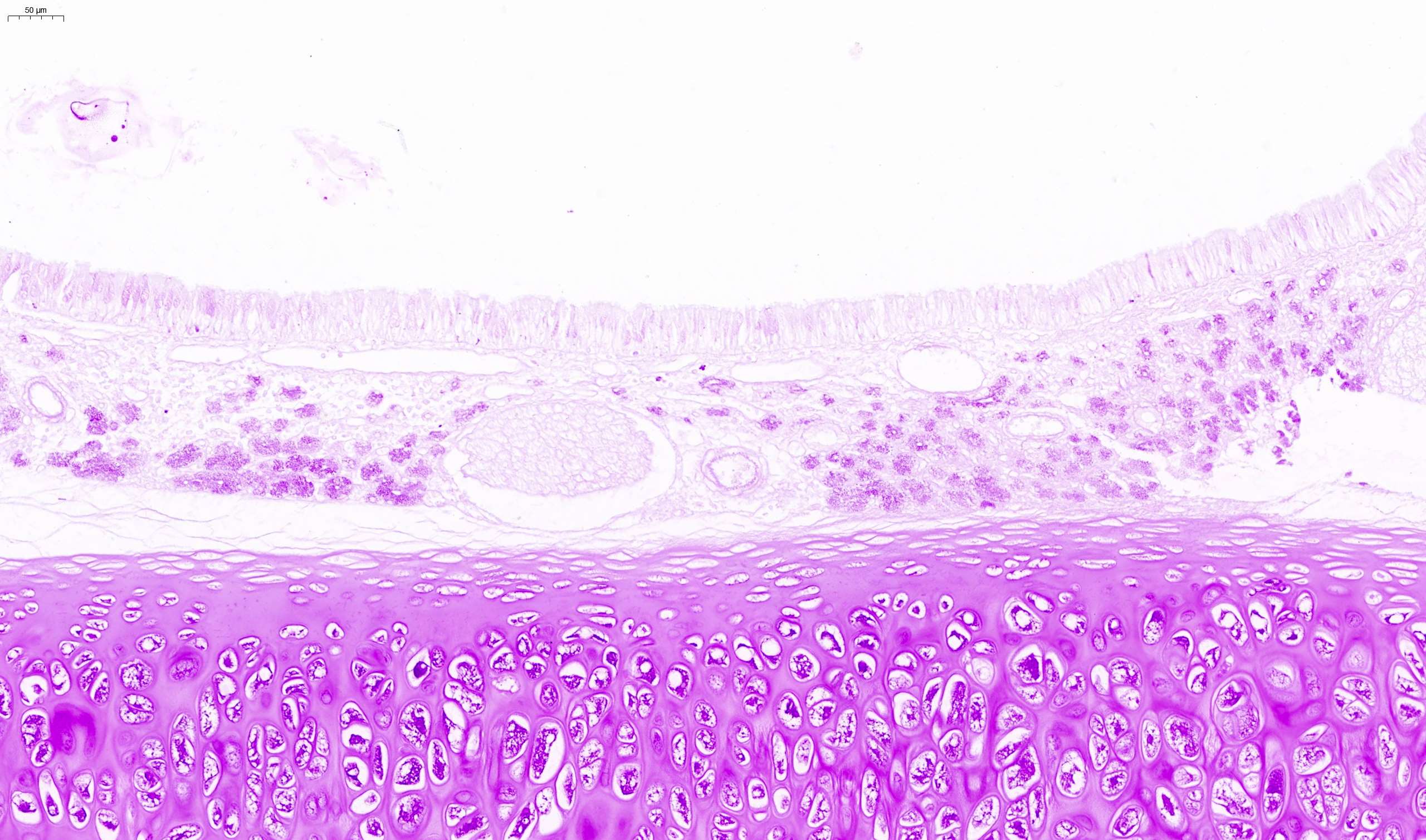

Supplement: Supplementary file 8 [file DataSheet7.ZIP › Microscopy images-PAS_200x_50um/CAVO-L/CAVO-L4 PAS_200x_50um_1.jpeg]

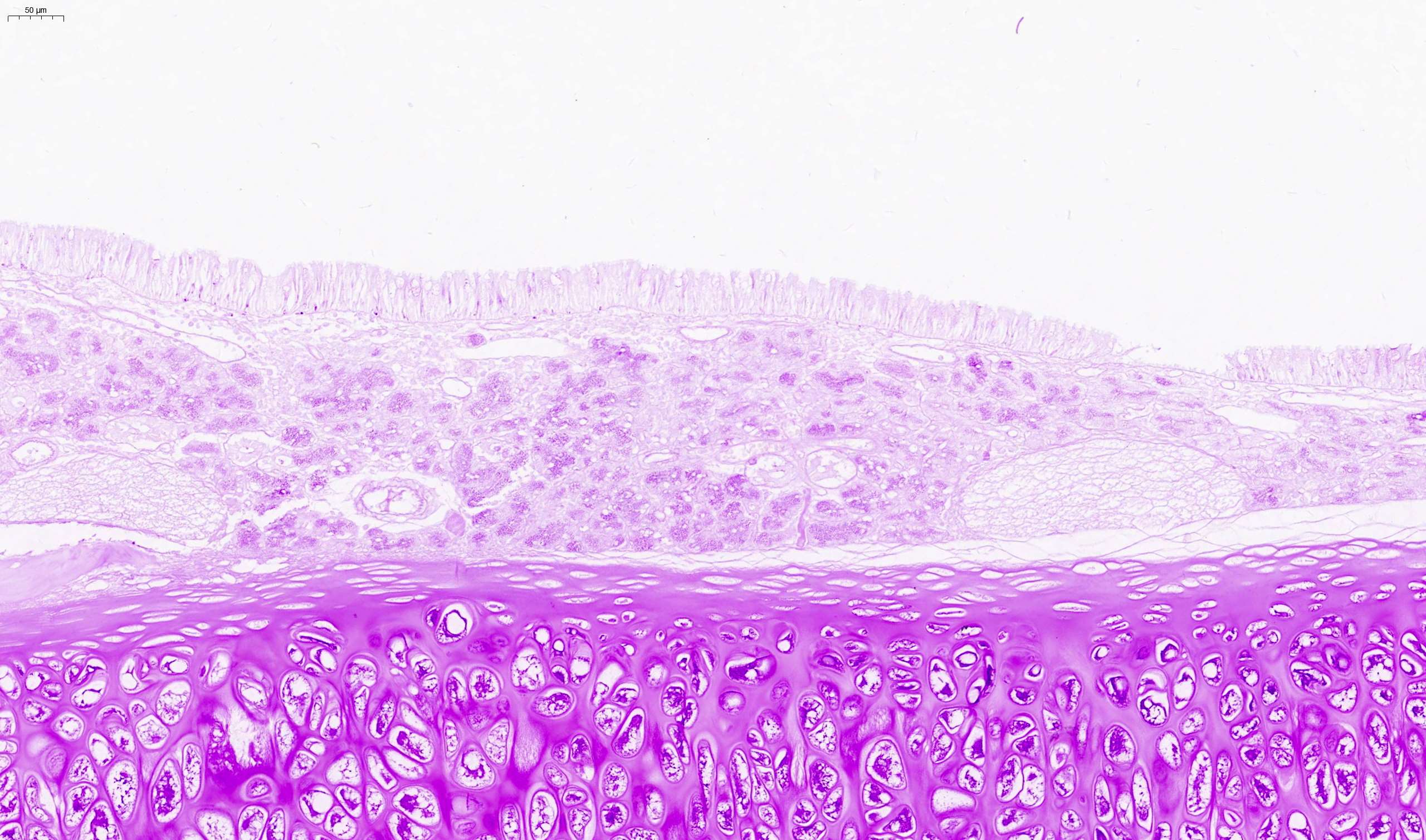

Supplement: Supplementary file 8 [file DataSheet7.ZIP › Microscopy images-PAS_200x_50um/CAVO-L/CAVO-L5 PAS_200x_50um_1.jpeg]

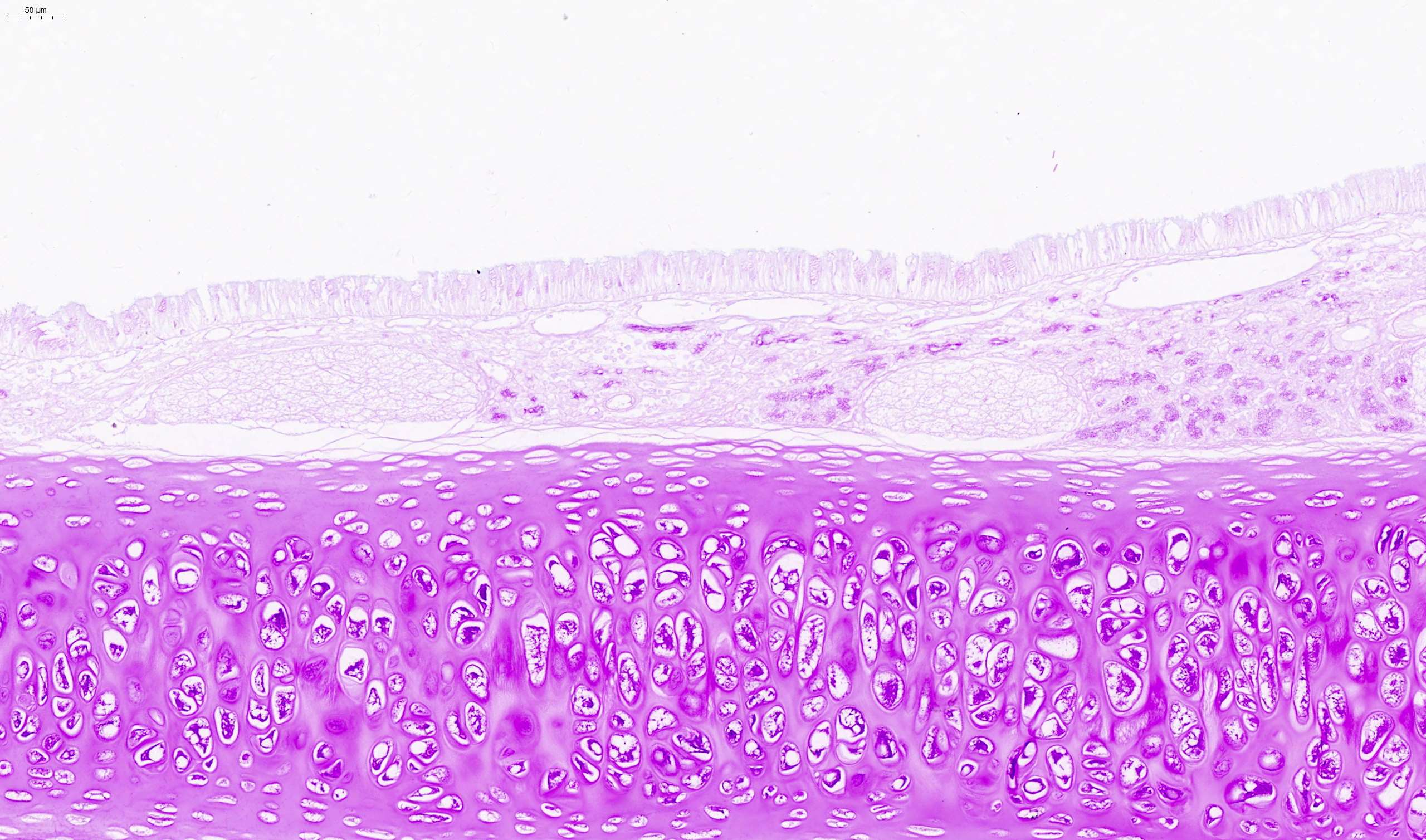

Supplement: Supplementary file 8 [file DataSheet7.ZIP › Microscopy images-PAS_200x_50um/CAVO-M/CAVO-M1 PAS_200x_50um_1.jpeg]

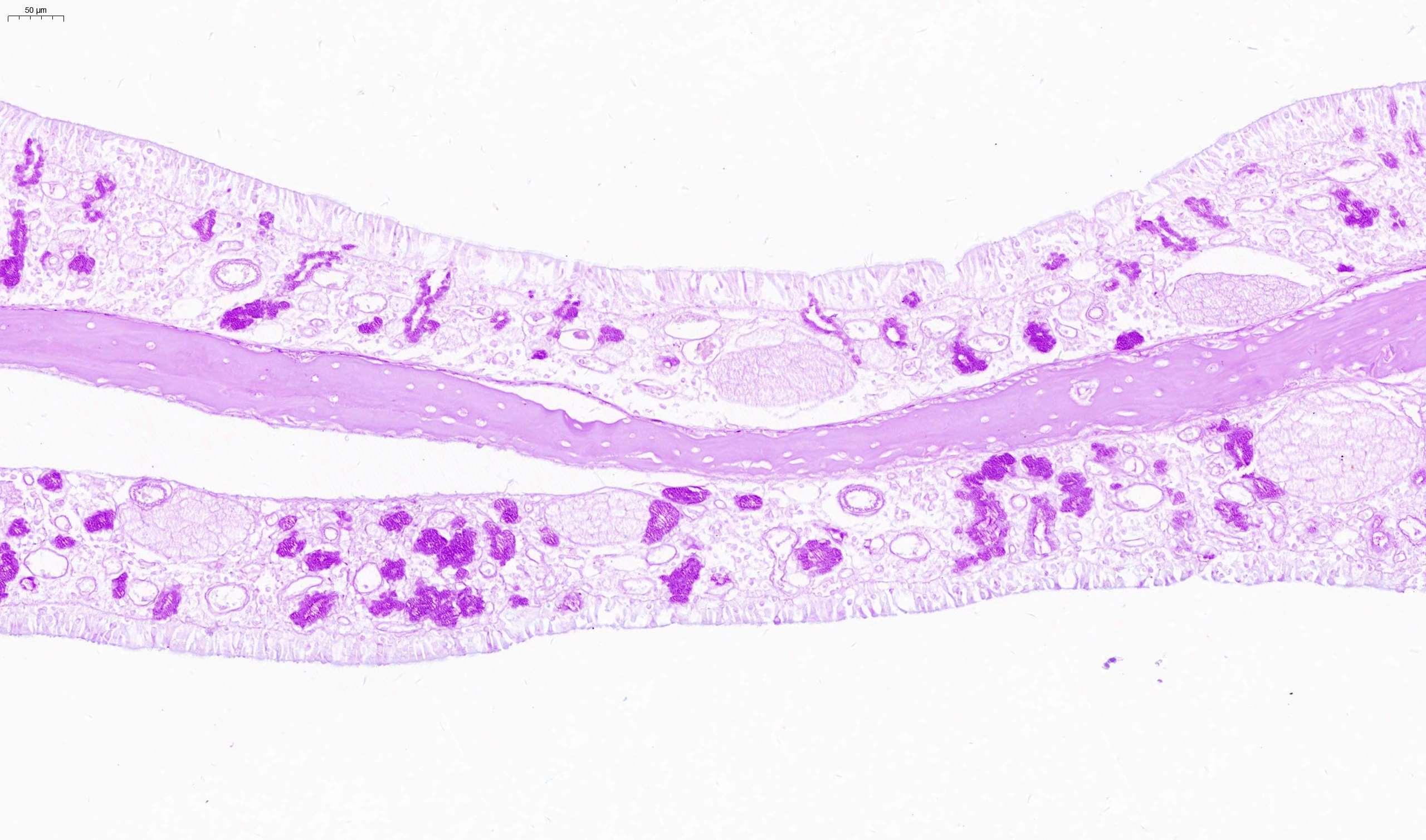

Supplement: Supplementary file 8 [file DataSheet7.ZIP › Microscopy images-PAS_200x_50um/CAVO-M/CAVO-M2 PAS_200x_50um_1.jpeg]

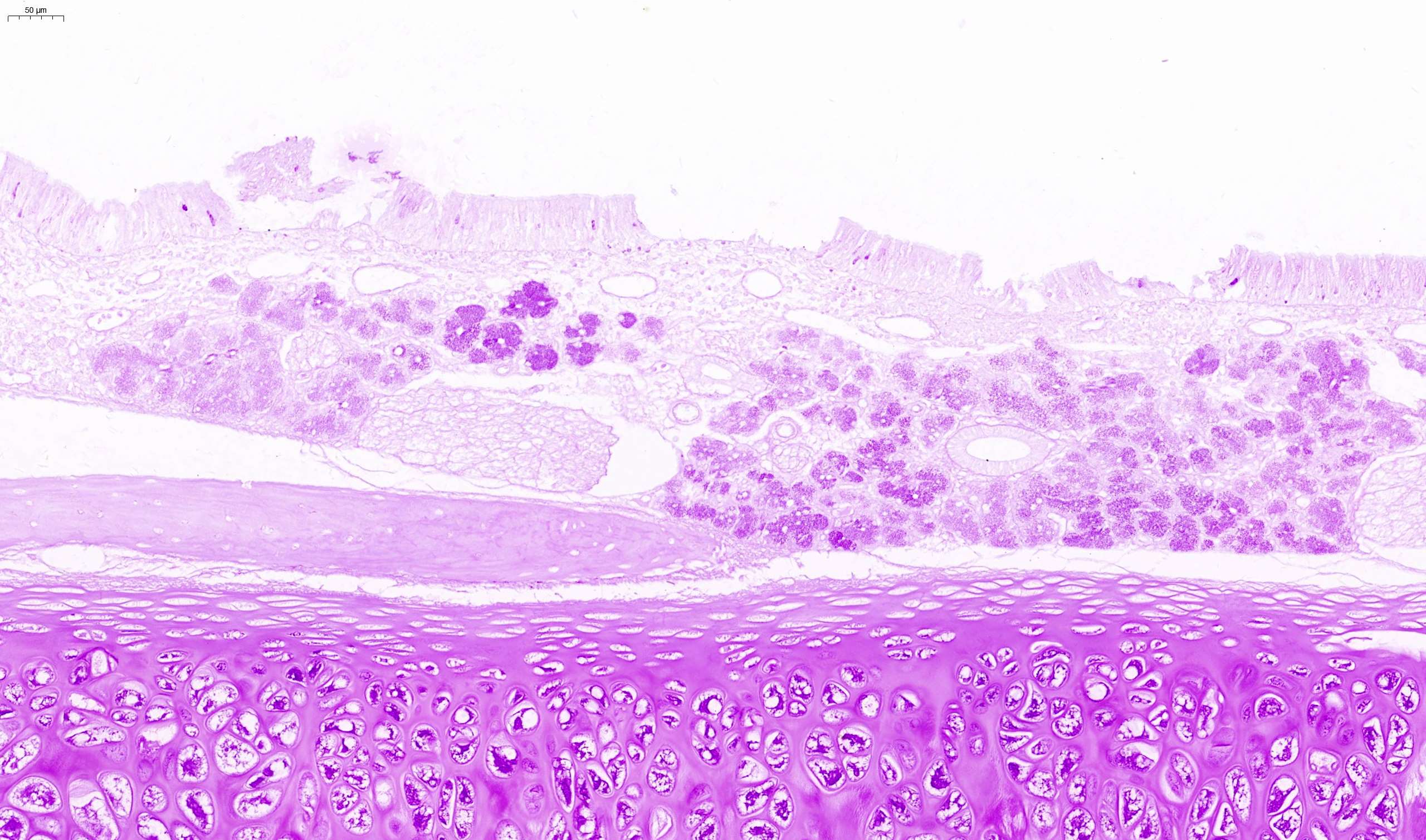

Supplement: Supplementary file 8 [file DataSheet7.ZIP › Microscopy images-PAS_200x_50um/CAVO-M/CAVO-M3 PAS_200x_50um_1.jpeg]

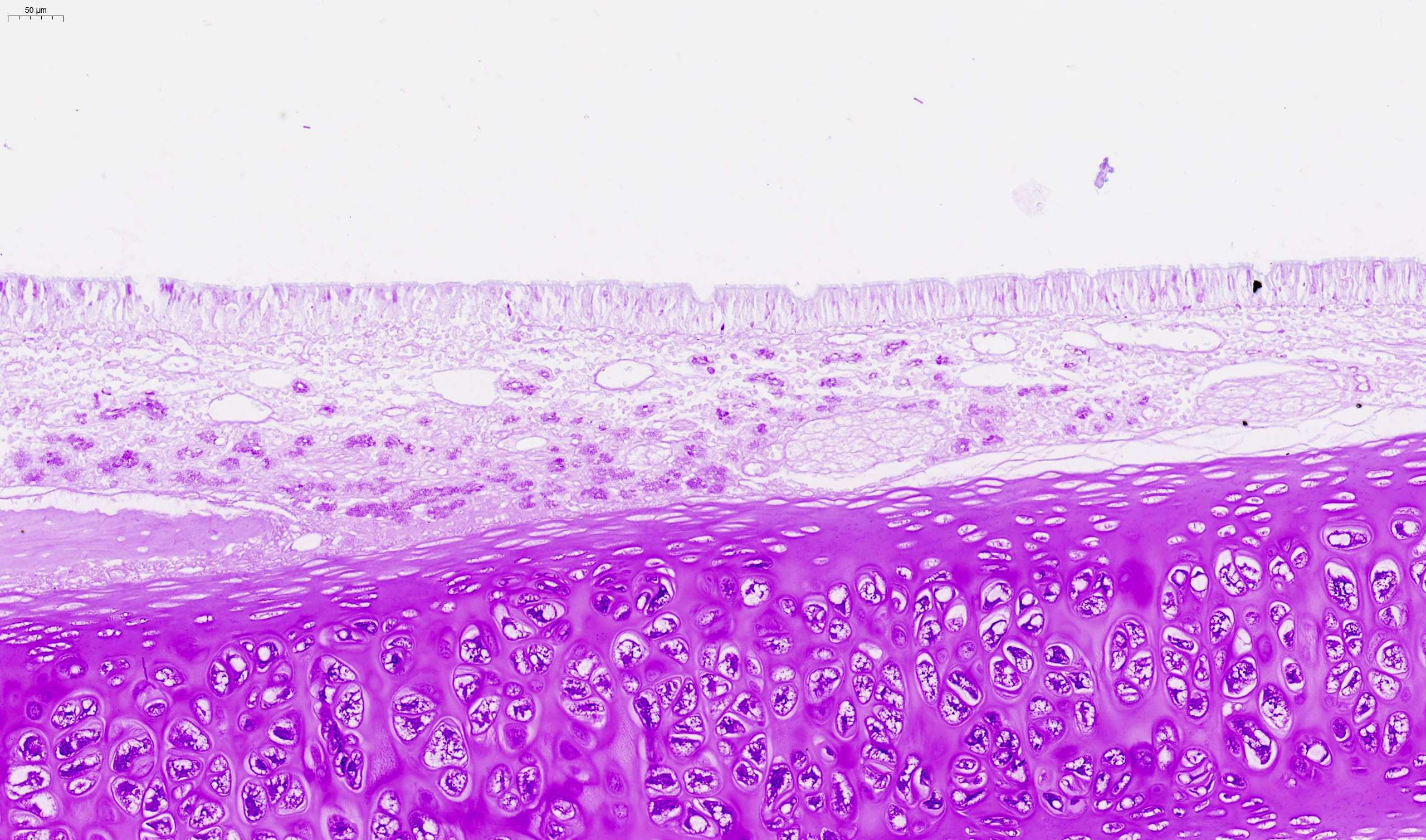

Supplement: Supplementary file 8 [file DataSheet7.ZIP › Microscopy images-PAS_200x_50um/CAVO-M/CAVO-M4 PAS_200x_50um_1.jpeg]

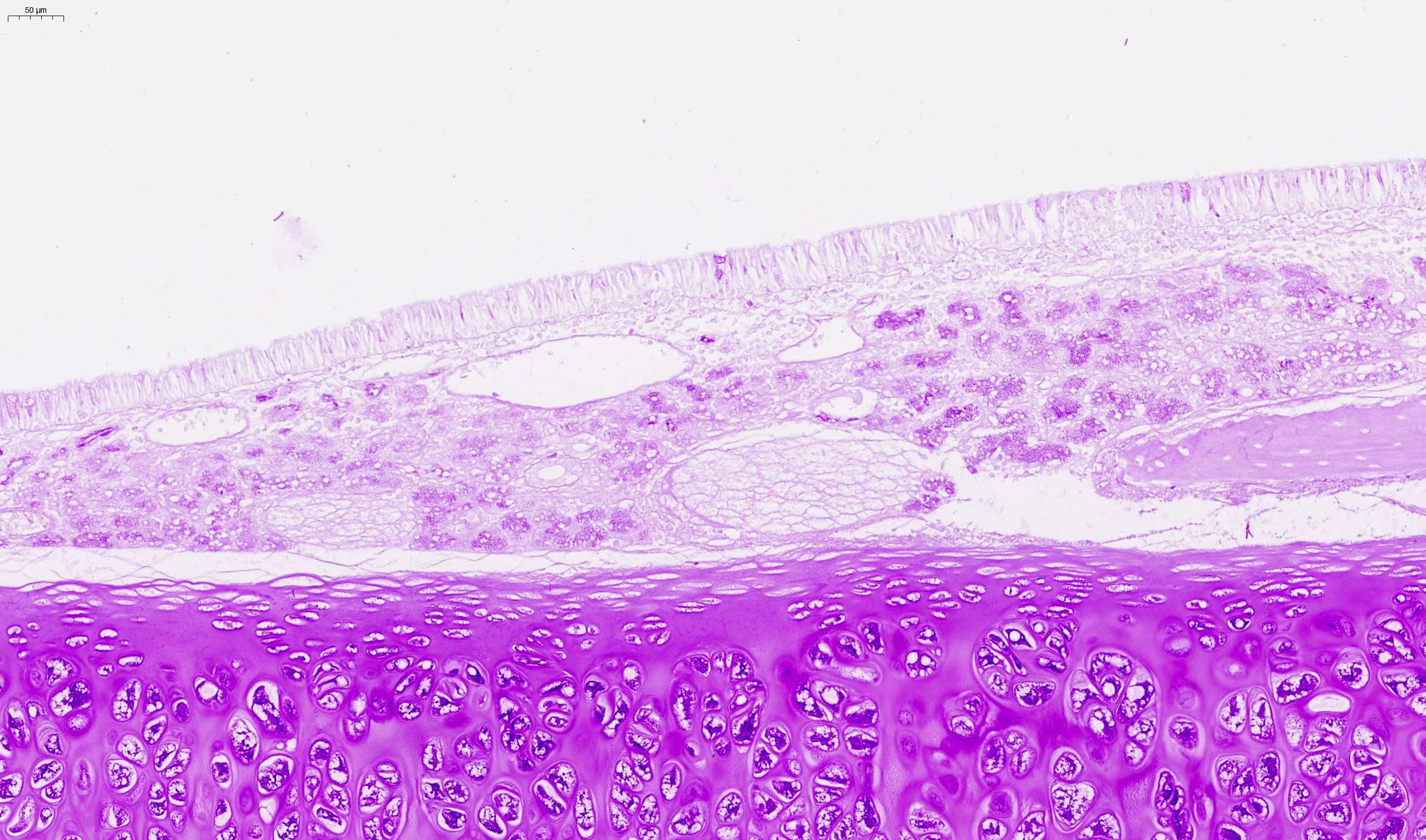

Supplement: Supplementary file 8 [file DataSheet7.ZIP › Microscopy images-PAS_200x_50um/CAVO-M/CAVO-M5 PAS_200x_50um_1.jpeg]

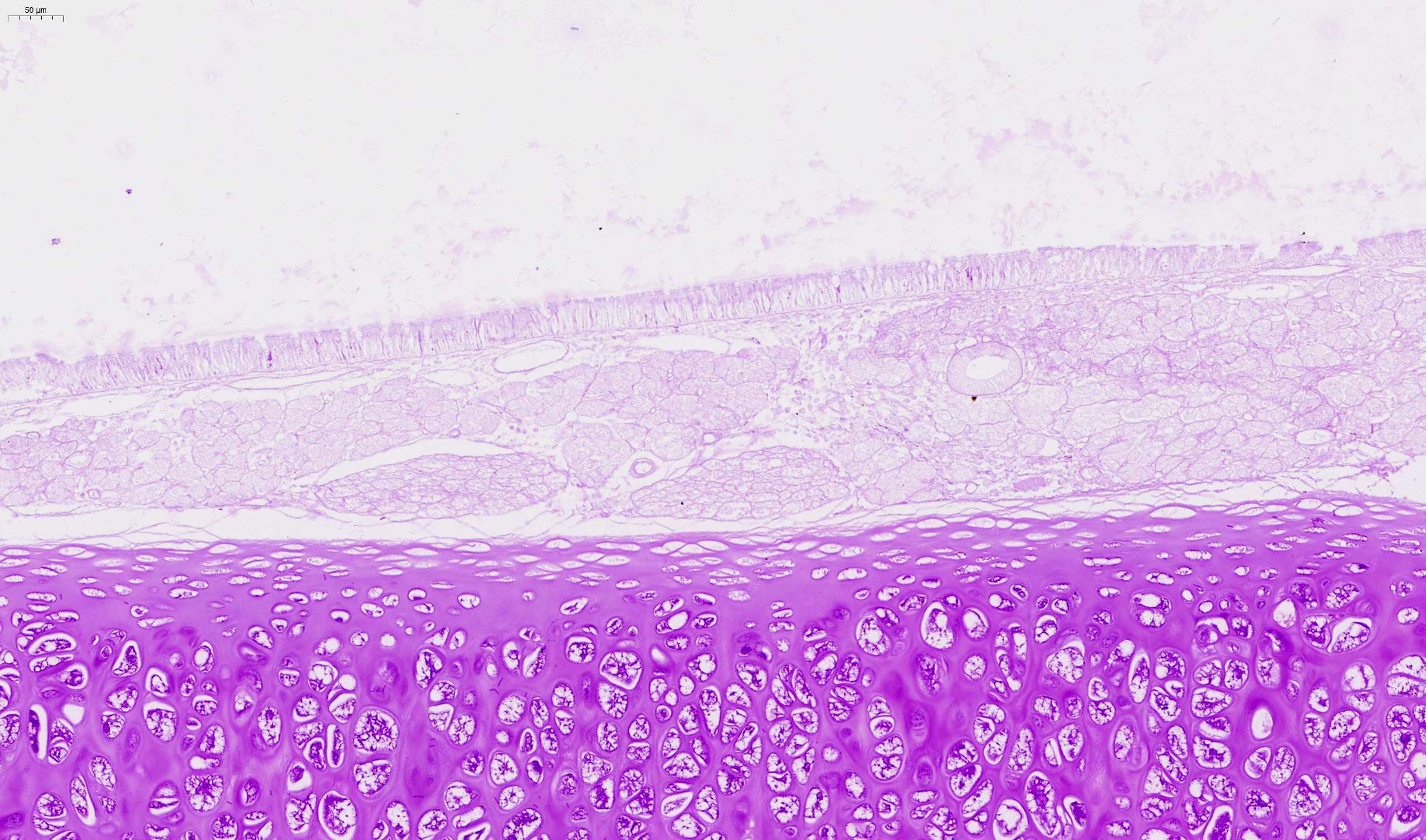

Supplement: Supplementary file 8 [file DataSheet7.ZIP › Microscopy images-PAS_200x_50um/Control/Control1 PAS_200x_50um_1.jpeg]

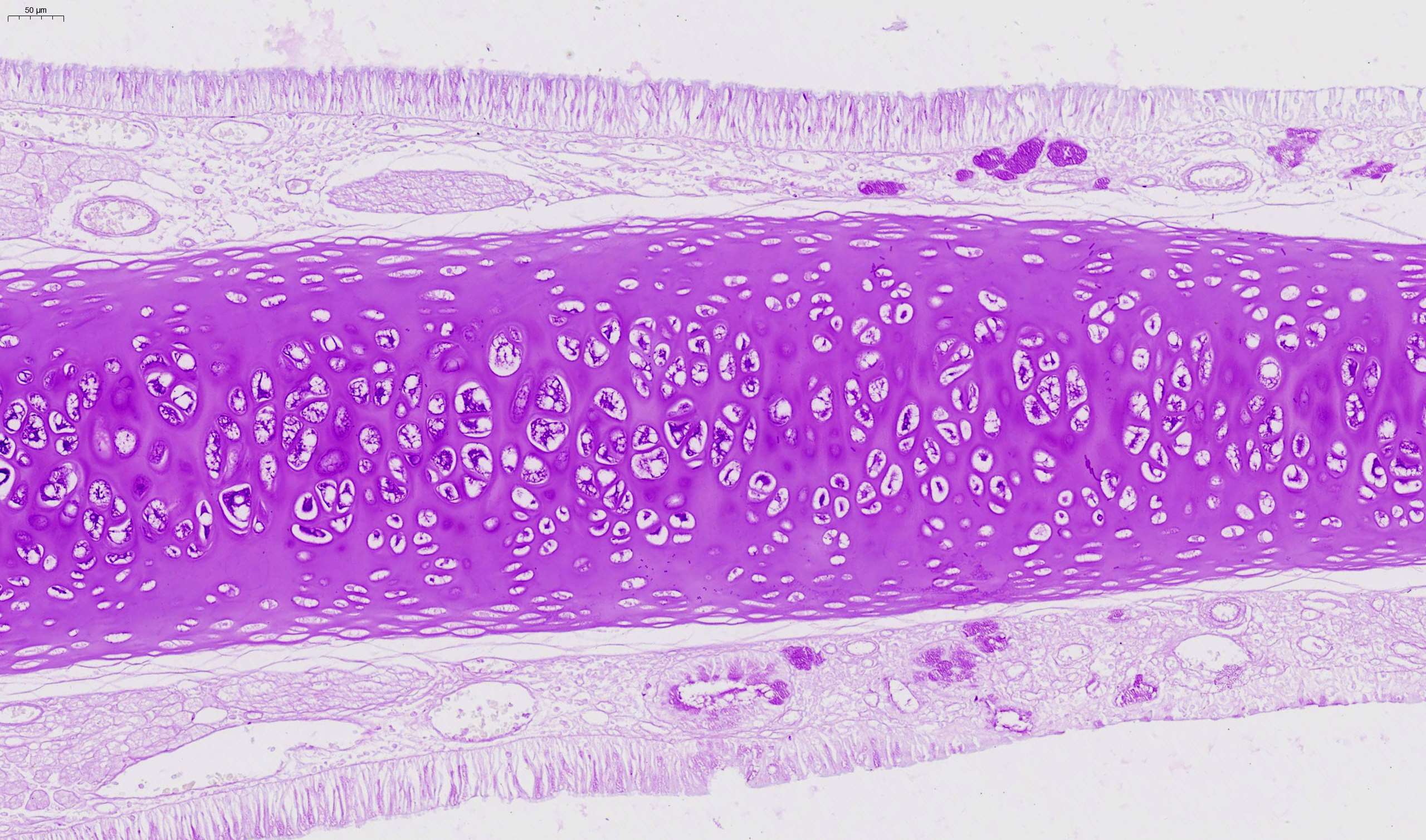

Supplement: Supplementary file 8 [file DataSheet7.ZIP › Microscopy images-PAS_200x_50um/Control/Control2 PAS_200x_50um_1.jpeg]

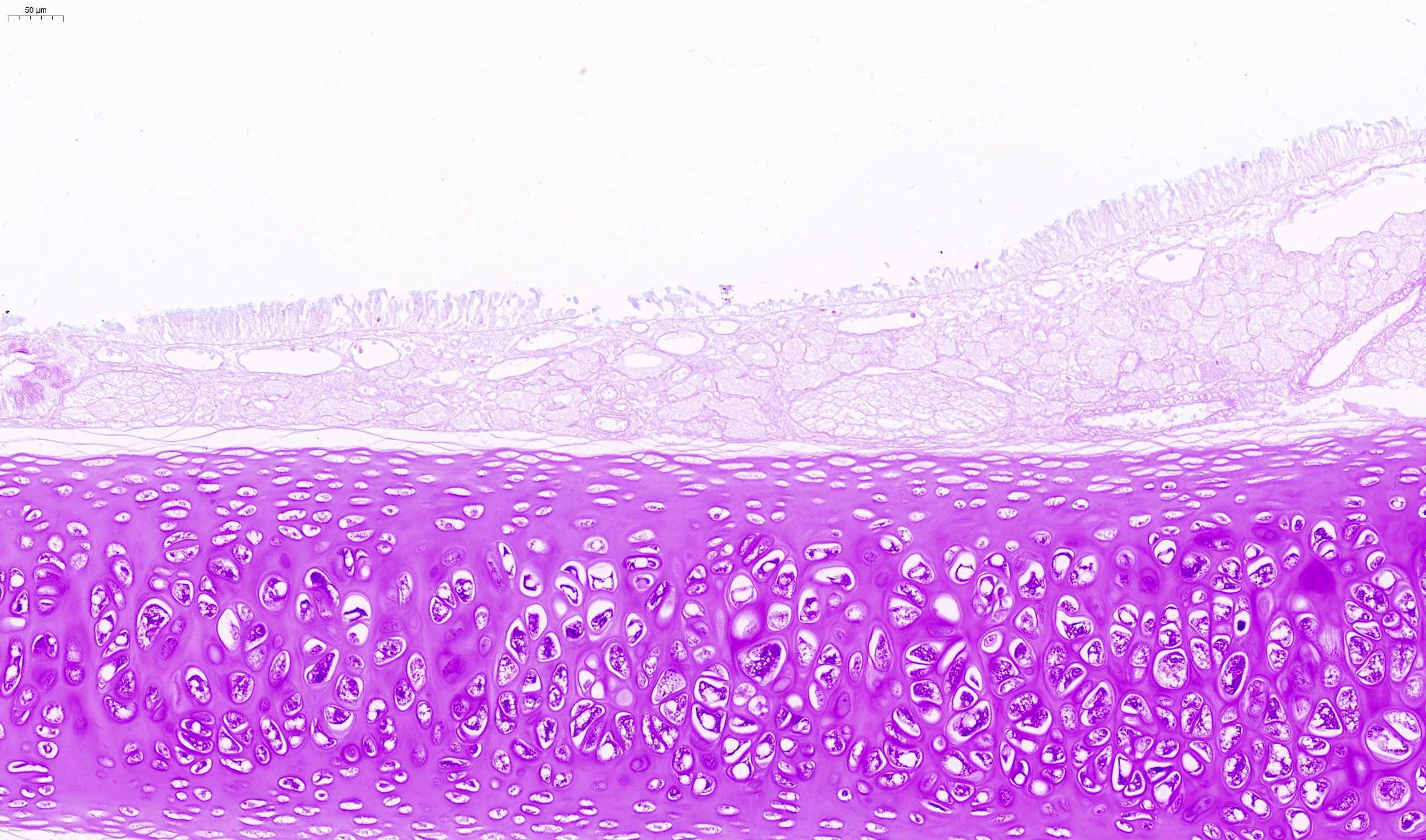

Supplement: Supplementary file 8 [file DataSheet7.ZIP › Microscopy images-PAS_200x_50um/Control/Control3 PAS_200x_50um_1.jpeg]

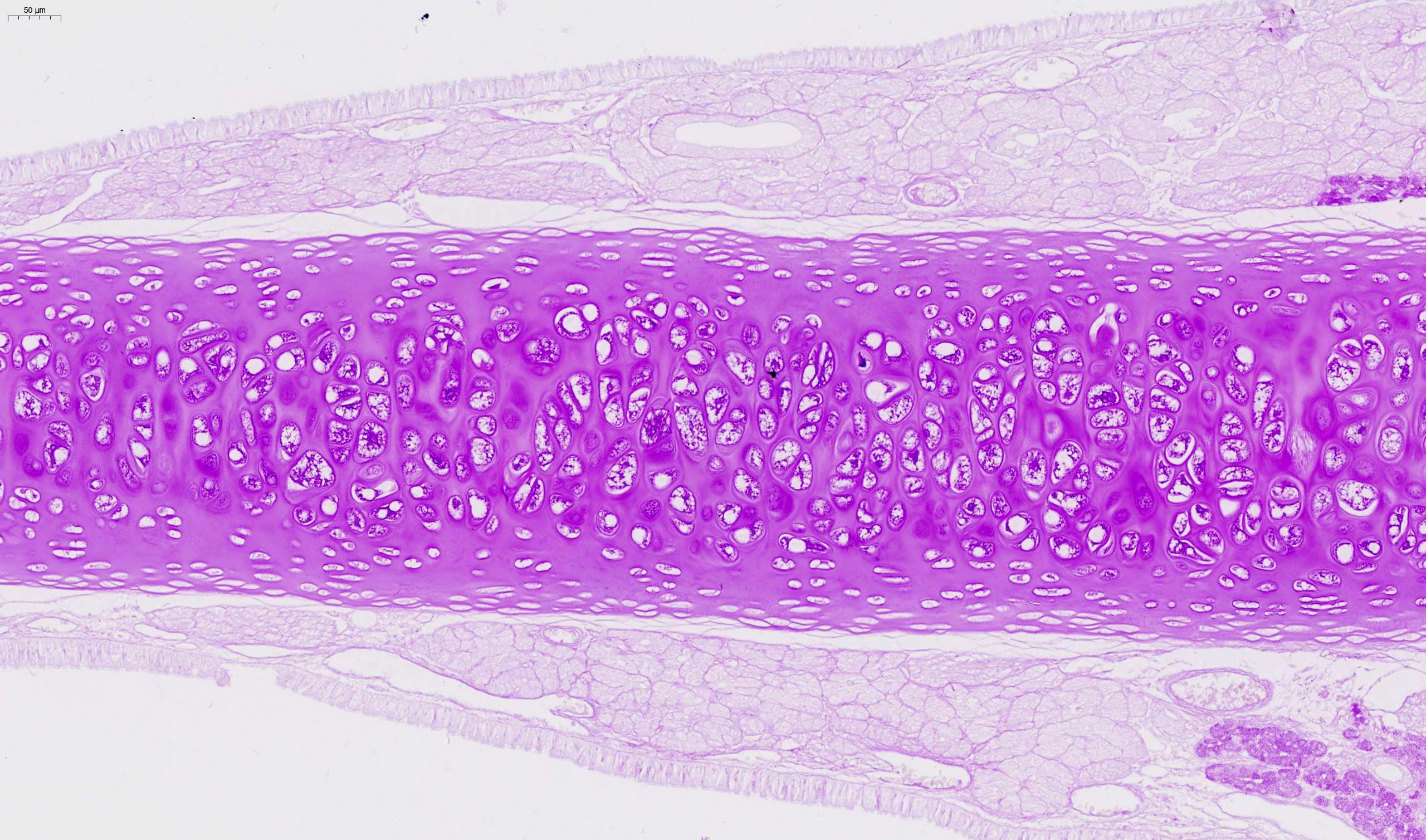

Supplement: Supplementary file 8 [file DataSheet7.ZIP › Microscopy images-PAS_200x_50um/Control/Control4 PAS_200x_50um_1.jpeg]

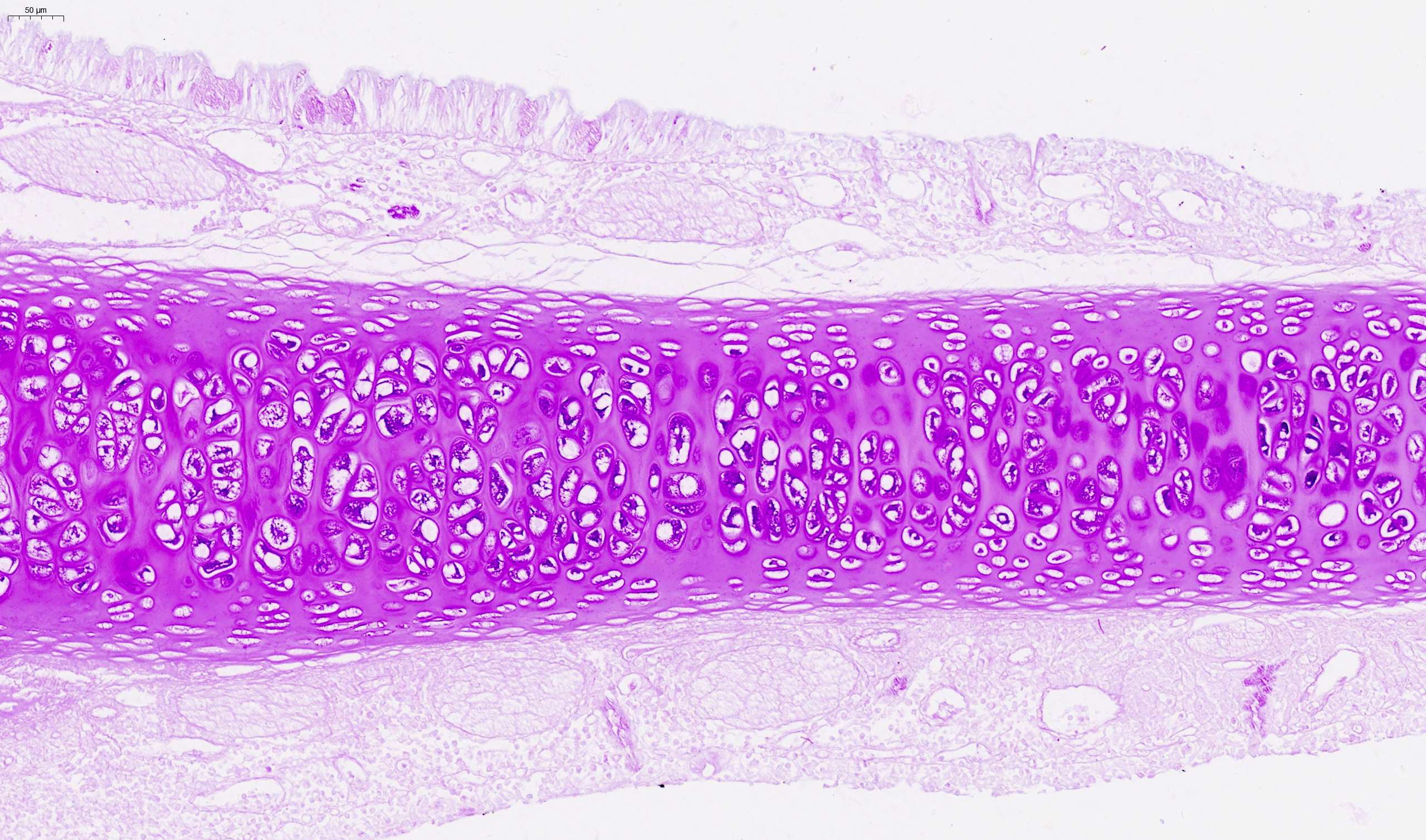

Supplement: Supplementary file 8 [file DataSheet7.ZIP › Microscopy images-PAS_200x_50um/Control/Control5 PAS_200x_50um_1.jpeg]

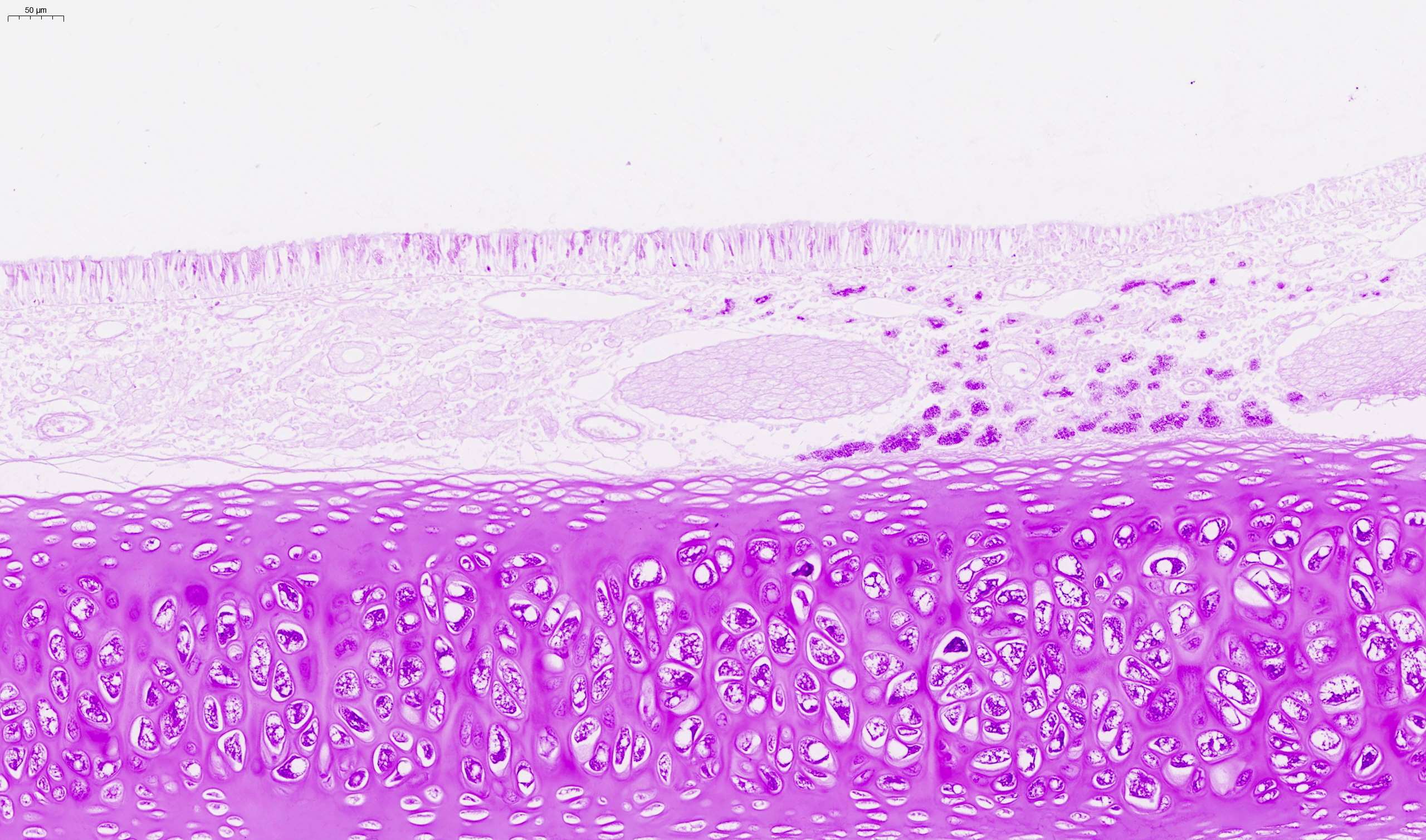

Supplement: Supplementary file 8 [file DataSheet7.ZIP › Microscopy images-PAS_200x_50um/Loratadine/Loratadine1 PAS_200x_50um_1.jpeg]

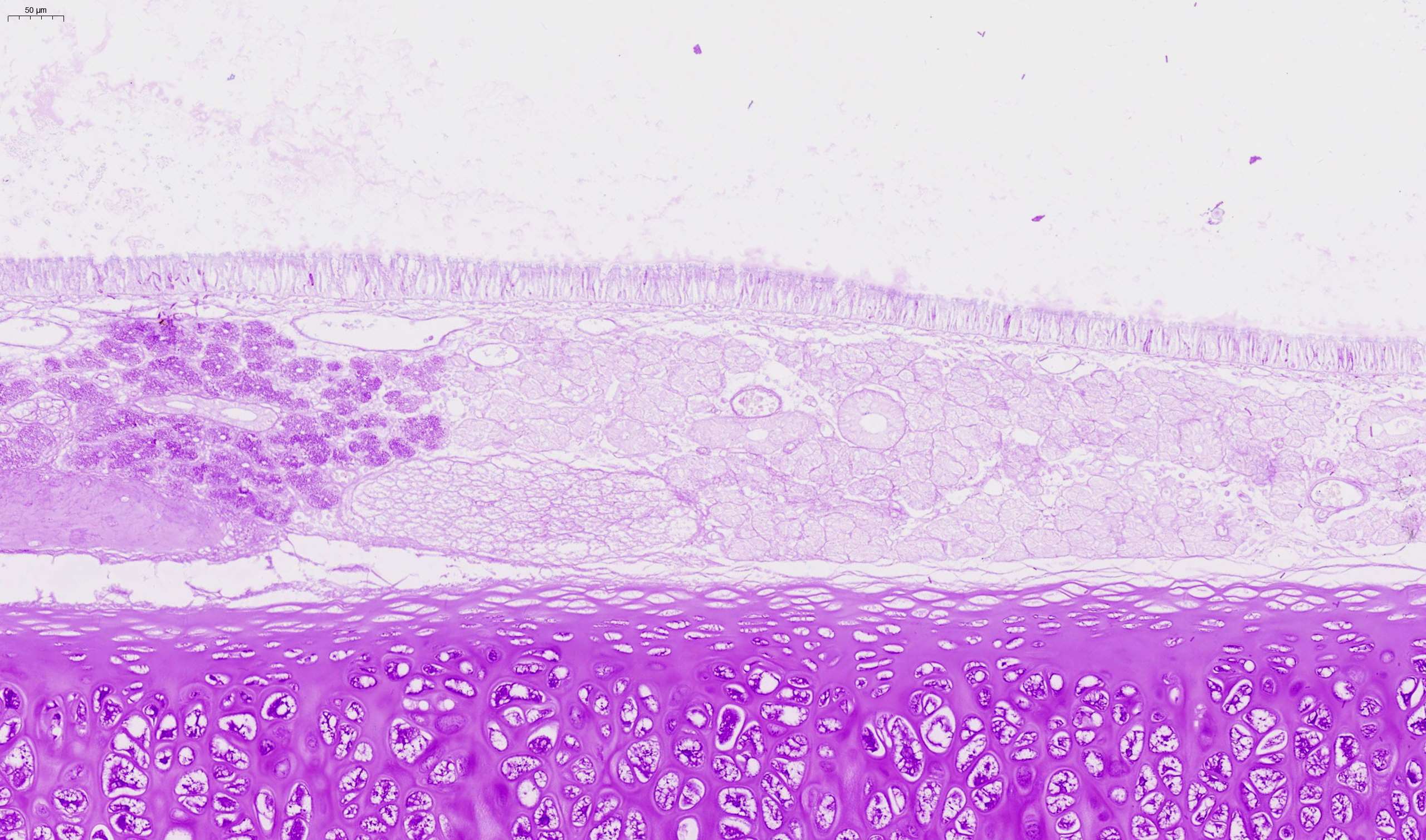

Supplement: Supplementary file 8 [file DataSheet7.ZIP › Microscopy images-PAS_200x_50um/Loratadine/Loratadine2 PAS_200x_50um_1.jpeg]

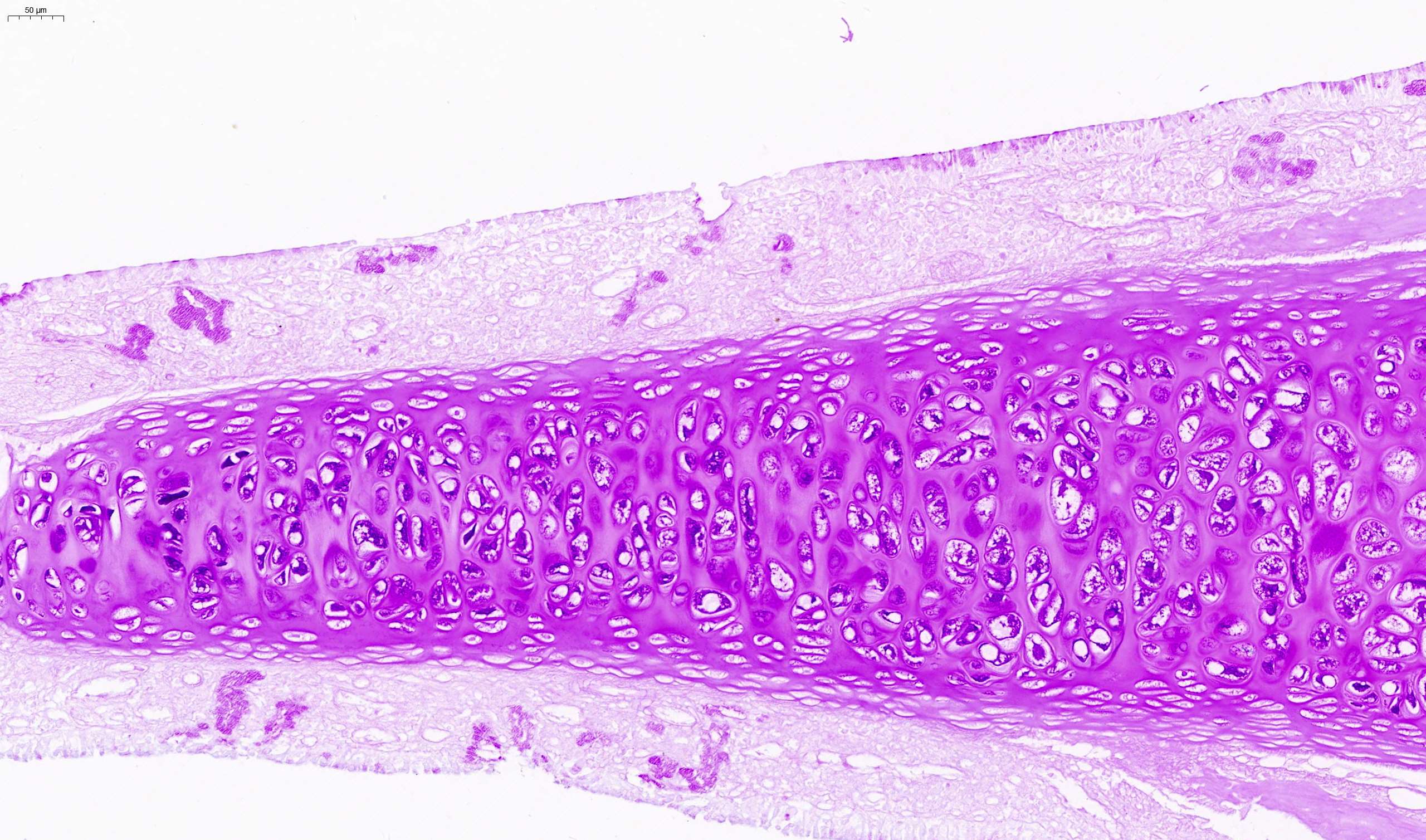

Supplement: Supplementary file 8 [file DataSheet7.ZIP › Microscopy images-PAS_200x_50um/Loratadine/Loratadine3 PAS_200x_50um_1.jpeg]

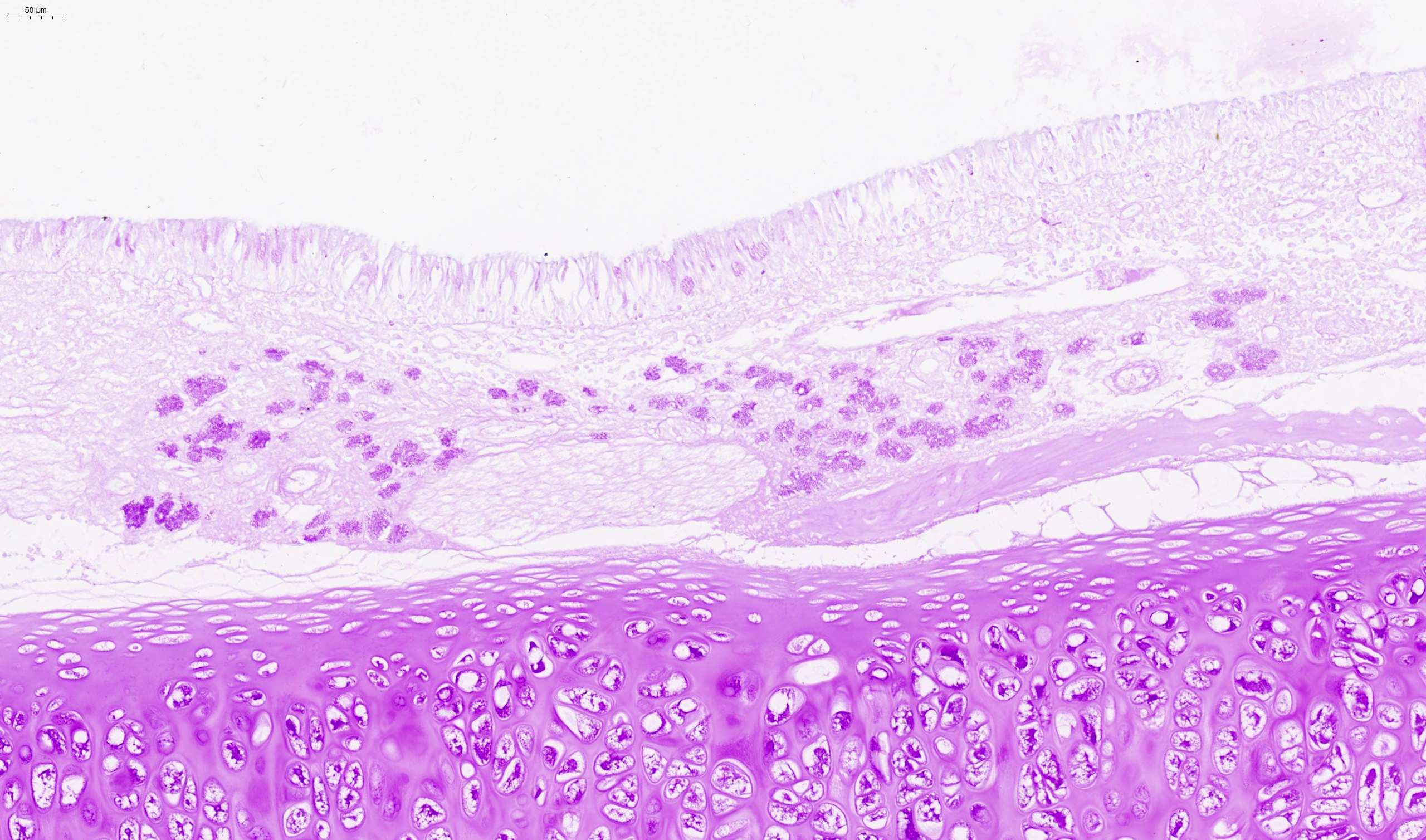

Supplement: Supplementary file 8 [file DataSheet7.ZIP › Microscopy images-PAS_200x_50um/Loratadine/Loratadine4 PAS_200x_50um_1.jpeg]

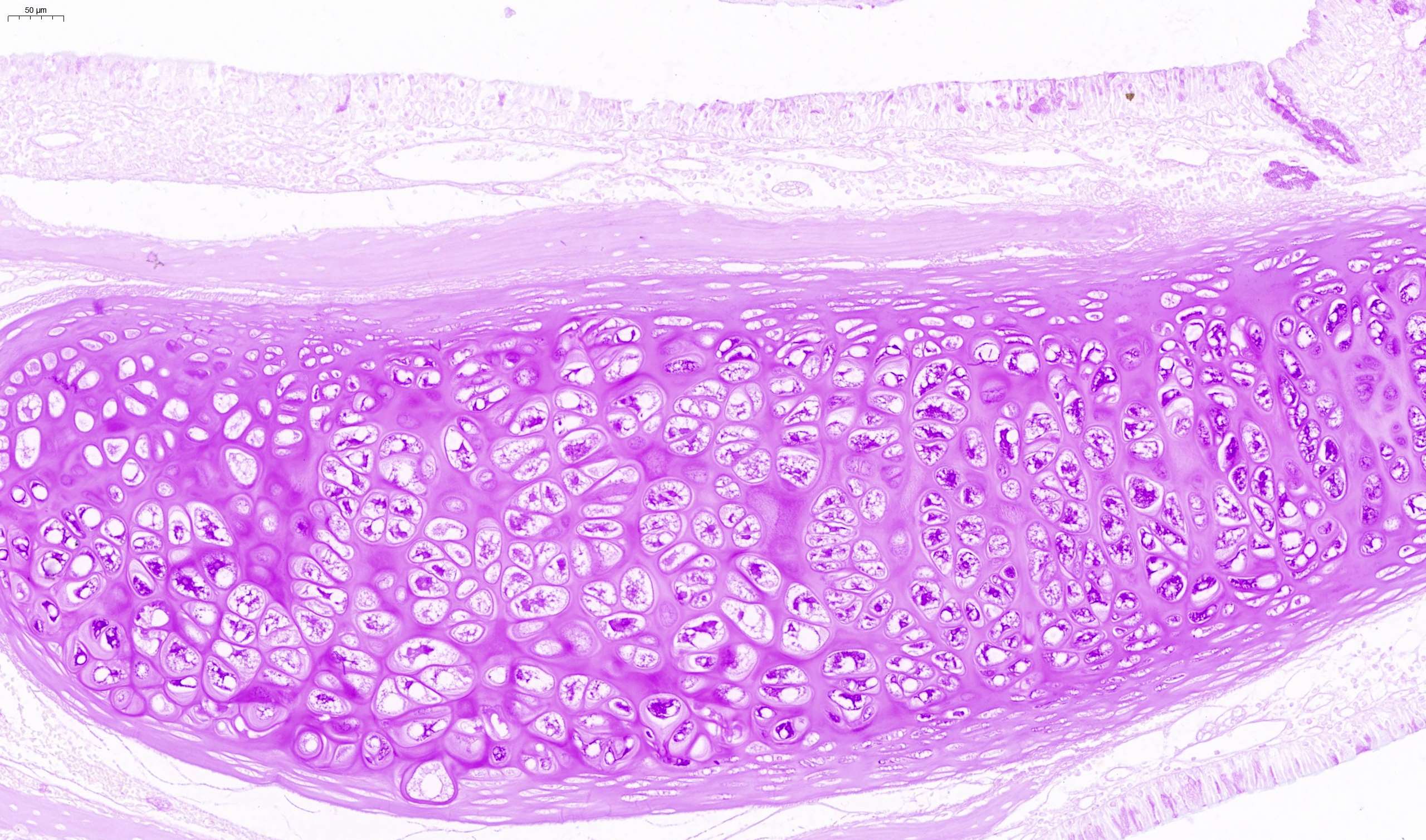

Supplement: Supplementary file 8 [file DataSheet7.ZIP › Microscopy images-PAS_200x_50um/Loratadine/Loratadine5 PAS_200x_50um_1.jpeg]

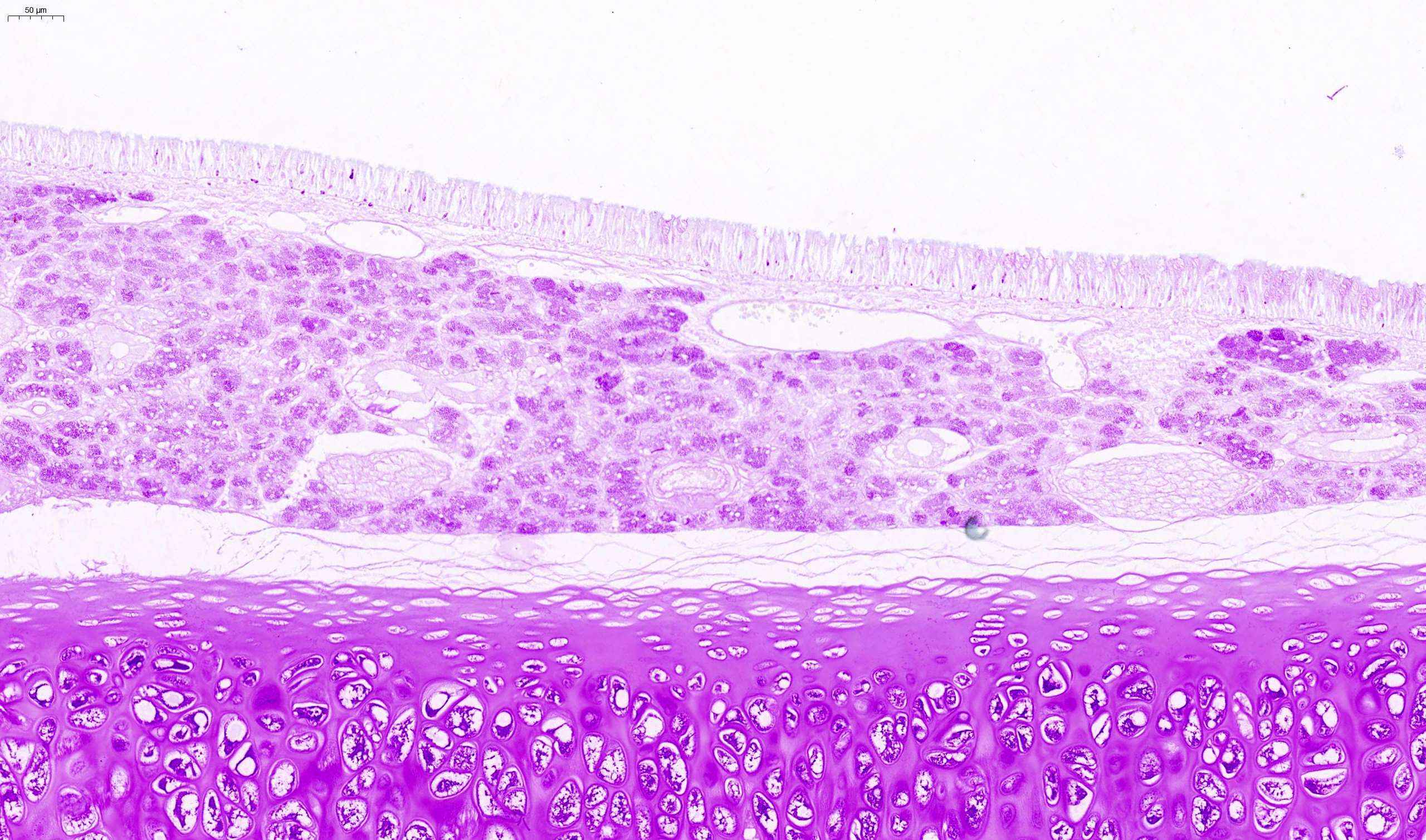

Supplement: Supplementary file 8 [file DataSheet7.ZIP › Microscopy images-PAS_200x_50um/Model/Model1 PAS_200x_50um_1.jpeg]

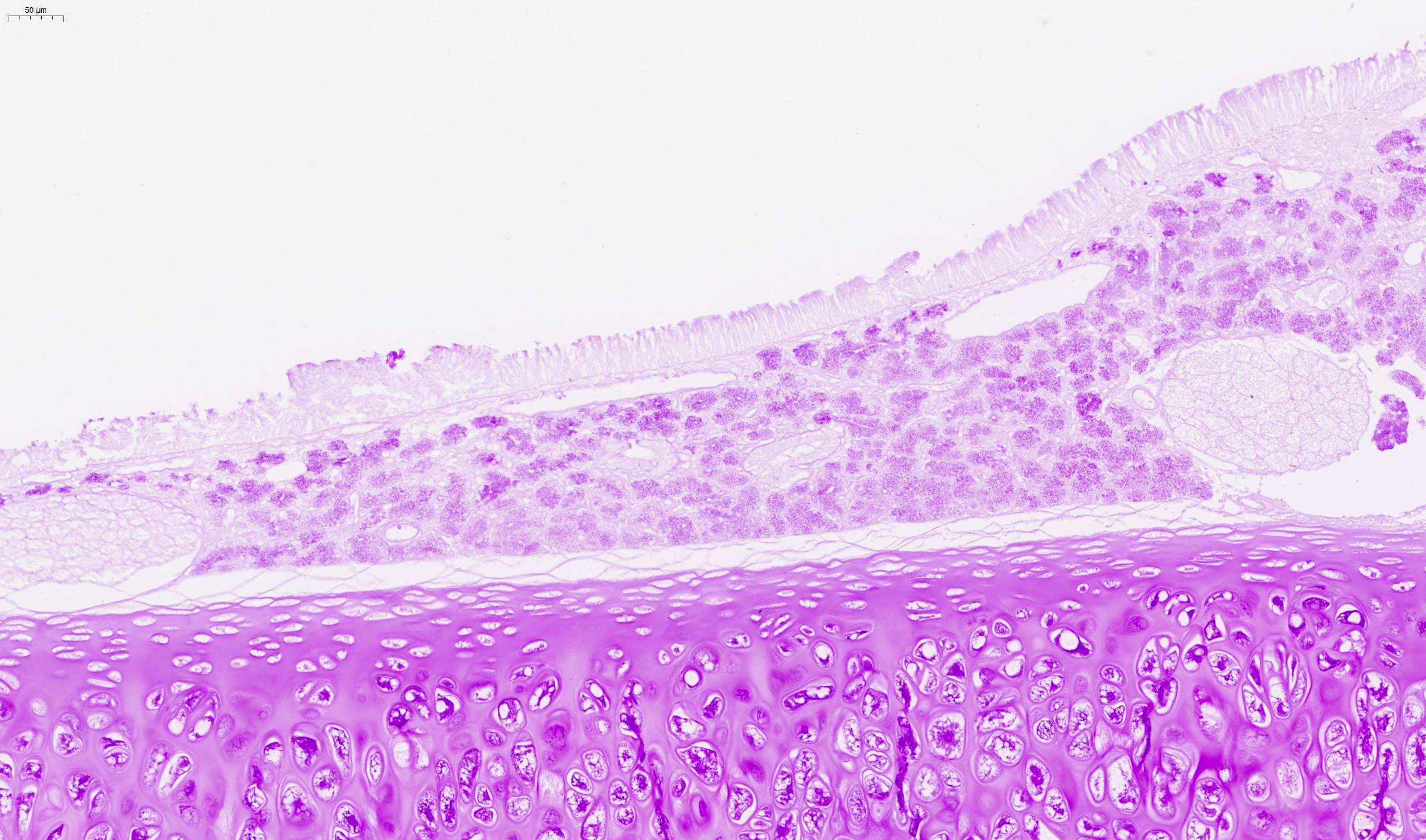

Supplement: Supplementary file 8 [file DataSheet7.ZIP › Microscopy images-PAS_200x_50um/Model/Model2 PAS_200x_50um_1.jpeg]

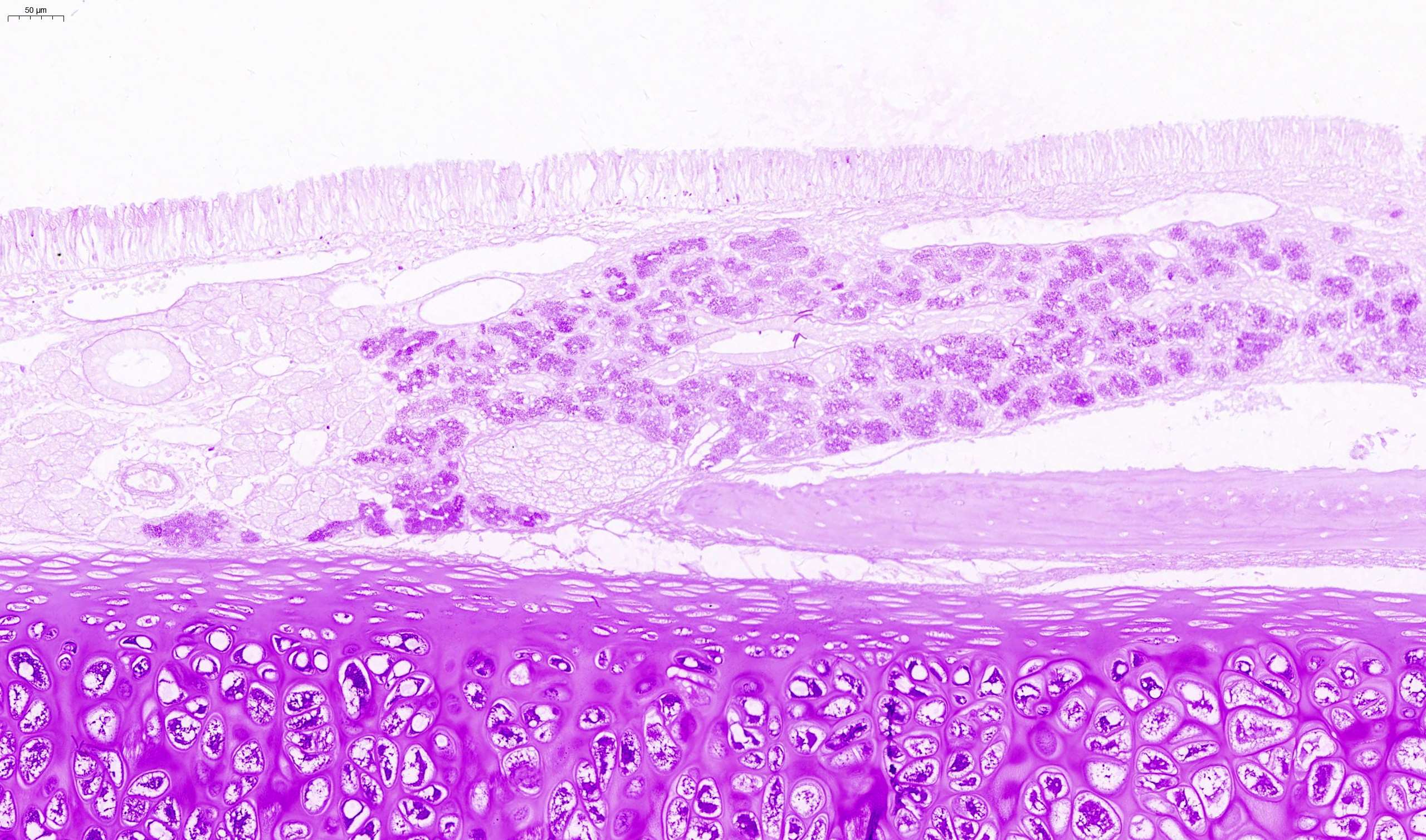

Supplement: Supplementary file 8 [file DataSheet7.ZIP › Microscopy images-PAS_200x_50um/Model/Model3 PAS_200x_50um_1.jpeg]

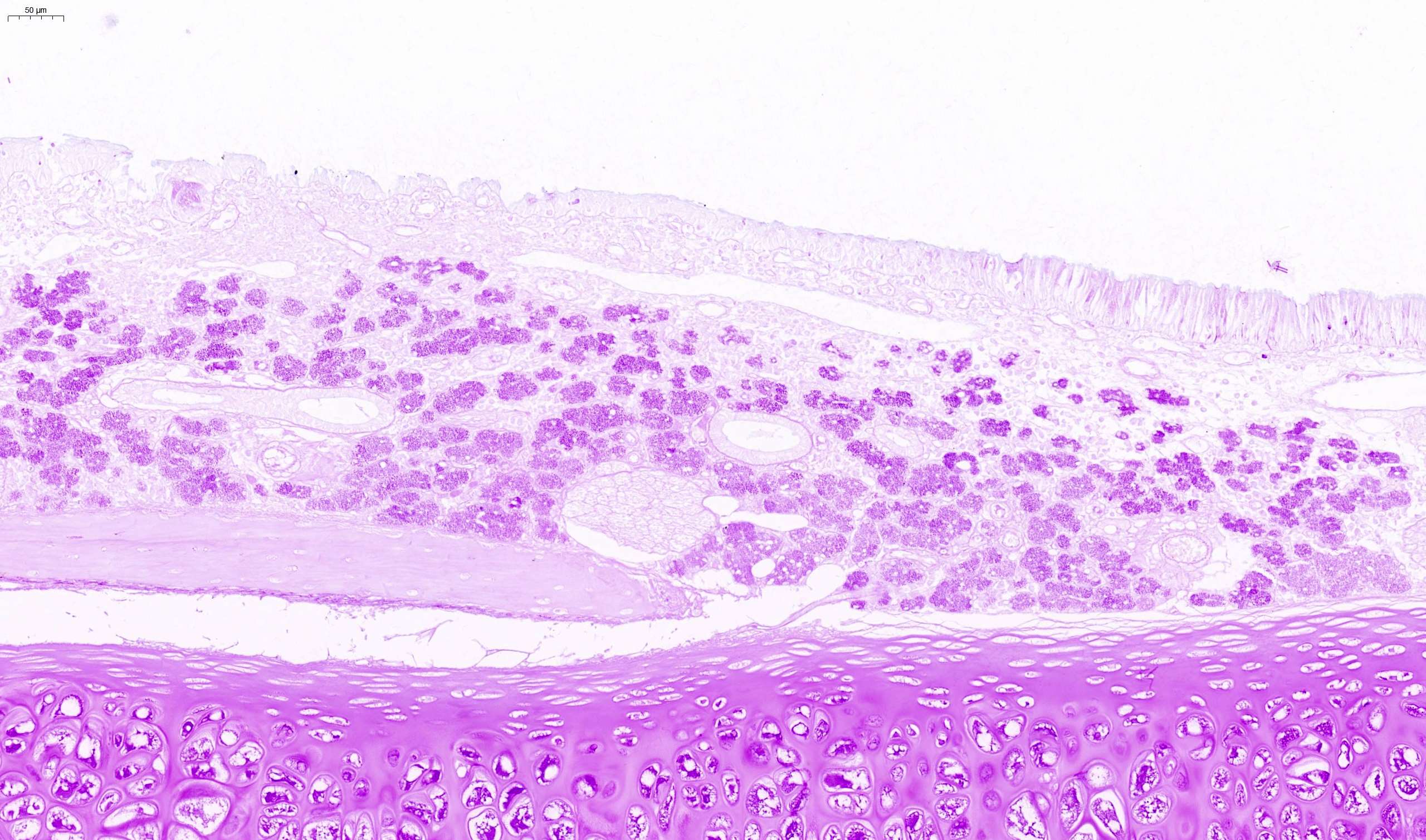

Supplement: Supplementary file 8 [file DataSheet7.ZIP › Microscopy images-PAS_200x_50um/Model/Model4 PAS_200x_50um_1.jpeg]

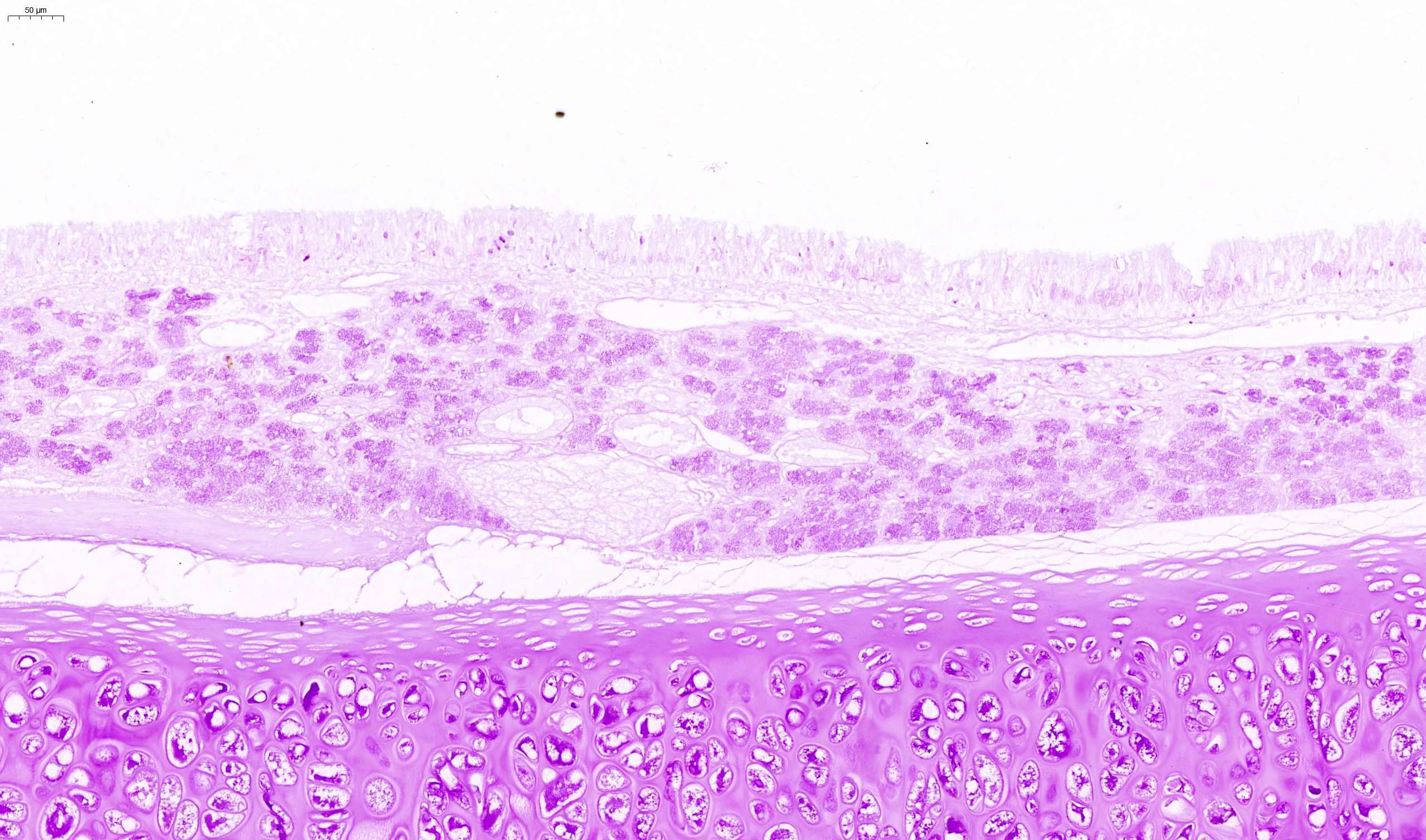

Supplement: Supplementary file 8 [file DataSheet7.ZIP › Microscopy images-PAS_200x_50um/Model/Model5 PAS_200x_50um_1.jpeg]
